# The evolution of antibiotic resistance in Europe, 1998–2019

**DOI:** 10.1371/journal.ppat.1012945

**Published:** 2025-04-03

**Authors:** Martin Emons, François Blanquart, Sonja Lehtinen

**Affiliations:** 1 Department of Environmental Systems Sciences, Institute for Integrative Biology, ETH Zurich, Zurich, Switzerland; 2 Department of Molecular Life Sciences, University of Zurich, Zurich, Switzerland; 3 Swiss Institute of Bioinformatics, Lausanne, Switzerland; 4 Center for Interdisciplinary Research in Biology, CNRS, Collège de France, PSL Research University, Paris, France; 5 Department of Computational Biology, University of Lausanne, Lausanne, Switzerland; University of Mississippi Medical Center, UNITED STATES OF AMERICA

## Abstract

The evolutionary dynamics of antibiotic resistance are not well understood, particularly the long-term trajectories of resistance frequencies and their dependence on antibiotic consumption. Here, we systematically analyse resistance trajectories for 887 bug-drug-country combinations in Europe across 1998–2019, for eight bacterial species with a considerable resistance-associated public health burden. Our analyses support a model in which, after an initial increase, resistance frequencies reach a stable intermediate equilibrium. The plurality (37%) of analysed trajectories were best described as ‘stable’ (neither increasing nor decreasing). 21% of trajectories were best described as ‘stabilising’ – i.e. showing a transition from increasing frequency to a stable plateau; 21% as decreasing and 20% as increasing. The antibiotic consumption in a country predicts both the equilibrium frequency of the corresponding resistance and the speed at which this equilibrium is reached. Moreover, we find weak evidence that temporal fluctuations in resistance frequency are driven by temporal fluctuations in hospital antibiotic consumption. A large fraction of the variability in the speed of increase and the equilibrium level of resistance remains unexplained by antibiotic use, suggesting other factors may also drive resistance dynamics. Overall, our results indicate that ever increasing antibiotic resistance frequencies are not inevitable.

## Author summary

Antibiotic resistance is an important public health threat: resistant infections are currently associated with 5 million deaths per year globally. This burden may increase further in the next decades if the frequency of resistance among important bacterial pathogens rises. There is great uncertainty about future trajectories of resistance and how to predict them. Intuitively, if resistance provides bacteria with enough of an advantage to emerge in the first place, we might expect resistance frequencies to continue to increase through natural selection, until antibiotic resistant strains replace antibiotic susceptible ones. However, when we systematically examined how resistance frequencies evolve with time, a more complex picture emerged. We analysed temporal trajectories of antibiotic resistance in Europe over two decades. The results overall support a model in which, after an initial increase, antibiotic frequencies stabilise at an intermediate frequency. We find that a country’s antibiotic consumption is associated with both the speed of the initial increase and the eventual plateau frequency. However, differences in antibiotic consumption do not explain all of the differences in resistance frequencies between countries. A better understanding of the factors that determine antibiotic resistance frequencies and lead these frequencies to stabilise is crucial for combating this threat.

## Introduction

Antibiotic resistance is a serious public health concern, with an estimated 5 million resistance-associated deaths per year globally [1]. Resistance phenotypes emerge following the introduction of new antibiotics [2], but how the frequency of these phenotypes evolves over long time frames is not well understood. Existing studies into temporal trends in resistance frequencies, often focused on specific species-antibiotic (‘bug-drug’) combinations, report a variety of resistance trajectories—including increasing, decreasing, apparently stable, and non-monotonic time courses [3–14]. A recent study looking at 13 bug-drug combinations across a large number of countries also reported a mixture of increasing and decreasing trends [15].

Describing rising and falling trends in resistance dynamics can help us anticipate short-term developments in resistance frequencies. However, over longer time-scales, considering resistance trajectories in terms of models of evolutionary dynamics can provide important additional insights. Here, we analyse observed trajectories to address a series of inter-related questions bearing on the evolutionary dynamics of resistance and how antibiotic consumption affects these dynamics.

**Fig 1:**
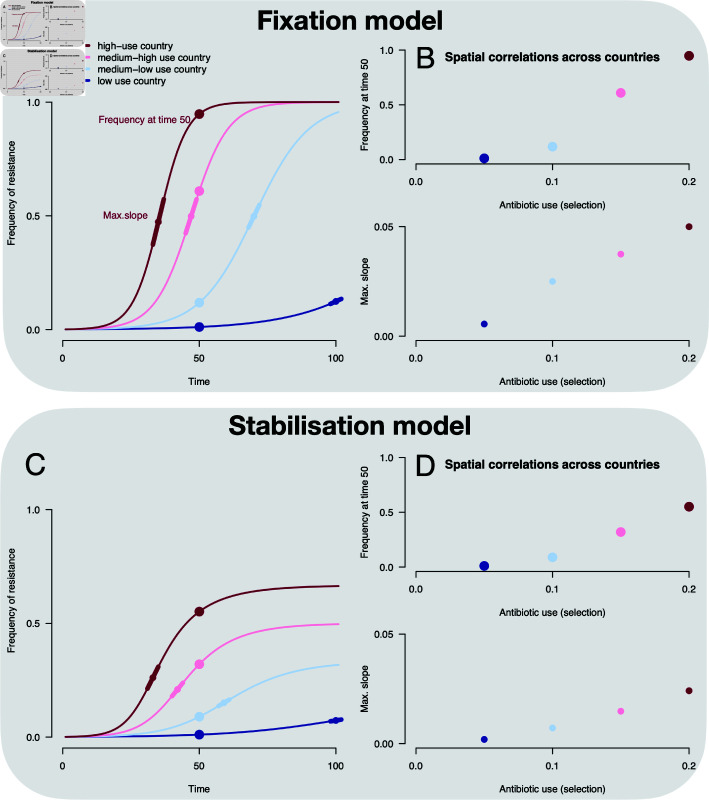
Schematic of fixation (A, B) and stabilisation (C, D) models of resistance dynamics. The different colours represent different strengths of selection pressure applied in different countries. (A) and (C) show the predicted temporal trajectories for each of the models. In the fixation model (A), resistance frequencies increase to 100% following a logistic trend, with the slope of the logistic curve reflecting the strength of selection (i.e. selection coefficient in units of time^-1^ [27]). A simple epidemiological model predicts a constant selection coefficient equal to the antibiotic consumption rate minus the cost of antibiotic resistance [16]. In contrast, balancing selection leads to a stabilising trend (C), with resistance frequencies plateauing below 100%. A number of candidate mechanisms lead to balancing selection, but no clear consensus has been reached on a definitive model. (B, D): If we suppose that different strengths of selection correspond to antibiotic use in different countries, both models generate a positive correlation across countries between the frequency of resistance (here at time 50, materialised by points on A, C) and antibiotic use, as well as between the maximum slope of resistance increase (tangents on A, C) and use.

Our first central question is the extent to which resistance trajectories are compatible with a simple evolutionary model. Intuitively, in order to emerge, resistance should provide a benefit over sensitivity. According to a simple model of resistance evolution, this competitive advantage will drive resistance frequencies to eventually reach 100% (‘fixation’). The simplest models of resistance dynamics predict a logistic rise in resistance frequencies (Fig 1), at a rate dependent on the population antibiotic consumption and the fitness cost associated with resistance, both assumed to be constant [16]. The intuition of fixation also holds for more complex models of resistance dynamics.

However, while some resistance trajectories (e.g. penicillin resistance in *Staphylococcus aureus* [4]) have indeed reached fixation, this is not the norm. A stable intermediate equilibrium frequency is possible if ‘balancing selection’ (sometimes also called ‘negative frequency-dependent selection’ although the two terms are not strictly synonymous [17]) acts to stabilise resistance frequencies at intermediate levels. The potential mechanisms giving rise to balancing selection have received considerable attention in the theoretical literature, with stabilising mechanisms suggested to arise from, for example, host population structure [18–20]; strain structure [21]; or within-host dynamics [22].

The distinction between these models is important as it determines long-term expectations about the burden of resistance. It is important to note that resistance is a significant public health concern at frequencies well below 100%. For example, antibiotic resistance led to ciprofloxacin and other fluoroquinolones no longer being recommended for treatment of *Neisseria gonorrhoeae* in the United States in 2007, when resistance frequencies for ciprofloxacin were below 15% [23]. Nevertheless, distinguishing between these models is important for informing management strategies. The lack of observed fixation is not in itself evidence of stabilisation. It is unclear whether resistance frequencies are at equilibrium; if not, resistance trajectories could still be slowly rising towards 100%. One way to resolve this uncertainty is to specifically assess evidence of stabilisation in temporal trajectories—i.e. whether trajectories exhibit a rising phase in resistance followed by a plateau, indicating the presence of balancing selection.

The distinction between the fixation and stabilisation models is also relevant for understanding the relationship between antibiotic consumption and resistance. Antibiotic consumption has been found to correlate with resistance frequencies across European countries and US states [24,25], although this signal is variable and weak. How this correlation arises is not well established. Under the fixation model, the correlation occurs because antibiotic consumption affects the *rate* at which resistance increases (Fig 1). On the other hand, under the balancing selection model, antibiotic consumption may affect the rate of increase in the rising (‘non-equilibrium’) phase but also the *equilibrium frequency* of resistance (Fig 1). The observed correlation could therefore reflect either or both of these effects. This distinction matters for predicting the impact of reducing antibiotic consumption. Under the balancing selection model, if resistance is at equilibrium, a reduction in antibiotic consumption would lead to a lower plateau. Under non-equilibrium dynamics however, a reduction in antibiotic consumption would affect the rate of frequency change: either slowing or reversing it, depending on the magnitude of the reduction.

Here we assess resistance trajectories in light of these evolutionary questions. We analyse over 3.3 million bacterial isolates sampled from invasive infections in Europe, collected by the European Centre for Disease Control and Prevention (ECDC), together with antibiotic consumption data. These data cover 30 countries, 36 antibiotics and 8 species: *Streptococcus pneumoniae*, *Staphylococcus aureus*, *Enterococcus faecalis*, *Enterococcus faecium*, *Escherichia coli*, *Klebsiella pneumoniae*, *Pseudomonas aeruginosa*, and *Acinetobacter* spp. These species can all be considered opportunistic pathogens but vary in lifestyle, from obligate human colonisers (e.g. *S. pneumoniae*) to species often found in the environment (e.g. *P. aeruginosa*, *Acinetobacter* spp.). These species are all associated with nosocomial infection; some also have a considerable burden of community-acquired disease (e.g. *S. pneumoniae*, *E. coli*); and all feature among the species for which the global burden of resistance-associated mortality is the greatest [1]. Importantly in the context of antibiotic resistance dynamics, these species are often carried asymptomatically, and are therefore exposed to “bystander selection,” i.e. selection through the full range of antibiotics prescribed for any reason, not specifically due to infection caused by the species in question [26].

We provide a quantitative and systematic view of the temporal trends in resistance and their relation to antibiotic consumption in Europe over two decades. Firstly, we provide an overview of speed and direction of resistance dynamics and assess evidence for stabilisation. Secondly, we quantify the association between antibiotic consumption and i) the equilibrium frequency of resistance and ii) the speed of increase of resistance. Thirdly, we explore the correlation between year-on-year variation in antibiotic use and resistance. We thus provide a comprehensive picture of antibiotic resistance evolution in Europe over the past two decades.

## Results

### Resistance is not systematically increasing and dynamics are slow

We used data from the European Center for Disease Prevention and Control (ECDC) to systematically investigate resistance trajectories in Europe from 1998 to 2019. In brief, the dataset consists of bacterial invasive isolates from blood and cerebrospinal fluid, tested for antibiotic resistance, with data for 30 countries, 36 antibiotics and 8 species (*Streptococcus pneumoniae*, *Staphylococcus aureus*, *Enterococcus faecalis*, *Enterococcus faecium*, *Escherichia coli*, *Klebsiella pneumoniae*, *Pseudomonas aeruginosa*, and *Acinetobacter* spp.) The population coverage of data varies by country and year, the lowest being 6% in Romania (2021), 16% in Poland (2020), to 100% in a number of countries (see Table A3.2 in [14]). Moreover, although the data include isolates from both outpatients and inpatients, the frequencies of resistance in either type of isolates were quite similar (difference in mean resistance frequency across all bug-drug-country-year: +0.035 in inpatients relative to outpatients, Pearson correlation coefficient: 0.766), and we focused on resistance in inpatients (see Methods, Preprocessing). Following data cleaning steps that filtered out trajectories with insufficient sample size, the dataset retained for analysis consisted of 887 bug-drug-country combinations (see Methods for full details).

For an overview of the temporal trends in resistance over decades, we began by fitting a standard logistic model to each trajectory (i.e. all bug-drug-country combinations). Overall, increasing trajectories were more common than decreasing ones, with 61% rising vs 39% declining. Only 29% of trajectories are significantly increasing (at the p<0.05 level, without correction for multiple testing), and 16% significantly decreasing (Fig B in S1 Text). The distinction between significant and non-significant gives an indication of the strength of support for detected trends. If looking at a specific trajectory, it is important to note that the p-values are not corrected for multiple testing (a multiple testing correction would lead to fewer trajectories being categorised as significant—i.e. decrease type 1 error but increase type 2 error). The median slope—called the ‘selection coefficient’ in the fixation model of resistance, Fig 1A—was 0.056 per year for the rising trajectories (-0.051 for the declining trajectories). For context, in the fixation model of resistance, this would translate to an increase from 1% to 99% resistance in 165 years.

### Evidence of stabilising resistance frequencies

**Fig 2:**
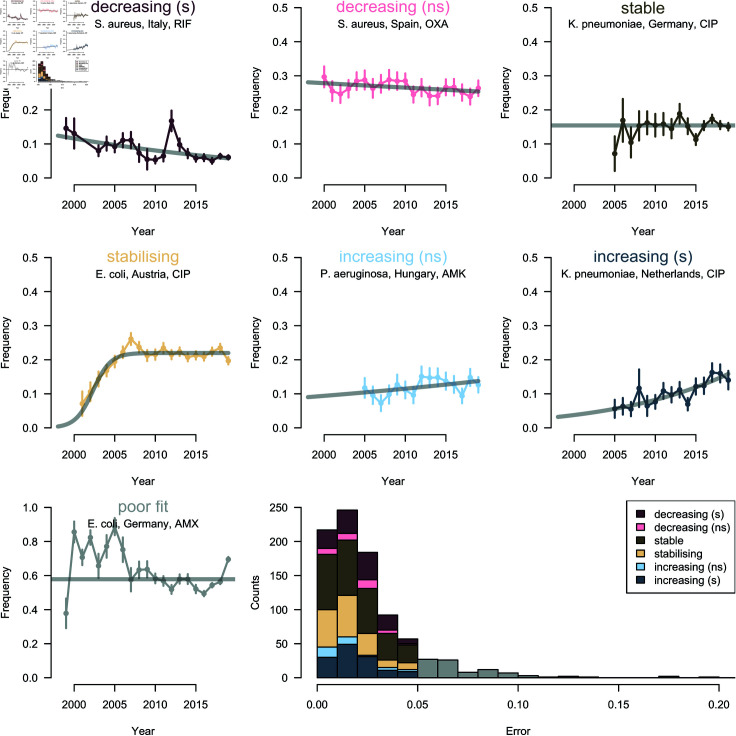
Example trajectories of antibiotic resistance illustrating the different categories. One example (a specific country-bug-drug combination) is shown for each category in the eight panels. The points are the data, the bars are 95% binomial confidence intervals. In the panel titles, ‘s’ and ‘ns’ stand for significant and non-significant (at the p<0.05 level), respectively. The last panels shows the distribution of errors for each category, for all country-bug-drug combinations. Error is quantified as the mean of the absolute value of the deviation between model predicted and actual resistance frequency. Trajectories with poor fit (i.e. error above 0.05), coloured grey on the plot, are excluded from further analysis. Abbreviated antibiotic names are the following: RIF: Rifampicin, OXA: Oxacillin, CIP: Ciprofloxacin, AMK: Amikacin, AMX: Amoxicillin.

In order to test whether resistance trajectories stabilise at an intermediate plateau, we fitted two further models to each trajectory (analysis 1 in Table 1; S1 Appendix displays all trajectories; see Methods for details and Fig A in S1 Text): a scaled logistic model, in which the frequency is increasing towards an intermediate plateau rather than fixation, and a flat straight line with slope 0. We then compared the fits of the standard logistic (2 parameters), the scaled logistic (3 parameters) and the flat line (1 parameter) models using the corrected Akaike Information Criterion (AICc), and selected the best fitting model for each trajectory. The distribution of the difference in AICc (*Δ*AICc) between the best and the second best models is presented in Fig F in S1 Text. To check how well the best-fitting model described the data, we quantified model error in terms of the mean absolute value of the deviance between predicted and observed frequency. The best-fitting model generally fit the data well (Fig 2). Nevertheless, for 10% of trajectories, this error was above 0.05, and we excluded these trajectories from further analysis.

**Table 1:** Summary of the questions addressed in this study and the corresponding analyses. The analyses are referred to with their number in the main text.

Question	Analysis
1) What is the shape of resistance trajectories?	1) Fit logistic models and choose best model based on AICc
2) Do increasing trajectories reflect non-equilibrium dynamics?	2) Compare slopes for "increasing" trajectories vs. rising phase of "stabilising" trajectories
3) Is the plateau level of resistance determined by antibiotic use?	3) Correlate across countries the plateau level of resistance vs. the use of corresponding antibiotic
4) Is the rate of increase of resistance determined by antibiotic use?	4) Correlate across countries the slope of rise in resistance vs. the use of corresponding antibiotic
5) Do year-on-year fluctuations in antibiotic use affect resistance?	5a) Correlate across years the resistance with use of corresponding antibiotic
	5b) Compare correlation across years to correlation across countries
	5c) Compare sustained trends in use vs. category of trajectory
6) Do resistances to same drug follow similar trajectories in different species?	6) Correlate across years all pairs of resistance trajectories

Trajectories best characterised by the plateauing logistic were categorised as ‘stabilising’ (21% ; here and thereafter, the percentages are computed after exclusion of the small fraction of poor fits) and those best characterised by the flat line as ‘stable’ (37%). The trajectories best characterised by the standard logistic model were further divided into non-significant (‘ns’) or significant (‘s’) trends, based on the statistical significance of the slope. This resulted in the classification of these trajectories into ‘increasing (s)’ (16%), ‘increasing (ns)’ (4%), ‘decreasing (ns)’ (5%) and ‘decreasing (s)’ (16%) categories.

**Fig 3:**
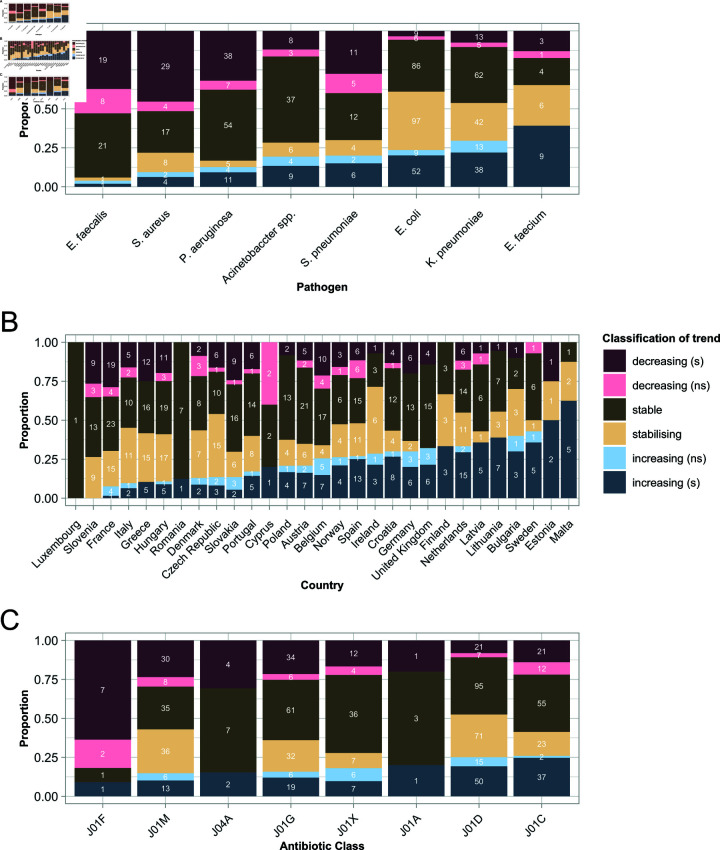
Proportion of trajectories falling into each temporal trend category, by species (A), country (B) and antibiotic class (C). The antibiotic categories are: J01A - tetracyclines, J01C - penicillins, J01D - other betalactams, J01F - macrolides, J01G - aminoglycosides, J01M - quinolones, J01X - others, J04A - drugs for treatment of tuberculosis. The numbers give the actual sample size of trajectories in each category, that is drug-country, bug-drug, bug-country for panels A, B, C respectively. This figure does not correct for correlations among the categories (e.g. some species being more frequently sampled in some countries). A corrected analysis is presented in S1 Text and is consistent with the information presented here.

Overall, these results suggest that resistance frequencies have indeed stabilised for many bug-drug combinations (Fig 3): a large proportion (67%) of the trajectories which appeared to be increasing (‘s’ and ‘ns’) in the standard logistic model were better fit by either the plateauing logistic or the flat line and therefore re-classified as ‘stable’ or ‘stabilising’. Out of the trajectories for which the standard logistic model was significantly increasing, 47% were re-classified as ‘stabilising’ (and, as expected, none as ‘stable’). This shift from significantly increasing to ‘stabilising’ was particularly striking for the species *E. coli* (58%). It is worth noting that the stabilising trends give more support to the balancing selection model than the stable trends, because the transition from increasing to stable resistance is observed for these trajectories.

The frequency of each trend varied by bacterial species, country and antibiotic class (Fig 3). To further understand the role of these factors as determinants of the trend, we performed a multivariate regression analysis (S1 Text) to estimate the effect of each factor (i.e. species, country and antibiotic class) independently of the other factors. This allows correcting for correlations among predictors—some species being more frequently sampled in some countries, for example. The results (Figs C and D in S1 Text) are consistent with the information conveyed in Fig 3.

Finally, we considered the question of whether the trajectories categorised as increasing reflect *non-equilibrium dynamics*—i.e. an increase towards a stable equilibrium. An alternative explanation for these trends would be that they reflect a *changing equilibrium* in response to changing selection pressure—due to, for example, increasing antibiotic consumption. We reasoned that looking at the rate of increase could help distinguish between these scenarios. If the increasing trajectories reflect the rise of resistance from initial emergence towards its equilibrium frequency (i.e. non-equilibrium dynamics), we would expect this rate of increase to be similar to the rate observed in the non-equilibrium phase of the stabilising trajectories. On the other hand, if the increasing trajectories reflect a changing equilibrium, we would assume the increase to be slower. This is because year-on-year variation in antibiotic usage, which would determine the rate at which the equilibrium frequency changes, is small compared to the actual level of antibiotic usage, which is assumed to determine the speed of increase after initial emergence in the stabilising trajectories.

We therefore compared the rate of increase observed in the ‘increasing (s)’ and the non-equilibrium phase of the ‘stabilising’ trajectories (analysis 2; Fig E in S1 Text). We quantified this speed as the maximal rate of increase in the time frame for which we had data (see Methods). The mean rate of increase was significantly lower for the ‘increasing (s)’ than the ‘stabilising’ category (2.2 vs 3.9 percentage points per year, $p=4.7\;\times \;10^{-4}$ in a linear regression model with ‘stabilising’ vs ‘increasing (s)’ as a predictor of the speed of increase, n = 299). The effect remains significant when additionally adjusting for species, country and antibiotic class ($p=1.9\;\times \;10^{-6}$). Thus, while some of the increasing trajectories likely reflect genuine non-equilibrium dynamics, the slower rate of change suggests some of these trajectories may instead represent resistance frequencies responding to a changing selection pressure.

### Antibiotic consumption predicts the level of stabilisation and the rate of increase of antibiotic resistance

Next, we investigated which properties of the temporal trajectories of antibiotic resistance are predicted by antibiotic use. Focusing on bug-drug combinations for which we had data from at least five countries, we examined the correlation (Spearman’s rank correlation) between a country’s median antibiotic use over years and i) the plateau frequency of resistance (analysis 3) and ii) the maximum rate of increase of resistance (analysis 4), computed as above. The correlation with the plateau frequency was performed for the ‘stable’ and ‘stabilising’ trajectories—the plateau frequency is not defined for the ‘increasing’ and ‘decreasing’ categories, because, if these categories correspond to non-equilibrium dynamics, we do not know at what frequency these trajectories might eventually plateau. The correlation with the rate of increase was performed for the ‘increasing (s)’ and ‘stabilising’ categories.

**Fig 4:**
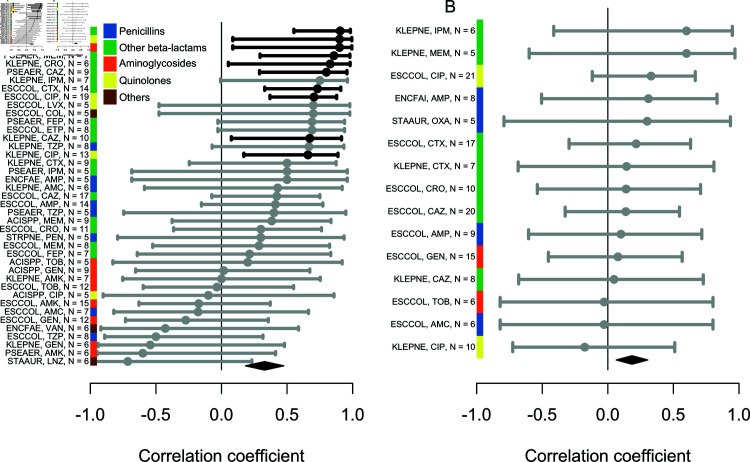
Correlation (Spearman’s rho) across countries between (A) the plateau frequency of antibiotic resistance, or (B) rate of increase, and the rate of use of the corresponding antibiotic in the community, for all bug-drug combinations. Significantly positive correlations at the 0.05 level are shown in black, others in grey. The abbreviations on the y-axis indicate the species (ACISPP = *Acinetobacter* spp, ENCFAE = *Enterococcus faecalis*, ENCFAI = *Enterococcus faecium*, ESCCOL = *Escherichia coli*, KLEPNE = *Klebsiella pneumoniae*, PSEAER = *Pseudomonas aeruginosa*, STAUR = *Staphylococcus aureus*) and antibiotic (see Methods for meanings) each correlation is for. The *N* number indicates the number of countries included for each combination. The black diamond shows the overall mean and 95% confidence interval. Trends are similar when considering hospital instead of community use (Fig I in S1 Text).

We observed a positive correlation between the plateau level of resistance and the consumption of the corresponding class of antibiotics in the community for most bug-drug combinations (Fig 4A). Overall, the mean correlation across all combinations was significantly greater than 0 (mean correlation coefficient ρ=0.33[95%CI:0.18;0.48], N=40 combinations, t-test on the distribution of correlations, $p=5.2\;\times \;10^{-5}$). Several individual coefficients were also significantly positive, despite considerable uncertainty associated with the estimates. This also held true when considering antibiotic use in the hospital instead of in the community (Fig I in S1 Text); mean ρ=0.32[95%CI:0.17;0.48], N=31 combinations, t-test $p=1.7\;\times \;10^{-4}$). This is consistent with selection by antibiotics increasing the plateau level of resistance.

Second, the rate of increase of resistance frequency also correlated with antibiotic use, but the signal was weaker than for the plateau (Fig 4B). This weaker signal may be explained by the estimates of the slope parameter being associated with greater uncertainty than the estimates of the plateau parameter, relative to the variability of the parameters across countries (Fig G in S1 Text). None of the correlations for individual bug-drug combinations were statistically significant (Fig 4B), but overall the set of correlation coefficients had a mean significantly greater than 0 (mean ρ=0.18[95%CI:0.06;0.30], N=14 combinations, t-test p=0.005). The mean was similar when considering antibiotic use in the hospital sector, though not significantly different from 0 (mean ρ=0.19[95%CI:−0.08;0.45], N=10 combinations, t-test p=0.15) (Fig I in S1 Text). Because the rate of increase in the ‘increasing (s)’ trajectories may not reflect non-equilibrium dynamics, we repeated the analysis considering only ‘stabilising’ trajectories. This resulted in very few bug-drug combinations, most of them from *E. coli* and showing a positive correlation (Fig J in S1 Text). All in all, there was evidence that the frequency of antibiotic resistance tends to increase faster in countries where antibiotic use is larger.

### Antibiotic consumption as a predictor of temporal variation in antibiotic resistance

**Fig 5:**
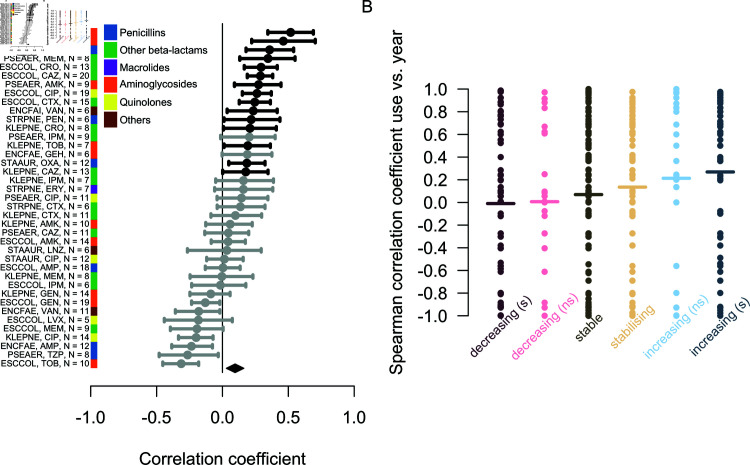
Temporal variation in hospital antibiotic use as a predictor of temporal variation in antibiotic resistance. (A), the correlation across years (Spearman’s rho) between the frequency of resistance and hospital antibiotic use in the same year across bug-drug combinations. Correlations across years significantly positive at the 0.05 level are shown in black, others in grey. The number of countries included is indicated for each combination. The black diamond shows the overall mean and 95% confidence interval. (B), the distribution of temporal trends in antibiotic consumption in hospitals for each category of trend in resistance. The horizontal line shows the mean.

Following the earlier result that increasing trajectories may reflect a changing equilibrium frequency, we investigated whether year-to-year variation in antibiotic use could cause corresponding year-to-year fluctuations in the level of resistance. For all categories of temporal trajectory, we computed the correlation between antibiotic resistance and consumption across years, for bug-drug-country combinations that had at least five years of data (analysis 5a). For each bug-drug combination, we averaged these temporal correlation across countries, retaining only bug-drug combinations for which we had at least five countries. There was overall a weak positive mean correlation between resistance and antibiotic use in the hospital sector (mean ρ=0.1[0.03;0.16], N=38 combinations, t-test p=0.0054) (Fig 5A), but this was not true when considering the use of antibiotics in the community sector (mean ρ=−0.02[−0.07;0.04], N=46 combinations, t-test p=0.52) (Fig L in S1 Text).

Because of the mixed evidence, to further investigate the possibility that the frequency of resistance follows the fluctuations in antibiotic use, we did a series of complementary analyses, all confirming our finding.

First, we computed an additional partially independent indicator comparing correlations across years with correlations across countries (analysis 5b; Fig M in S1 Text) [28]. Correlations across years were consistent with correlations across countries for hospital sector, but not community use, also suggesting that antibiotic resistance follows temporal fluctuations in the use of antibiotics in the hospital sector, but not in the community.

Second, we looked at how sustained trends in antibiotic consumption differed between temporal trajectories (analysis 5c). We computed the temporal trend in antibiotic consumption as the Spearman correlation between calendar year and consumption level (Fig 5B). We then tested whether decreasing trajectories are associated with decreasing antibiotic consumption and increasing trajectories with increasing consumption. The hospital antibiotic consumption rate was increasing on average for all resistance trend categories. The increase in consumption was significantly higher for the increasing (s and ns) than decreasing (s and ns) categories (Wilcox test, p=0.002), and the mean trends in consumption were consistently increasing from decreasing to increasing temporal trend categories. Again, no significant trend was found when considering community instead of hospital consumption (Wilcox test, p=0.14).

Last, we investigated the correlation between pairs of resistance trajectories, independently of antibiotic consumption (analysis 6). For each country, we examined cross-years correlations in all pairs of bug-drug combinations. We found that while bug-drug pairs of distinct bug and drug did not particularly correlate, bug-drug pairs of the same bug or of the same drug positively correlated (Fig N in S1 Text). This indicates that distinct resistances in the same bacterial species exhibit similar temporal trends, perhaps due to genetic linkage. Moreover, resistances to the same drug exhibit similar fluctuations in different species, suggesting they are driven by the same underlying factor such as hospital antibiotic use. This supports that temporal fluctuations in use may impact resistance fluctuations.

All in all, the frequency of antibiotic resistance follows changes in the use of the antibiotic—either year-on-year fluctuations and/or sustained trends—in the hospital, but not community, sector.

## Discussion

Our analysis of resistance trajectories in Europe suggests that antibiotic resistance frequencies are not consistently increasing and instead often appear stable over two decades (Table 1). This observation has been previously discussed in the context of *S. pneumoniae* [18,19,21,22] and, to a more limited extent, in other species [20,22]. Our work provides systematic evidence for stability and—notably—stabilisation of resistance in eight important bacterial species.

However, a non-negligible minority of trajectories were categorised as either significantly increasing (16%) or significantly decreasing (16%). These trajectories could reflect either non-equilibrium dynamics or a changing equilibrium frequency in response to changing antibiotic use. While we cannot determine for certain which is the case for individual trajectories, our analysis suggests a mix of these scenarios.

Our approach is motivated by questions about the long-term evolutionary dynamics of resistance and focuses on systematically fitting relatively simple models to the longest available time-span across a broad range of bug-drug-country combinations. Thus, an ‘increasing’ trend means that the trajectory is compatible with a model in which resistance frequencies are increasing over the long term—as opposed to one where we see evidence of stabilisation. This approach is complementary to other approaches focusing on shorter time spans, which give insights into recent trajectories. The ECDC, notably, produces yearly reports [14] mainly focusing on resistance data from the last year, and additionally reporting increasing, decreasing or stable trends over the last few years (typically five years) for each bug, drug and country. These reports provide detailed information on resistance in each country, which is especially valuable to monitor recent trends in the most preoccupying and the emerging resistances. However, in contrast to what we do here, these reports do not analyse trends over decades, fit linear trends but not more complex shapes, do not synthesise results across bug-drug-country combinations, and do not attempt to correlate variability in resistance frequency with antibiotic use.

There was considerable variability in trends by bacterial species, country, and antibiotic class. We were able to explain some of this variability. First, resistances were in part associated with the rate of use of the corresponding antibiotic. In addition to previous work showing an association between median consumption and median resistance [24,25] (Fig K in S1 Text), we evidenced the effect of antibiotic consumption on both the rise and the stable level of resistance. We also investigated the *temporal* correlation between antibiotic use and resistance frequency. Resistance was weakly associated with hospital—but not community—antibiotic consumption, both in terms of year-on-year fluctuations and sustained trends. While the analysed resistance frequencies were from hospital inpatients, this is unlikely to explain why the association is seen with hospital, but not community, consumption: resistance in inpatient and outpatient was highly correlated, suggesting common evolutionary dynamics in hospital and community populations. Thus, it might be that resistance is more strongly driven by hospital than community consumption. This is supported by recent work using ECDC data on antibiotic resistance and consumption to parameterise a transmission model of cephalosporin- and carbapenem-resistance in *Klebsiella pneumoniae*. The modelling suggested that changes in hospital consumption had a greater effect on resistance levels than community consumption because higher *per capita* antibiotic use and higher transmission in hospital settings led to a disproportionate effect on overall resistance in this species [29]. While this could potentially explain why we see a correlation with hospital and not community consumption when looking at year-on-year variation, it remains unclear why resistance correlates with both community and hospital consumption across countries. We note that community and hospital use correlate across countries, and to a lesser extent across years within countries (Fig O in S1 Text). This complicates the identification of the impact of the two contexts of use on resistance.

Second, the species *E. coli* and *K. pneumoniae* were associated with a particularly large number of increasing or stabilising trajectories. This might be linked to the emergence and spread of the CTX-M beta-lactamase enzyme in the late 1990s and early 2000s, which confers resistance to beta-lactams and can be associated to other resistances like fluoroquinolones [30].

A large part of the variability in resistance within and across countries remained unexplained. One reason could be that differences in host population structure, and heterogeneity in antibiotic consumption *within* this structure, can lead to different resistance frequencies for populations with the same average consumption [20]. A second explanation is the spillover of resistance from neighbouring countries: even small amounts of migration from other countries are sufficient to influence local levels of resistance, which would tend to weaken the correlation across countries [19,31]. Thirdly, the association of multiple resistance genes on bacterial strains (‘linkage disequilibrium’) [32] means that the frequency of resistance against a particular antibiotic could be affected by the consumption of other antibiotics. Finally, a number of studies have reported statistical associations between the frequency of resistance and other factors—e.g. temperature, pollution, governance- and infrastructure-related factors [15,33,34]. While this suggests selection for resistance is not solely dependent on antibiotic consumption, it is challenging to determine which factors are truly causal. Determining the causal role of factors beyond average antibiotic consumption requires detailed epidemiological and genomic data, including host metadata (host characteristics, geographic location), contact networks, and sequence data conveying the genetic coupling between different resistances and other loci determining important bacterial phenotypes.

One important limitation of our study is data quality and consistency of sampling and reporting across countries. The tested isolates do not necessarily reflect a random sample of bacteria circulating in the human population, or even of bacteria infecting hospital patients. For example, hospital outbreaks might cause the over-representation of particular clones in a specific country and year. Contributing hospitals may change from year to year. Resistance frequencies are not comparable across years if the threshold for resistance is changed, as was the case in France for *P. aeruginosa* [35]. Such changes in data collection might have caused some of the systematic decreasing or increasing trends in resistance, if they unfolded over the two decades covered by our study. The antibiotic consumption data is subject to similar data quality considerations. To limit the inclusion of biased or poor-quality data in our analyses, we did not consider the trajectories for which none of our models fitted the data well (‘poor fit’ category) which may partly reflect biases or data quality issues, and we removed temporal outliers from the consumption data. Despite these concerns, most trajectories were quite smooth temporally (Fig 2). Furthermore, while potential biases will affect the overall estimate of resistance frequency, if these biases remain constant in time, they would not affect the detection and categorisation of temporal trends.

The data from the ECDC may represent one of the largest and best standardised datasets on antibiotic resistance and consumption but there are limitations to the generality of the conclusions that can be drawn from our analysis. Firstly, the dataset include only European countries. It is important to determine whether the pattern of stability and stabilising also holds elsewhere, particularly in low and middle income countries (LMICs). Data sources covering a wider range of countries (e.g. ResistanceMap [36] and the ATLAS database [37]) have limited temporal span for LMICs. Thus, while quantifying overall resistance trends in LMICs is possible [15], assessing evidence for stabilisation may prove more challenging. Secondly, while many species for which the burden of resistance is the highest [1] are included in ECDC data, some species where resistance represents a significant public health problem are not included—e.g. *Mycobacterium tuberculosis*, *Enterobacter spp.*, *Salmonella spp.* [1] and *Neisseria gonorrhoeae* [38]. In spite of potentially vastly different epidemiology, these species also often exhibit qualitatively stable or stabilising trends (*M. tuberculosis*: [39]; *Salmonella*: [40]; *N. gonorrhoeae*: [41–43]). It largely remains unexplored how the rise, plateau and potential fluctuations of these trajectories depend on antibiotic use in hospitals, in the community, or in non-human compartments.

Finally, the stability of resistance trends may not be robust to evolutionary innovation. With sustained selection pressure, bacteria may evolve new and less costly resistance mechanisms and compensatory mutations that alleviate the cost of resistance. This would result in the progressive increase of the equilibrium level of resistance. Compensatory evolution is readily observed under laboratory conditions, but its contribution to real-word resistance dynamics is unclear for the pathogens studied here.

In conclusion, using antibiotic resistance data from 3,375,774 bacterial isolates from invasive infections in hospitals, encompassing 8 bacterial species among those causing the largest burden of resistance, 30 European countries, and 20 years, we present a systematic analysis of long-term resistance trajectories, motivated by fundamental evolutionary questions. We show that resistance trajectories are for the most part stable or stabilising. Observed resistance dynamics were, to some extent, explainable by levels of antibiotic consumption, both in terms of the level at which resistance frequencies stabilised and the rate at which this plateau was reached. Taken together, these findings suggest that ever increasing resistance frequencies are not inevitable and highlight the potential impact of curbing antibiotic consumption in resistance management. Yet, the explanatory power of antibiotic consumption was relatively low, highlighting important gaps in our understanding of resistance dynamics. In spite of unexplained variability, we suggest that the trajectory of resistance frequencies in the human population follows in many cases a sigmoidal shape with an intermediate-frequency, robust, plateau. This specific trajectory could be used as a phenomenological model to describe resistance evolution, helping to monitor and characterize the emergence of new resistances.

## Methods

### Data

#### Resistance raw data.

The data on the antimicrobial resistance frequencies are released by the European Surveillance System - TESSy, provided by Finland, Sweden, Belgium, Germany, Greece, Ireland, Italy, Luxembourg, Netherlands, Norway, Portugal, United Kingdom, Austria, Bulgaria, Czech Republic, Denmark, Estonia, Spain, Malta, Slovenia, France, Croatia, Hungary, Poland, Slovakia, Romania, Cyprus, Latvia, Lithuania and released by the European Centre for Disease Prevention and Control (ECDC). The data cover the years 1998-2019.

The raw data, available upon request from the ECDC, is in case-based format, and describes the results of antimicrobial susceptibility testing conducted in hospitals for invasive isolates of the eight bacterial species listed below, isolated from the blood and cerebrospinal fluid of outpatients and inpatients. More precisely, the data consist of, for each isolate: the date of the isolation, the pathogen species, the patient type (inpatient or outpatient), and the antimicrobial resistance level (sensitive, intermediate, resistant according to EUCAST clinical breakpoints) to various antibiotics. Outpatients consist of patients presenting at the hospital for dialysis, other day hospital care, or emergency. A more detailed description of the raw resistance data is made available by the ECDC [44].

#### Antibiotic consumption raw data.

Data on antibiotic consumption is publicly available from the ECDC, and consists of defined daily dose (DDD) per 1000 inhabitants by antibiotic class, country, year, sector (community, hospital care, total care) and uptake route (oral, parenteral, rectal, inhalation, implant, all). The dataset includes the same 30 countries as the resistance dataset, spans the years 1997-2016, and consists of the following classes of antibiotics, which are the main classes represented in the resistances tested: J01A (tetracyclines), J01B (amphenicols), J01C (penicillins), J01D (other betalactams), J01E (sulfonamides), J01F (macrolides), J01G (aminoglycosides), J01M (quinolones), J01R (combinations of antibacterials), J01X (others), P01A (agents against amoebiasis and other protozoal diseases), A07A (intestinal anti-infectives). A detailed description of the consumption data is made available by the ECDC [45].

### Preprocessing

#### Processing of antibiotic resistance data.

We computed the frequency of resistant for each bug, drug, country, year, and patient type combination. Intermediate isolates were considered resistant. We compared the temporal trends in resistance in inpatients and outpatients for drug-pathogen-country combinations that had data on both types of patients. The temporal trends in outpatients and inpatients were very correlated, with similar or higher levels of resistance in inpatients. Therefore we only considered resistance measured in inpatients for further analyses. We also restricted downstream analysis to combinations with a time series of at least 5 years, where all years had at least 30 isolates tested for drug resistance, and in total at least 10 resistant bacterial isolates.

#### List of the bug-drug combinations investigated.

*S. aureus*: CIP CLO DAP DIC FLC FOX LNZ LVX MET NOR OFX OXA RIF TEC

*S. pneumoniae*: AZM CIP CLR CRO CTX ERY LVX MFX NOR OFX OXA PEN

*E. faecalis*: AMP AMX GEH LNZ TEC VAN

*E. faecium*: AMP AMX GEH LNZ TEC VAN

*E. coli*: AMC AMK AMP AMX CAZ CIP COL CRO CTX DOR ETP FEP GEN IPM LVX MEM MFX NAL NET NOR OFX PIP POL TGC TOB TZP

*K. pneumoniae*: AMC AMK CAZ CIP COL CRO CTX DOR ETP FEP GEN IPM LVX MEM MFX NAL NET NOR OFX PIP POL TGC TOB TZP

*P. aeruginosa*: AMK CAZ CIP COL DOR ETP FEP GEN IPM LVX MEM NET PIP POL TOB TZP

*Acinetobacter spp.*: AMC AMK AMP AMX CAZ CIP COL CTX DOR FOX GEH GEN IPM LVX MEM NAL NET POL TOB TZP VAN

The abbreviations for antibiotic names are as follows: AMC: Amoxicillin and clavulanic acid; AMK: Amikacin; AMP: Ampicillin; AMX: Amoxicillin; AZM: Azithromycin; CAZ: Ceftazidime; CIP:Ciprofloxacin; CLO: Cloxacillin; CLR: Clarithromycin; COL: Colistin; CRO: Ceftriaxone; CTX: Cefotaxime; DAP: Daptomycin; DIC: Dicloxacillin; DOR: Doripenem; ERY: Erythromycin; ETP: Ertapenem; FEP: Cefepime; FLC: Flucloxacillin; FOX: Cefoxitin; GEH: Gentamicin High; GEN: Gentamicin; IPM: Imipenem; LNZ: Linezolid; LVX: Levofloxacin; MEM: Meropenem; MET: Meticillin; MFX: Moxifloxacin; NAL: Nalidixic acid; NET: Netilmicin; NOR: Norfloxacin; OFX: Ofloxacin; OXA: Oxacillin; PEN: Penicillin; PIP: Piperacillin; POL: Polymyxin B; RIF: Rifampicin; TEC: Teicoplanin; TGC: Tigecycline; TOB: Tobramycin; TZP: Piperacillin + Tazobactam; VAN: Vancomycin.

#### Processing of antibiotic use data.

For many countries and years, the total consumption of antibiotics by all routes of administration was reported. We primarily used this information when it was available. Moreover, some countries and years recorded the consumption decomposed by route of administration of antibiotics. This was most commonly oral and parenteral, and, less frequently, inhalation, rectal, implant and ‘other’ routes. When total consumption was not available, we summed the consumption recorded over all routes to obtain an estimate of total consumption. We considered consumption both in the hospital sector and in the community. To remove all outliers for a given antibiotic class, country, sector, route of administration for each combination we removed datapoints for years not within  ± 3 × *IQR* (IQR = Inter quartile range) of the temporal median. The cleaning of antibiotic consumption data is visualised for the community antibiotic consumption in S2 Appendix, with outliers highlighted in red. Furthermore, Fig H in S1 Text shows a histogram of how many outliers are found per temporal trajectory of consumption.

### Inferring temporal trends in antibiotic resistance

For combinations of bug, drug, country, we fitted logistic models to the temporal trends in the frequency of resistance. Fitting was done with non-linear least square regression, with the function nlsLM from the minpack.lm R package, except for the flat line which was fit using lm from the base stats package [46,47]. The error is assumed to be normally distributed with variance proportional to the inverse of the sample size for each year. For a small number of trajectories with very low resistance frequency, the fitting failed to converge. These were excluded from further analysis.

We fitted three models to the temporal trends data:

1. A standard logistic model with two parameters, an offset and a slope. The standard logistic model results in an increasing sigmoid from 0 to 1, or a decreasing sigmoid from 1 to 0 if the slope is negative. This corresponds to the standard population genetics model describing the action of selection on resistance frequency. The resistance frequency *f* for year *t* under this model is given by:
  $$\begin{array}{ccc} f(t)=\frac{1}{1+e^{-k_{1}(t-k_{0})}} & & \end{array}$$ (1)
  where $k_{1}$ is the slope parameter and $k_{0}$ determines the offset.
2. A ‘flat’ model with slope 0 and an intercept. In this model, the resistance frequency *f* for year *t* is given by:
  $$\begin{array}{ccc} f(t)=k_{2} & & \end{array}$$ (2)
3. A plateauing logistic model with a maximum frequency below 1, where the sigmoid function is scaled by parameter $k_{2}$. In this model, the resistance frequency *f* for year *t* is given by:
  $$\begin{array}{ccc} f(t)=\frac{k_{2}}{1+e^{-k_{1}(t-k_{0})}} & & \end{array}$$ (3)

We compared the models using the corrected Akaike Information Criterion (AICc), with the best model corresponding to the smallest AICc [48]. The AICc is given by:


$$\begin{array}{ccc} 2k-2\ln(\hat{L})+\frac{2k^{2}+2k}{n-k-1} & & \end{array}$$ (4)

where $\hat{L}$ is the maximum likelihood estimate, *k* the number of parameters and *n* the sample size (the number of years for which we have data for the bug-drug-country combination).

We quantified the goodness of fit with the mean absolute error of the best fit model. For the two logistic models, we determined the statistical significance of the slope based on a threshold of p<0.05.

This resulted in six categories of temporal trends:

1. significantly increasing, when the best model was the standard logistic and the slope was significantly positive.
2. non-significantly increasing, when the best model was the standard logistic and the slope was positive but not significantly different from 0
3. non-significantly decreasing, when the best model was logistic and the slope was negative but not significantly different from 0.
4. significantly decreasing, when the best model was logistic and the slope was significantly negative.
5. stable, when the best model was a flat fit.
6. stabilising, when the best model was the logistic with plateau different from 1.

Additionally, some trajectories were classified as ‘poor fit’ when the mean absolute error exceeded 0.05 and were not analysed further. The ‘stabilising’ category can be subdivided further in ‘stabilising significantly increasing’ and ‘stabilising non-significantly increasing’ depending on the significance of the slope leading to the plateau.

### Comparison of rate of resistance change across models

The slope parameters in the standard and scaled logistic models ($k_{1}$ in Eqs 1 and 3) are not directly comparable. This is because the parameter reflects how quickly resistance will reach its maximum level. As the maximum is different in the two models, the same slope parameter in the two models translates to a different rate of change in the actual resistance frequencies. For the analyses in which we sought to compare the speed of change across the two models—i.e. the comparison of increasing and stabilising trajectories and the correlation with antibiotic usage—we computed a comparable measure of the change in resistance frequencies. Specifically, we looked at the maximal rate of change predicted by the best fitting model in the temporal window for which we had data.

### Correlation between antimicrobial consumption and resistance

We correlated antimicrobial consumption and resistance across countries and time. In all cases, we correlated the level of resistance to a particular antibiotic to the total consumption of all antibiotics of this class.

#### Correlation between antimicrobial consumption and resistance across countries.

For each bug-drug combination, we correlated across countries the median antibiotic consumption across years, to three properties of the resistance temporal trends. These three properties were the median resistance across years, the resistance plateau (for ‘stable’ and ‘stabilising’ categories), the slope of the logistic (for ‘increasing (s)’ and ’stabilising’ categories). As described above, the slope was computed as the maximal rate of change reached in the temporal window for which we had data. This ensures the slope is comparable across the logistic model with and without intermediate plateau, unlike the slope parameter. We quantified the correlation with Spearman’s rank correlation coefficient across countries, for bug-drug combinations represented by at least five countries. The correlation was calculated using SpearmanRho from DescTools version 0.99.49 [49].

Formally, the resistance data consist in frequencies of resistance in bug *b* to drug *d*, for country *c*, year *y*, denoted $p_{b,d,c,y}$. Properties of the temporal trajectory, such as the plateau level of resistance *P*, do not depend on year, and are denoted for example $P_{b,d,c}$. The consumption data consist in the use of a particular drug *d* in country *c*, year *y*, denoted $u_{d,c,y}$. The median consumption across years was used to represent the typical antibiotic use in each country over the time period and is denoted $\text{med}{\left(u\right)}_{d,c}$. The correlation between use and resistance plateau is:


$$\begin{array}{ccc} \rho_{c}\left(P_{b,d,c},\text{med}{\left(u\right)}_{d,c}\right)=\Sigma_{b,d} & & \end{array}$$ (5)

Where the function $\rho_{c}$ denotes the Spearman’s rank correlation coefficient operated on the two vectors $P_{b,d,c}$ and $\text{med}{\left(u\right)}_{d,c}$ indexed by countries. The resulting quantity, $\Sigma_{b,d}$, is the correlation across countries for bug-drug combination *b* , *d*.

This analysis resulted in a number of correlation coefficients (the $\Sigma_{b,d}$) for different bug-drug combinations. These coefficients are calculated for combinations of antibiotics prescribed in the community vs. the hospital sector, (we remind that we consider only resistance in inpatients), and of the three properties of temporal trends which are correlated (total of 6 combinations).

#### Correlation between antimicrobial consumption and resistance across time.

For each bug-drug-country combination, we correlated across years the level of resistance and level of antibiotic consumption. This results in a Spearman’s rank correlation coefficient for the bug-drug-country combinations and community vs. hospital sector use. This was again calculated using SpearmanRho from DescTools version 0.99.49 [49]. We kept only bug-drug-country combinations with at least 5 years of data available. We averaged these correlations over countries for each bug-drug combination, retaining only bug-drug combinations for which 5 countries of more were represented. It is expected that the correlation across years between the frequency of resistance and the corresponding antibiotic use is positive if the bacterial population adapts to the changing use of specific antibiotic.

Formally, we use here the frequency of resistance in bug *b* to drug *d*, for country *c*, year *y*, denoted $p_{b,d,c,y}$, and the consumption of drug *d* in country *c*, year *y*, denoted $u_{d,c,y}$. The correlation across years between use and resistance is:


$$\begin{array}{ccc} \rho_{y}\left(P_{b,d,c,y},u_{d,c,y}\right)=\Theta_{b,d,c} & & \end{array}$$ (6)

Where the function $\rho_{y}$ denotes the Spearman’s rank correlation coefficient operated on the two vectors $P_{b,d,c,y}$ and $u_{d,c,y}$ indexed by years. The resulting quantity, $\Theta_{b,d,c}$, is the correlation across years for a bug-drug-country combination. Finally we averaged $\Theta_{b,d,c}$ over countries to obtain ${\bar{\Theta }}_{b,d}$, the correlation across years for this bug-drug combination *b* , *d*.

We compared the correlation across years vs countries for different bug-drug combinations. Indeed, if the bacterial population evolves in response to changing antibiotic use, it is expected that levels of resistance match both the contemporaneous and local antibiotic use [28]. We therefore tested whether bug-drug combinations with stronger correlation across countries also exhibited stronger correlation across years. To this end, we looked at the association between the two correlation coefficients across bug-drug combinations (Fig M in S1 Text).

## Supporting information

S1 Text Combined file of supporting information (text and figures).
(PDF)

S1 Appendix **Antibiotic resistance trajectories for all bug-drug-country combinations.** The plots show resistance frequency against calendar year for all bug-drug-country combinations analysed in the paper. The error bars represent 95% confidence intervals. The transparent line shows a smoothing function – not any of the models fitted in the paper. The text within each plot indicates how the trajectory was categorised.
(PDF)

S2 Appendix **Antibiotic consumption trajectories for all drug-country combinations.** The plots show antibiotic consumption, as measured in defined daily doses (DDDs), against calendar year for all drug-country combinations. The dark green line shows mean consumption, the light green line median consumption (before removal of outliers). Red points indicate outliers, defined as not within 3x the inter-quartile range. These were removed for the analyses in the paper.
(PDF)

S2 Appendix Difference between the AICc value of the best and second best fitting model for each temporal trajectory.
(CSV)
