# Supplementary material for: The evolution of antibiotic resistance in Europe, 1998–2019
Source: PLoS Pathog. 2025 Apr 3;21(4):e1012945. doi: 10.1371/journal.ppat.1012945 (PMC11967945; doi:10.1371/journal.ppat.1012945)
Supplement: S1 Text — Combined file of supporting information (text and figures). (PDF) [file ppat.1012945.s001.pdf]

Supporting Information for Temporal Trends in Antibiotic Resistance in Europe, 1998-2019

February 11, 2025

1 Categorisation of temporal trends

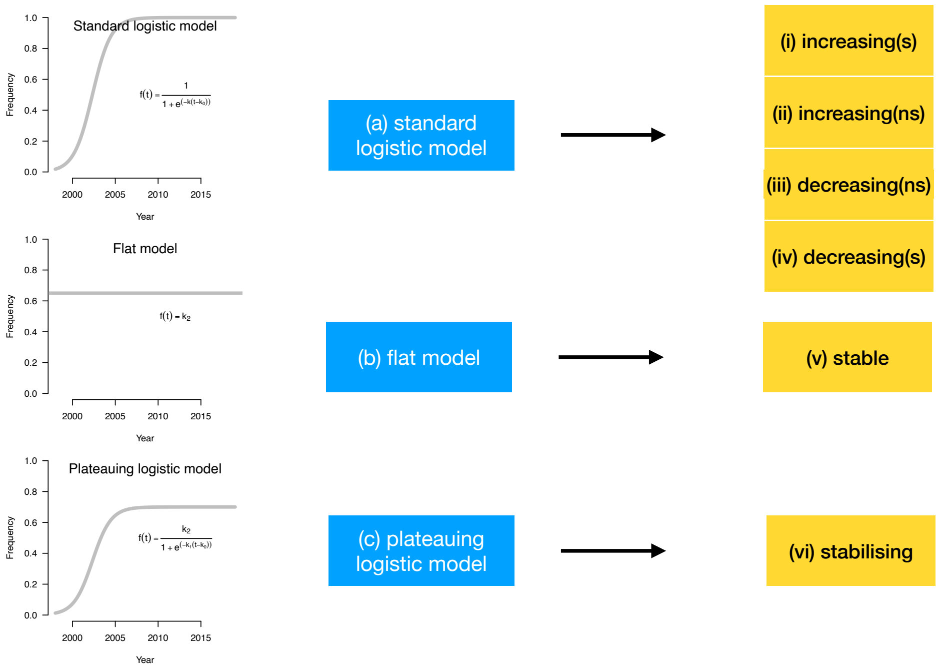

Figure A: **Model fitting and categorisation of temporal trends**. This diagram shows schematically how the process of model fitting works. The three different models a-c are represented by the blue boxes. The categories that are created from the models a-c are represented by the yellow boxes i-vi.

2 Speed of increase or decrease in European resistance frequencies

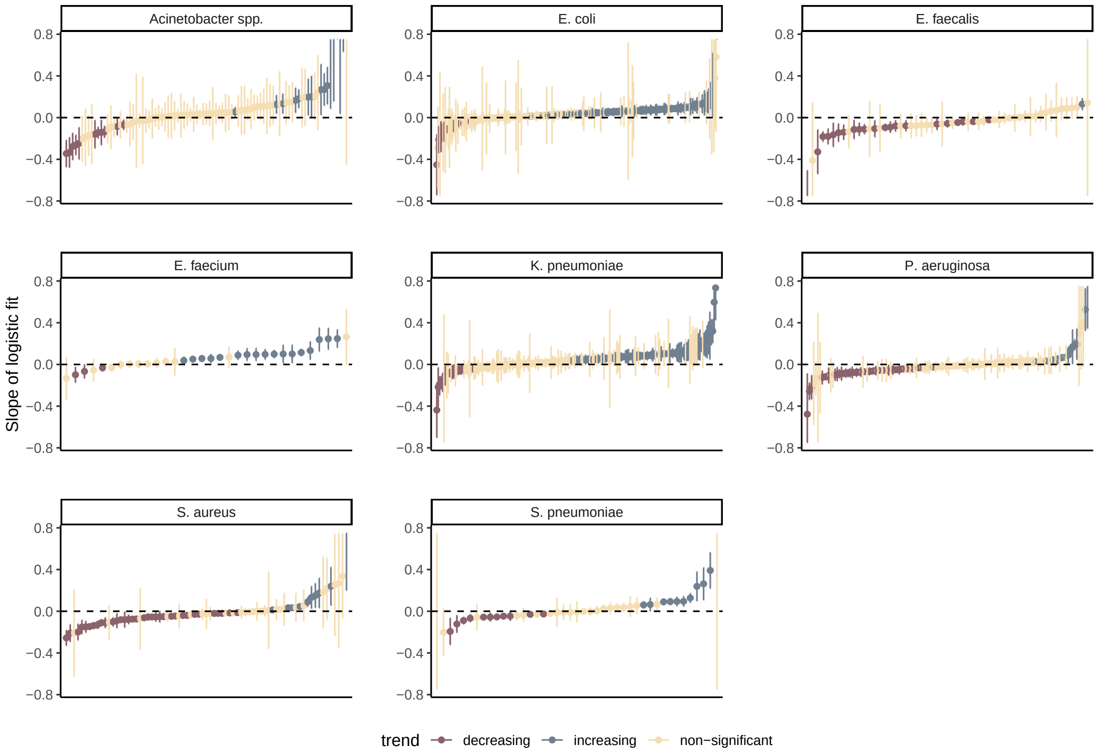

Figure B: The speed of increase or decrease in European resistance frequencies, as measured by the slope parameter in a standard logistic model, for all bug-drug-country combinations. The x-axis is the index of the bug-drug-country combination, ordered by the magnitude of the slope parameter. For clarity, 27 combinations with very high or low slopes (outside of the interval [-0.75;0.75]) have been excluded (see Supporting Information). The plot includes both in- and outpatients. The depicted 95% confidence intervals are based on the t-distribution.

3 Predictors of temporal trends

To reduce the complexity of the analysis and make it easier to interpret, we first combined our categories into 'rising' (increasing (s) and (ns)), 'declining' (decreasing (s) and (ns)) and 'equilibrium' (stable and stabilising) trends. We then fitted a separate binomial regression for each combined category (i.e. 'rising' vs not as the outcome variable, 'declining' vs not declining, equilibrium vs not equilibrium) using `glm` from `R stats version 3.6.2`, with species, country and antibiotic class as predictor variables (SI Figure C). For each regression, we used *Acinetobacter spp.* as the reference species, Austria as the reference country and J01A as the reference antibiotic class.

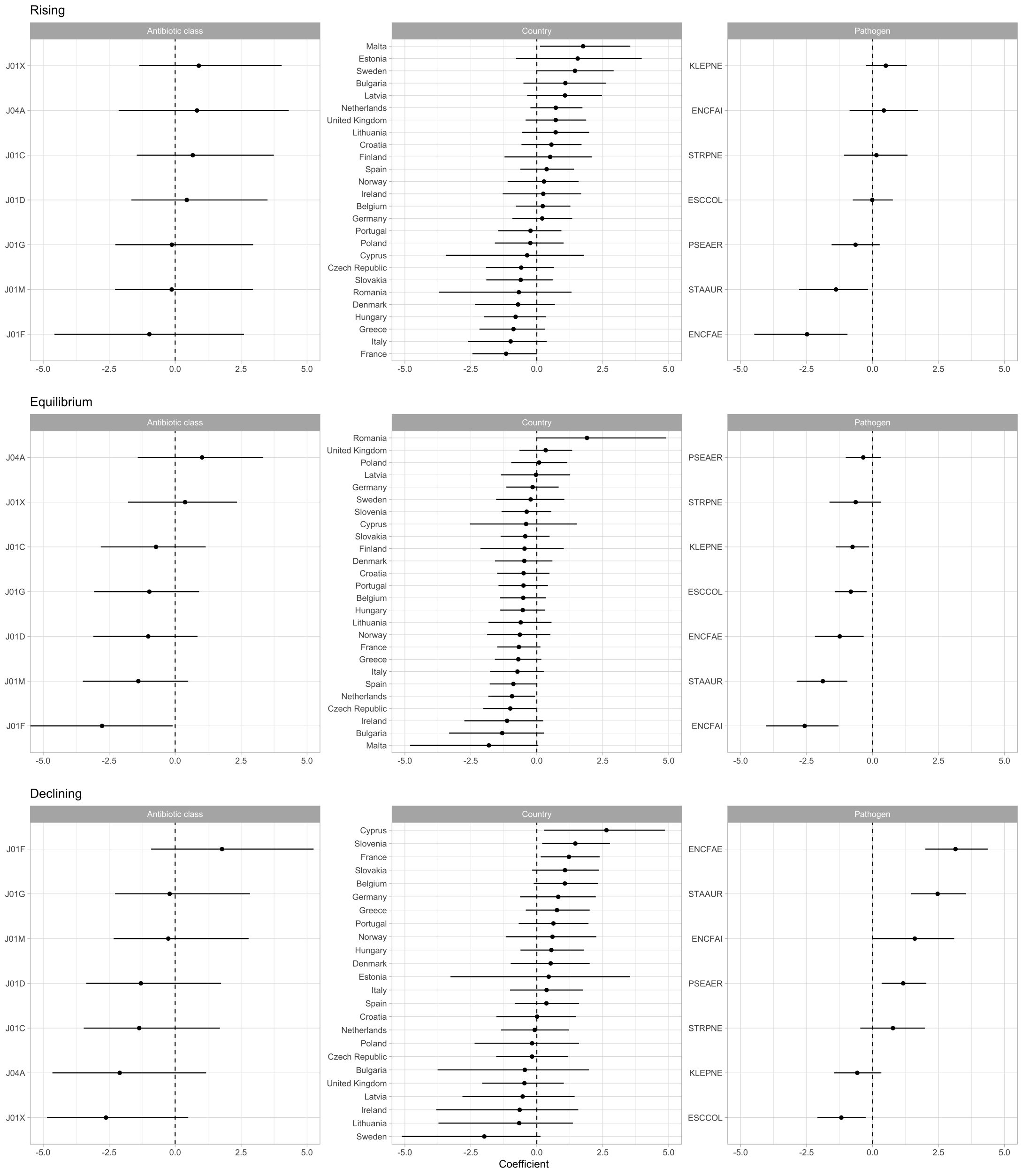

Figure C: Predictors of each type of temporal trend: 'rising' (increasing (s) and (ns)), 'declining' (decreasing (s) and (ns)) and 'equilibrium' (stable and stabilising). The x-axis represents the coefficient of a binomial regression with species, country and antibiotic class as predictors of temporal trend (e.g. rising vs others). The reference species (for which the effect size is 0) is *Acinetobacter spp.*. The reference country is Austria. The reference antibiotic class is J01A. Error bars show 95% confidence intervals. In a small number of cases, countries had very little variability in temporal trends (e.g. no rising trajectories detected in Slovenia and Luxembourg); this results in an extreme value for the country's coefficient and very high uncertainty. We have therefore omitted these cases from the plots.

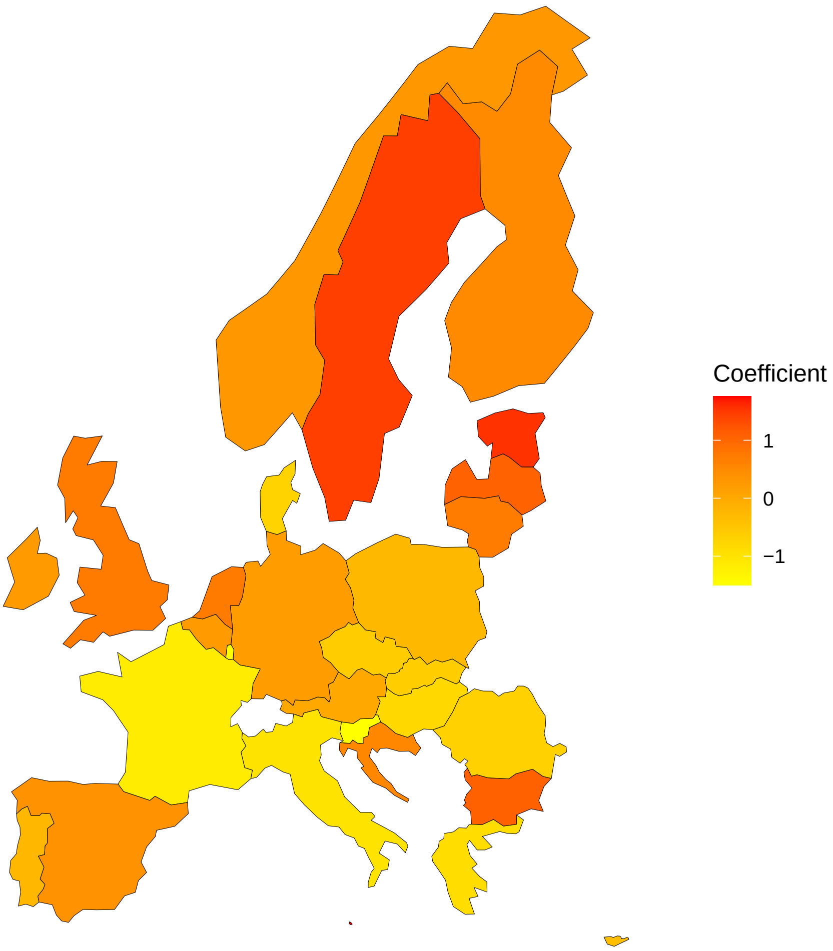

Figure D: Predictors of rising vs. not rising as depicted on a map of Europe. A map of Europe coloured by the coefficient of the binomial regression. The reference country is Austria (coefficient equal to 0). Slovenia and Luxembourg do not present any increasing trend, and were associated with an extremely negative coefficient of -16 in the binomial regression; for visualisation purpose, we set the coefficient of these countries to -1.5. Map contours are from the R package 'rworldmap' which uses country borders derived from public domain Natural Earth data v1.4.0 available at https://www.naturalearthdata.com/.

4 Comparison of rate of change of resistance frequencies

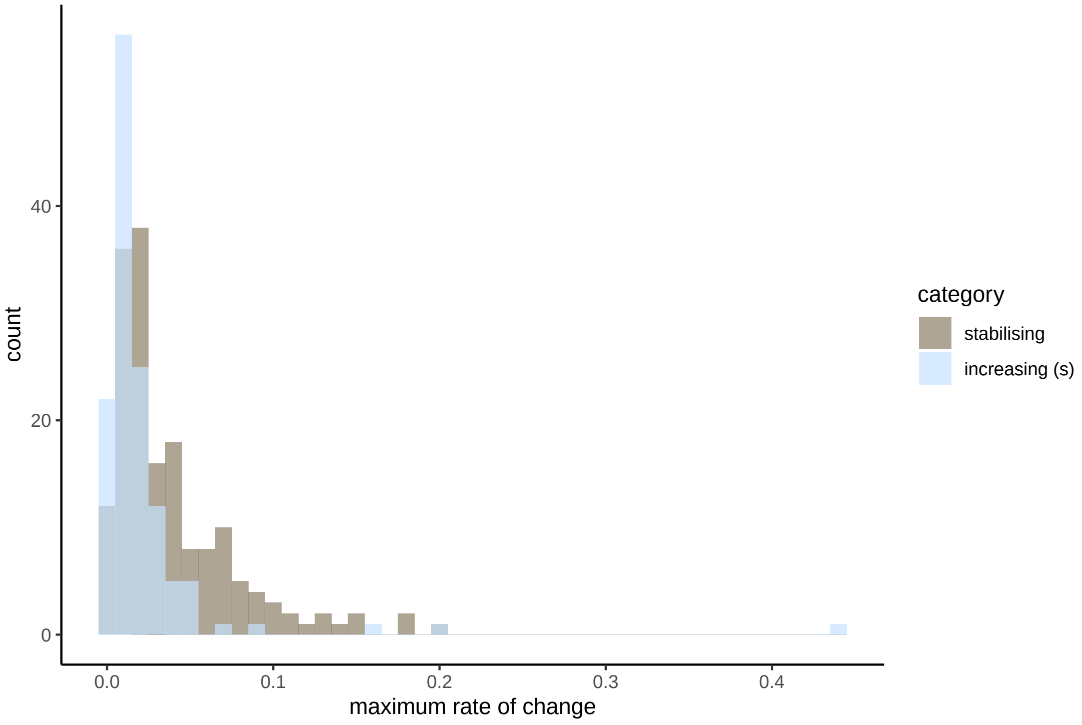

Figure E: Comparison of the speed of change of resistance frequencies in the significantly increasing and stabilising categories. The speed of change is quantified as the maximum rate of change in the time window we have data for. The lower rate of change in the increasing category suggest that the increase may reflect a changing equilibrium rather than non-equilibrium dynamics.

5 Uncertainty in model selection

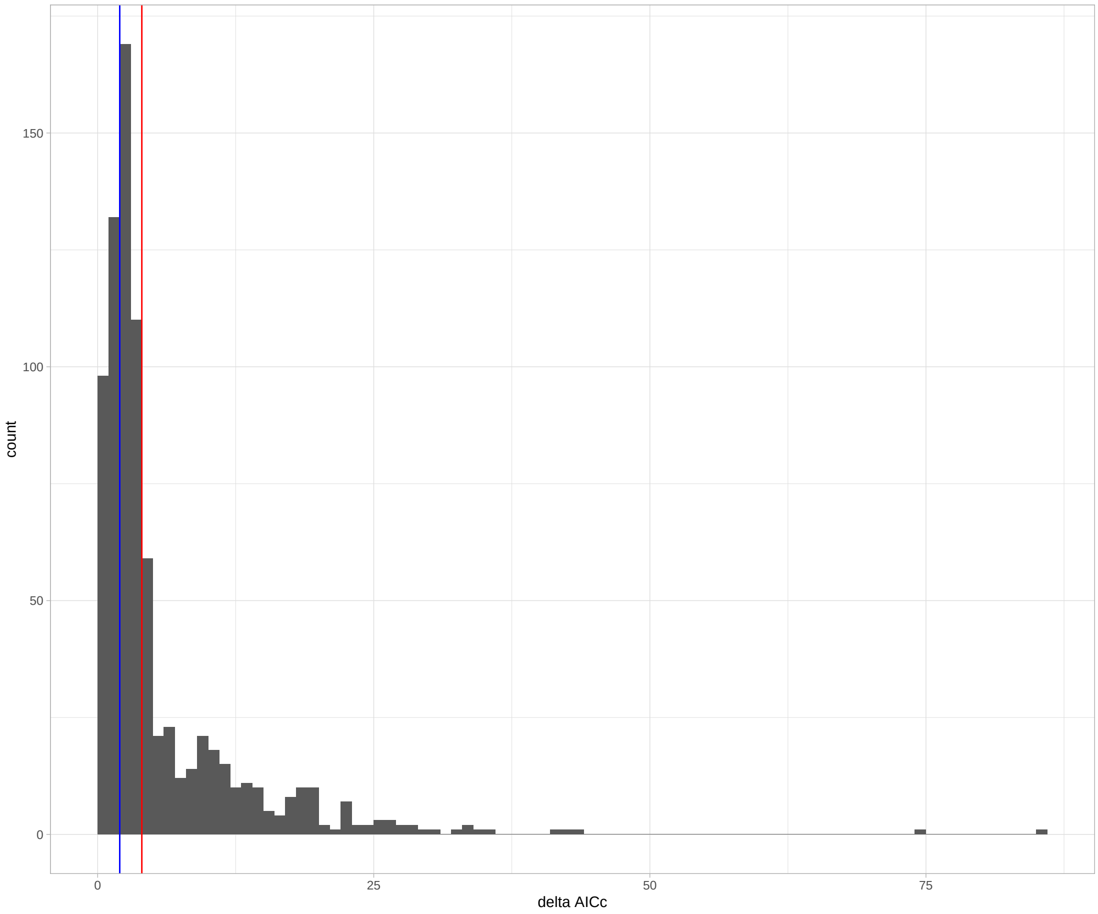

Figure F: The distribution of the difference in AICc between the second smallest and the minimal AICc value, reflecting how strongly the best fit model was preferred over the second best. The blue and red vertical lines mark the thresholds ΔAICc $= 2$ and ΔAICc $= 4$. These thresholds are often chosen to suggest moderate and strong support for the best model. More information about the trajectories for which ΔAICc < 2 is given in Appendix C.

6 Uncertainty in plateau and slope estimates

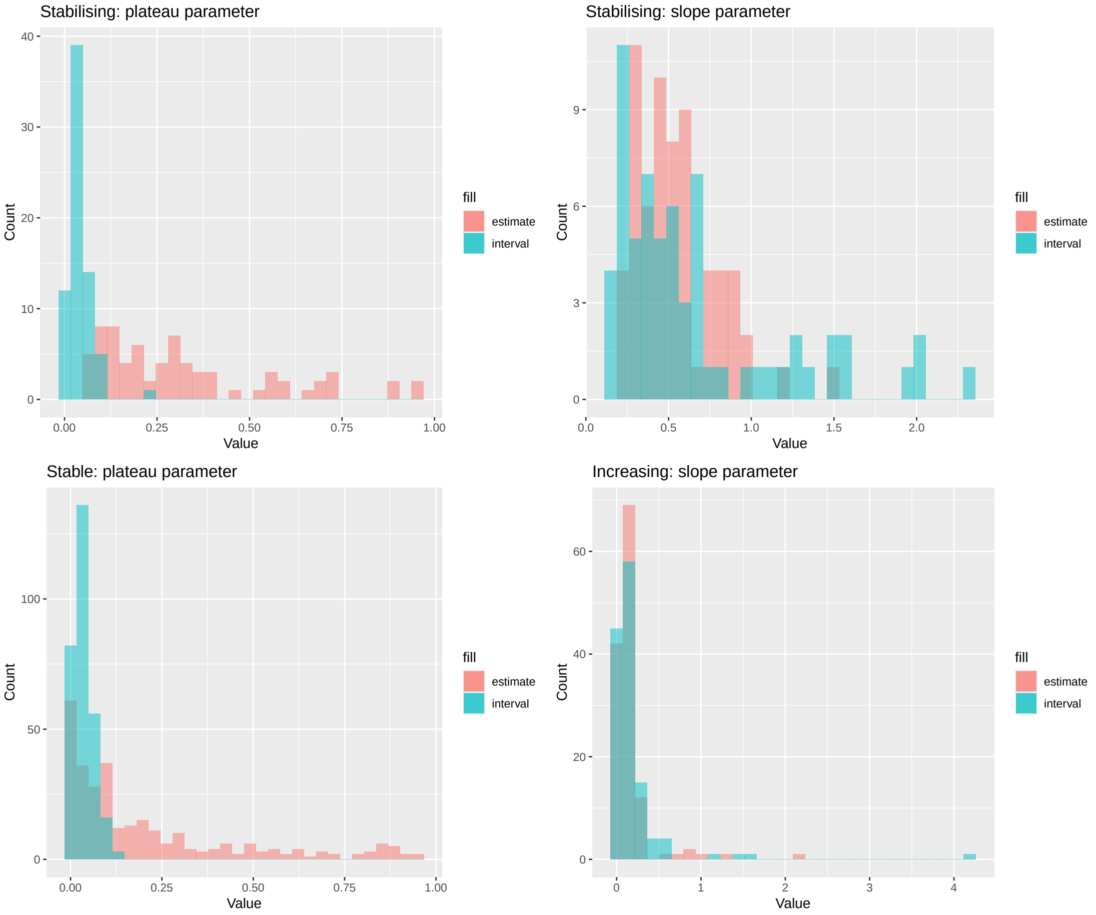

Figure G: Uncertainty associated with estimates of the plateau vs slope parameter for stabilising (plateau and slope), stable (plateau) and increasing (slope) trajectories. For each parameter, we show the distribution of the estimates themselves and the distribution of the magnitude of the 95% confidence intervals associated with these estimates. When the distributions overlap, the uncertainty in the estimates is large comparative to the variability of the estimates across countries. This would make it more difficult to detect a correlation between the parameter value and antibiotic consumption across countries, potentially contributing to the weaker signal we find for the slope parameter.

7 Link between resistance and antibiotic consumption

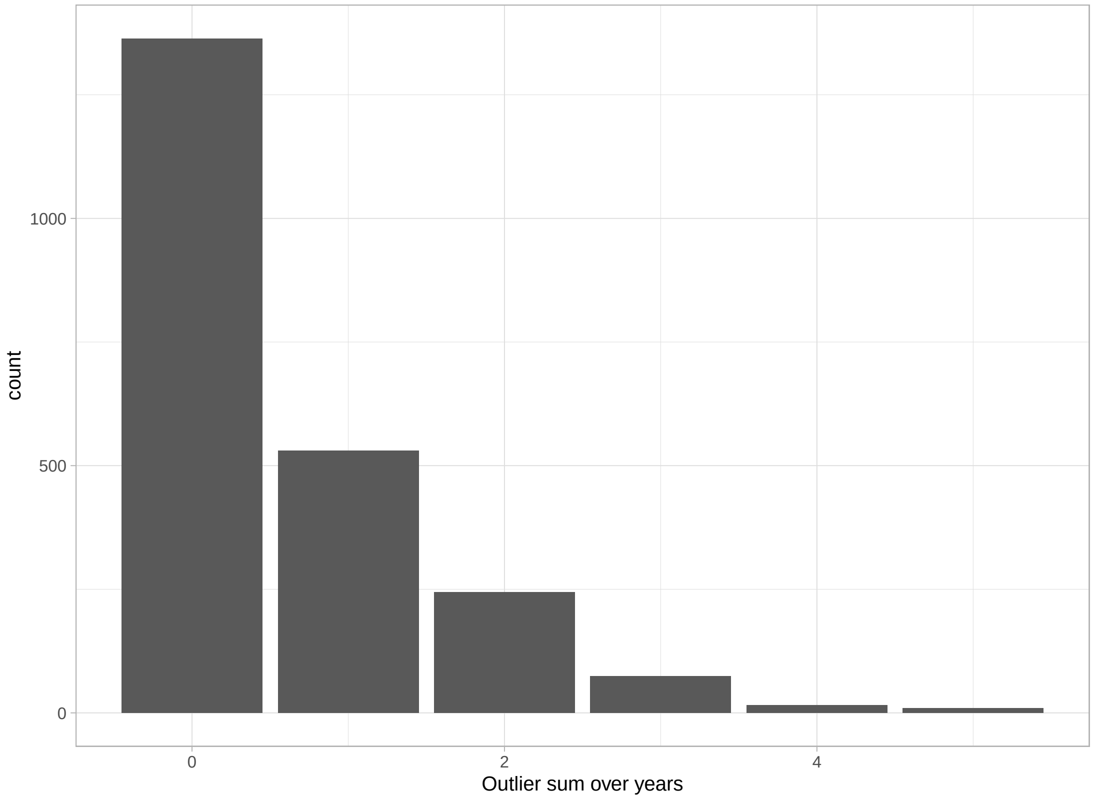

Figure H: Number of outliers in antibiotic consumption trajectories eliminated at the data cleaning step. For a given antibiotic class, country, sector, route of administration; for each combination, we removed datapoints for years not within $\pm 3 \times IQR$ (IQR = Inter quartile range) of the temporal median. The vast majority of consumption trajectories present 0 or 1 outlier only (i.e., 0 or 1 year of consumption data removed at the data cleaning step).

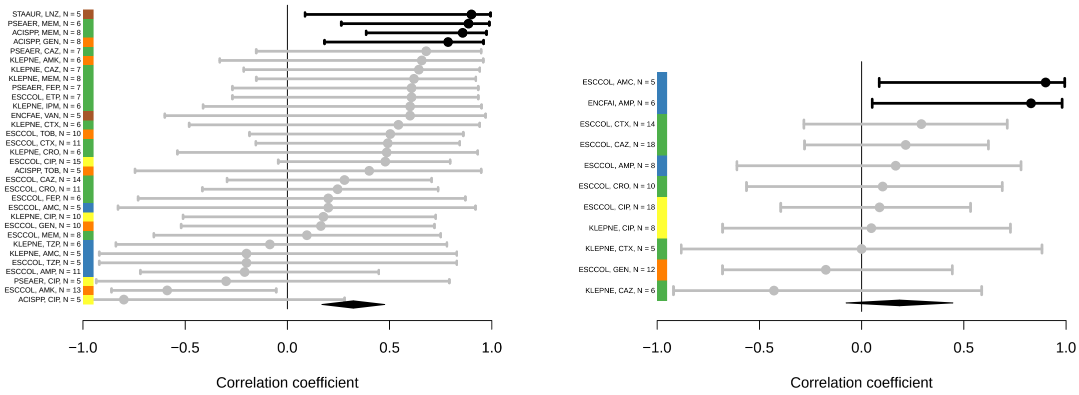

Figure I: Spatial correlation coefficients between the **plateau** frequency of antibiotic resistance (left), or the **slope** (right), and the rate of use of the corresponding antibiotic in **hospitals**, for all bug-drug combinations. The number of countries included is indicated for each combination. The black diamond shows the overall mean and 95% confidence interval.

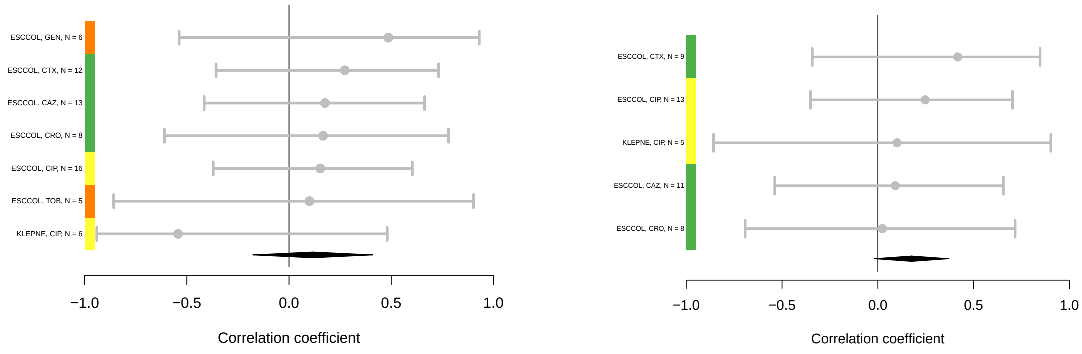

Figure J: Spatial correlation coefficients between the rate of increase of antibiotic resistance, and the rate of use of the corresponding antibiotic in the community (left) or in the hospital (right), for all bug-drug combinations, when **restricting to stabilising temporal trajectories**. The number of countries included is indicated for each combination. The black diamond shows the overall mean and 95% confidence interval.

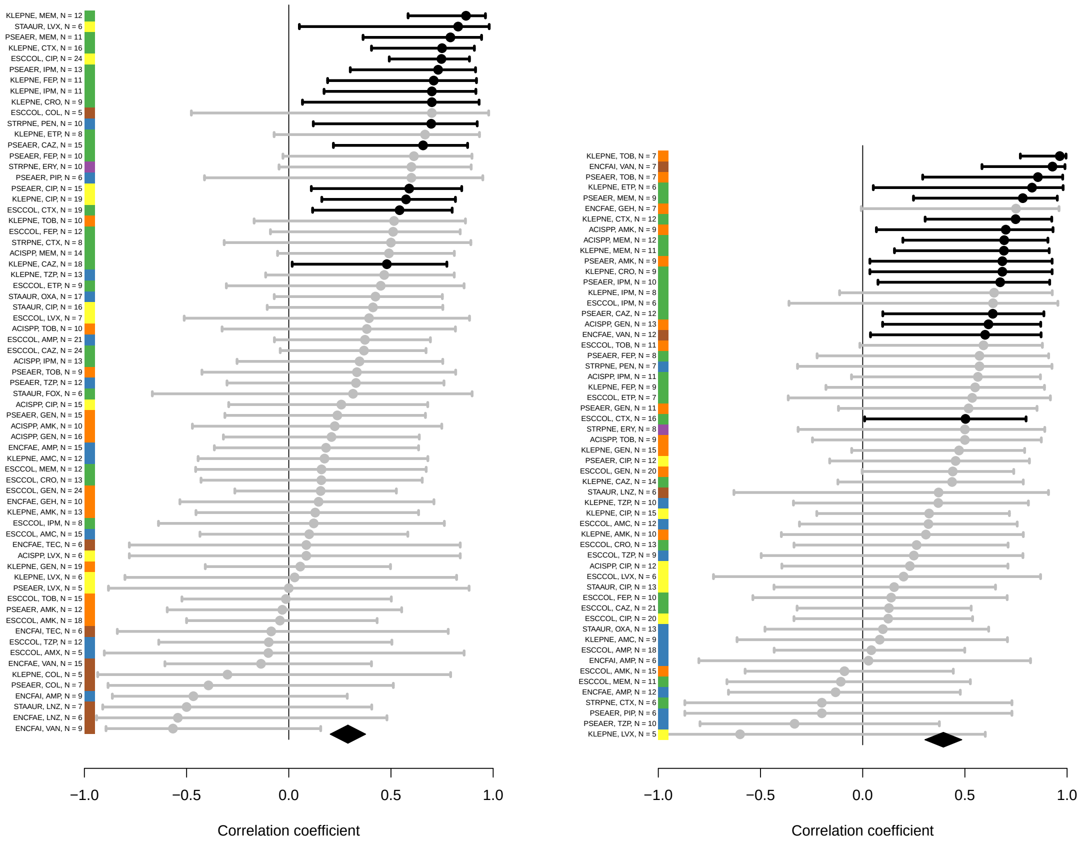

Figure K: Spatial correlation coefficients between the **median** frequency of antibiotic resistance and the rate of use of the corresponding antibiotic in the **community** (left), or in the **hospitals** (right), for all bug-drug combinations. The number of countries included is indicated for each combination. The black diamond shows the overall mean and 95% confidence interval.

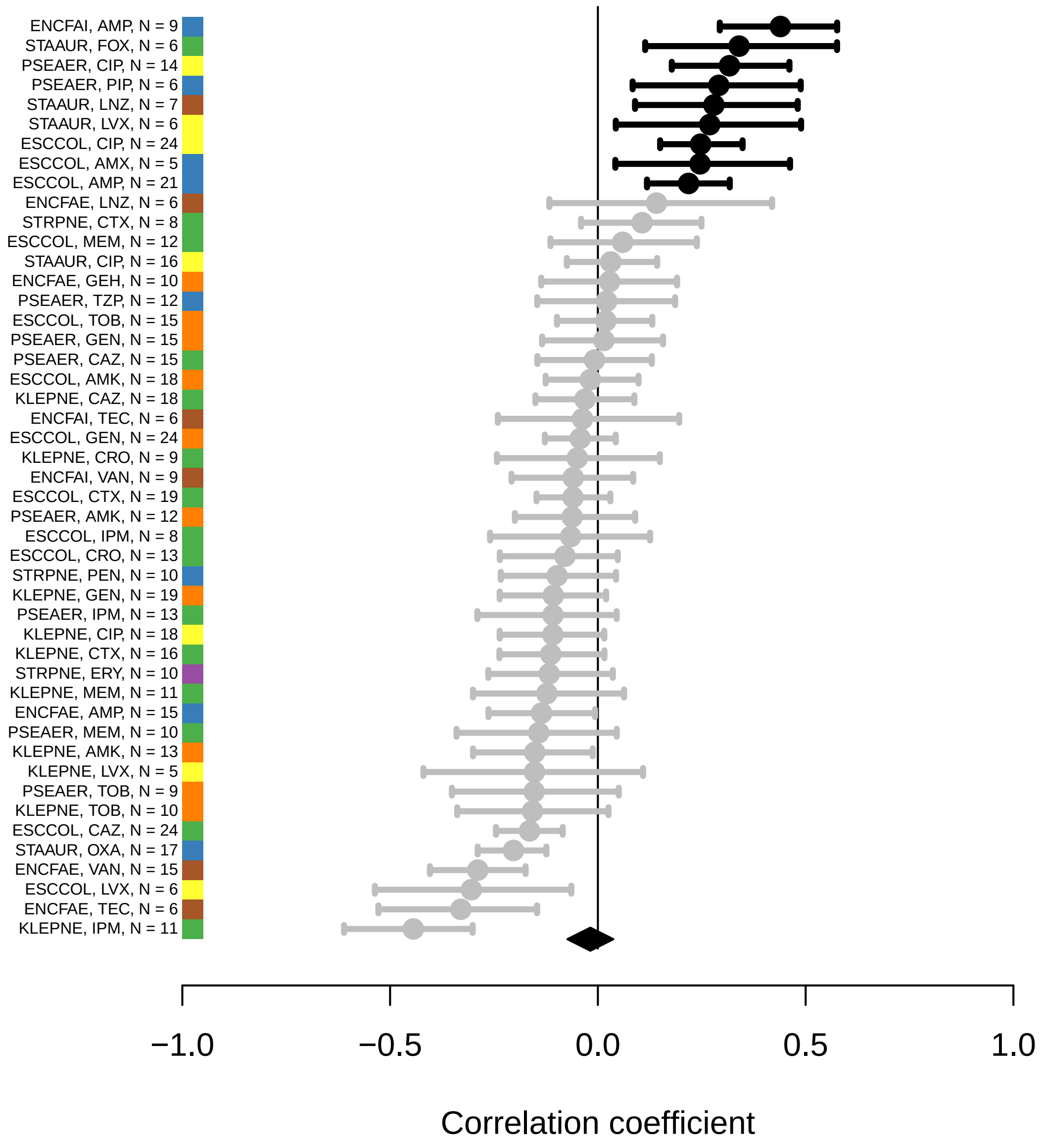

Figure L: Temporal correlation (Spearman's rho) between the frequency of resistance and **community** antibiotic use in the same year across bug-drug combinations. The number of countries included is indicated for each combination. The black diamond shows the overall mean and 95% confidence interval.

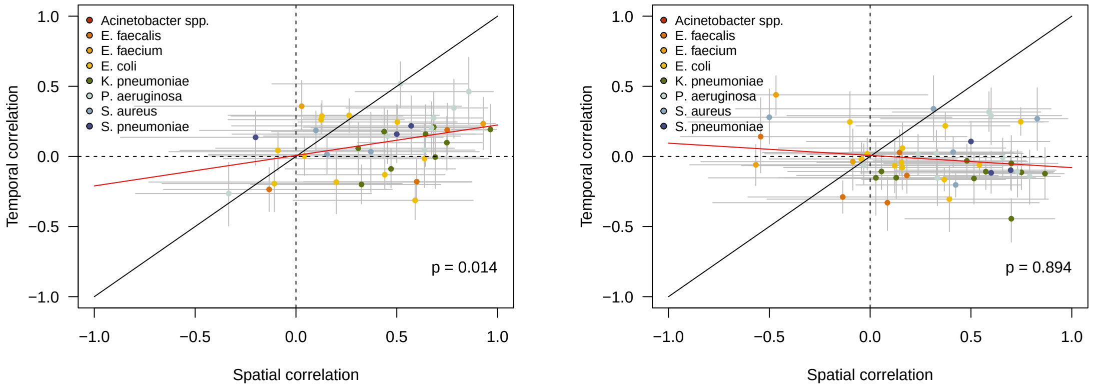

Figure M: **Temporal correlation of the frequency of resistance with hospital antibiotic use (left) or community antibiotic use (right) across bug-drug combinations, versus the corresponding spatial correlations**. A positive correlation between temporal and spatial correlations indicates that bug-drug combinations with more spatial adaptation also exhibit more temporal adaptation. The red line is a linear regression between these two quantities, with the p-value indicated. The positive correlation suggest antibiotic resistance follows temporal fluctuations in the use of antibiotics in the hospital sector (left panel), but not in the community (right panel).

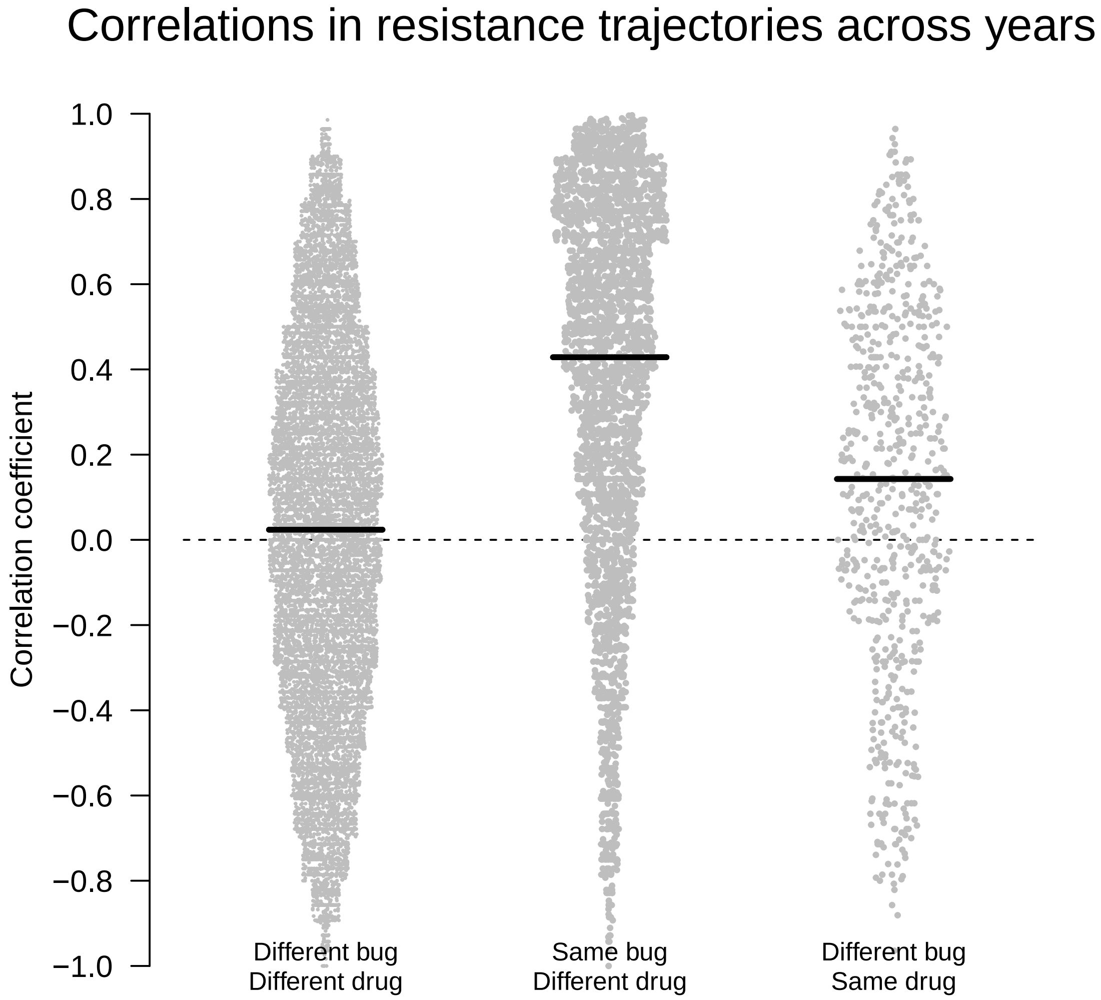

Figure N: **Correlations between all pairs of AMR trajectories.** For each country, we considered all pairs of bug-drug combinations. As for the analysis of resistance trajectories, we focused on combinations where we had at least five overlapping years, all years had at least 30 isolates tested for drug resistance, and in total at least 10 resistant bacterial isolates. For each pair of resistance trajectories, we correlated resistance across years on the overlapping years. We show these correlations for pairs of different bug, different drugs (left); for pairs of same bug, different drugs (middle); and for pairs of different bug, same drug (right); Each point is the correlation coefficient for a country and pair of bug-drug combination. The horizontal line shows the median. The measure of correlation was Spearman rank correlation coefficient.

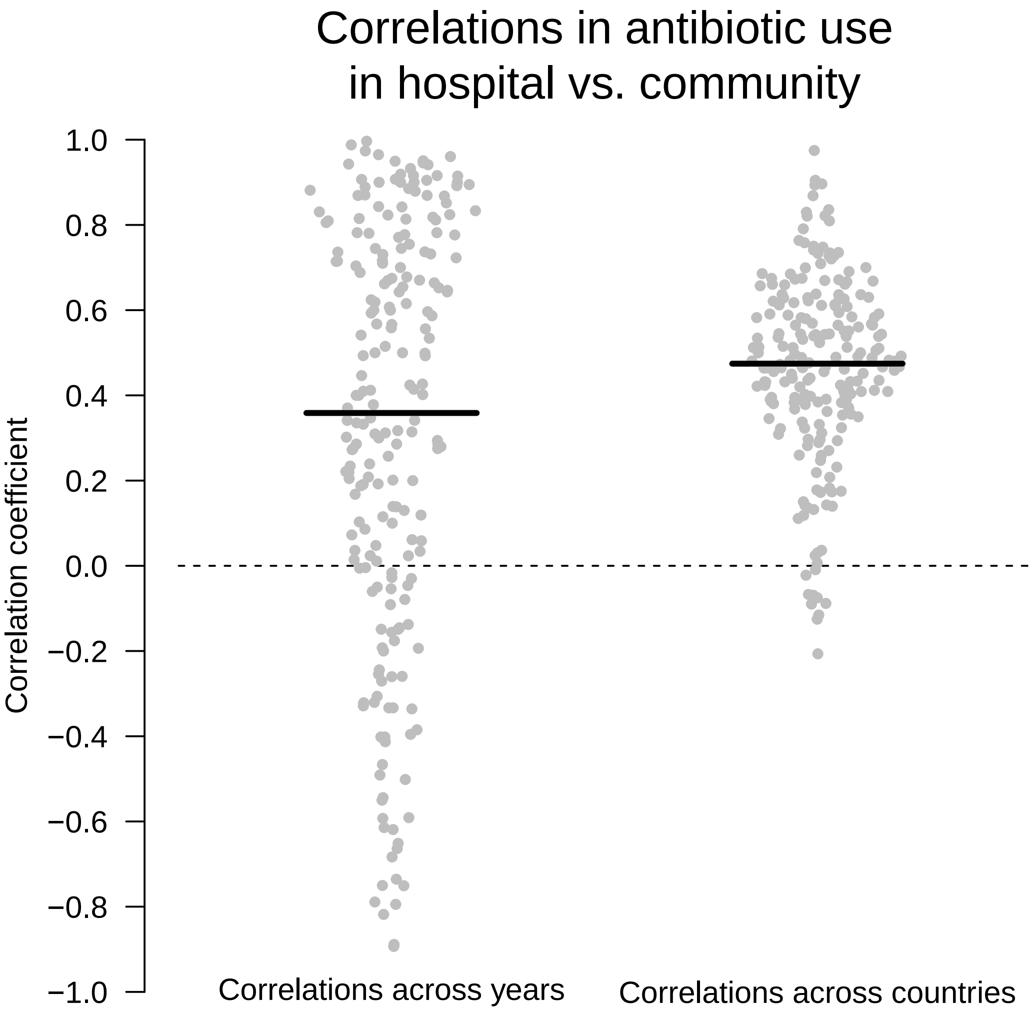

Figure O: **Correlations between hospital use and community use of antibiotics.** We show both correlations across years (left) and correlations across countries (right). The correlation across years is calculated as such: for each drug-country combination, we correlated the anbiotic use in the hospital sector with that of the community. The correlation across countries is calculated as such: for each drug-year combination, we correlated the anbiotic use in the hospital sector with that of the community. Each point is the correlation coefficient for a drug-country (left) or a drug-year (right) combination. The horizontal line shows the median. In all cases, the measure of correlation was Spearman rank correlation coefficient.
