# Supplementary material for: The evolution of antibiotic resistance in Europe, 1998–2019
Source: PLoS Pathog. 2025 Apr 3;21(4):e1012945. doi: 10.1371/journal.ppat.1012945 (PMC11967945; doi:10.1371/journal.ppat.1012945)

Appendix A for *Temporal Trends in Antibiotic Resistance in Europe, 1998-2019*

Antibiotic resistance trajectories for all bug-drug-country combinations

The plots show resistance frequency against calendar year for all bug-drug-country combinations analysed in the paper. The error bars represent 95% confidence intervals. The transparent line shows a smoothing function – not any of the models fitted in the paper. The text within each plot indicates how the trajectory was categorised.

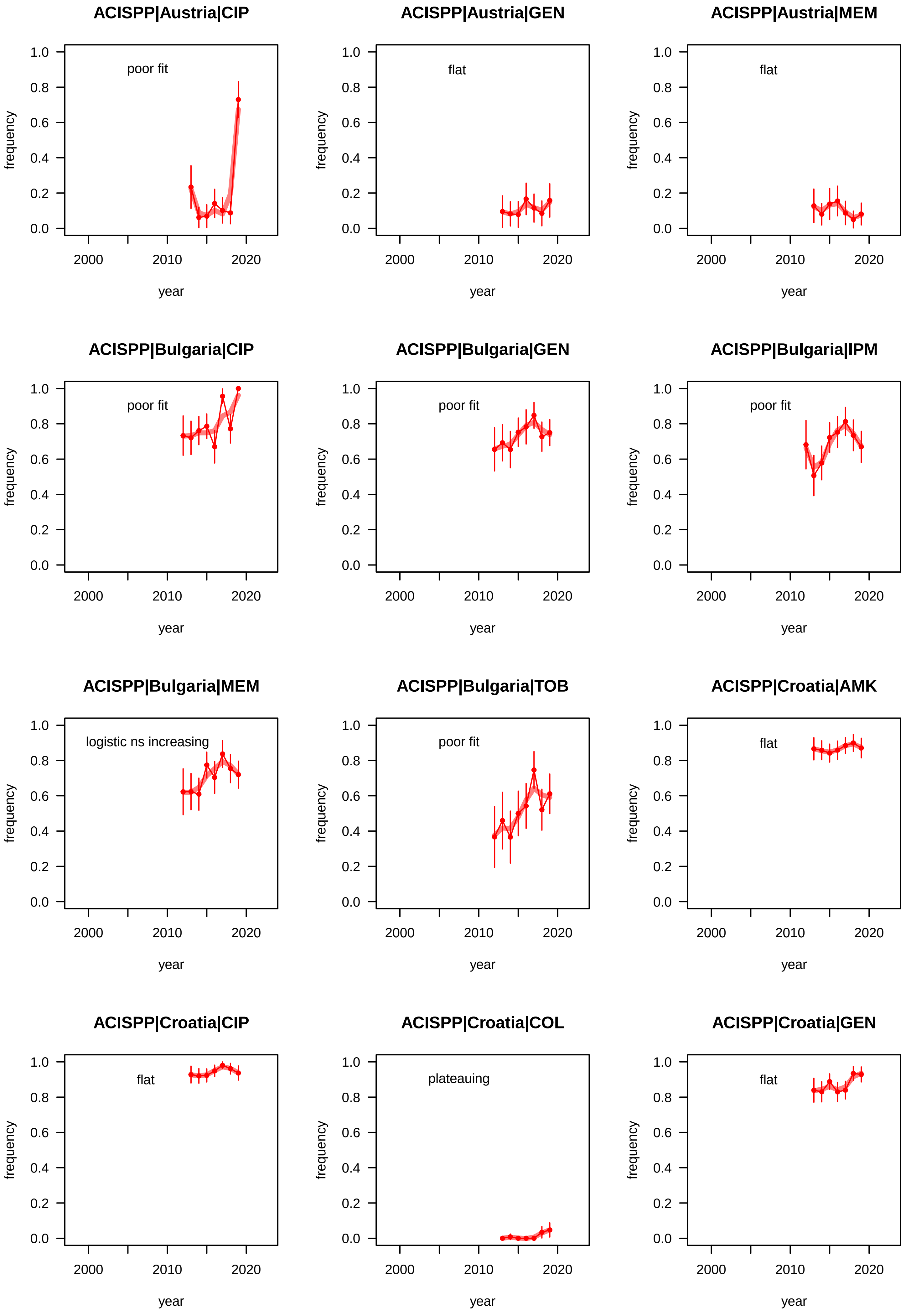
ACISPP|Austria|CIP
poor fit
ACISPP|Austria|GEN
flat
ACISPP|Austria|MEM
flat
ACISPP|Bulgaria|CIP
poor fit
ACISPP|Bulgaria|GEN
poor fit
ACISPP|Bulgaria|IPM
poor fit
ACISPP|Bulgaria|MEM
logistic ns increasing
ACISPP|Bulgaria|TOB
poor fit
ACISPP|Croatia|AMK
flat
ACISPP|Croatia|CIP
flat
ACISPP|Croatia|COL
plateauing
ACISPP|Croatia|GEN
flat
frequency
0.0
0.2
0.4
0.6
0.8
1.0
year
2000
2010
2020

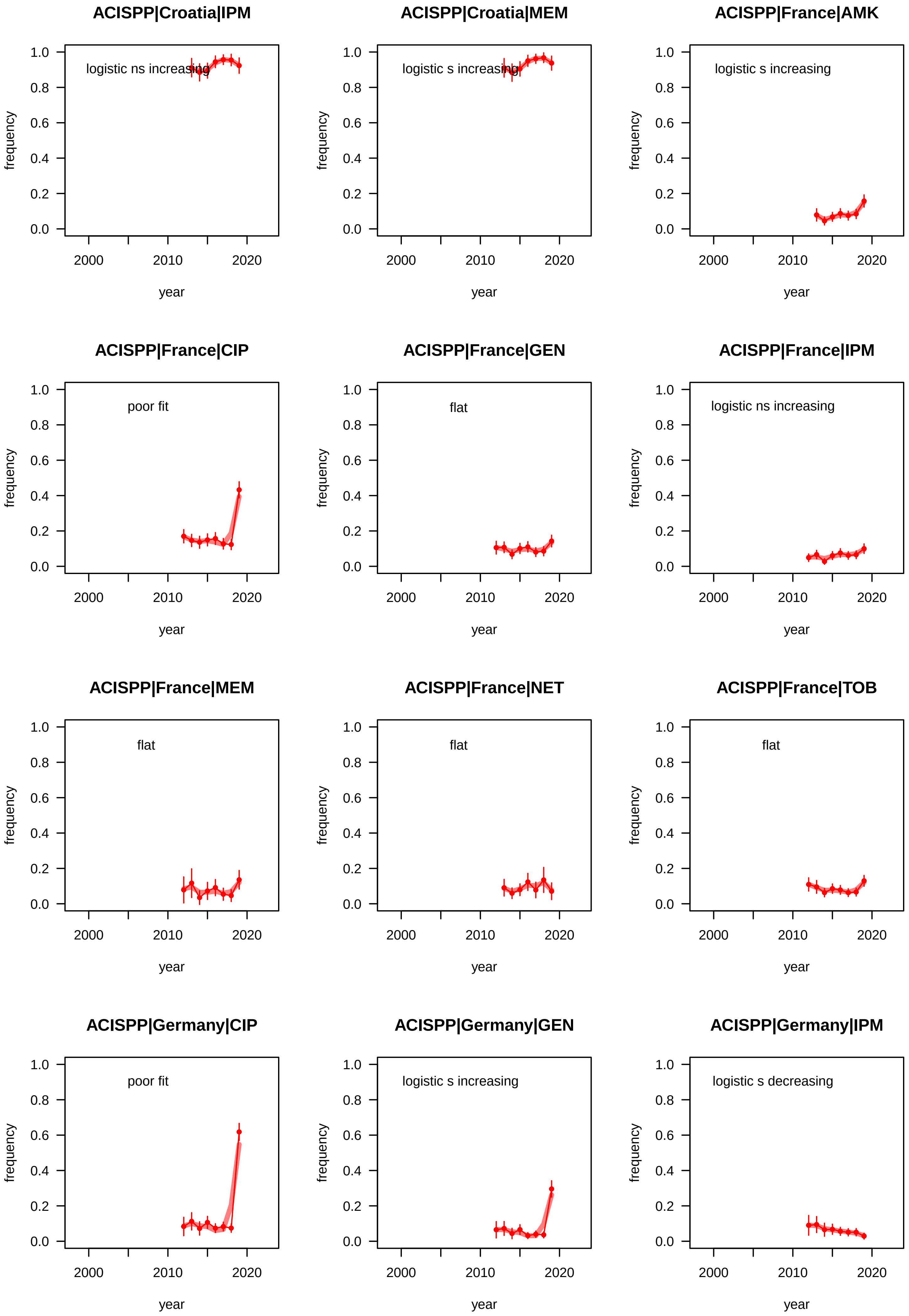

ACISPP|Croatia|IPM
logistic ns increasing
ACISPP|Croatia|MEM
logistic s increasing
ACISPP|France|AMK
logistic s increasing
ACISPP|France|CIP
poor fit
ACISPP|France|GEN
flat
ACISPP|France|IPM
logistic ns increasing
ACISPP|France|MEM
flat
ACISPP|France|NET
flat
ACISPP|France|TOB
flat
ACISPP|Germany|CIP
poor fit
ACISPP|Germany|GEN
logistic s increasing
ACISPP|Germany|IPM
logistic s decreasing
frequency
0.0
0.2
0.4
0.6
0.8
1.0
year
2000
2010
2020

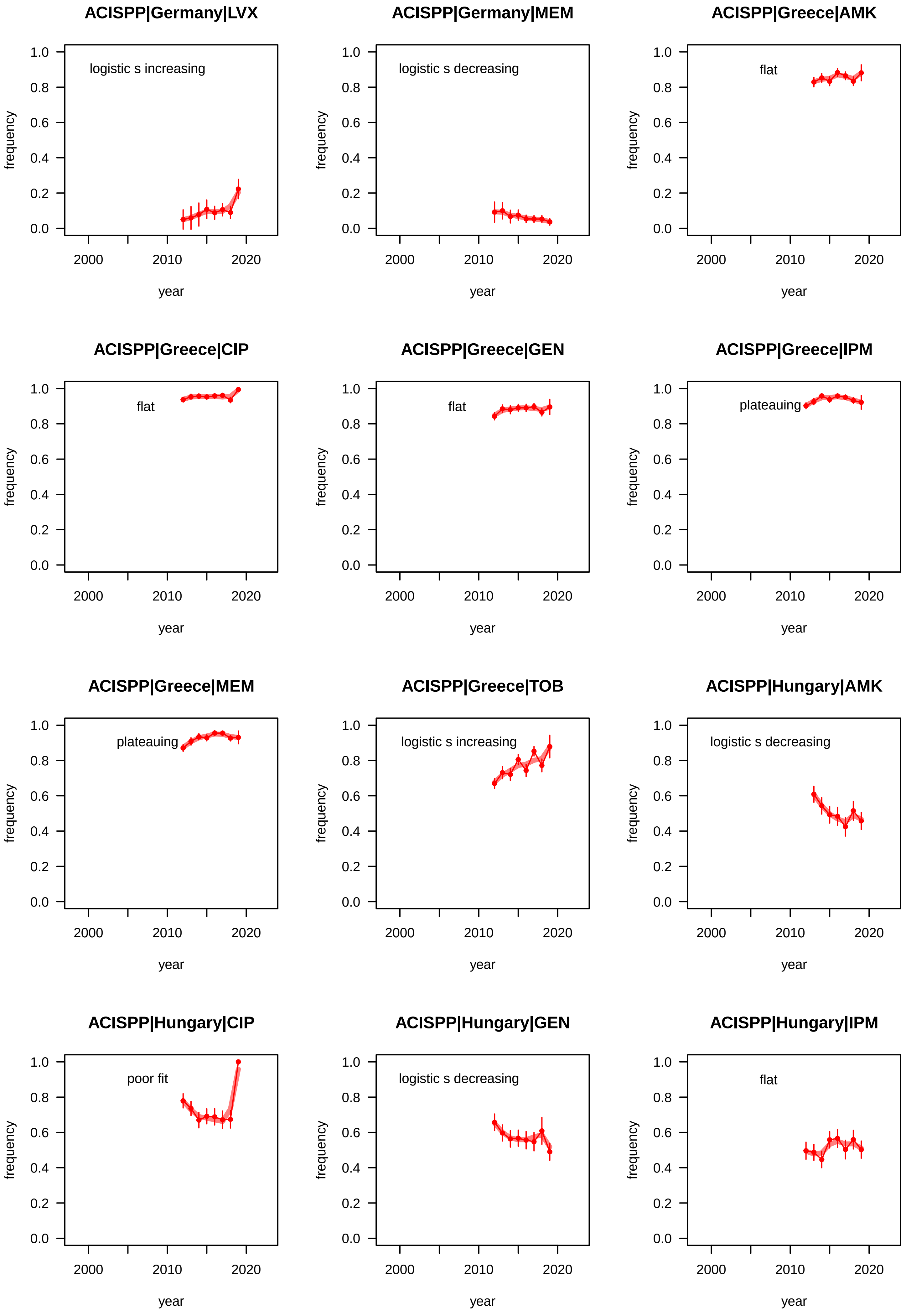

ACISPP|Germany|LVX
logistic s increasing
ACISPP|Germany|MEM
logistic s decreasing
ACISPP|Greece|AMK
flat
ACISPP|Greece|CIP
flat
ACISPP|Greece|GEN
flat
ACISPP|Greece|IPM
plateauing
ACISPP|Greece|MEM
plateauing
ACISPP|Greece|TOB
logistic s increasing
ACISPP|Hungary|AMK
logistic s decreasing
ACISPP|Hungary|CIP
poor fit
ACISPP|Hungary|GEN
logistic s decreasing
ACISPP|Hungary|IPM
flat
frequency
year
0.0
0.2
0.4
0.6
0.8
1.0
2000
2010
2020

ACISPP|Hungary|LVX

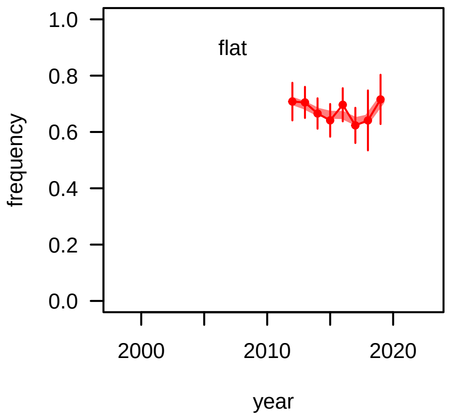

ACISPP|Hungary|MEM

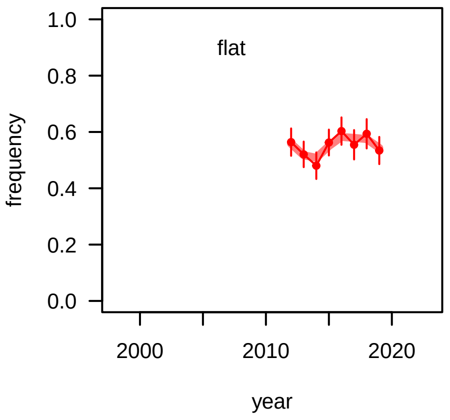

ACISPP|Hungary|TOB

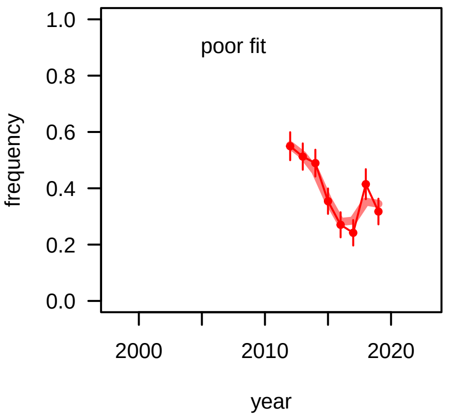

ACISPP|Italy|CIP

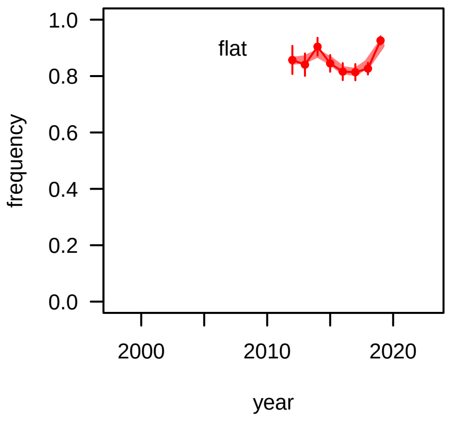

ACISPP|Italy|GEN

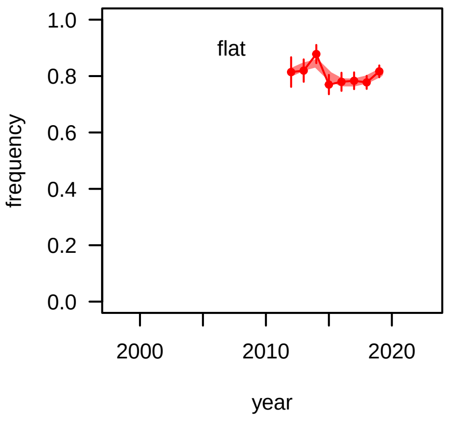

ACISPP|Italy|IPM

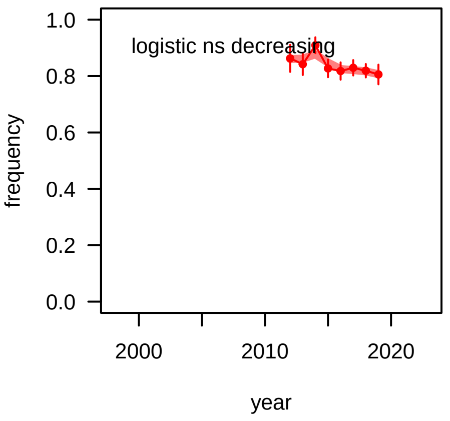

ACISPP|Italy|LVX

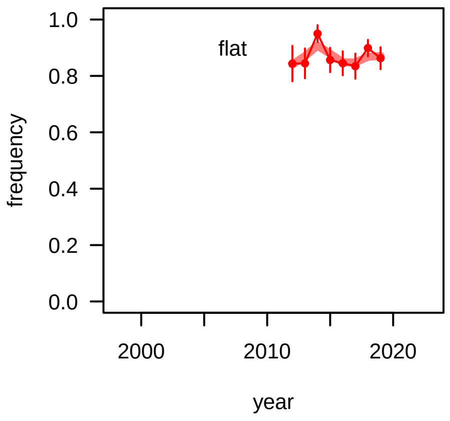

ACISPP|Italy|MEM

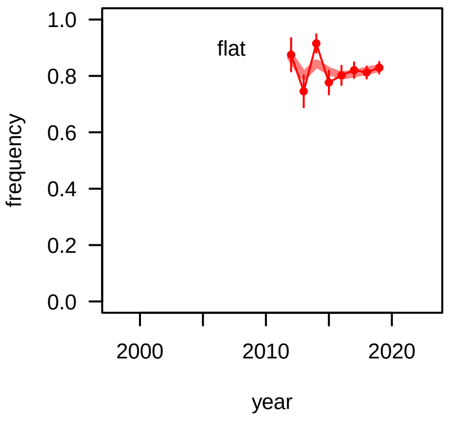

ACISPP|Italy|TOB

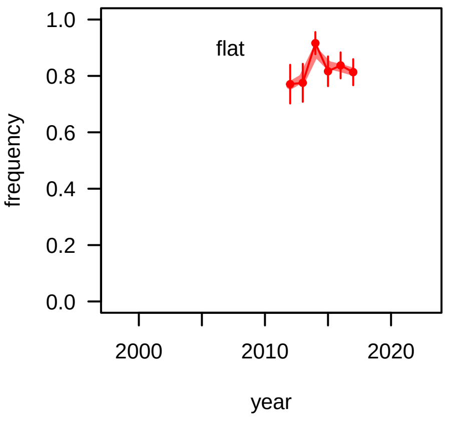

ACISPP|Latvia|AMK

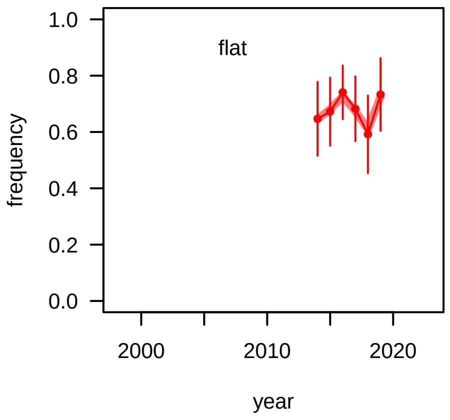

ACISPP|Latvia|GEN

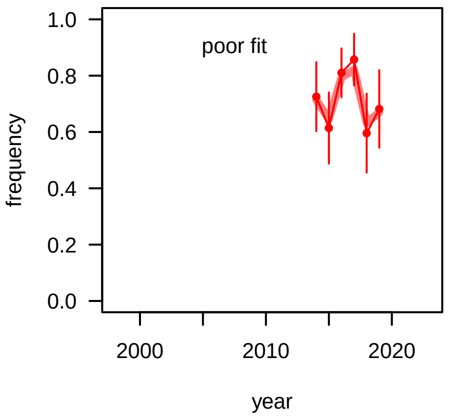

ACISPP|Latvia|IPM

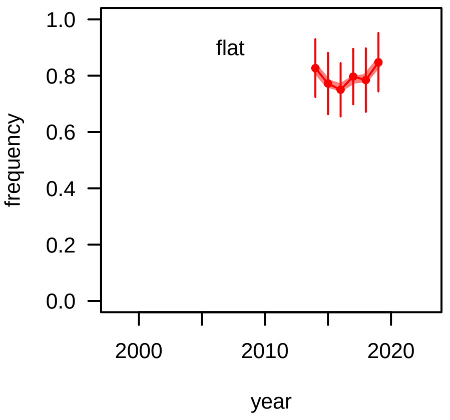

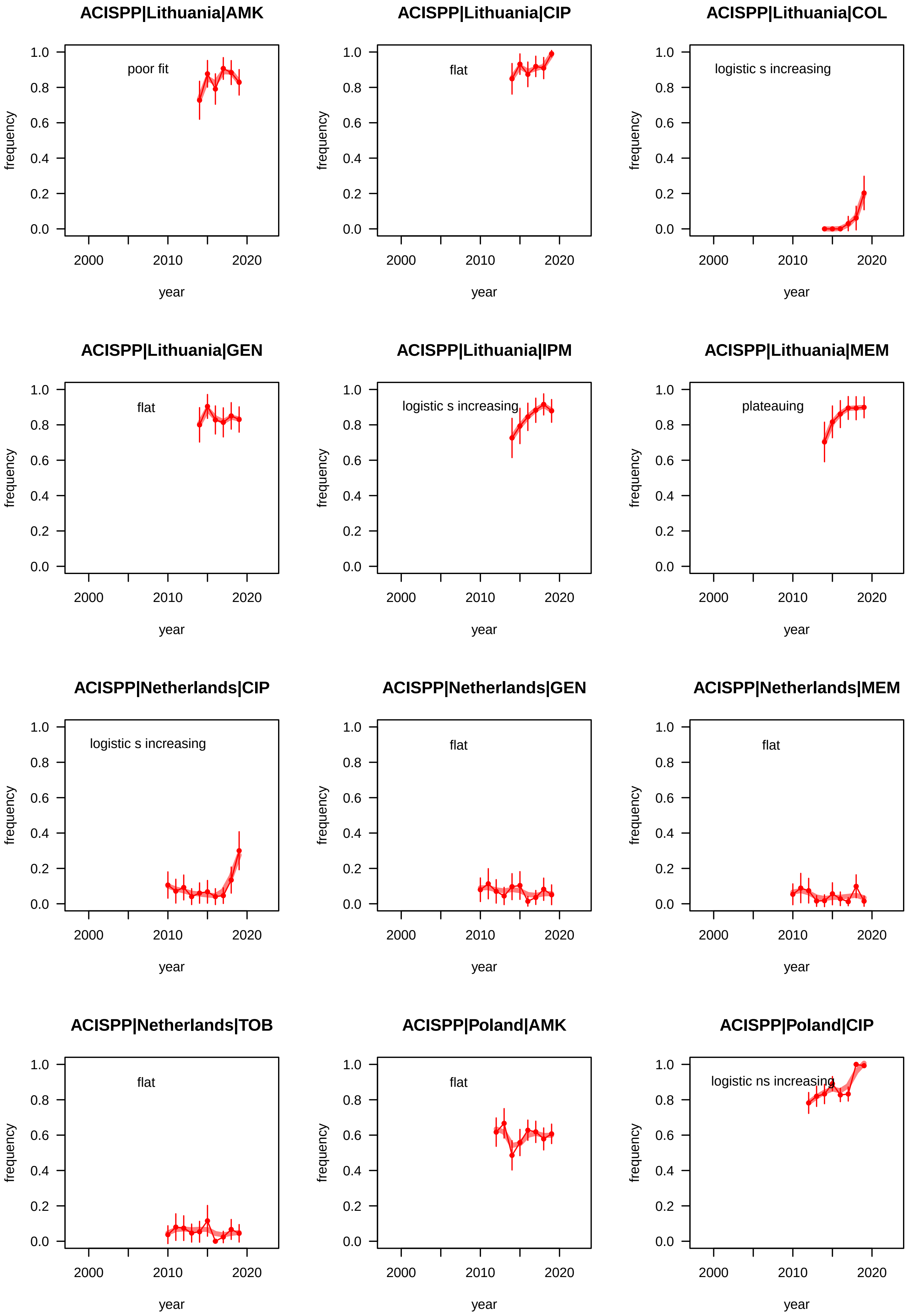
ACISPP|Lithuania|AMK
poor fit
ACISPP|Lithuania|CIP
flat
ACISPP|Lithuania|COL
logistic s increasing
ACISPP|Lithuania|GEN
flat
ACISPP|Lithuania|IPM
logistic s increasing
ACISPP|Lithuania|MEM
plateauing
ACISPP|Netherlands|CIP
logistic s increasing
ACISPP|Netherlands|GEN
flat
ACISPP|Netherlands|MEM
flat
ACISPP|Netherlands|TOB
flat
ACISPP|Poland|AMK
flat
ACISPP|Poland|CIP
logistic ns increasing
frequency
year
0.0
0.2
0.4
0.6
0.8
1.0
2000
2010
2020

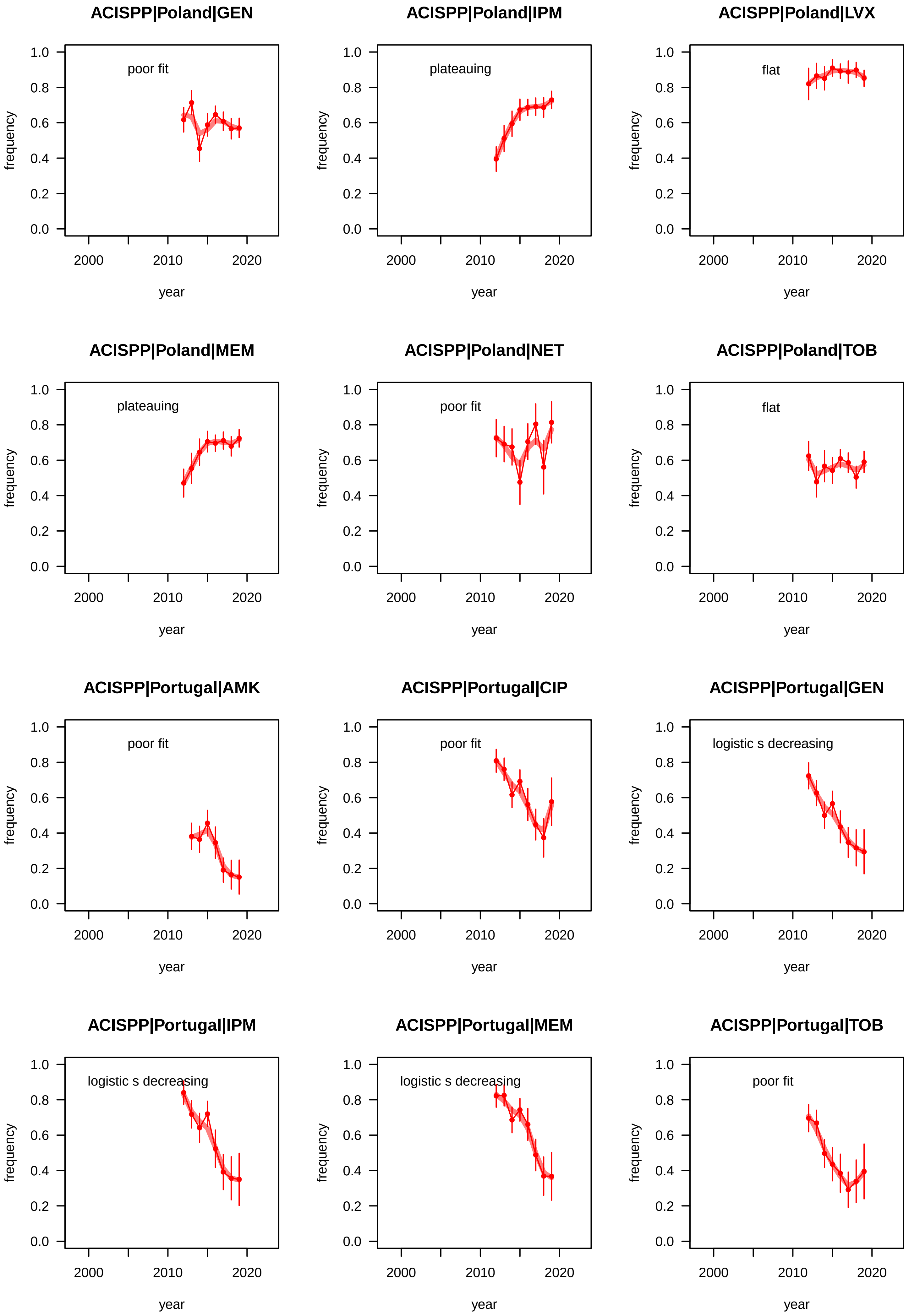
ACISPP|Poland|GEN
poor fit
ACISPP|Poland|IPM
plateauing
ACISPP|Poland|LVX
flat
ACISPP|Poland|MEM
plateauing
ACISPP|Poland|NET
poor fit
ACISPP|Poland|TOB
flat
ACISPP|Portugal|AMK
poor fit
ACISPP|Portugal|CIP
poor fit
ACISPP|Portugal|GEN
logistic s decreasing
ACISPP|Portugal|IPM
logistic s decreasing
ACISPP|Portugal|MEM
logistic s decreasing
ACISPP|Portugal|TOB
poor fit
frequency
0.0
0.2
0.4
0.6
0.8
1.0
year
2000
2010
2020

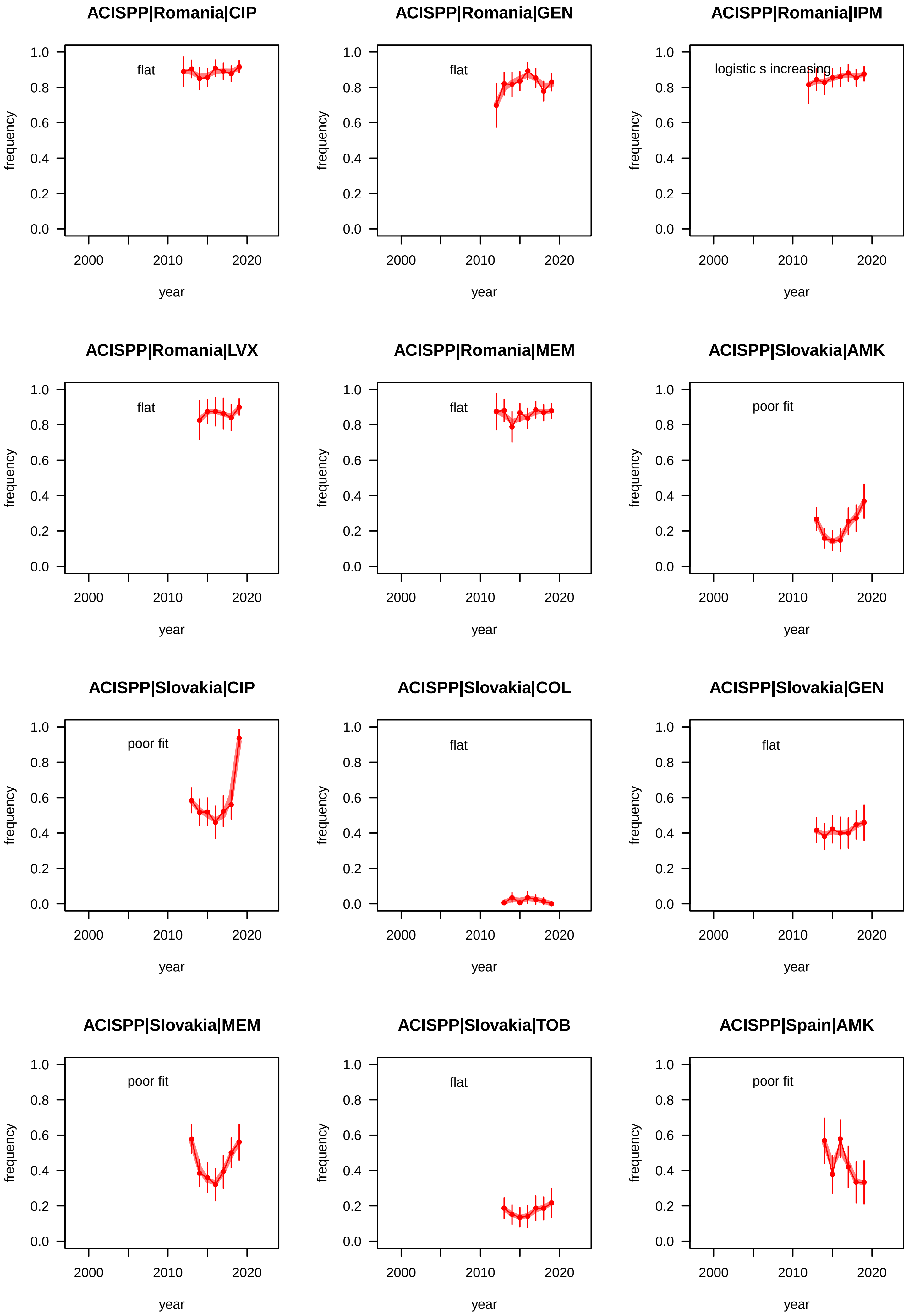
ACISPP|Romania|CIP
flat
ACISPP|Romania|GEN
flat
ACISPP|Romania|IPM
logistic s increasing
ACISPP|Romania|LVX
flat
ACISPP|Romania|MEM
flat
ACISPP|Slovakia|AMK
poor fit
ACISPP|Slovakia|CIP
poor fit
ACISPP|Slovakia|COL
flat
ACISPP|Slovakia|GEN
flat
ACISPP|Slovakia|MEM
poor fit
ACISPP|Slovakia|TOB
flat
ACISPP|Spain|AMK
poor fit
frequency
year
0.0
0.2
0.4
0.6
0.8
1.0
2000
2010
2020

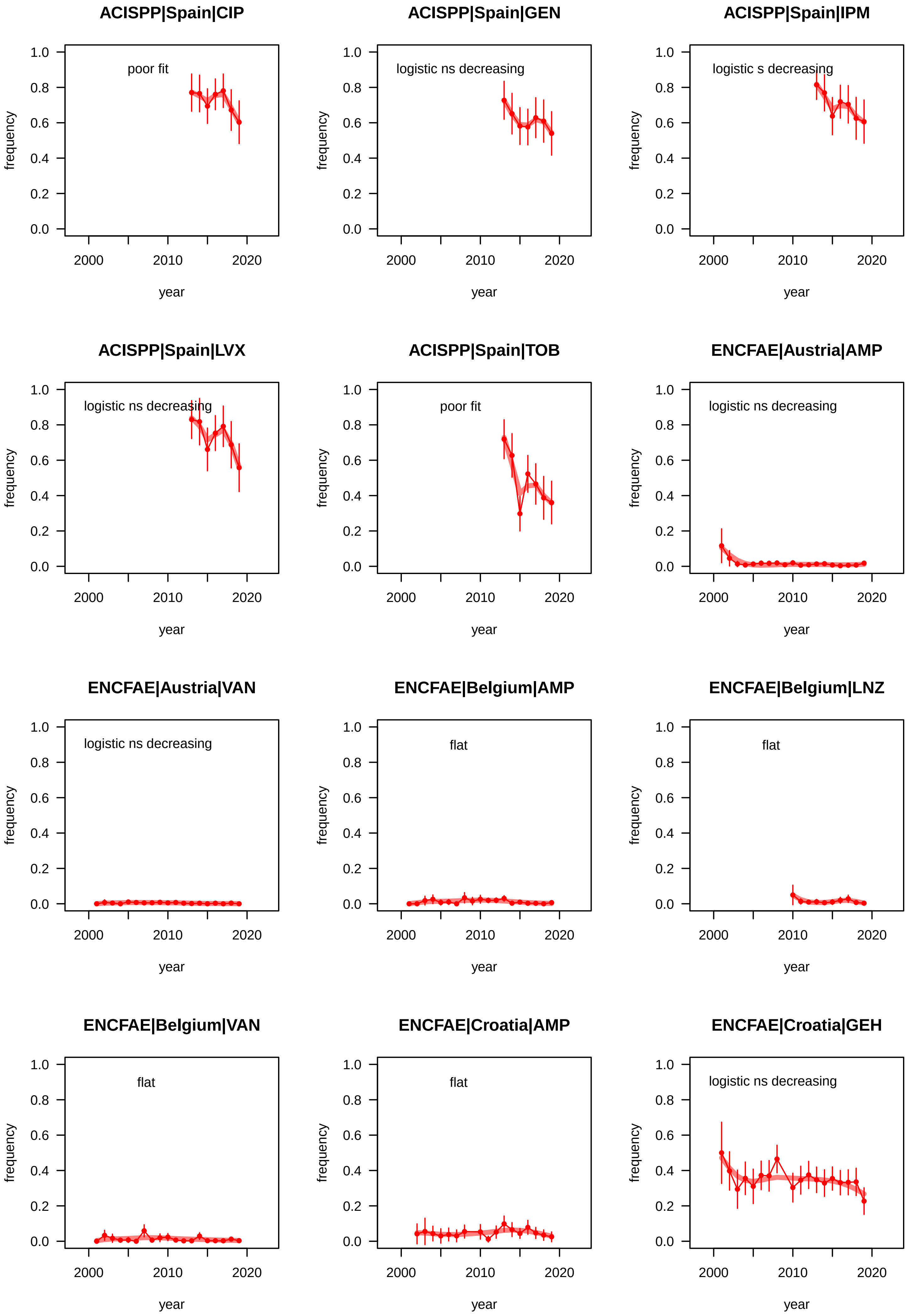
ACISPP|Spain|CIP
poor fit
ACISPP|Spain|GEN
logistic ns decreasing
ACISPP|Spain|IPM
logistic s decreasing
ACISPP|Spain|LVX
logistic ns decreasing
ACISPP|Spain|TOB
poor fit
ENCFAE|Austria|AMP
logistic ns decreasing
ENCFAE|Austria|VAN
logistic ns decreasing
ENCFAE|Belgium|AMP
flat
ENCFAE|Belgium|LNZ
flat
ENCFAE|Belgium|VAN
flat
ENCFAE|Croatia|AMP
flat
ENCFAE|Croatia|GEH
logistic ns decreasing
frequency
year
0.0
0.2
0.4
0.6
0.8
1.0
2000
2010
2020

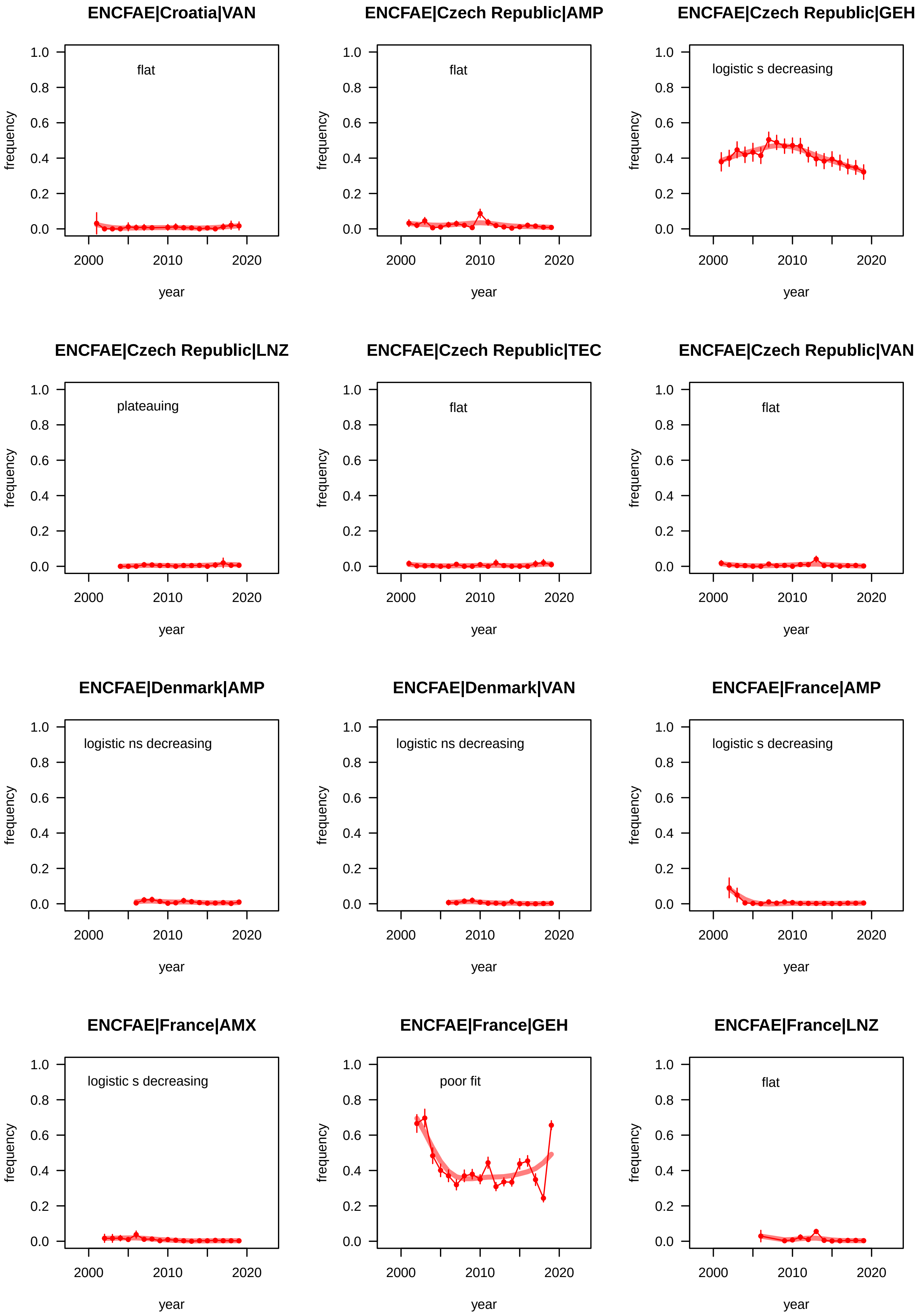
ENCFAE|Croatia|VAN
flat
ENCFAE|Czech Republic|AMP
flat
ENCFAE|Czech Republic|GEH
logistic s decreasing
ENCFAE|Czech Republic|LNZ
plateauing
ENCFAE|Czech Republic|TEC
flat
ENCFAE|Czech Republic|VAN
flat
ENCFAE|Denmark|AMP
logistic ns decreasing
ENCFAE|Denmark|VAN
logistic ns decreasing
ENCFAE|France|AMP
logistic s decreasing
ENCFAE|France|AMX
logistic s decreasing
ENCFAE|France|GEH
poor fit
ENCFAE|France|LNZ
flat
frequency
year
0.0
0.2
0.4
0.6
0.8
1.0
2000
2010
2020

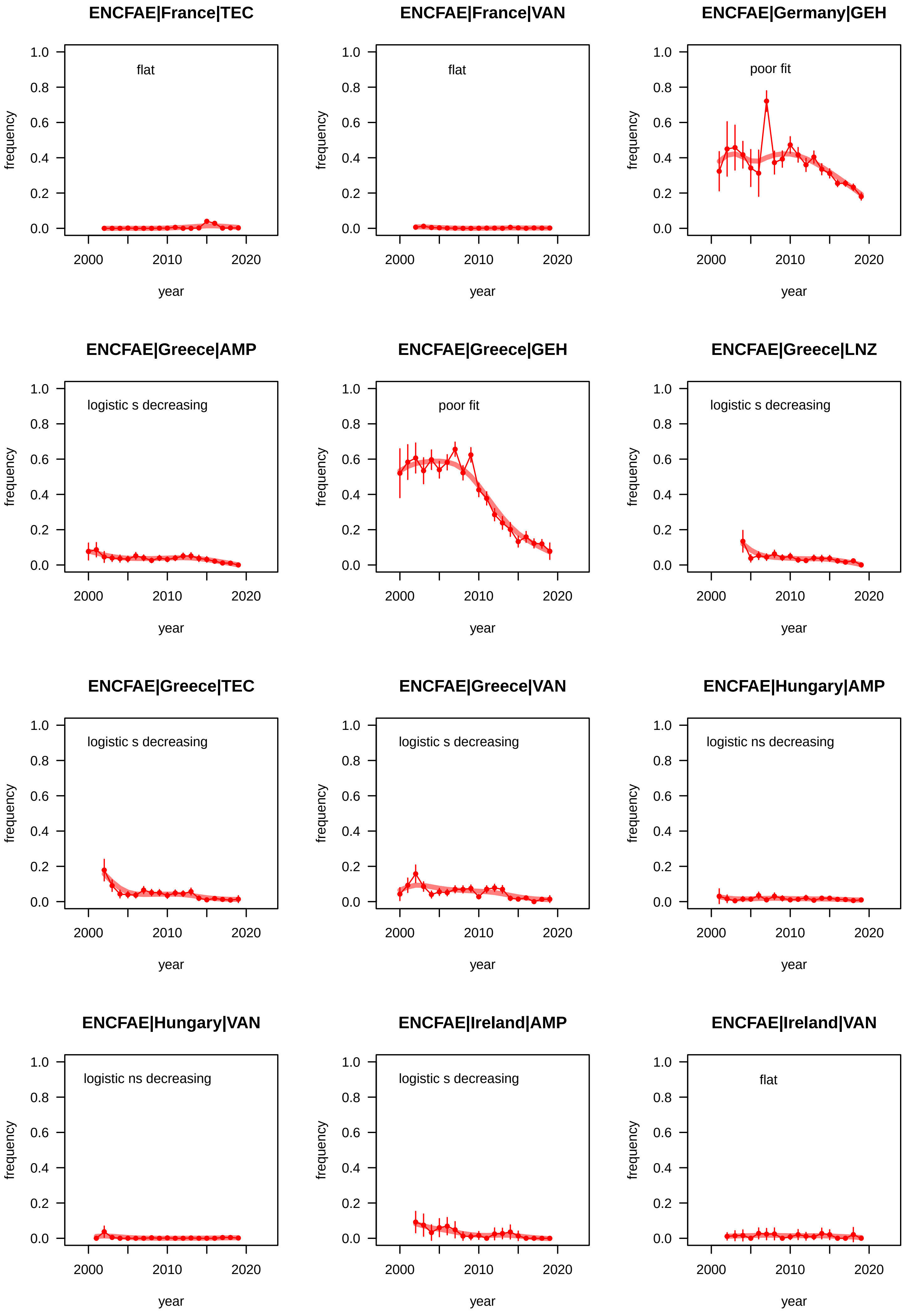
ENCFAE|France|TEC
flat
ENCFAE|France|VAN
flat
ENCFAE|Germany|GEH
poor fit
ENCFAE|Greece|AMP
logistic s decreasing
ENCFAE|Greece|GEH
poor fit
ENCFAE|Greece|LNZ
logistic s decreasing
ENCFAE|Greece|TEC
logistic s decreasing
ENCFAE|Greece|VAN
logistic s decreasing
ENCFAE|Hungary|AMP
logistic ns decreasing
ENCFAE|Hungary|VAN
logistic ns decreasing
ENCFAE|Ireland|AMP
logistic s decreasing
ENCFAE|Ireland|VAN
flat
frequency
0.0
0.2
0.4
0.6
0.8
1.0
year
2000
2010
2020

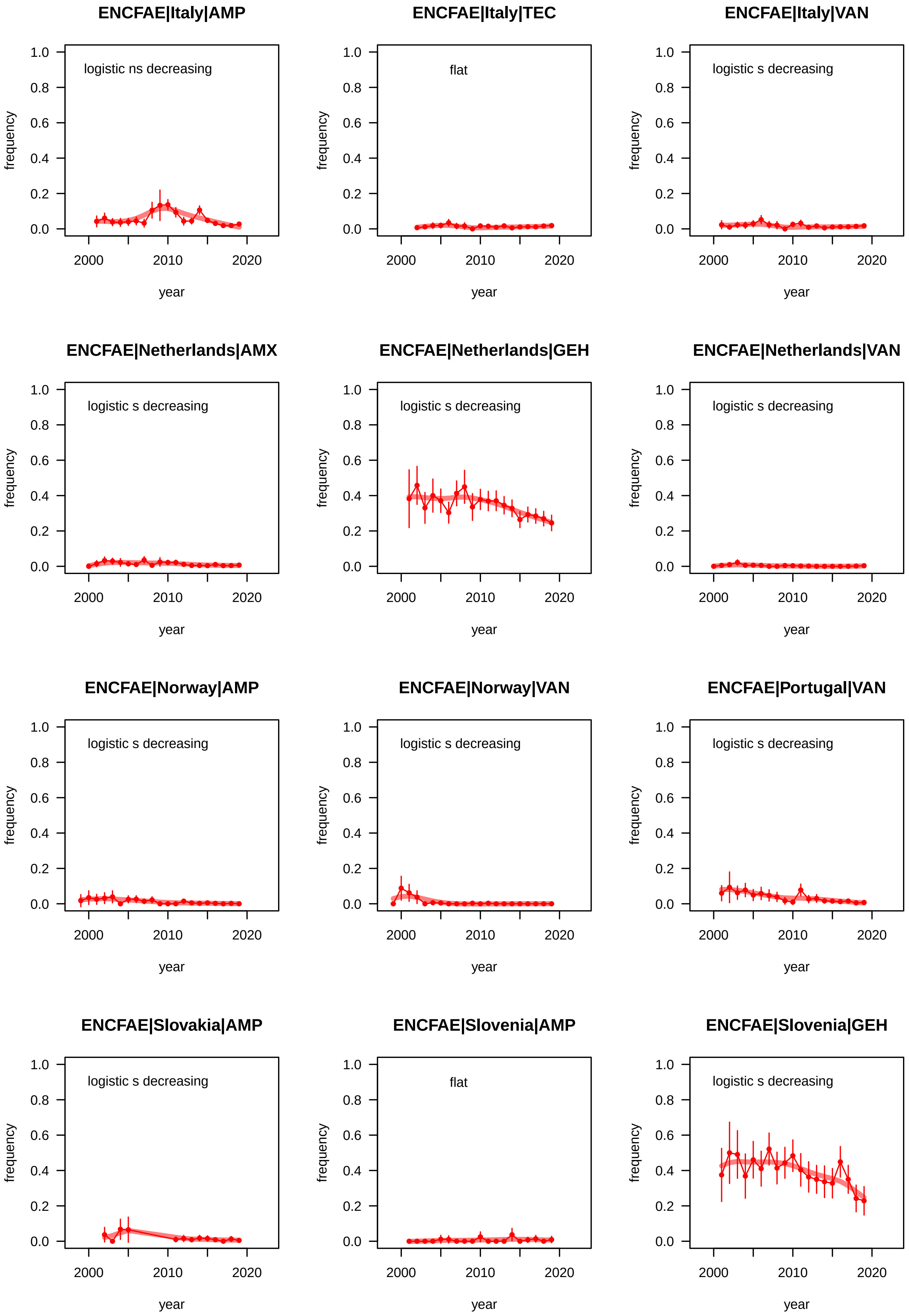
ENCFAE|Italy|AMP
logistic ns decreasing
ENCFAE|Italy|TEC
flat
ENCFAE|Italy|VAN
logistic s decreasing
ENCFAE|Netherlands|AMX
logistic s decreasing
ENCFAE|Netherlands|GEH
logistic s decreasing
ENCFAE|Netherlands|VAN
logistic s decreasing
ENCFAE|Norway|AMP
logistic s decreasing
ENCFAE|Norway|VAN
logistic s decreasing
ENCFAE|Portugal|VAN
logistic s decreasing
ENCFAE|Slovakia|AMP
logistic s decreasing
ENCFAE|Slovenia|AMP
flat
ENCFAE|Slovenia|GEH
logistic s decreasing
frequency
0.0
0.2
0.4
0.6
0.8
1.0
year
2000
2010
2020

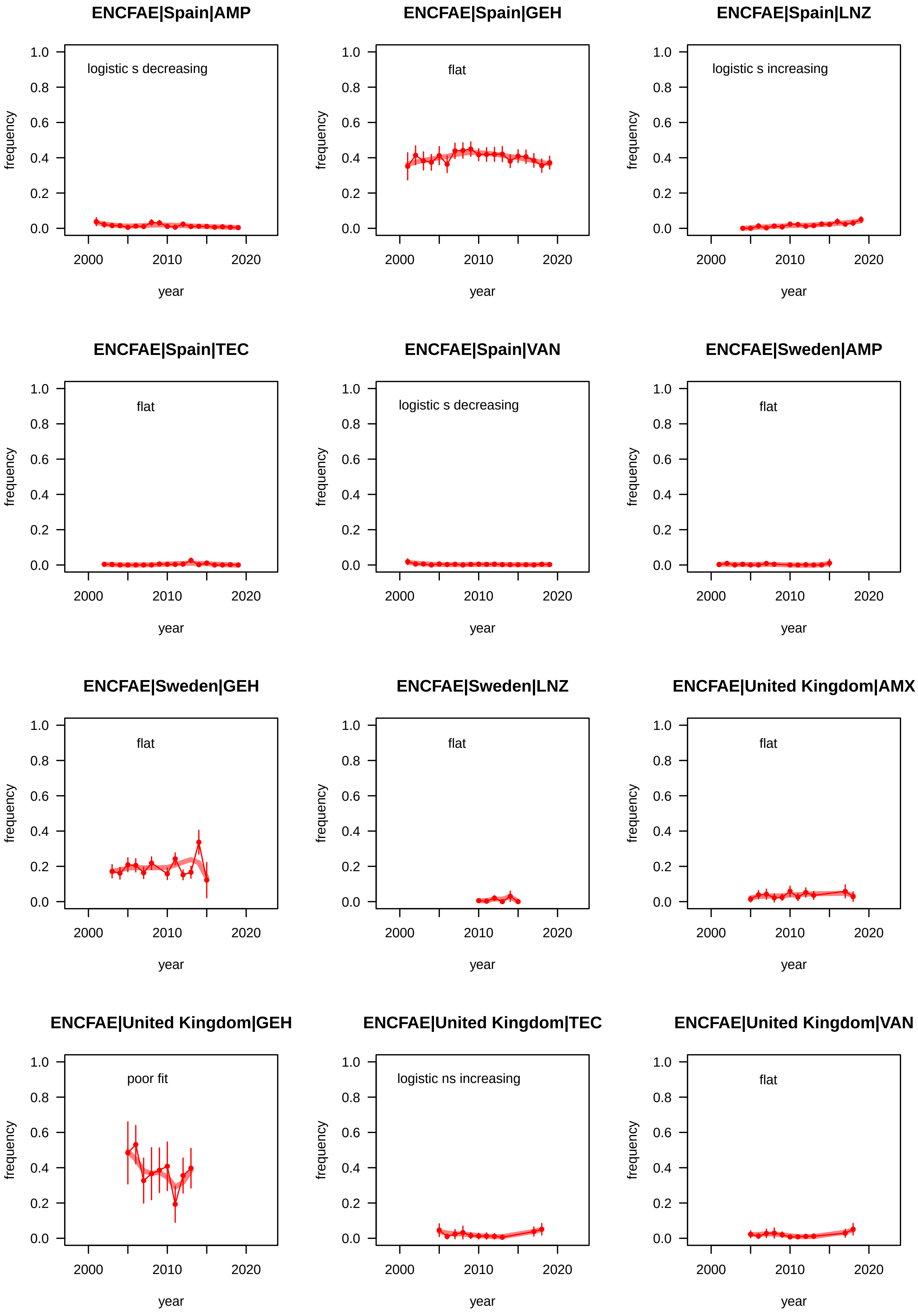
ENCFAE|Spain|AMP
logistic s decreasing
ENCFAE|Spain|GEH
flat
ENCFAE|Spain|LNZ
logistic s increasing
ENCFAE|Spain|TEC
flat
ENCFAE|Spain|VAN
logistic s decreasing
ENCFAE|Sweden|AMP
flat
ENCFAE|Sweden|GEH
flat
ENCFAE|Sweden|LNZ
flat
ENCFAE|United Kingdom|AMX
flat
ENCFAE|United Kingdom|GEH
poor fit
ENCFAE|United Kingdom|TEC
logistic ns increasing
ENCFAE|United Kingdom|VAN
flat
frequency
year
0.0
0.2
0.4
0.6
0.8
1.0
2000
2010
2020

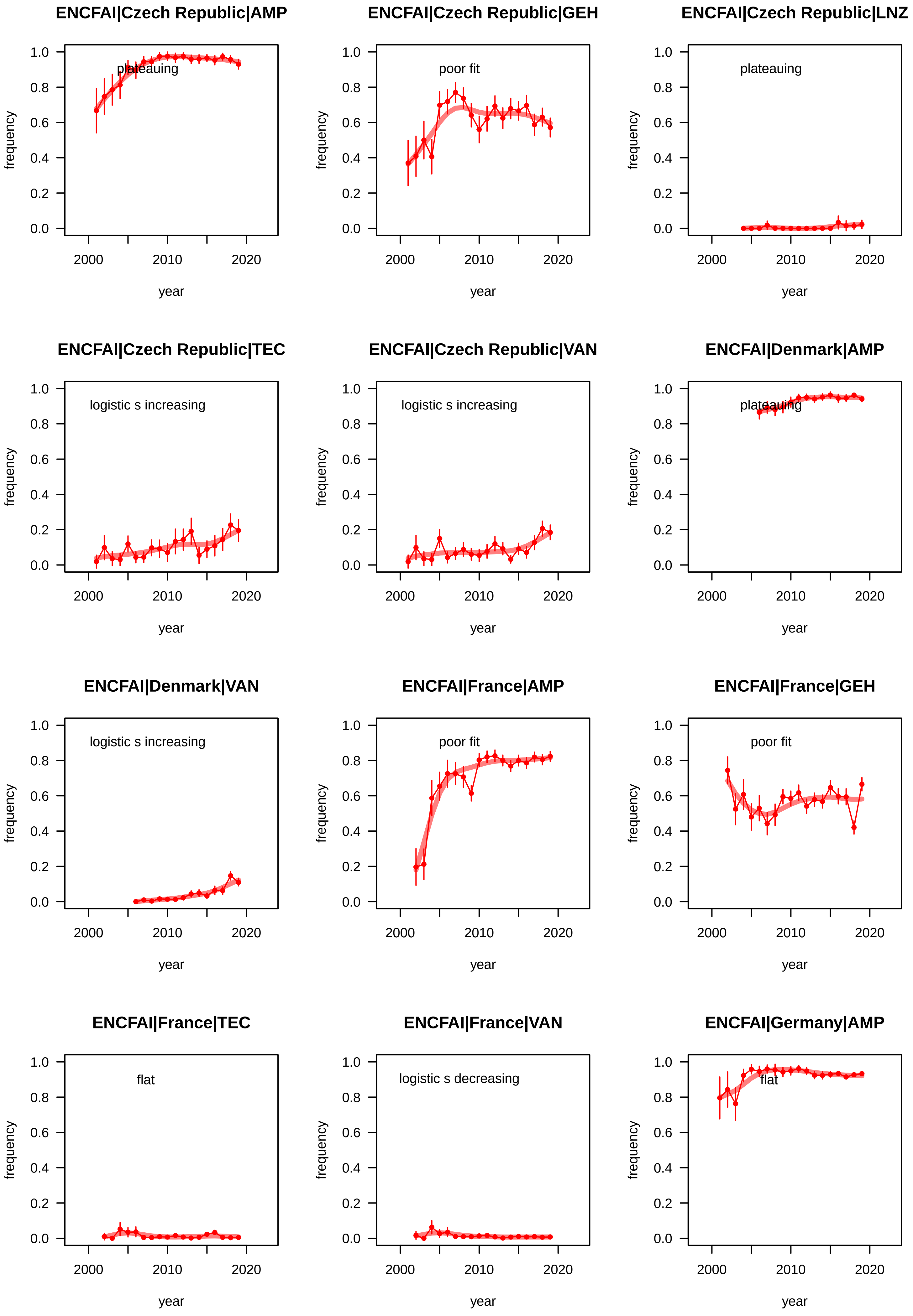

ENCFAI|Czech Republic|AMP
plateauing
ENCFAI|Czech Republic|GEH
poor fit
ENCFAI|Czech Republic|LNZ
plateauing
ENCFAI|Czech Republic|TEC
logistic s increasing
ENCFAI|Czech Republic|VAN
logistic s increasing
ENCFAI|Denmark|AMP
plateauing
ENCFAI|Denmark|VAN
logistic s increasing
ENCFAI|France|AMP
poor fit
ENCFAI|France|GEH
poor fit
ENCFAI|France|TEC
flat
ENCFAI|France|VAN
logistic s decreasing
ENCFAI|Germany|AMP
flat
frequency
year
0.0
0.2
0.4
0.6
0.8
1.0
2000
2010
2020

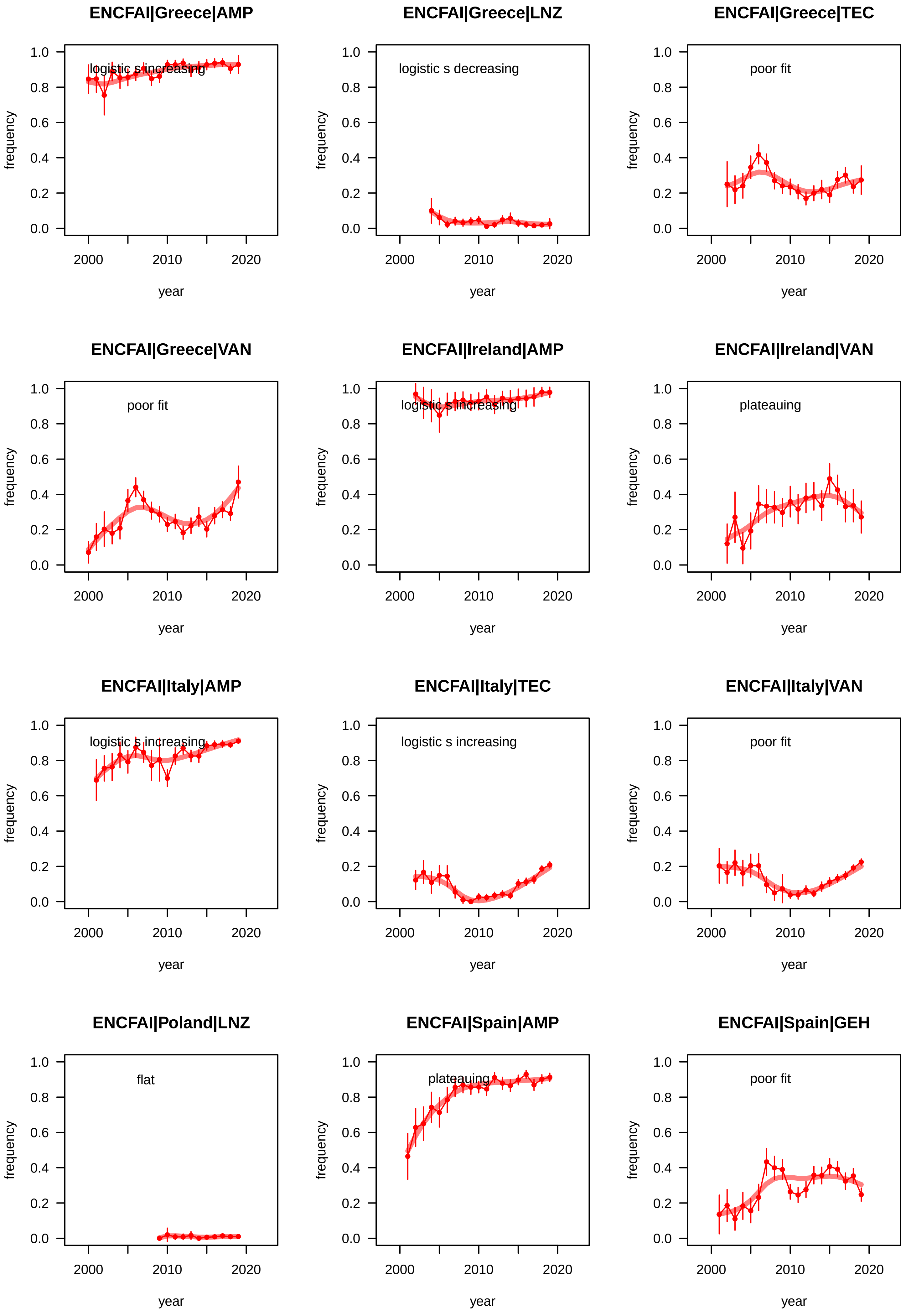
ENCFAI|Greece|AMP
logistic s increasing
ENCFAI|Greece|LNZ
logistic s decreasing
ENCFAI|Greece|TEC
poor fit
ENCFAI|Greece|VAN
poor fit
ENCFAI|Ireland|AMP
logistic s increasing
ENCFAI|Ireland|VAN
plateauing
ENCFAI|Italy|AMP
logistic s increasing
ENCFAI|Italy|TEC
logistic s increasing
ENCFAI|Italy|VAN
poor fit
ENCFAI|Poland|LNZ
flat
ENCFAI|Spain|AMP
plateauing
ENCFAI|Spain|GEH
poor fit
frequency
year
0.0
0.2
0.4
0.6
0.8
1.0
2000
2010
2020

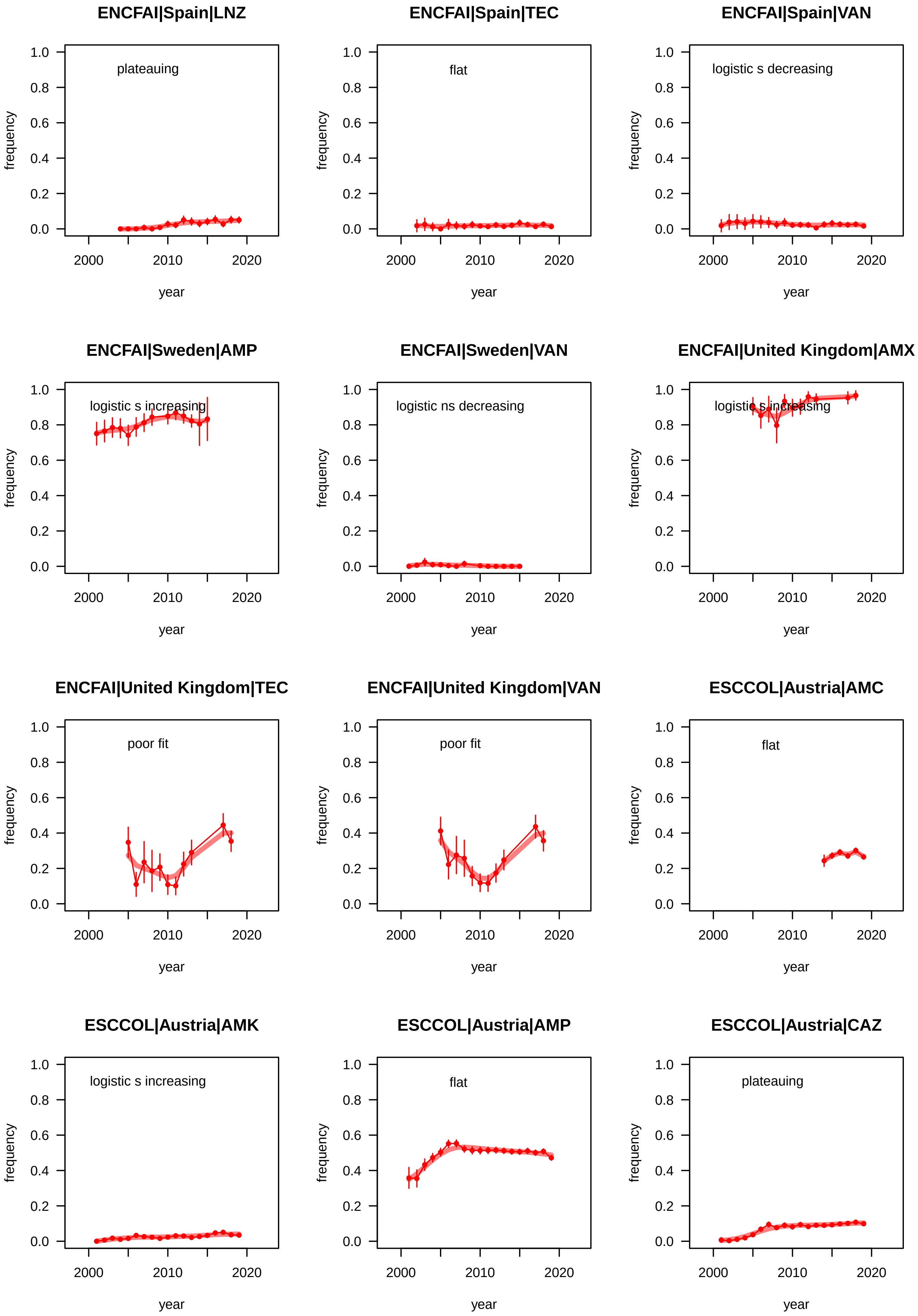
ENCFAI|Spain|LNZ
plateauing
ENCFAI|Spain|TEC
flat
ENCFAI|Spain|VAN
logistic s decreasing
ENCFAI|Sweden|AMP
logistic s increasing
ENCFAI|Sweden|VAN
logistic ns decreasing
ENCFAI|United Kingdom|AMX
logistic s increasing
ENCFAI|United Kingdom|TEC
poor fit
ENCFAI|United Kingdom|VAN
poor fit
ESCCOL|Austria|AMC
flat
ESCCOL|Austria|AMK
logistic s increasing
ESCCOL|Austria|AMP
flat
ESCCOL|Austria|CAZ
plateauing
frequency
year
0.0
0.2
0.4
0.6
0.8
1.0
2000
2010
2020

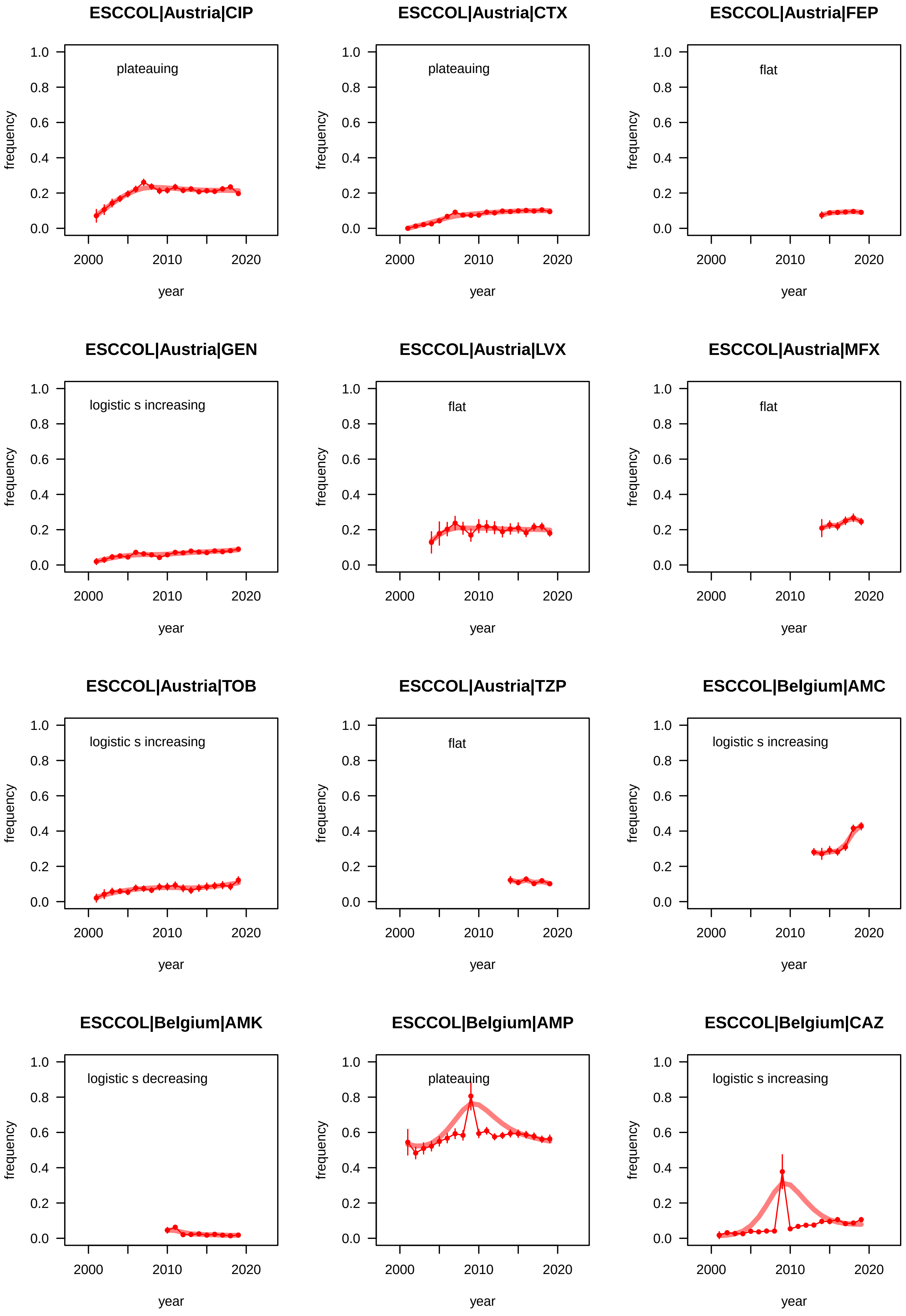
ESCCOL|Austria|CIP
plateauing
ESCCOL|Austria|CTX
plateauing
ESCCOL|Austria|FEP
flat
ESCCOL|Austria|GEN
logistic s increasing
ESCCOL|Austria|LVX
flat
ESCCOL|Austria|MFX
flat
ESCCOL|Austria|TOB
logistic s increasing
ESCCOL|Austria|TZP
flat
ESCCOL|Belgium|AMC
logistic s increasing
ESCCOL|Belgium|AMK
logistic s decreasing
ESCCOL|Belgium|AMP
plateauing
ESCCOL|Belgium|CAZ
logistic s increasing
frequency
year
0.0
0.2
0.4
0.6
0.8
1.0
2000
2010
2020

ESCCOL|Belgium|CIP

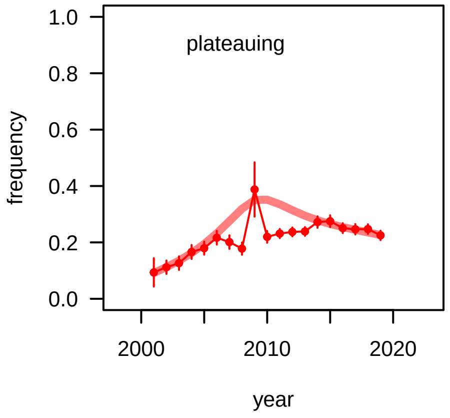

ESCCOL|Belgium|COL

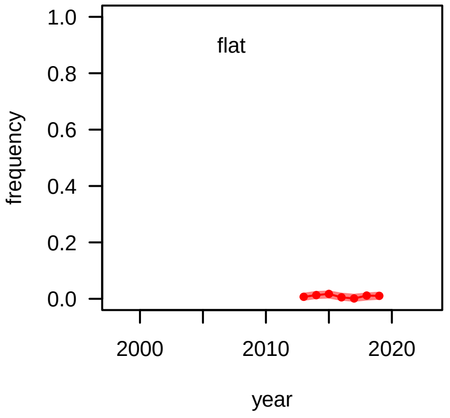

ESCCOL|Belgium|CRO

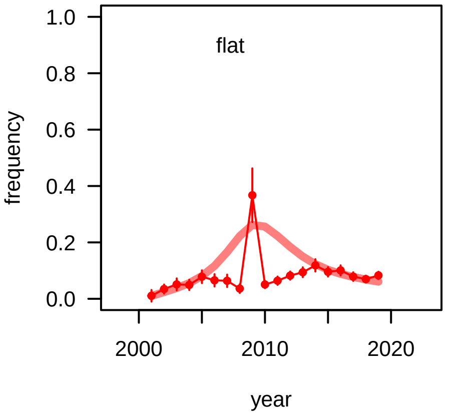

ESCCOL|Belgium|CTX

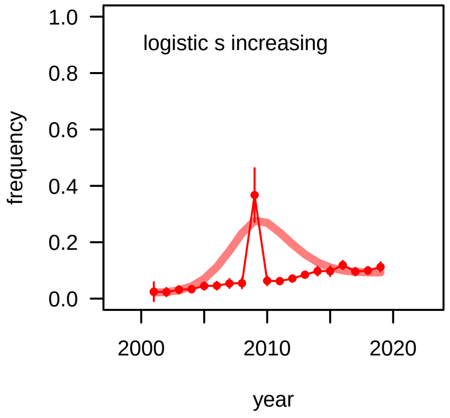

ESCCOL|Belgium|ETP

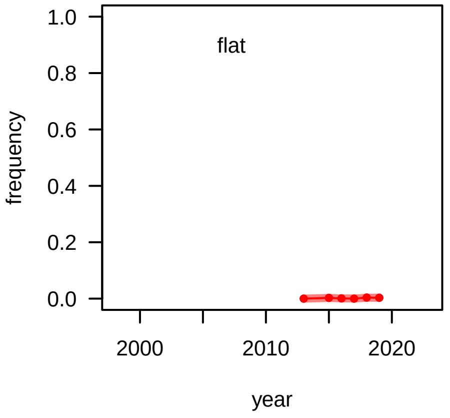

ESCCOL|Belgium|FEP

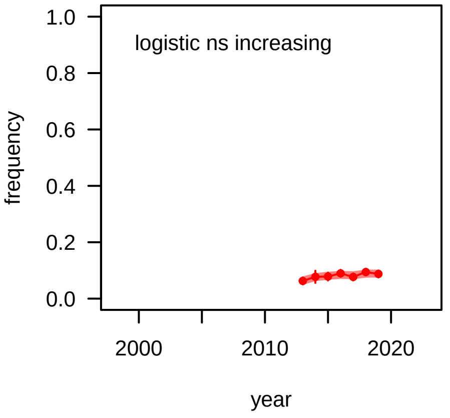

ESCCOL|Belgium|GEN

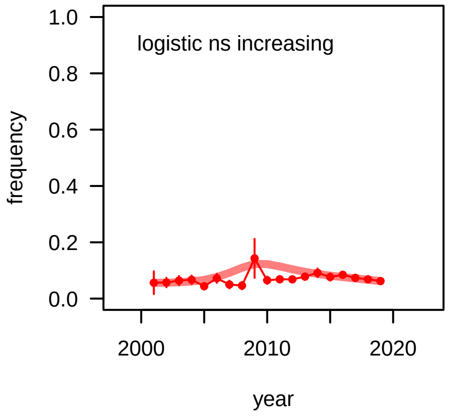

ESCCOL|Belgium|LVX

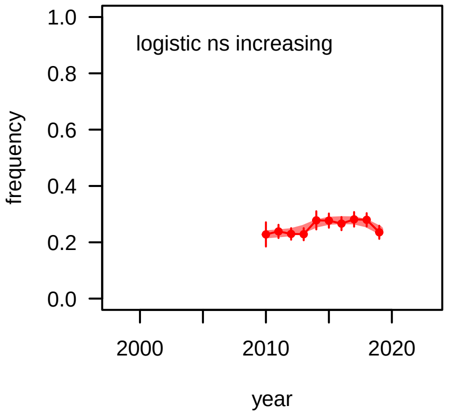

ESCCOL|Belgium|MEM

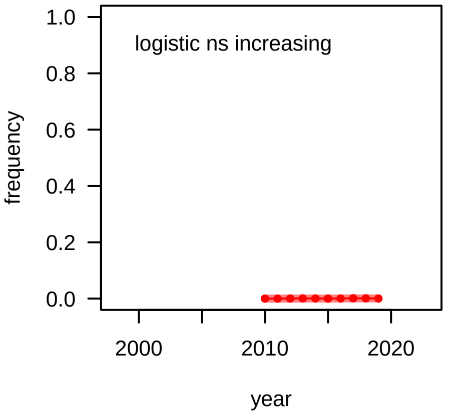

ESCCOL|Belgium|TOB

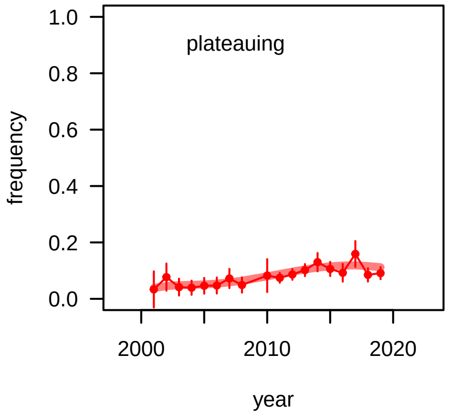

ESCCOL|Belgium|TZP

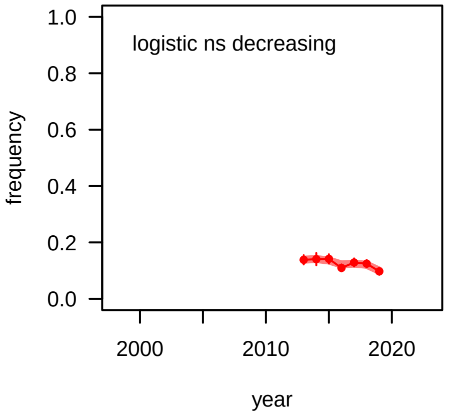

ESCCOL|Bulgaria|AMK

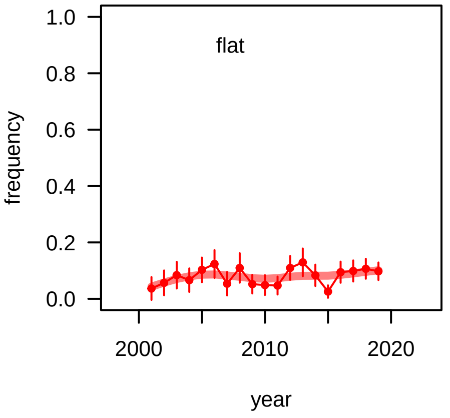

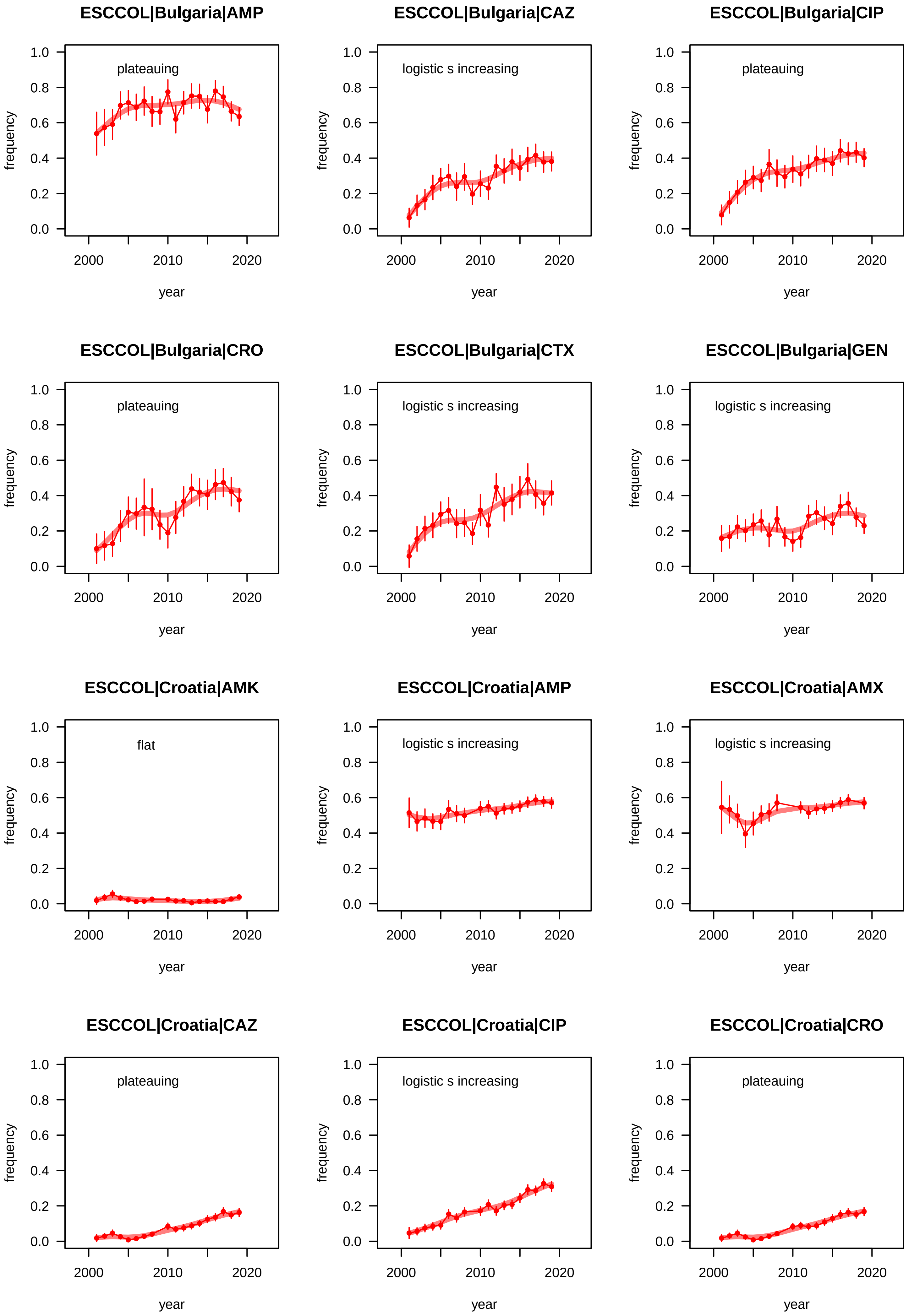
ESCCOL|Bulgaria|AMP
plateauing
ESCCOL|Bulgaria|CAZ
logistic s increasing
ESCCOL|Bulgaria|CIP
plateauing
ESCCOL|Bulgaria|CRO
plateauing
ESCCOL|Bulgaria|CTX
logistic s increasing
ESCCOL|Bulgaria|GEN
logistic s increasing
ESCCOL|Croatia|AMK
flat
ESCCOL|Croatia|AMP
logistic s increasing
ESCCOL|Croatia|AMX
logistic s increasing
ESCCOL|Croatia|CAZ
plateauing
ESCCOL|Croatia|CIP
logistic s increasing
ESCCOL|Croatia|CRO
plateauing
frequency
year
0.0
0.2
0.4
0.6
0.8
1.0
2000
2010
2020

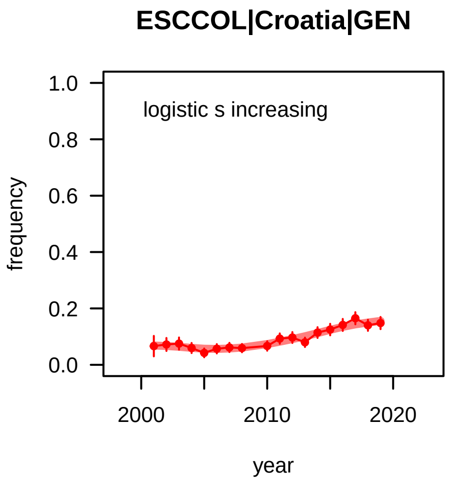
ESCCOL|Croatia|GEN
logistic s increasing
frequency
year
0.0 0.2 0.4 0.6 0.8 1.0
2000 2010 2020

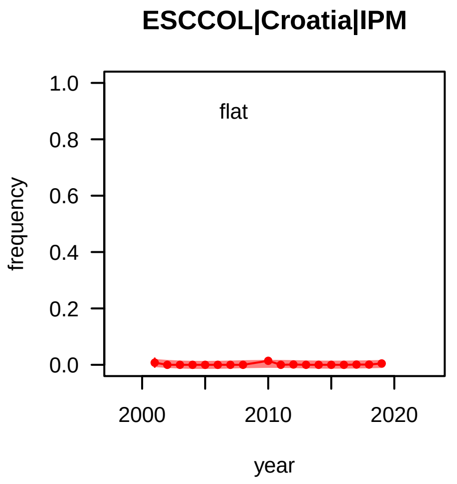
ESCCOL|Croatia|IPM
flat
frequency
year
0.0 0.2 0.4 0.6 0.8 1.0
2000 2010 2020

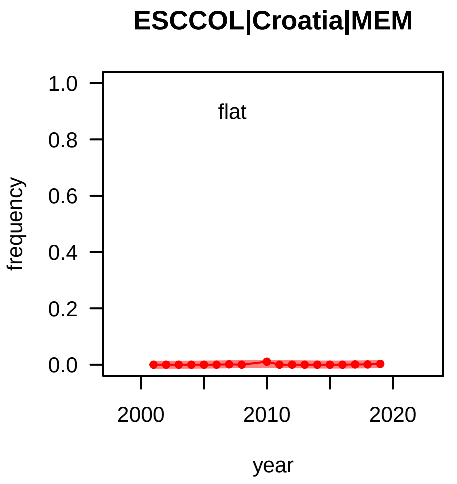
ESCCOL|Croatia|MEM
flat
frequency
year
0.0 0.2 0.4 0.6 0.8 1.0
2000 2010 2020

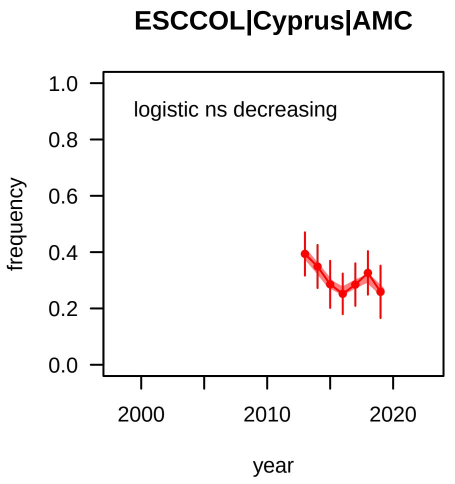
ESCCOL|Cyprus|AMC
logistic ns decreasing
frequency
year
0.0 0.2 0.4 0.6 0.8 1.0
2000 2010 2020

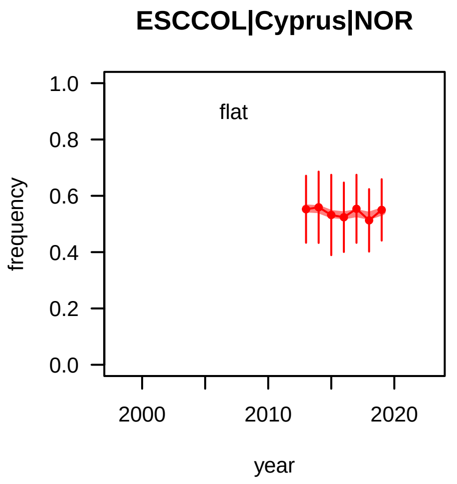
ESCCOL|Cyprus|NOR
flat
frequency
year
0.0 0.2 0.4 0.6 0.8 1.0
2000 2010 2020

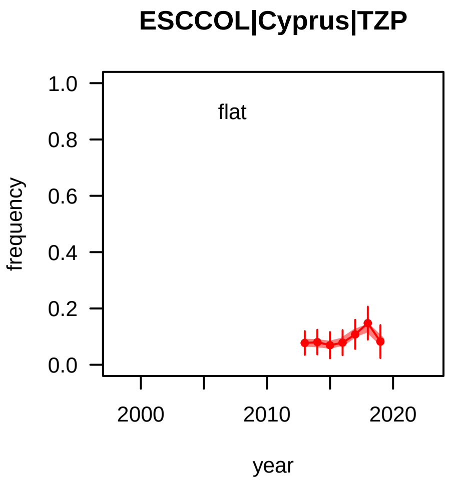
ESCCOL|Cyprus|TZP
flat
frequency
year
0.0 0.2 0.4 0.6 0.8 1.0
2000 2010 2020

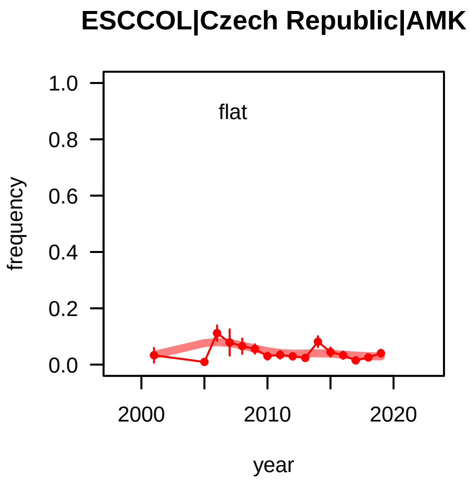
ESCCOL|Czech Republic|AMK
flat
frequency
year
0.0 0.2 0.4 0.6 0.8 1.0
2000 2010 2020

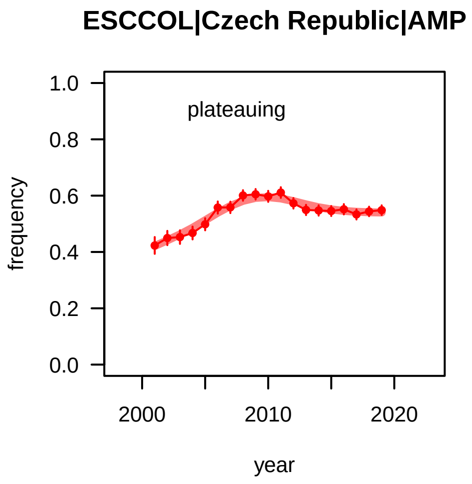
ESCCOL|Czech Republic|AMP
plateauing
frequency
year
0.0 0.2 0.4 0.6 0.8 1.0
2000 2010 2020

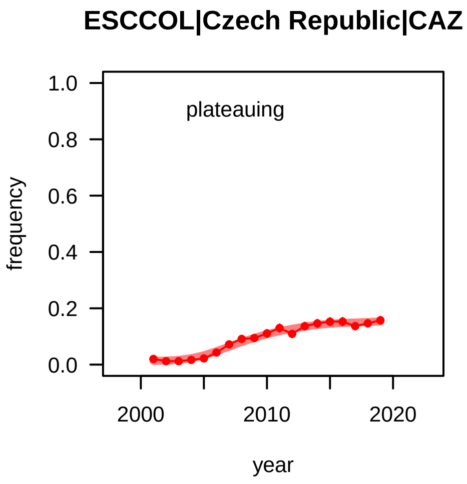
ESCCOL|Czech Republic|CAZ
plateauing
frequency
year
0.0 0.2 0.4 0.6 0.8 1.0
2000 2010 2020

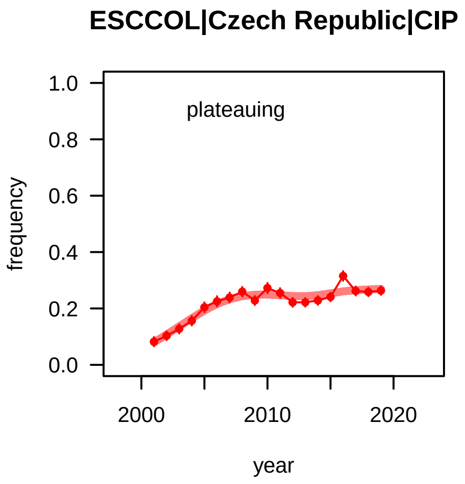
ESCCOL|Czech Republic|CIP
plateauing
frequency
year
0.0 0.2 0.4 0.6 0.8 1.0
2000 2010 2020

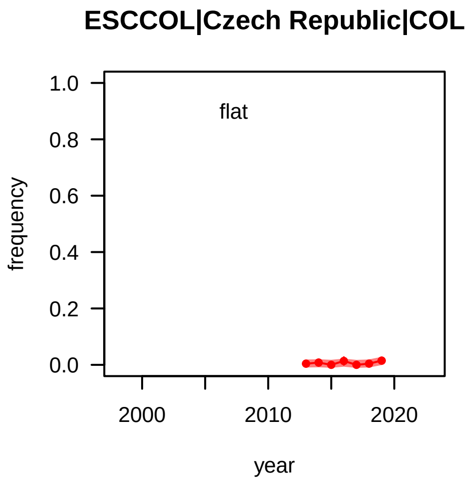
ESCCOL|Czech Republic|COL
flat
frequency
year
0.0 0.2 0.4 0.6 0.8 1.0
2000 2010 2020

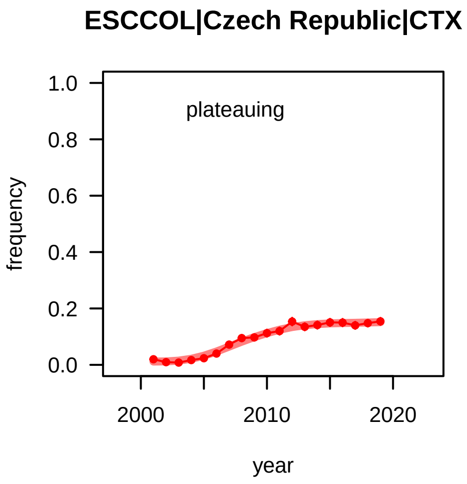
ESCCOL|Czech Republic|CTX
plateauing
frequency
year
0.0 0.2 0.4 0.6 0.8 1.0
2000 2010 2020

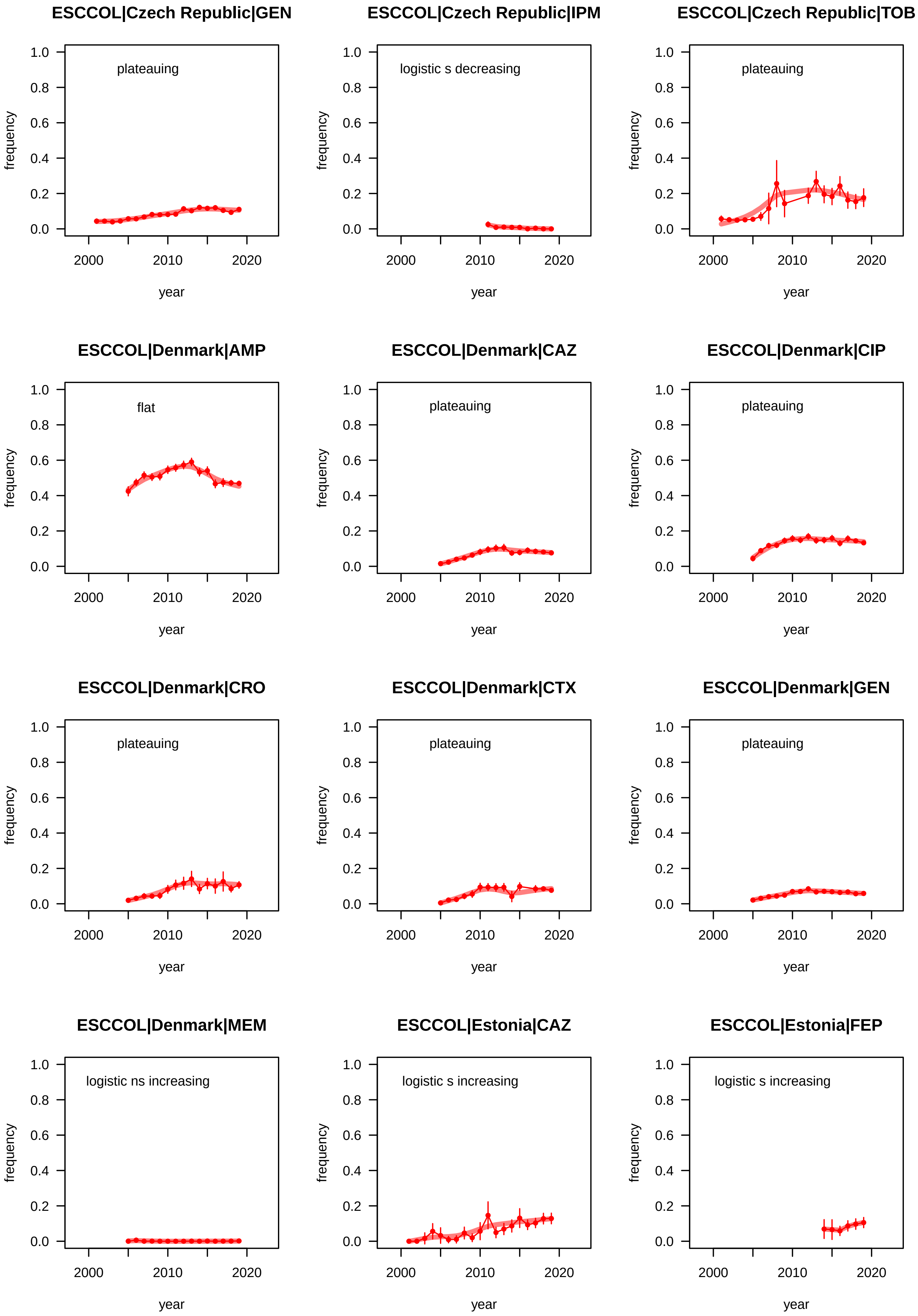
ESCCOL|Czech Republic|GEN
plateauing
ESCCOL|Czech Republic|IPM
logistic s decreasing
ESCCOL|Czech Republic|TOB
plateauing
ESCCOL|Denmark|AMP
flat
ESCCOL|Denmark|CAZ
plateauing
ESCCOL|Denmark|CIP
plateauing
ESCCOL|Denmark|CRO
plateauing
ESCCOL|Denmark|CTX
plateauing
ESCCOL|Denmark|GEN
plateauing
ESCCOL|Denmark|MEM
logistic ns increasing
ESCCOL|Estonia|CAZ
logistic s increasing
ESCCOL|Estonia|FEP
logistic s increasing
frequency
year

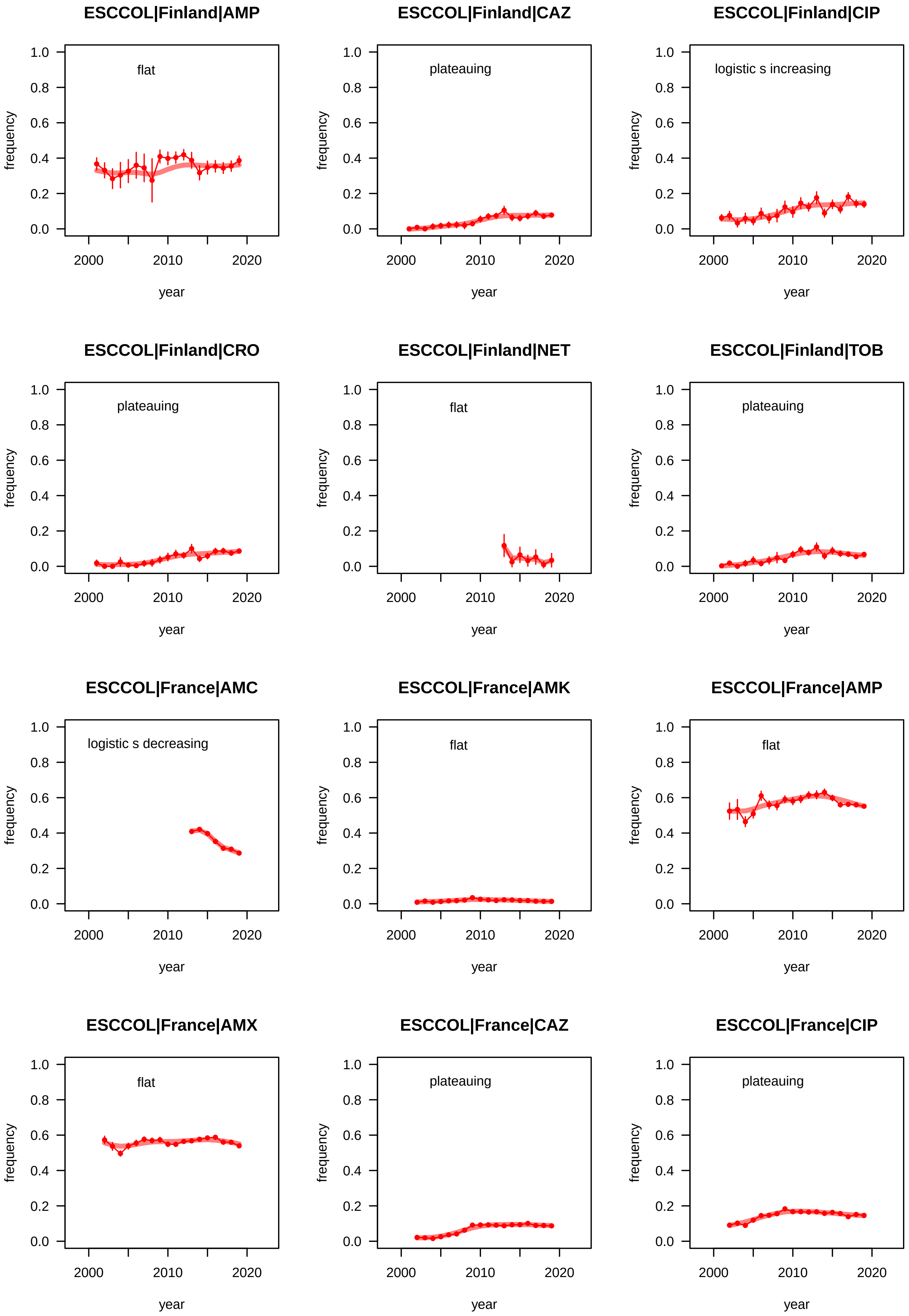
ESCCOL|Finland|AMP
flat
ESCCOL|Finland|CAZ
plateauing
ESCCOL|Finland|CIP
logistic s increasing
ESCCOL|Finland|CRO
plateauing
ESCCOL|Finland|NET
flat
ESCCOL|Finland|TOB
plateauing
ESCCOL|France|AMC
logistic s decreasing
ESCCOL|France|AMK
flat
ESCCOL|France|AMP
flat
ESCCOL|France|AMX
flat
ESCCOL|France|CAZ
plateauing
ESCCOL|France|CIP
plateauing
frequency
year
0.0
0.2
0.4
0.6
0.8
1.0
2000
2010
2020

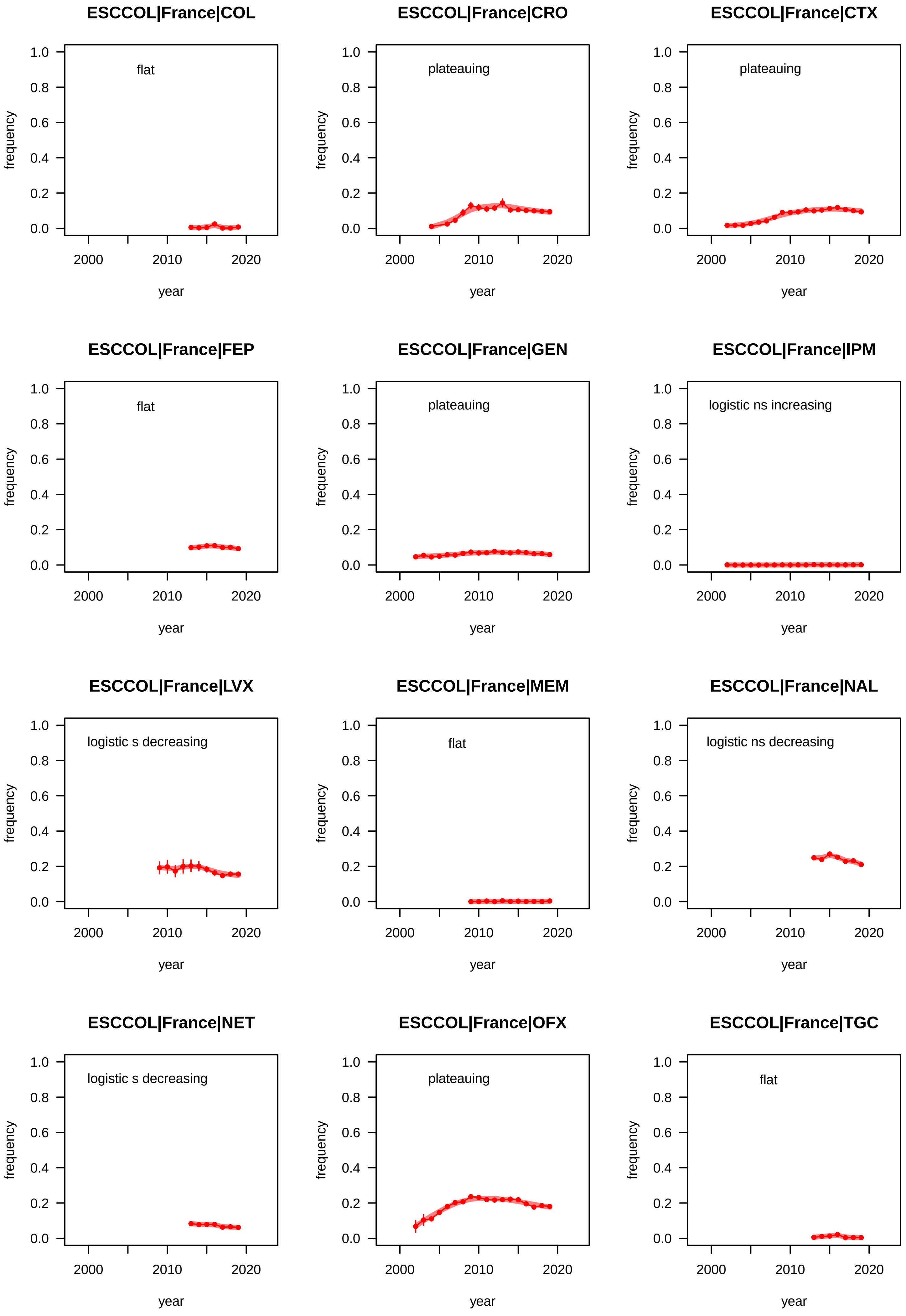
ESCCOL|France|COL
flat
ESCCOL|France|CRO
plateauing
ESCCOL|France|CTX
plateauing
ESCCOL|France|FEP
flat
ESCCOL|France|GEN
plateauing
ESCCOL|France|IPM
logistic ns increasing
ESCCOL|France|LVX
logistic s decreasing
ESCCOL|France|MEM
flat
ESCCOL|France|NAL
logistic ns decreasing
ESCCOL|France|NET
logistic s decreasing
ESCCOL|France|OFX
plateauing
ESCCOL|France|TGC
flat
frequency
0.0
0.2
0.4
0.6
0.8
1.0
year
2000
2010
2020

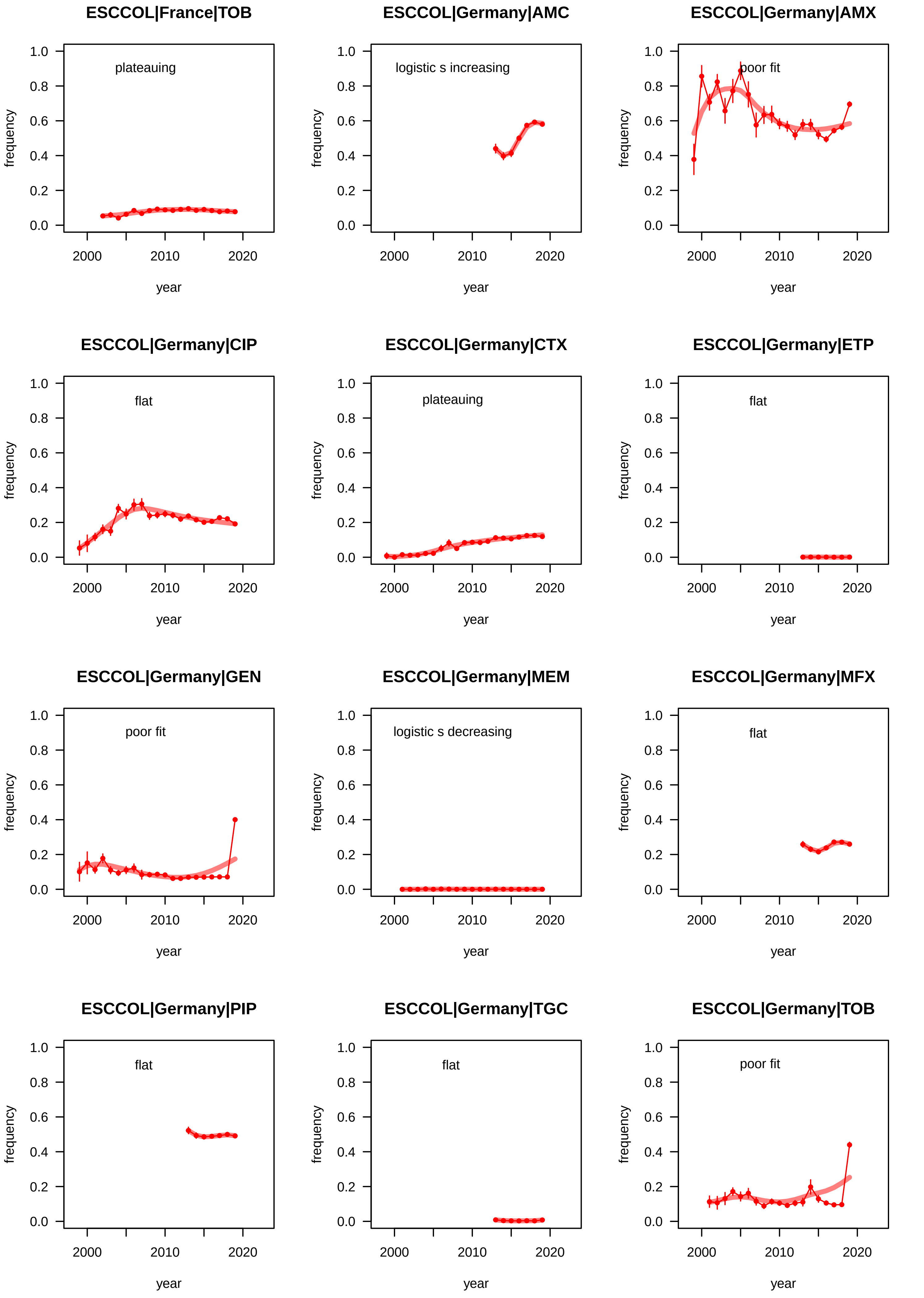
ESCCOL|France|TOB
plateauing
ESCCOL|Germany|AMC
logistic s increasing
ESCCOL|Germany|AMX
poor fit
ESCCOL|Germany|CIP
flat
ESCCOL|Germany|CTX
plateauing
ESCCOL|Germany|ETP
flat
ESCCOL|Germany|GEN
poor fit
ESCCOL|Germany|MEM
logistic s decreasing
ESCCOL|Germany|MFX
flat
ESCCOL|Germany|PIP
flat
ESCCOL|Germany|TGC
flat
ESCCOL|Germany|TOB
poor fit
frequency
year
0.0
0.2
0.4
0.6
0.8
1.0
2000
2010
2020

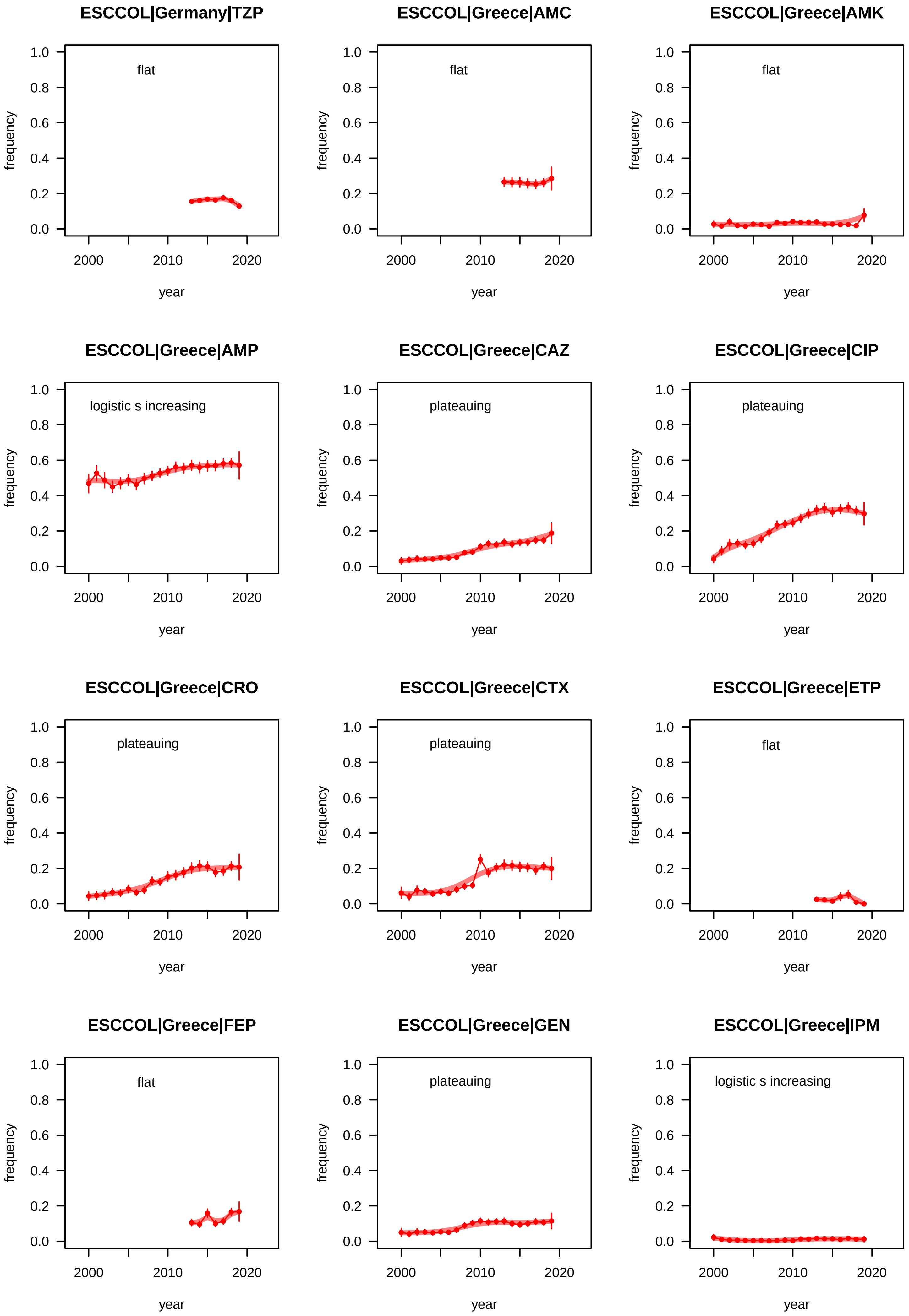
ESCCOL|Germany|TZP
flat
ESCCOL|Greece|AMC
flat
ESCCOL|Greece|AMK
flat
ESCCOL|Greece|AMP
logistic s increasing
ESCCOL|Greece|CAZ
plateauing
ESCCOL|Greece|CIP
plateauing
ESCCOL|Greece|CRO
plateauing
ESCCOL|Greece|CTX
plateauing
ESCCOL|Greece|ETP
flat
ESCCOL|Greece|FEP
flat
ESCCOL|Greece|GEN
plateauing
ESCCOL|Greece|IPM
logistic s increasing
frequency
year
0.0
0.2
0.4
0.6
0.8
1.0
2000
2010
2020

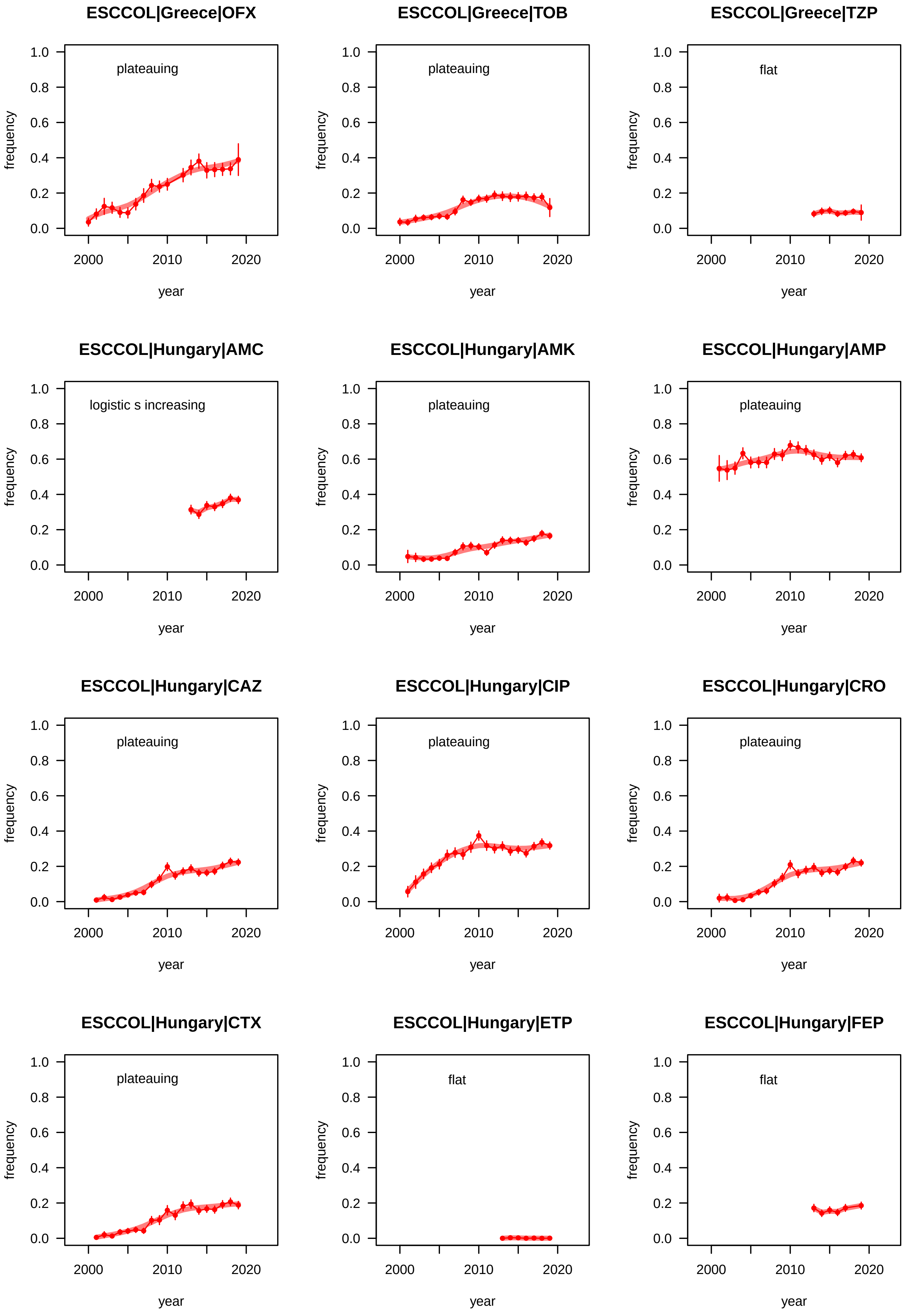
ESCCOL|Greece|OFX
plateauing
ESCCOL|Greece|TOB
plateauing
ESCCOL|Greece|TZP
flat
ESCCOL|Hungary|AMC
logistic s increasing
ESCCOL|Hungary|AMK
plateauing
ESCCOL|Hungary|AMP
plateauing
ESCCOL|Hungary|CAZ
plateauing
ESCCOL|Hungary|CIP
plateauing
ESCCOL|Hungary|CRO
plateauing
ESCCOL|Hungary|CTX
plateauing
ESCCOL|Hungary|ETP
flat
ESCCOL|Hungary|FEP
flat
frequency
year
0.0
0.2
0.4
0.6
0.8
1.0
2000
2010
2020

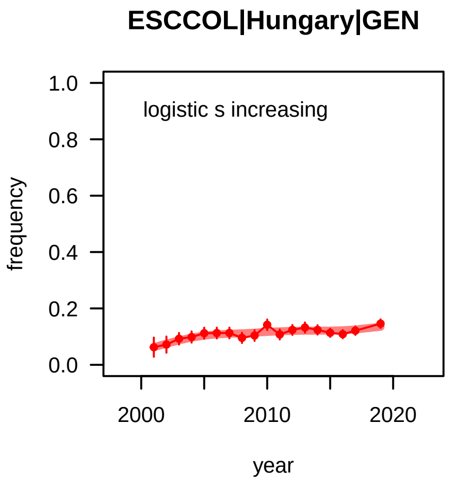
ESCCOL|Hungary|GEN
logistic s increasing
frequency
year

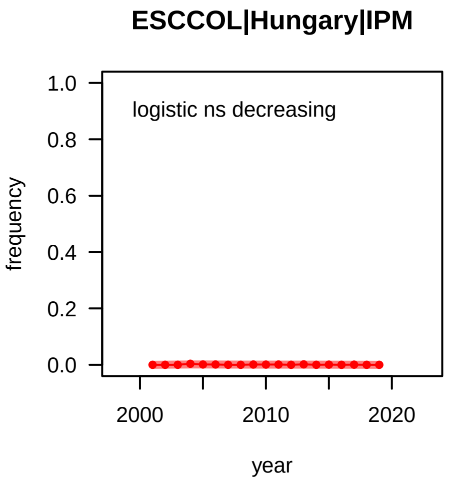
ESCCOL|Hungary|IPM
logistic ns decreasing
frequency
year

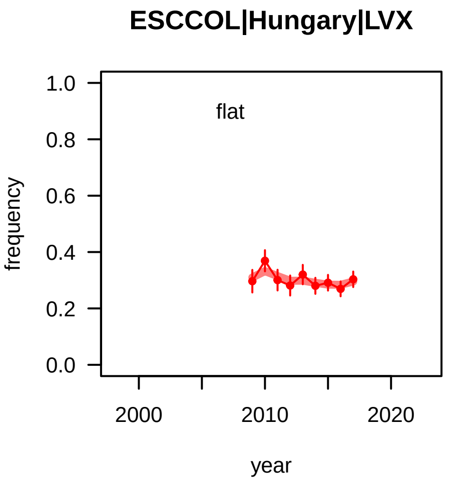
ESCCOL|Hungary|LVX
flat
frequency
year

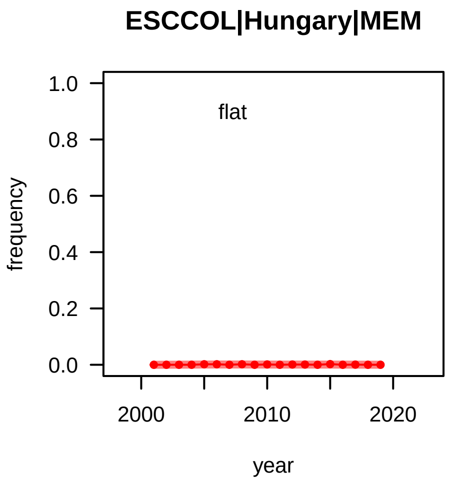
ESCCOL|Hungary|MEM
flat
frequency
year

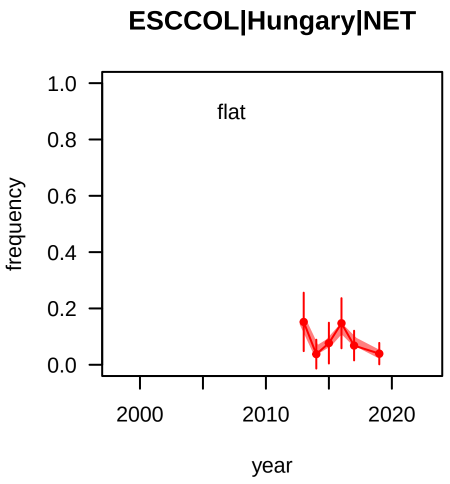
ESCCOL|Hungary|NET
flat
frequency
year

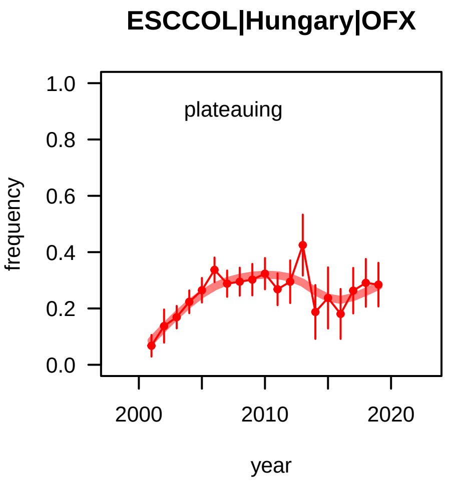
ESCCOL|Hungary|OFX
plateauing
frequency
year

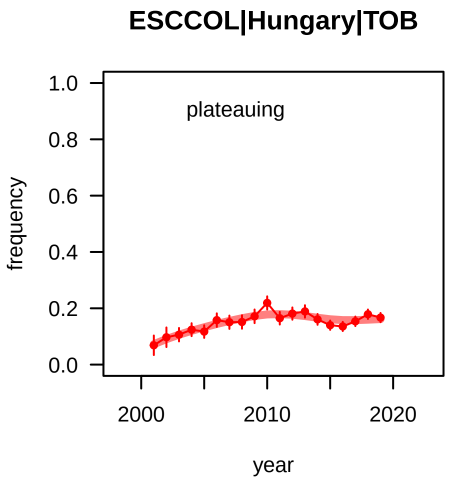
ESCCOL|Hungary|TOB
plateauing
frequency
year

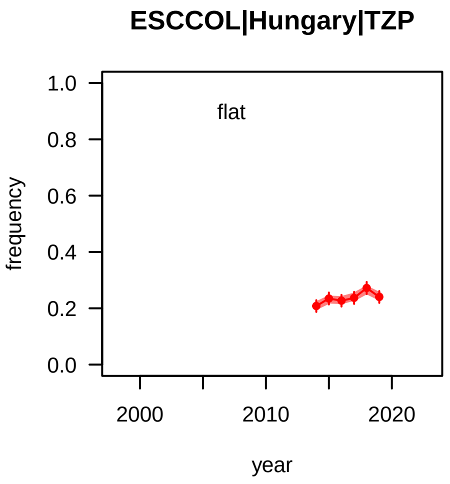
ESCCOL|Hungary|TZP
flat
frequency
year

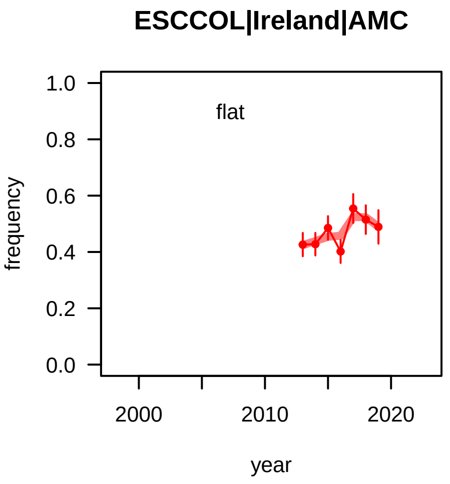
ESCCOL|Ireland|AMC
flat
frequency
year

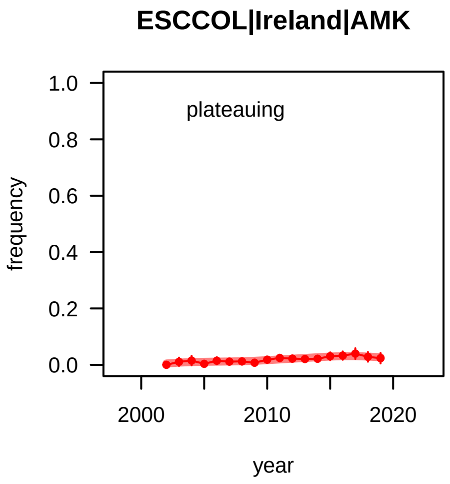
ESCCOL|Ireland|AMK
plateauing
frequency
year

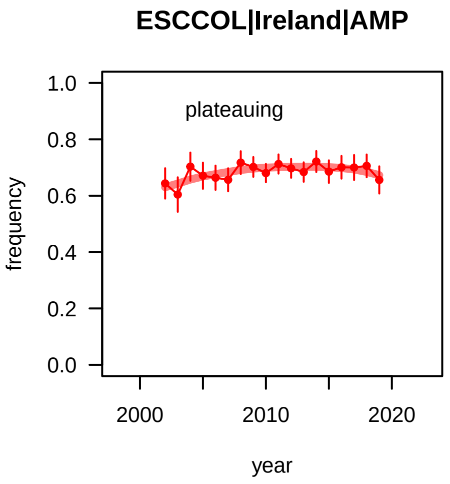
ESCCOL|Ireland|AMP
plateauing
frequency
year

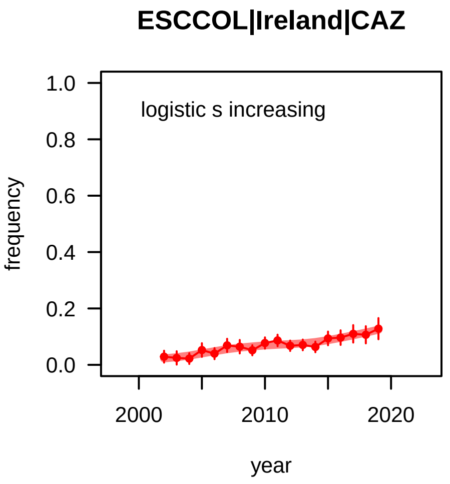
ESCCOL|Ireland|CAZ
logistic s increasing
frequency
year

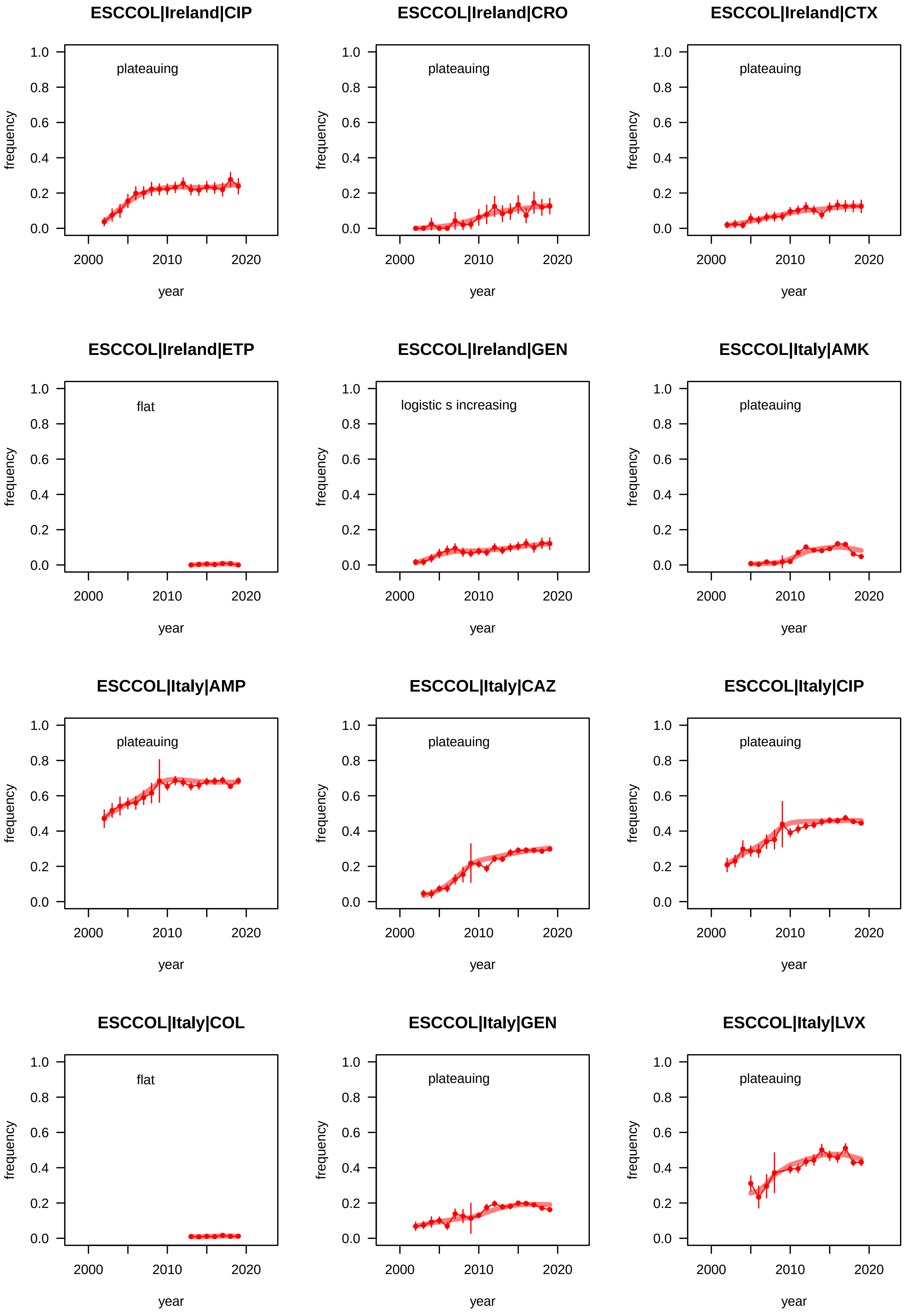
ESCCOL|Ireland|CIP
plateauing
ESCCOL|Ireland|CRO
plateauing
ESCCOL|Ireland|CTX
plateauing
ESCCOL|Ireland|ETP
flat
ESCCOL|Ireland|GEN
logistic s increasing
ESCCOL|Italy|AMK
plateauing
ESCCOL|Italy|AMP
plateauing
ESCCOL|Italy|CAZ
plateauing
ESCCOL|Italy|CIP
plateauing
ESCCOL|Italy|COL
flat
ESCCOL|Italy|GEN
plateauing
ESCCOL|Italy|LVX
plateauing
frequency
year
0.0
0.2
0.4
0.6
0.8
1.0
2000
2010
2020

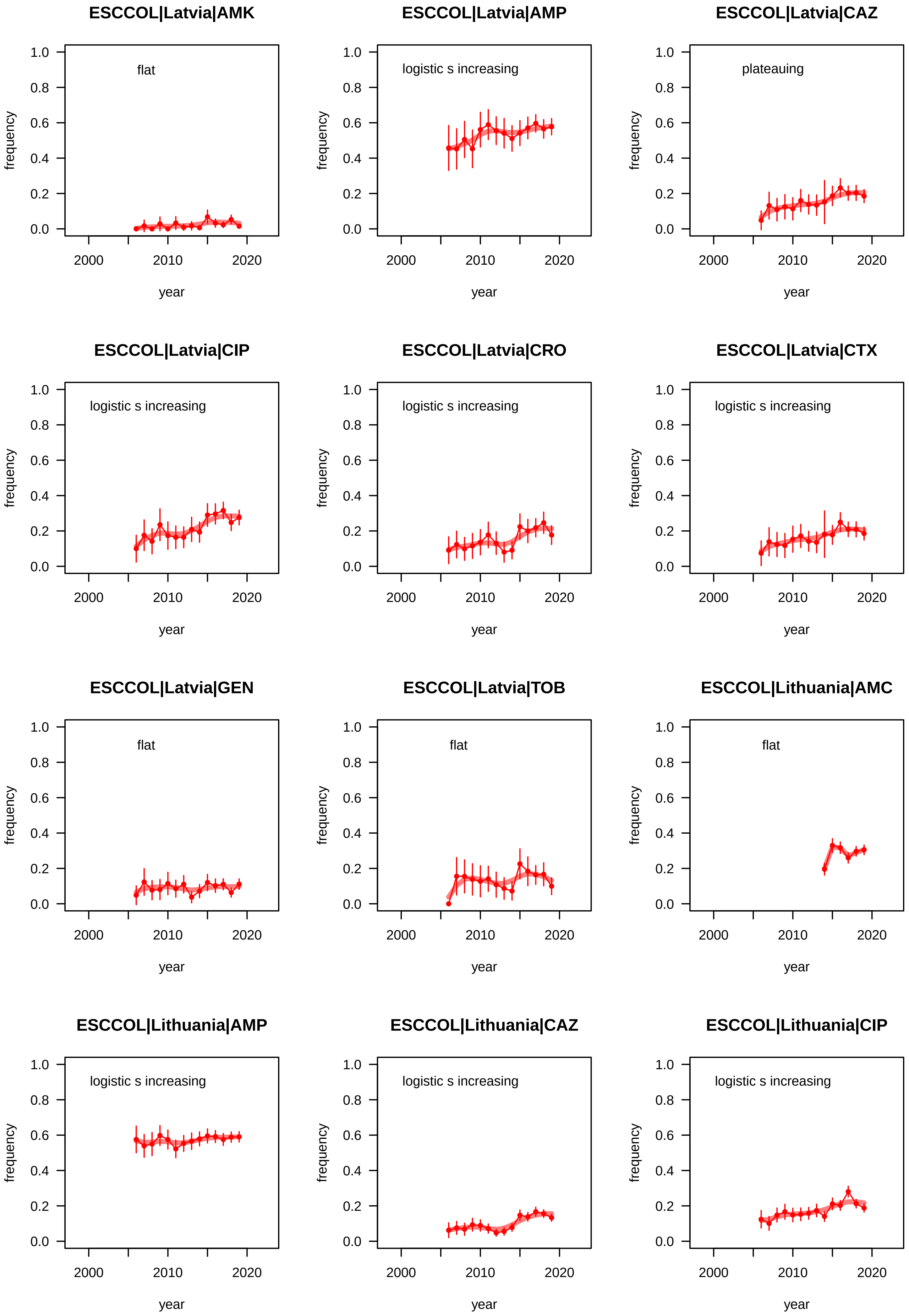
ESCCOL|Latvia|AMK
flat
ESCCOL|Latvia|AMP
logistic s increasing
ESCCOL|Latvia|CAZ
plateauing
ESCCOL|Latvia|CIP
logistic s increasing
ESCCOL|Latvia|CRO
logistic s increasing
ESCCOL|Latvia|CTX
logistic s increasing
ESCCOL|Latvia|GEN
flat
ESCCOL|Latvia|TOB
flat
ESCCOL|Lithuania|AMC
flat
ESCCOL|Lithuania|AMP
logistic s increasing
ESCCOL|Lithuania|CAZ
logistic s increasing
ESCCOL|Lithuania|CIP
logistic s increasing
frequency
year
0.0
0.2
0.4
0.6
0.8
1.0
2000
2010
2020

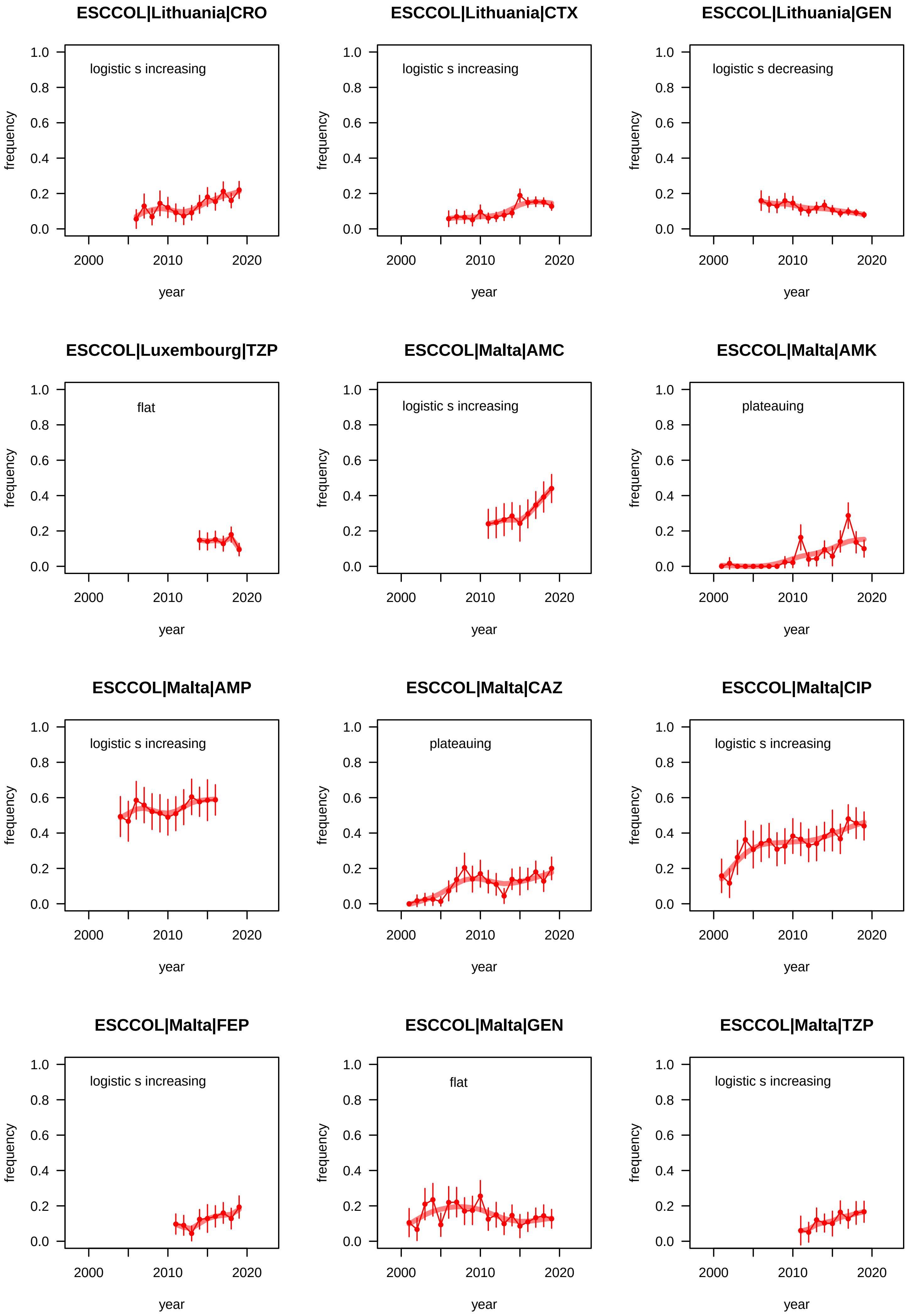
ESCCOL|Lithuania|CRO
logistic s increasing
ESCCOL|Lithuania|CTX
logistic s increasing
ESCCOL|Lithuania|GEN
logistic s decreasing
ESCCOL|Luxembourg|TZP
flat
ESCCOL|Malta|AMC
logistic s increasing
ESCCOL|Malta|AMK
plateauing
ESCCOL|Malta|AMP
logistic s increasing
ESCCOL|Malta|CAZ
plateauing
ESCCOL|Malta|CIP
logistic s increasing
ESCCOL|Malta|FEP
logistic s increasing
ESCCOL|Malta|GEN
flat
ESCCOL|Malta|TZP
logistic s increasing
frequency
year
0.0
0.2
0.4
0.6
0.8
1.0
2000
2010
2020

ESCCOL|Netherlands|AMC

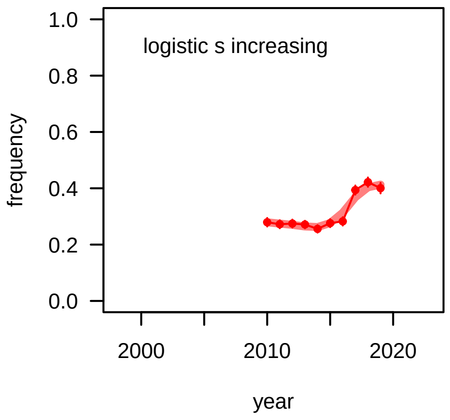

ESCCOL|Netherlands|AMK

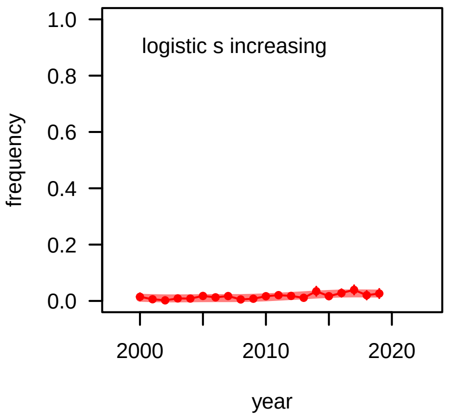

ESCCOL|Netherlands|AMX

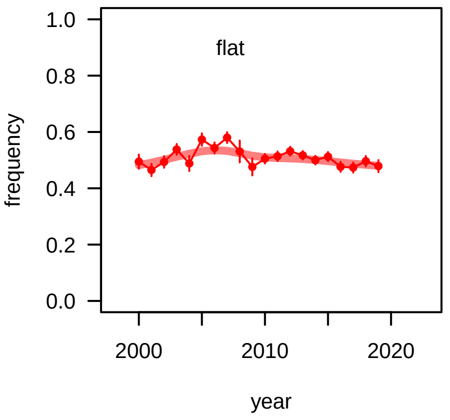

ESCCOL|Netherlands|CAZ

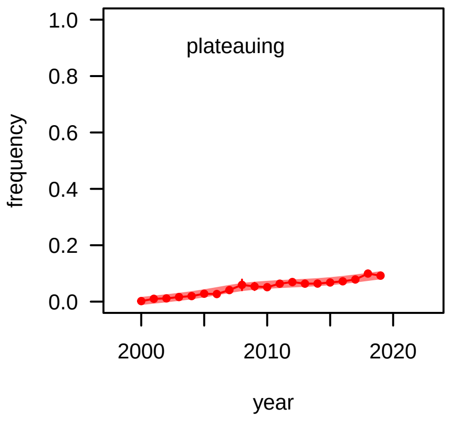

ESCCOL|Netherlands|CIP

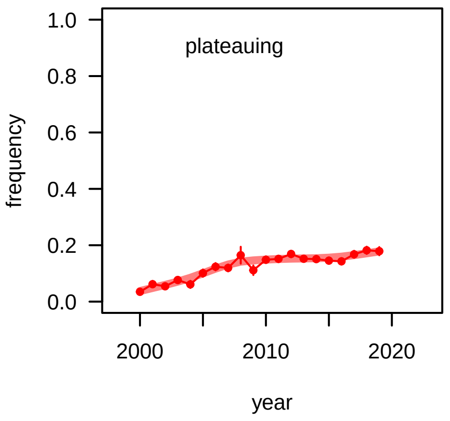

ESCCOL|Netherlands|CRO

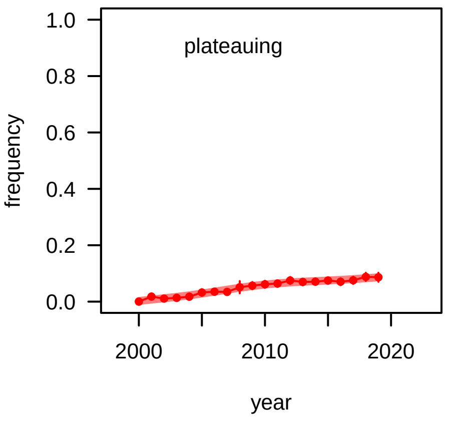

ESCCOL|Netherlands|CTX

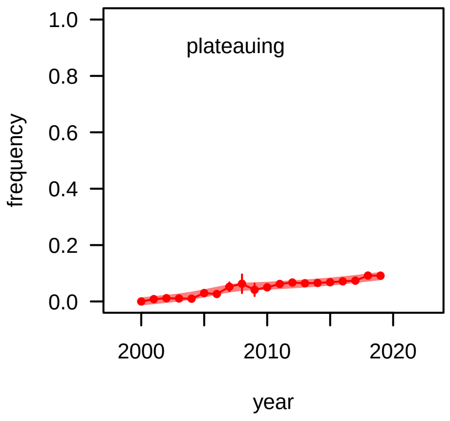

ESCCOL|Netherlands|FEP

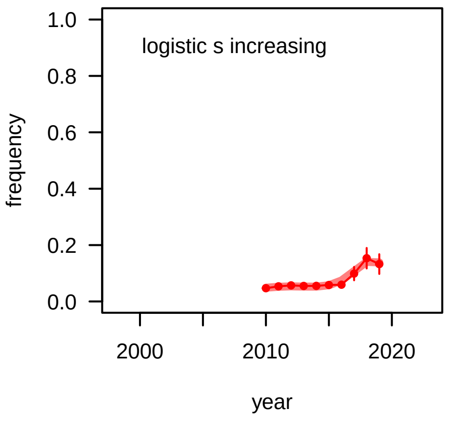

ESCCOL|Netherlands|GEN

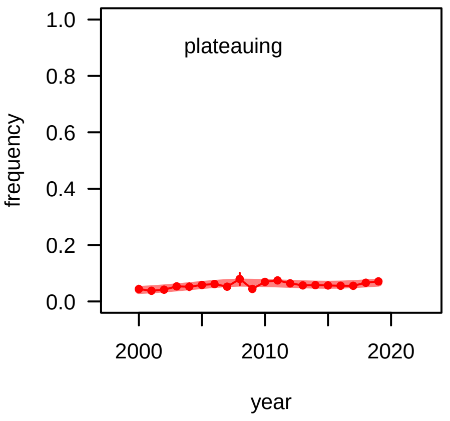

ESCCOL|Netherlands|IPM

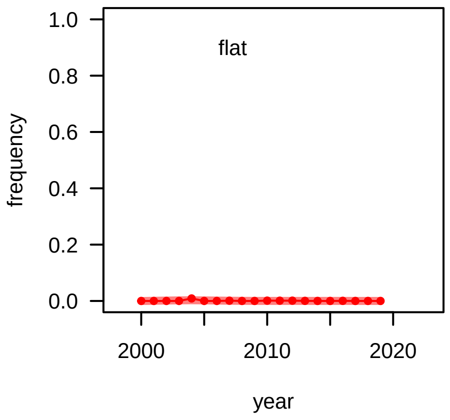

ESCCOL|Netherlands|LVX

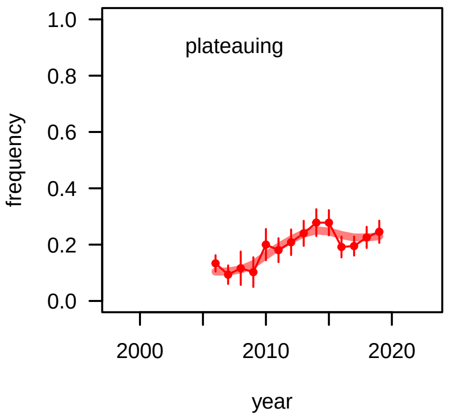

ESCCOL|Netherlands|MEM

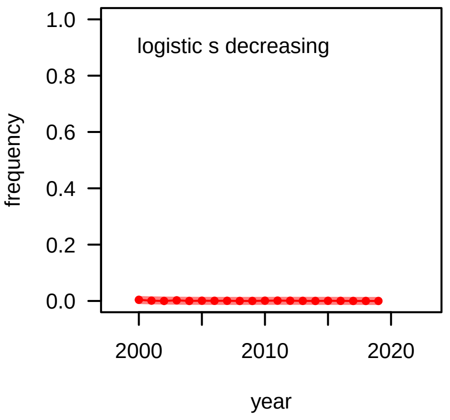

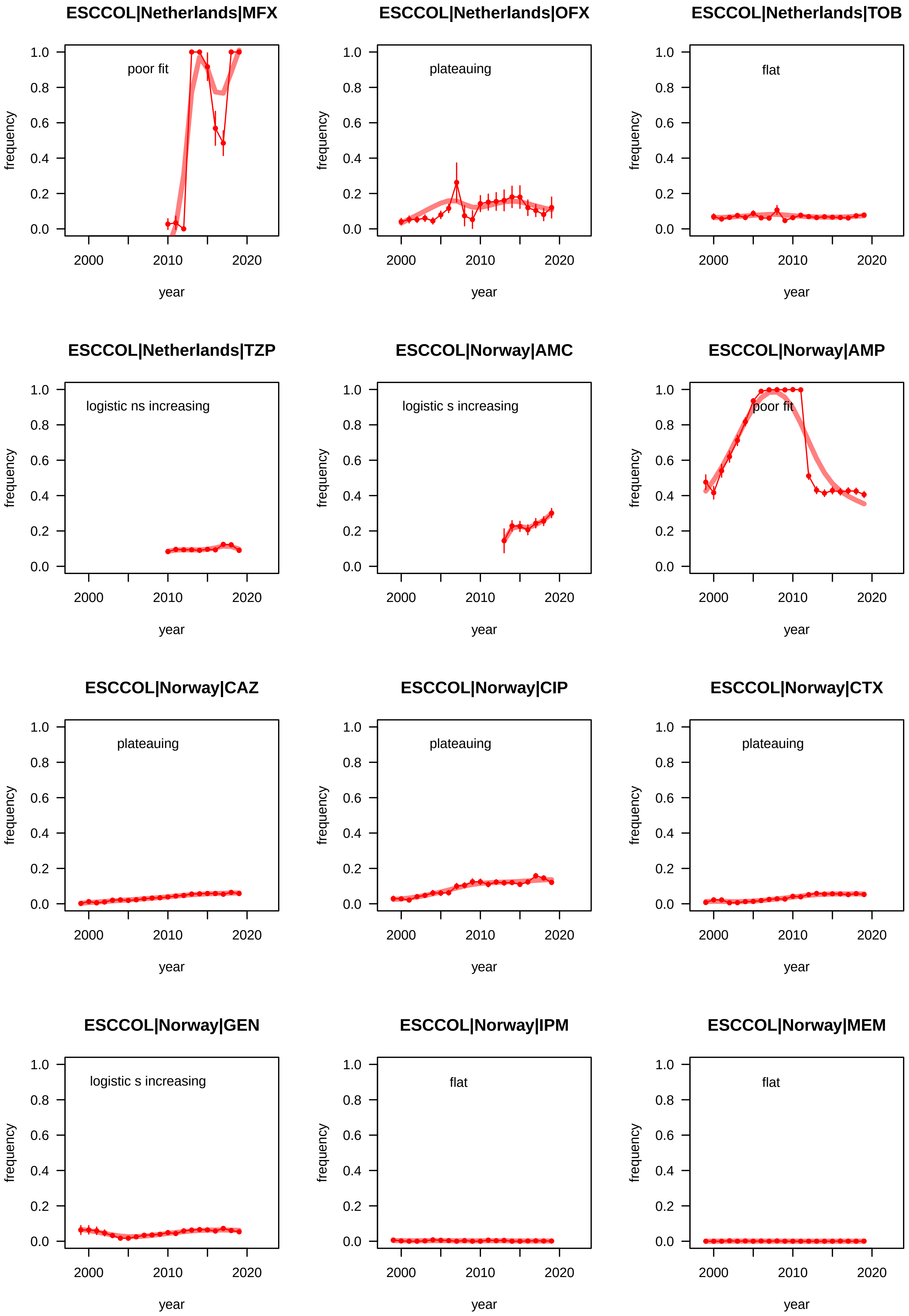

ESCCOL|Netherlands|MFX
poor fit
ESCCOL|Netherlands|OFX
plateauing
ESCCOL|Netherlands|TOB
flat
ESCCOL|Netherlands|TZP
logistic ns increasing
ESCCOL|Norway|AMC
logistic s increasing
ESCCOL|Norway|AMP
poor fit
ESCCOL|Norway|CAZ
plateauing
ESCCOL|Norway|CIP
plateauing
ESCCOL|Norway|CTX
plateauing
ESCCOL|Norway|GEN
logistic s increasing
ESCCOL|Norway|IPM
flat
ESCCOL|Norway|MEM
flat
frequency
year
0.0
0.2
0.4
0.6
0.8
1.0
2000
2010
2020

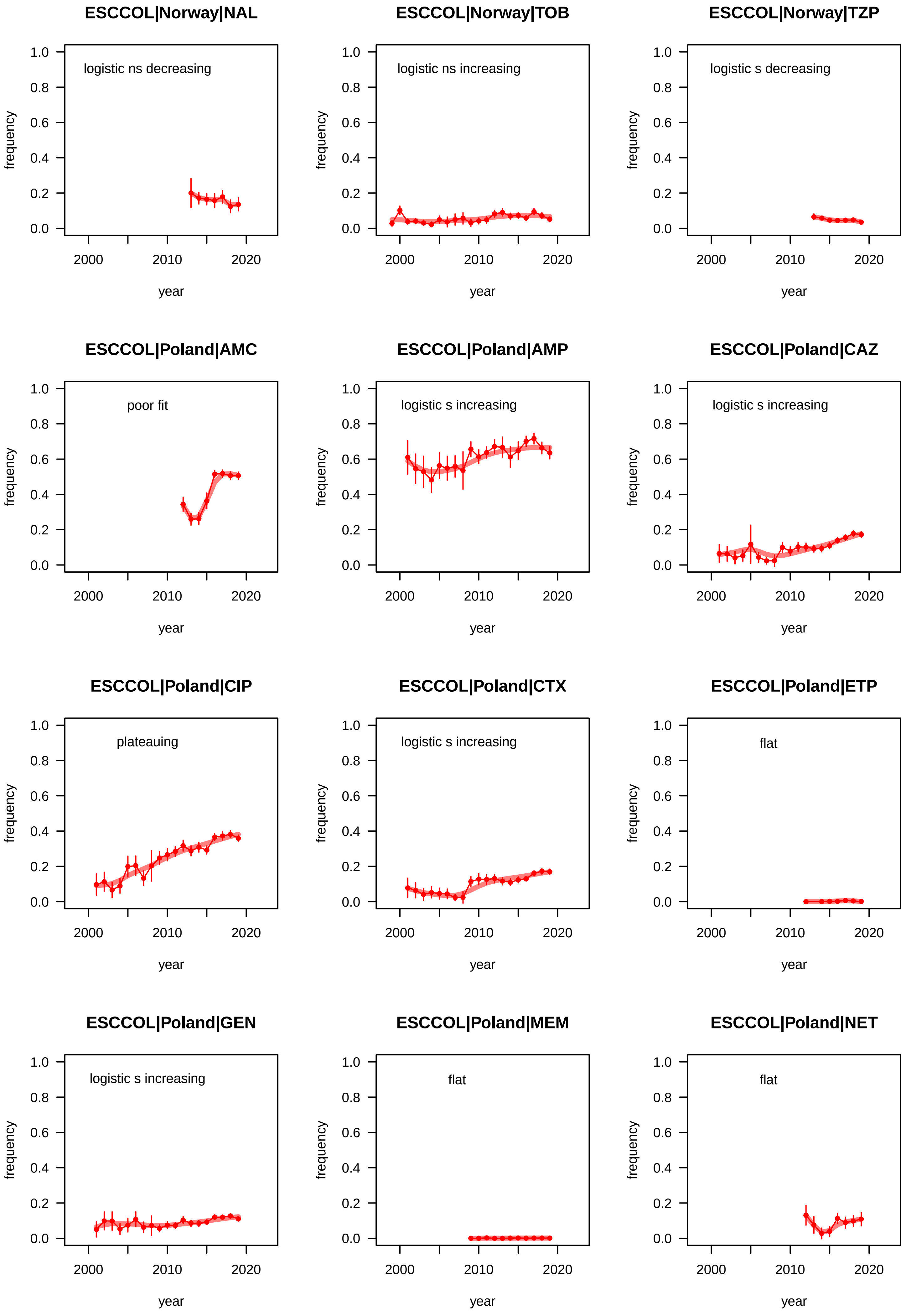
ESCCOL|Norway|NAL
logistic ns decreasing
ESCCOL|Norway|TOB
logistic ns increasing
ESCCOL|Norway|TZP
logistic s decreasing
ESCCOL|Poland|AMC
poor fit
ESCCOL|Poland|AMP
logistic s increasing
ESCCOL|Poland|CAZ
logistic s increasing
ESCCOL|Poland|CIP
plateauing
ESCCOL|Poland|CTX
logistic s increasing
ESCCOL|Poland|ETP
flat
ESCCOL|Poland|GEN
logistic s increasing
ESCCOL|Poland|MEM
flat
ESCCOL|Poland|NET
flat
frequency
year
0.0
0.2
0.4
0.6
0.8
1.0
2000
2010
2020

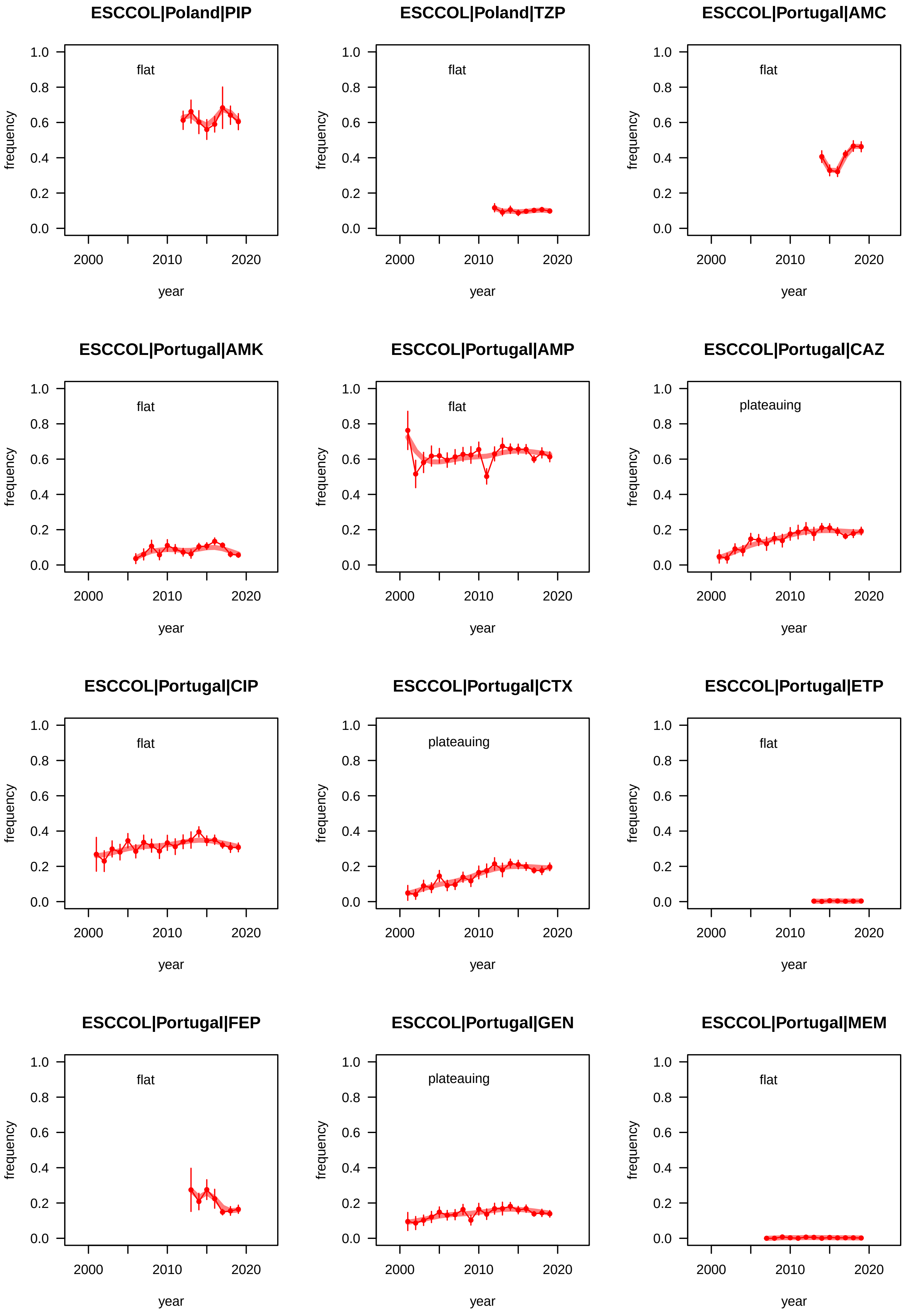
ESCCOL|Poland|PIP
flat
ESCCOL|Poland|TZP
flat
ESCCOL|Portugal|AMC
flat
ESCCOL|Portugal|AMK
flat
ESCCOL|Portugal|AMP
flat
ESCCOL|Portugal|CAZ
plateauing
ESCCOL|Portugal|CIP
flat
ESCCOL|Portugal|CTX
plateauing
ESCCOL|Portugal|ETP
flat
ESCCOL|Portugal|FEP
flat
ESCCOL|Portugal|GEN
plateauing
ESCCOL|Portugal|MEM
flat
frequency
year
0.0
0.2
0.4
0.6
0.8
1.0
2000
2010
2020

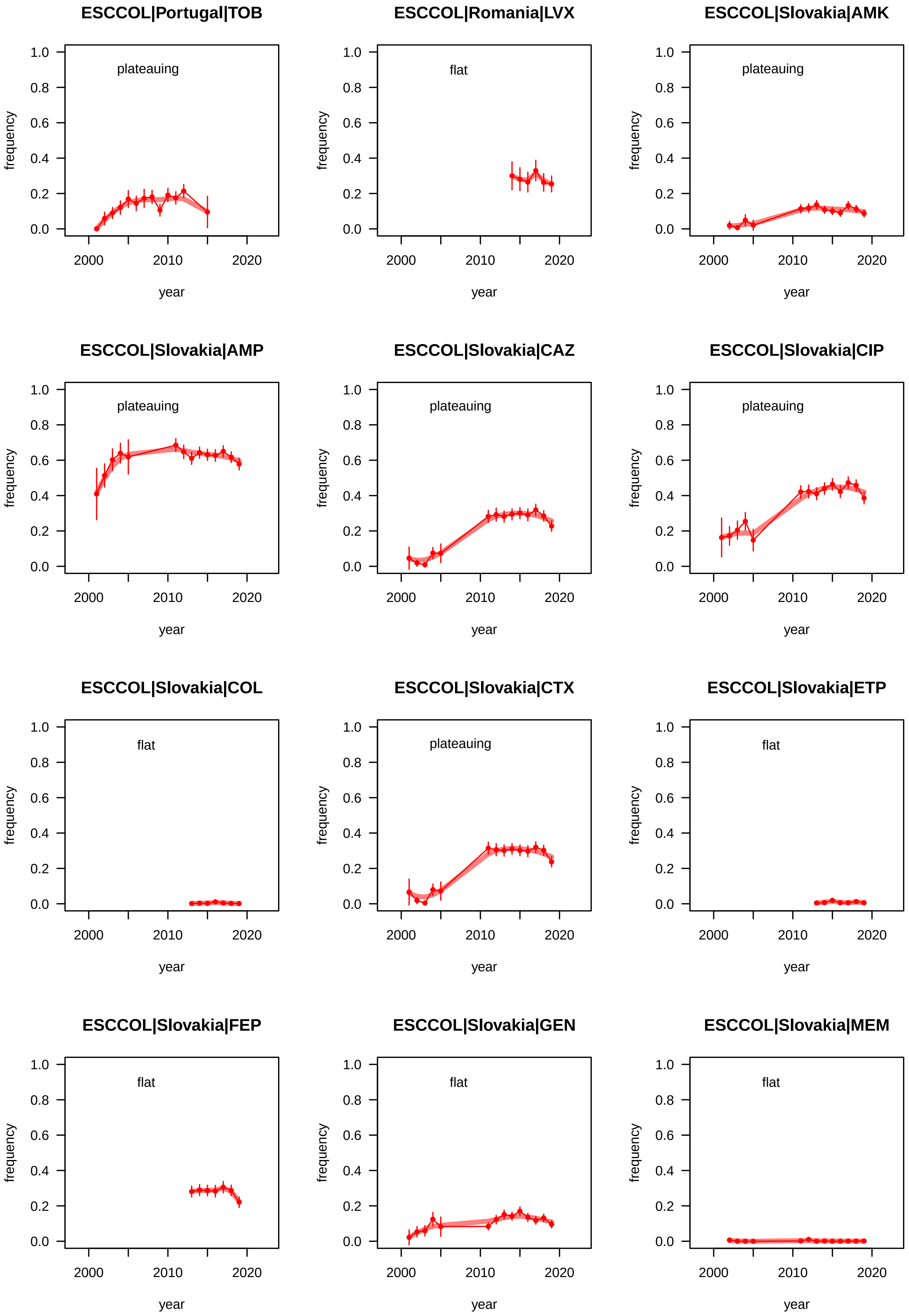
ESCCOL|Portugal|TOB
plateauing
ESCCOL|Romania|LVX
flat
ESCCOL|Slovakia|AMK
plateauing
ESCCOL|Slovakia|AMP
plateauing
ESCCOL|Slovakia|CAZ
plateauing
ESCCOL|Slovakia|CIP
plateauing
ESCCOL|Slovakia|COL
flat
ESCCOL|Slovakia|CTX
plateauing
ESCCOL|Slovakia|ETP
flat
ESCCOL|Slovakia|FEP
flat
ESCCOL|Slovakia|GEN
flat
ESCCOL|Slovakia|MEM
flat
frequency
year
0.0
0.2
0.4
0.6
0.8
1.0
2000
2010
2020

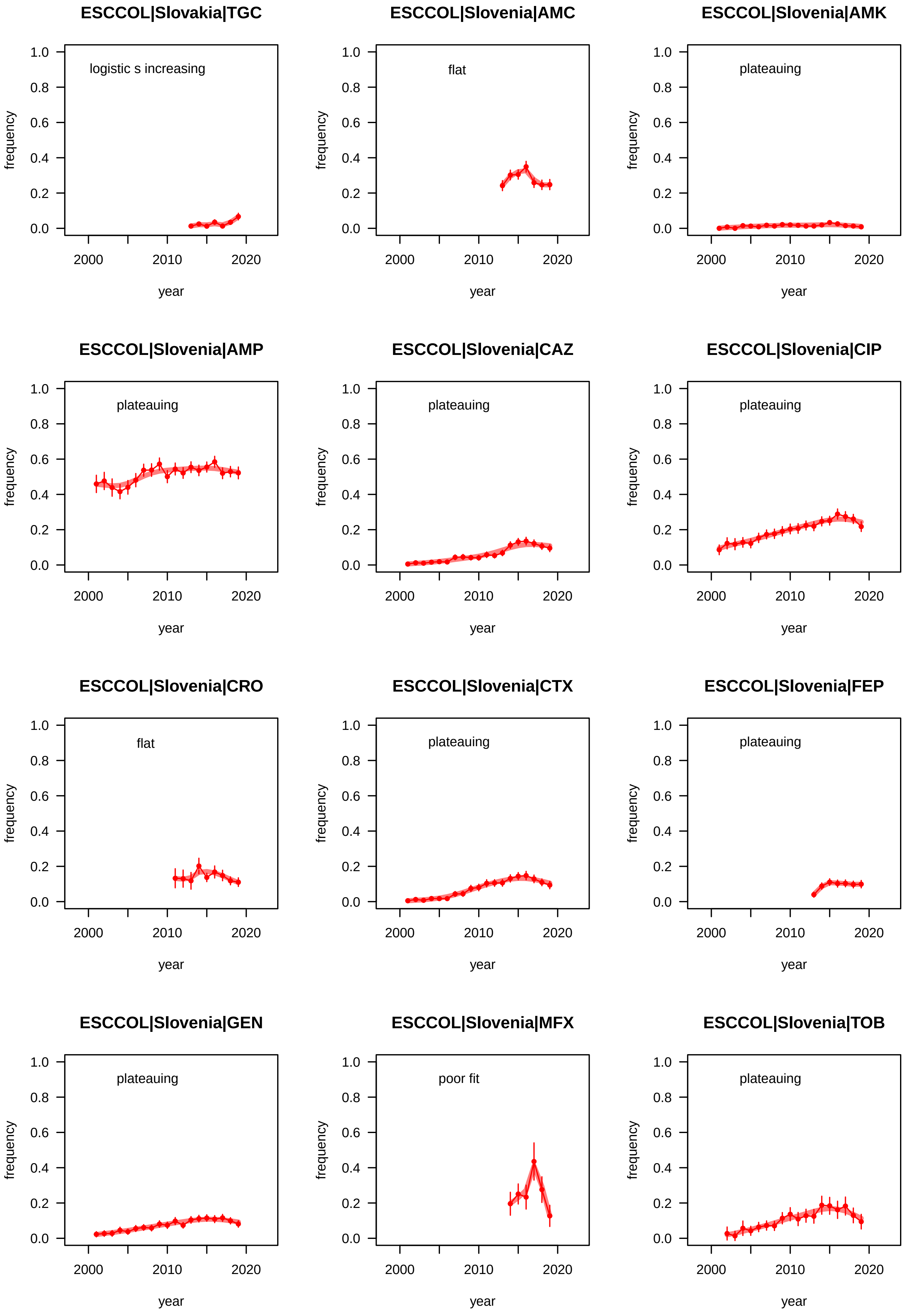
ESCCOL|Slovakia|TGC
logistic s increasing
ESCCOL|Slovenia|AMC
flat
ESCCOL|Slovenia|AMK
plateauing
ESCCOL|Slovenia|AMP
plateauing
ESCCOL|Slovenia|CAZ
plateauing
ESCCOL|Slovenia|CIP
plateauing
ESCCOL|Slovenia|CRO
flat
ESCCOL|Slovenia|CTX
plateauing
ESCCOL|Slovenia|FEP
plateauing
ESCCOL|Slovenia|GEN
plateauing
ESCCOL|Slovenia|MFX
poor fit
ESCCOL|Slovenia|TOB
plateauing
frequency
year
0.0
0.2
0.4
0.6
0.8
1.0
2000
2010
2020

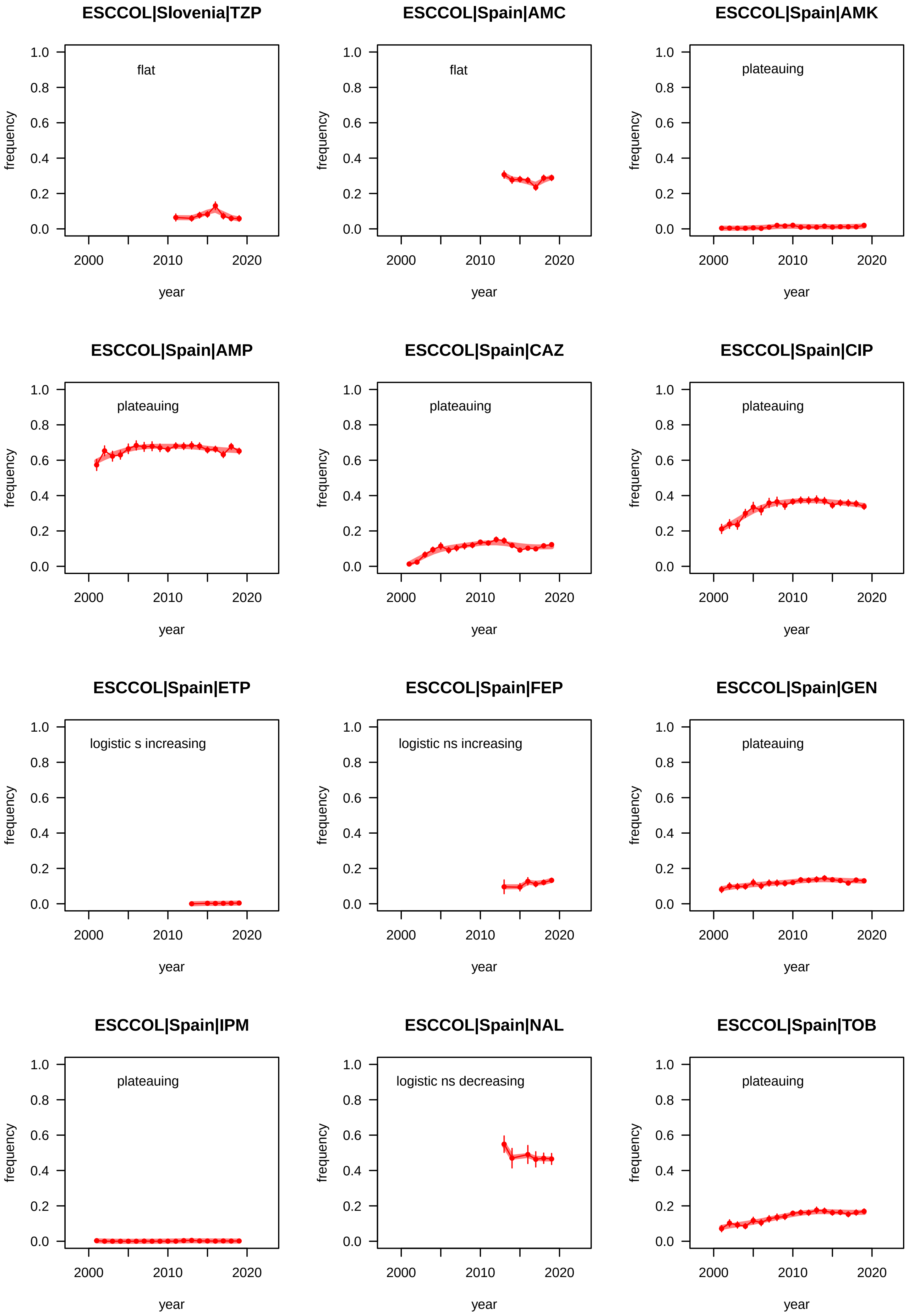
ESCCOL|Slovenia|TZP
flat
ESCCOL|Spain|AMC
flat
ESCCOL|Spain|AMK
plateauing
ESCCOL|Spain|AMP
plateauing
ESCCOL|Spain|CAZ
plateauing
ESCCOL|Spain|CIP
plateauing
ESCCOL|Spain|ETP
logistic s increasing
ESCCOL|Spain|FEP
logistic ns increasing
ESCCOL|Spain|GEN
plateauing
ESCCOL|Spain|IPM
plateauing
ESCCOL|Spain|NAL
logistic ns decreasing
ESCCOL|Spain|TOB
plateauing
frequency
year
0.0
0.2
0.4
0.6
0.8
1.0
2000
2010
2020

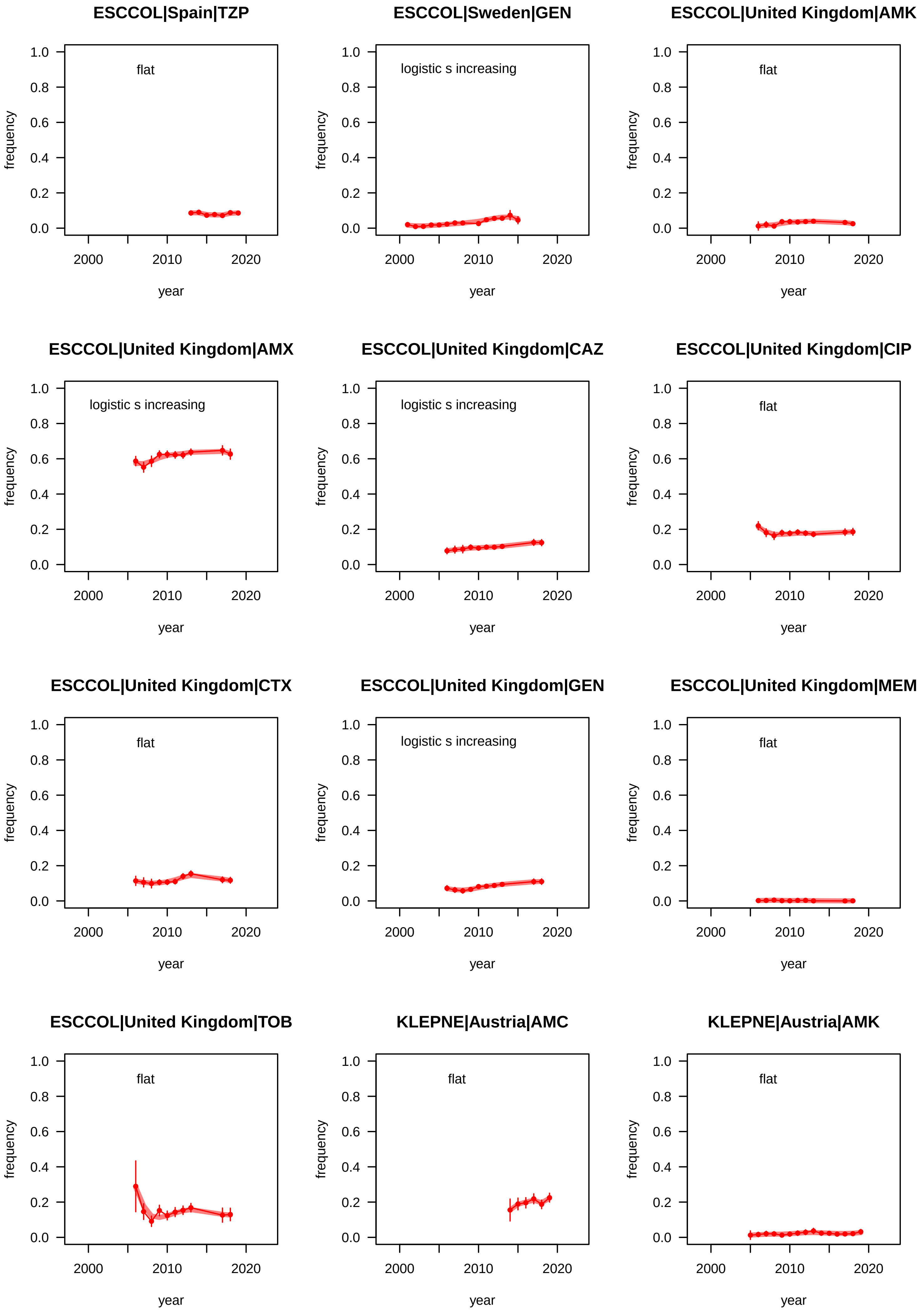
ESCCOL|Spain|TZP
flat
ESCCOL|Sweden|GEN
logistic s increasing
ESCCOL|United Kingdom|AMK
flat
ESCCOL|United Kingdom|AMX
logistic s increasing
ESCCOL|United Kingdom|CAZ
logistic s increasing
ESCCOL|United Kingdom|CIP
flat
ESCCOL|United Kingdom|CTX
flat
ESCCOL|United Kingdom|GEN
logistic s increasing
ESCCOL|United Kingdom|MEM
flat
ESCCOL|United Kingdom|TOB
flat
KLEPNE|Austria|AMC
flat
KLEPNE|Austria|AMK
flat
frequency
year
0.0
0.2
0.4
0.6
0.8
1.0
2000
2010
2020

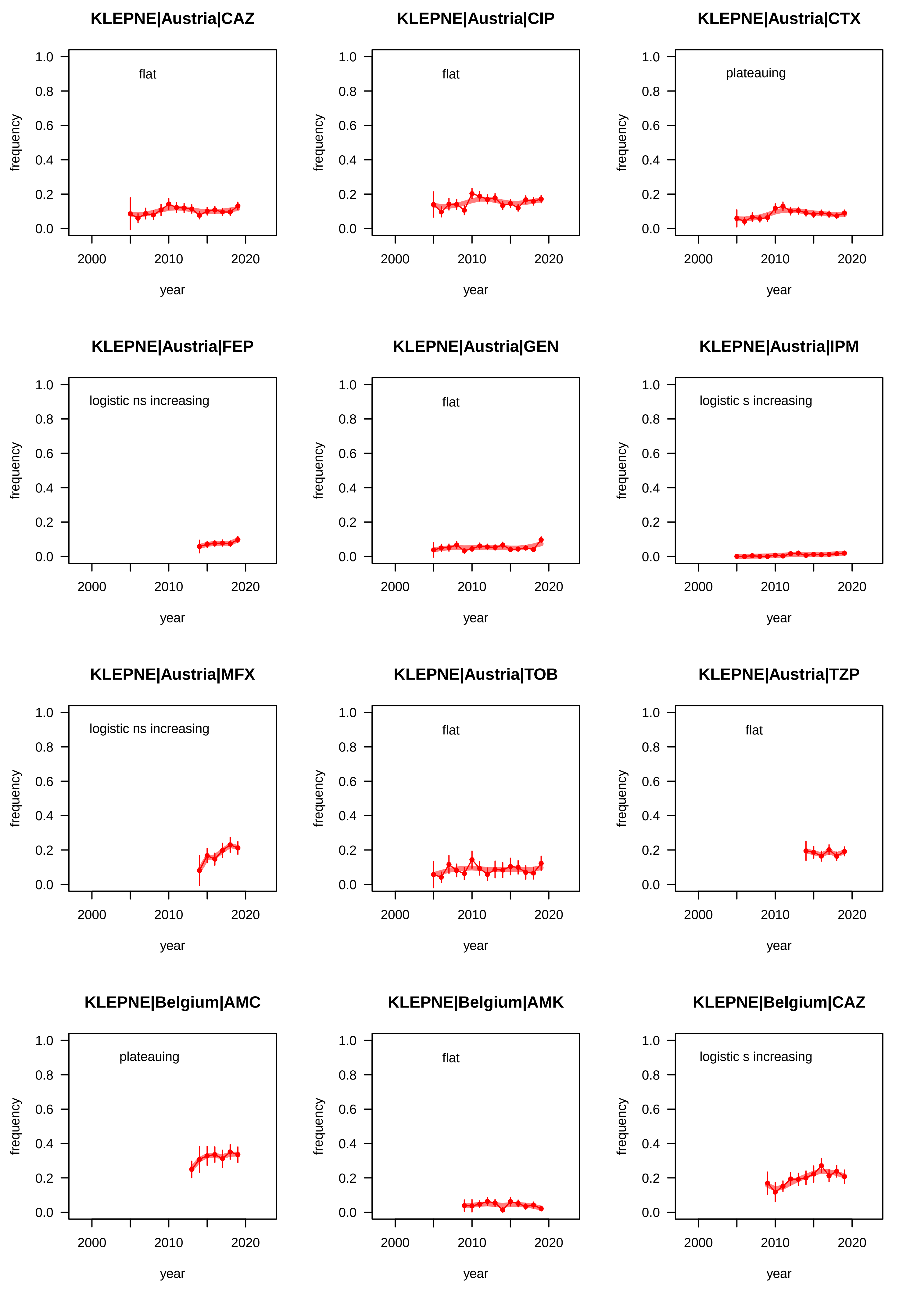
KLEPNE|Austria|CAZ
flat
KLEPNE|Austria|CIP
flat
KLEPNE|Austria|CTX
plateauing
KLEPNE|Austria|FEP
logistic ns increasing
KLEPNE|Austria|GEN
flat
KLEPNE|Austria|IPM
logistic s increasing
KLEPNE|Austria|MFX
logistic ns increasing
KLEPNE|Austria|TOB
flat
KLEPNE|Austria|TZP
flat
KLEPNE|Belgium|AMC
plateauing
KLEPNE|Belgium|AMK
flat
KLEPNE|Belgium|CAZ
logistic s increasing
frequency
year
1.0
0.8
0.6
0.4
0.2
0.0
2000
2010
2020

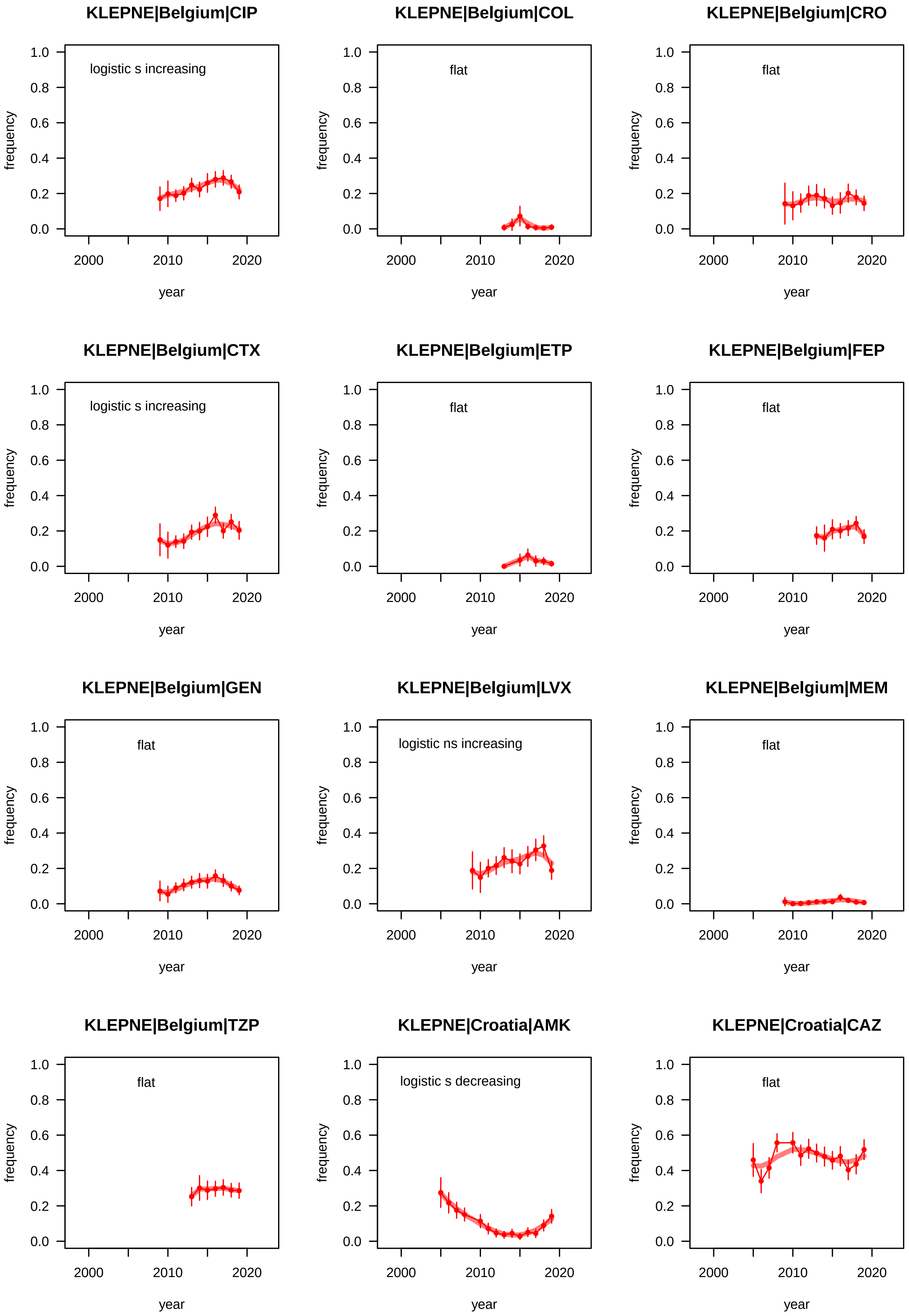
KLEPNE|Belgium|CIP
logistic s increasing
KLEPNE|Belgium|COL
flat
KLEPNE|Belgium|CRO
flat
KLEPNE|Belgium|CTX
logistic s increasing
KLEPNE|Belgium|ETP
flat
KLEPNE|Belgium|FEP
flat
KLEPNE|Belgium|GEN
flat
KLEPNE|Belgium|LVX
logistic ns increasing
KLEPNE|Belgium|MEM
flat
KLEPNE|Belgium|TZP
flat
KLEPNE|Croatia|AMK
logistic s decreasing
KLEPNE|Croatia|CAZ
flat
frequency
0.0
0.2
0.4
0.6
0.8
1.0
year
2000
2010
2020

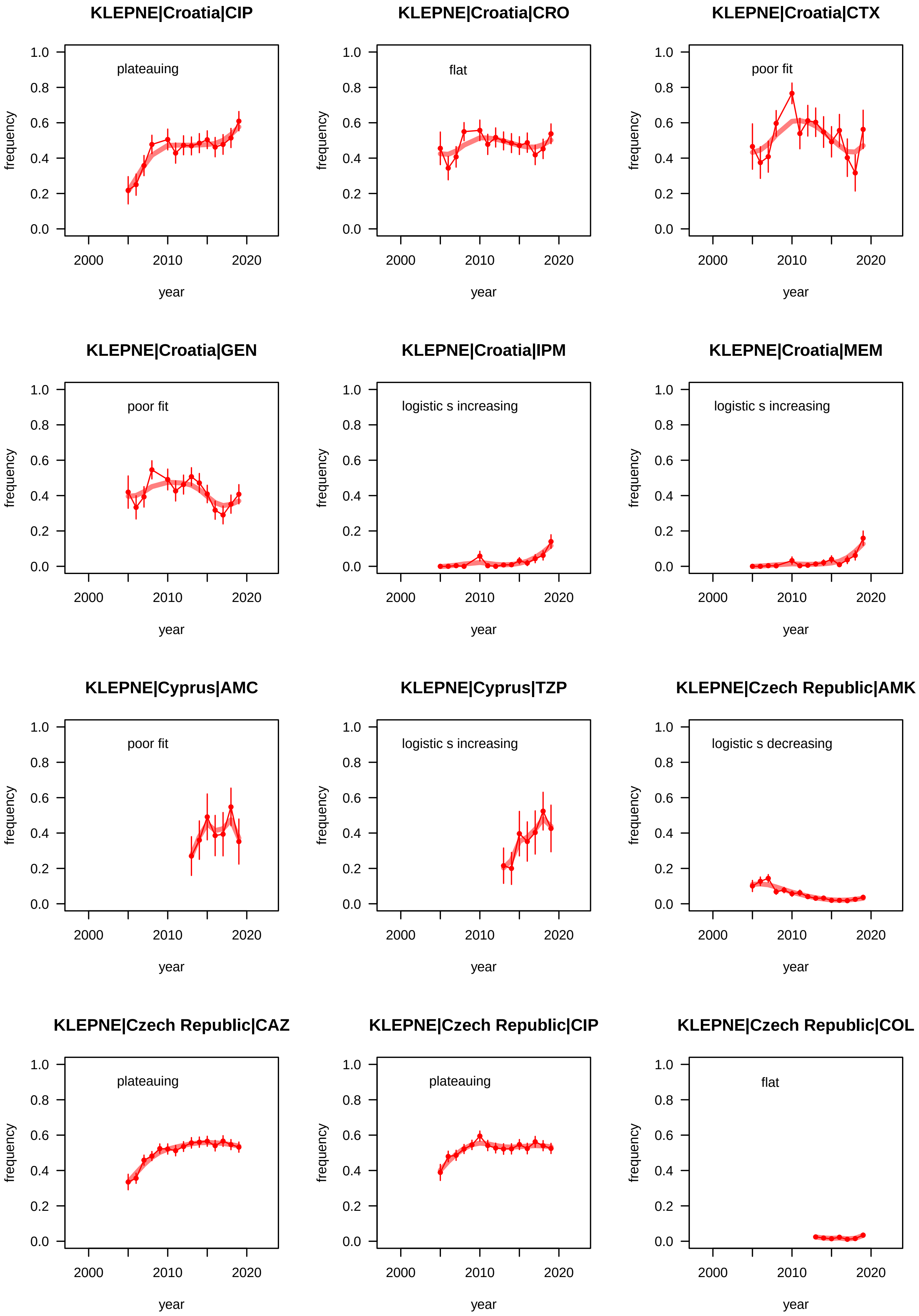
KLEPNE|Croatia|CIP
plateauing
KLEPNE|Croatia|CRO
flat
KLEPNE|Croatia|CTX
poor fit
KLEPNE|Croatia|GEN
poor fit
KLEPNE|Croatia|IPM
logistic s increasing
KLEPNE|Croatia|MEM
logistic s increasing
KLEPNE|Cyprus|AMC
poor fit
KLEPNE|Cyprus|TZP
logistic s increasing
KLEPNE|Czech Republic|AMK
logistic s decreasing
KLEPNE|Czech Republic|CAZ
plateauing
KLEPNE|Czech Republic|CIP
plateauing
KLEPNE|Czech Republic|COL
flat
frequency
year
0.0
0.2
0.4
0.6
0.8
1.0
2000
2010
2020

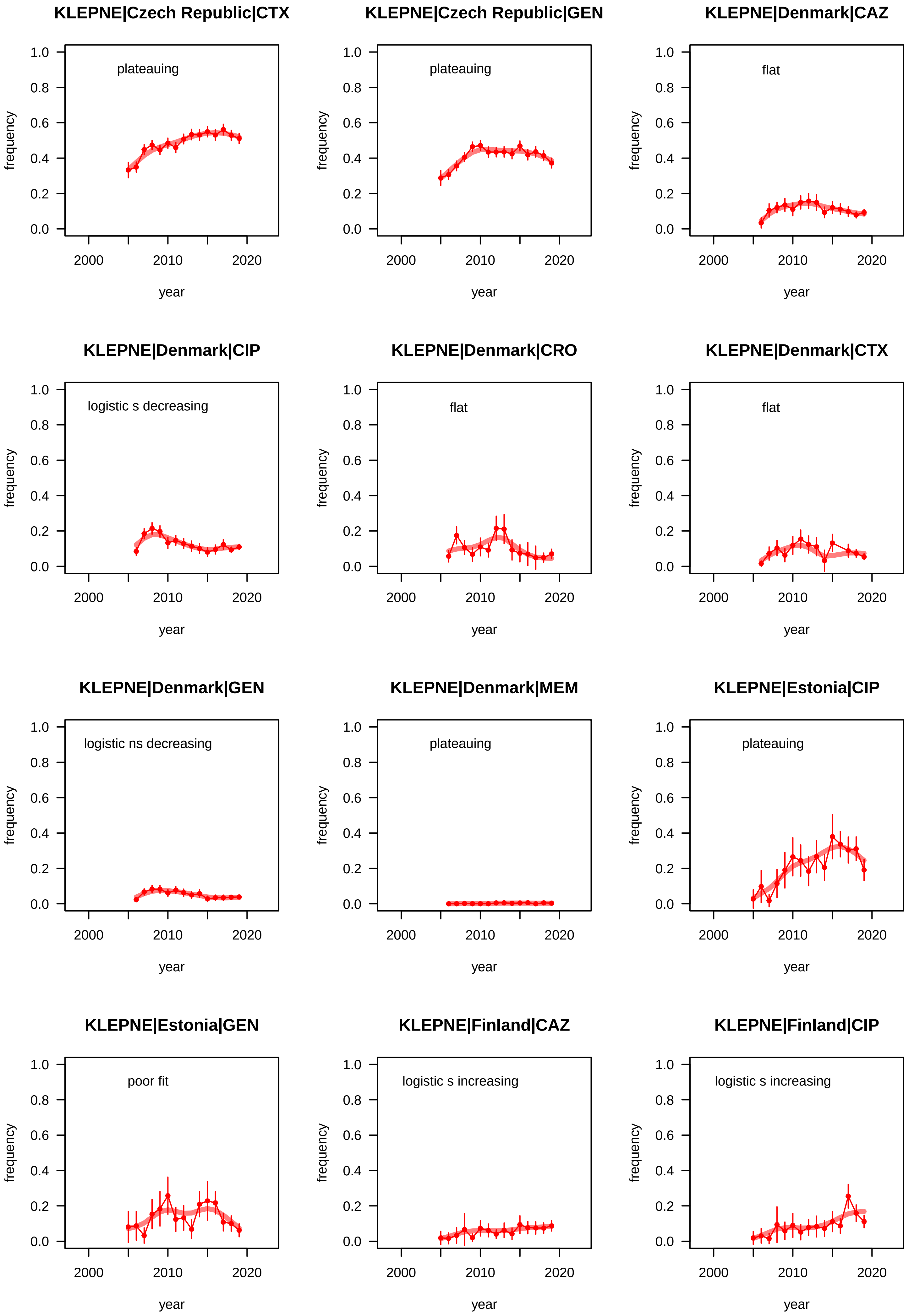

KLEPNE|Czech Republic|CTX
plateauing
KLEPNE|Czech Republic|GEN
plateauing
KLEPNE|Denmark|CAZ
flat
KLEPNE|Denmark|CIP
logistic s decreasing
KLEPNE|Denmark|CRO
flat
KLEPNE|Denmark|CTX
flat
KLEPNE|Denmark|GEN
logistic ns decreasing
KLEPNE|Denmark|MEM
plateauing
KLEPNE|Estonia|CIP
plateauing
KLEPNE|Estonia|GEN
poor fit
KLEPNE|Finland|CAZ
logistic s increasing
KLEPNE|Finland|CIP
logistic s increasing
frequency
0.0
0.2
0.4
0.6
0.8
1.0
year
2000
2010
2020

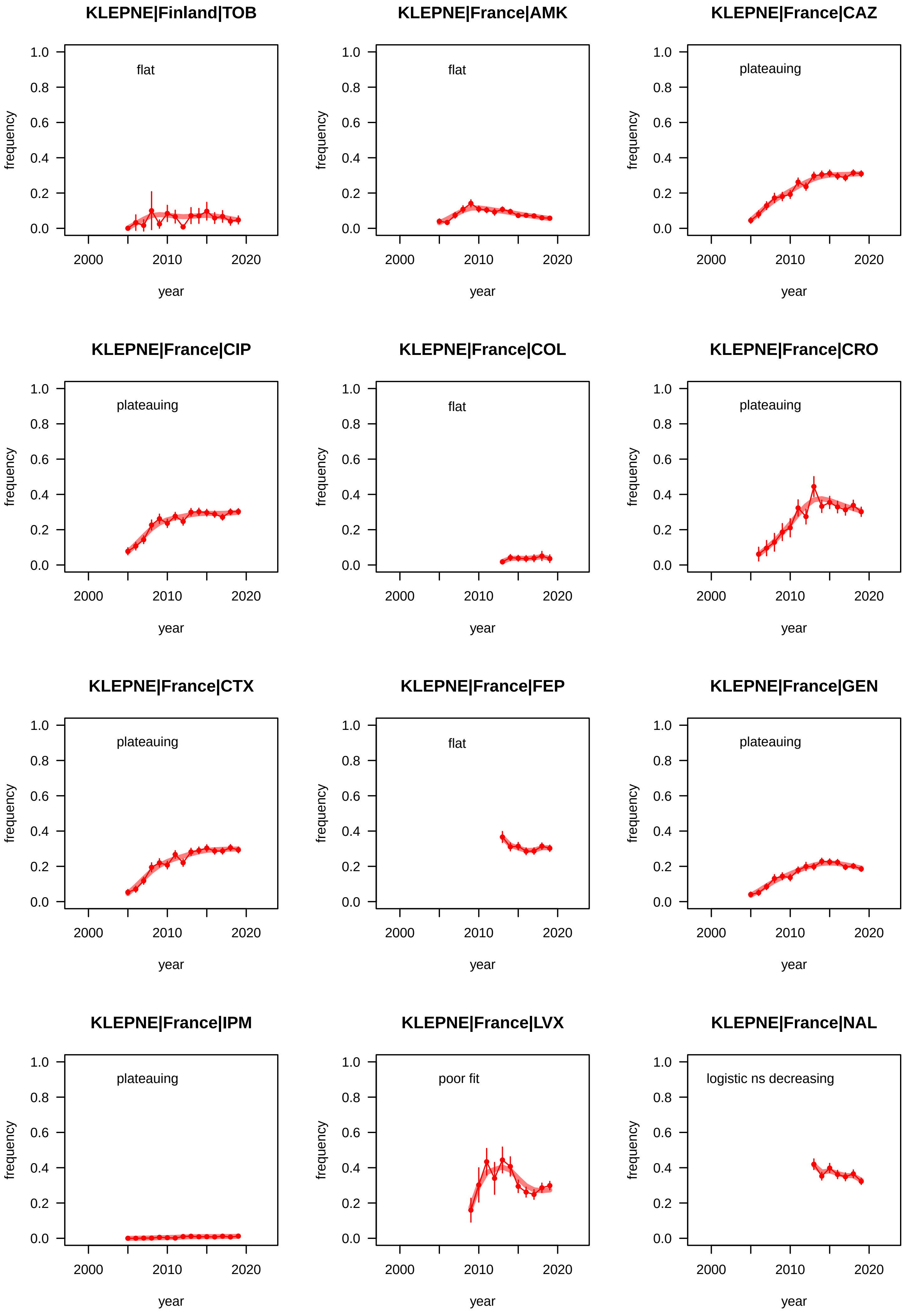
KLEPNE|Finland|TOB
flat
KLEPNE|France|AMK
flat
KLEPNE|France|CAZ
plateauing
KLEPNE|France|CIP
plateauing
KLEPNE|France|COL
flat
KLEPNE|France|CRO
plateauing
KLEPNE|France|CTX
plateauing
KLEPNE|France|FEP
flat
KLEPNE|France|GEN
plateauing
KLEPNE|France|IPM
plateauing
KLEPNE|France|LVX
poor fit
KLEPNE|France|NAL
logistic ns decreasing
frequency
year
0.0
0.2
0.4
0.6
0.8
1.0
2000
2010
2020

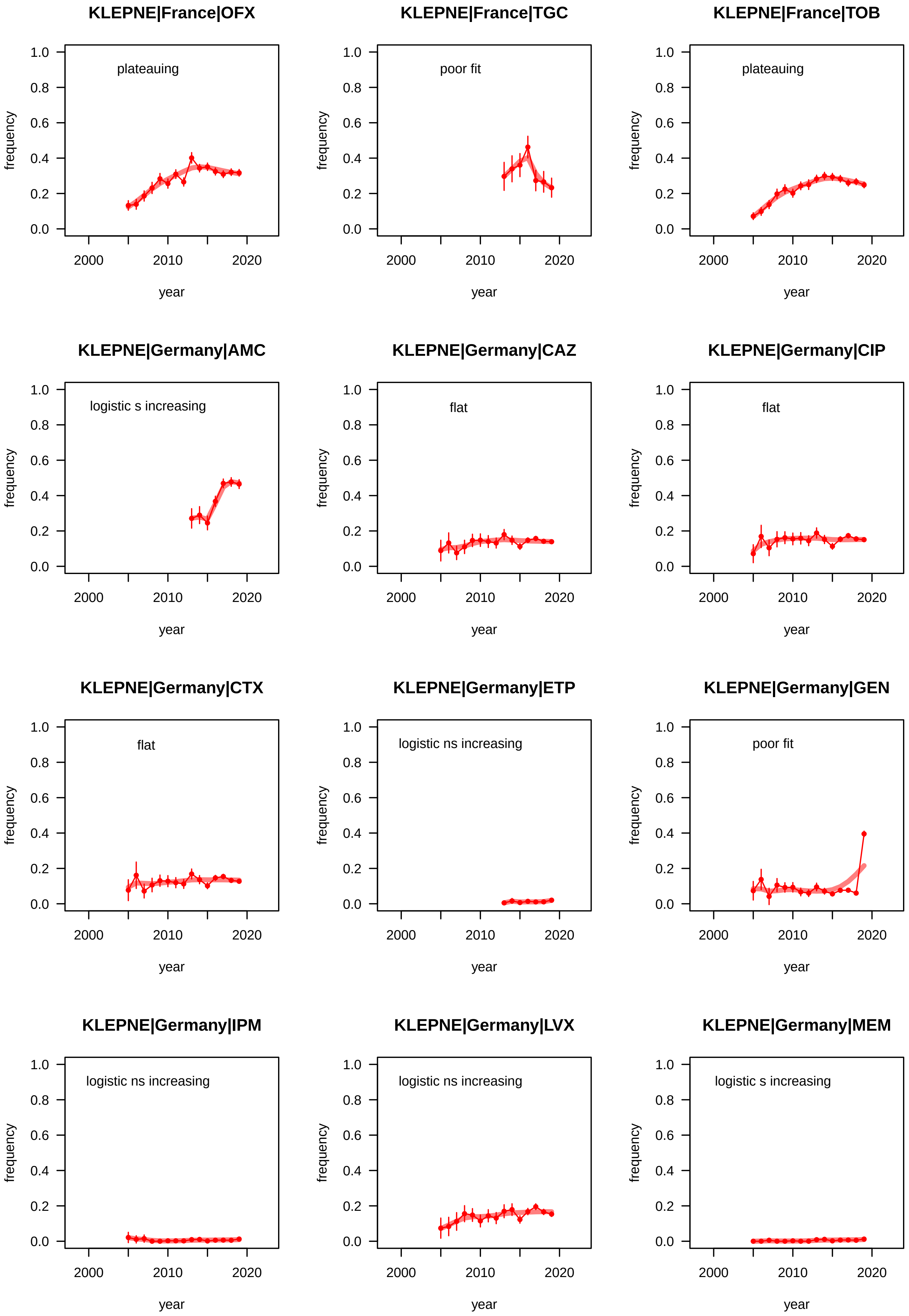
KLEPNE|France|OFX
plateauing
KLEPNE|France|TGC
poor fit
KLEPNE|France|TOB
plateauing
KLEPNE|Germany|AMC
logistic s increasing
KLEPNE|Germany|CAZ
flat
KLEPNE|Germany|CIP
flat
KLEPNE|Germany|CTX
flat
KLEPNE|Germany|ETP
logistic ns increasing
KLEPNE|Germany|GEN
poor fit
KLEPNE|Germany|IPM
logistic ns increasing
KLEPNE|Germany|LVX
logistic ns increasing
KLEPNE|Germany|MEM
logistic s increasing
frequency
year
0.0
0.2
0.4
0.6
0.8
1.0
2000
2010
2020

KLEPNE|Germany|MFX

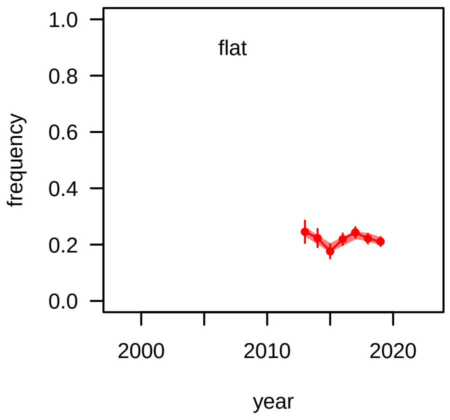

KLEPNE|Germany|PIP

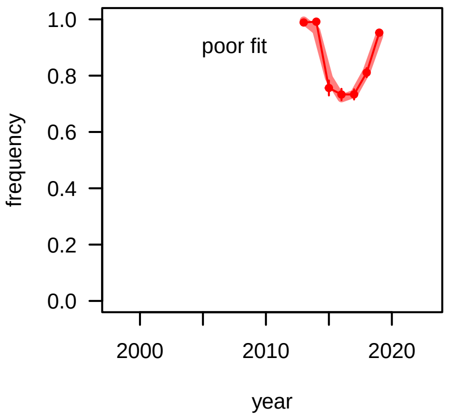

KLEPNE|Germany|TGC

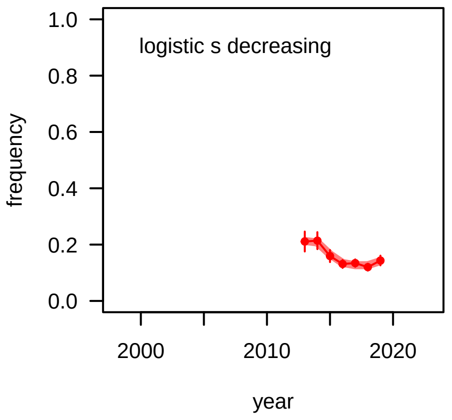

KLEPNE|Germany|TOB

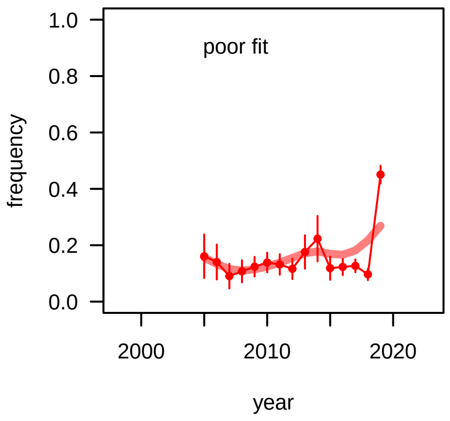

KLEPNE|Germany|TZP

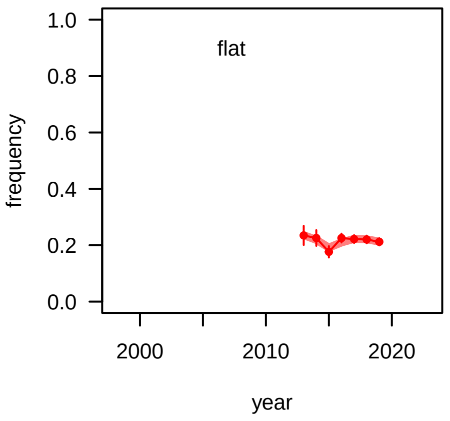

KLEPNE|Greece|AMC

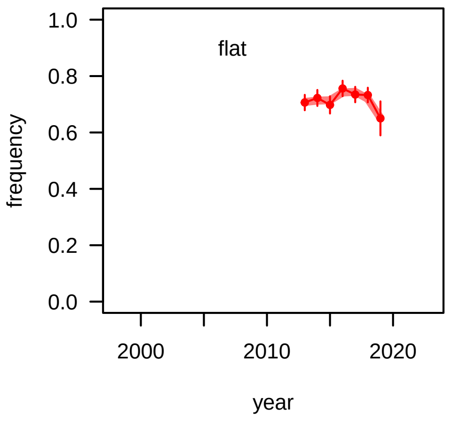

KLEPNE|Greece|AMK

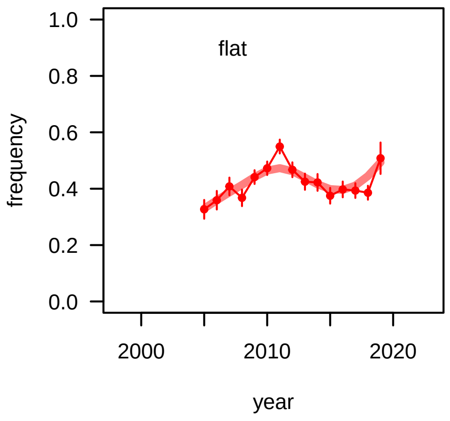

KLEPNE|Greece|CAZ

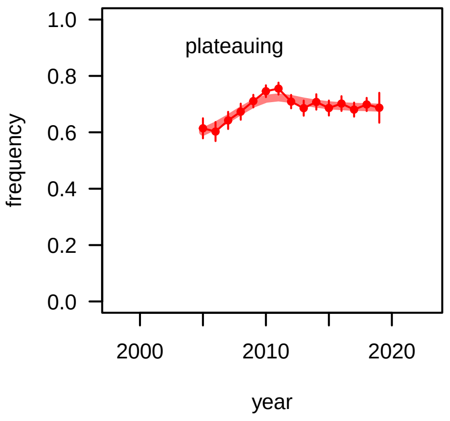

KLEPNE|Greece|CIP

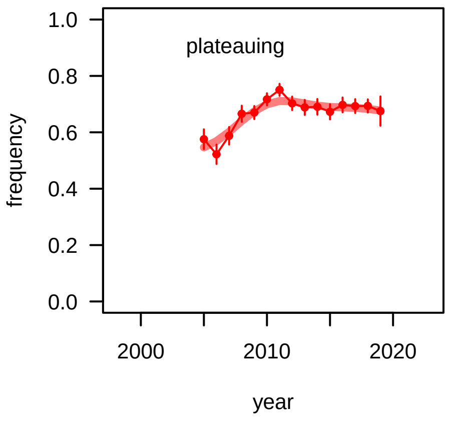

KLEPNE|Greece|CRO

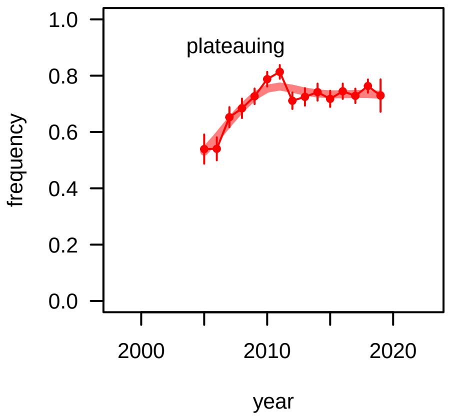

KLEPNE|Greece|CTX

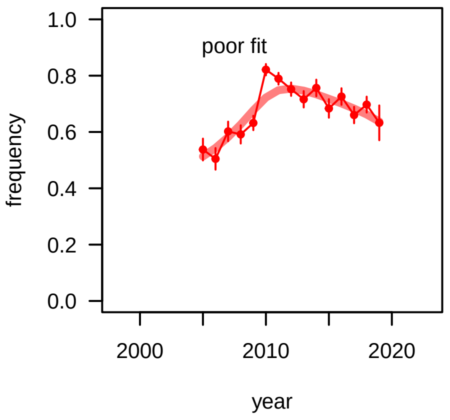

KLEPNE|Greece|ETP

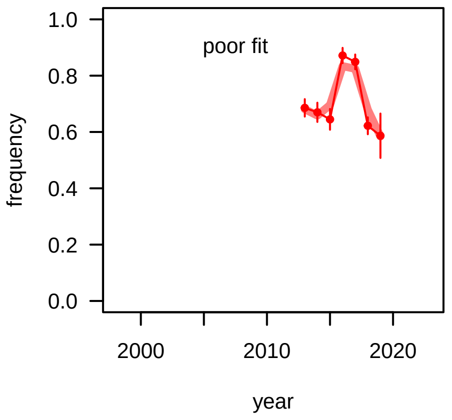

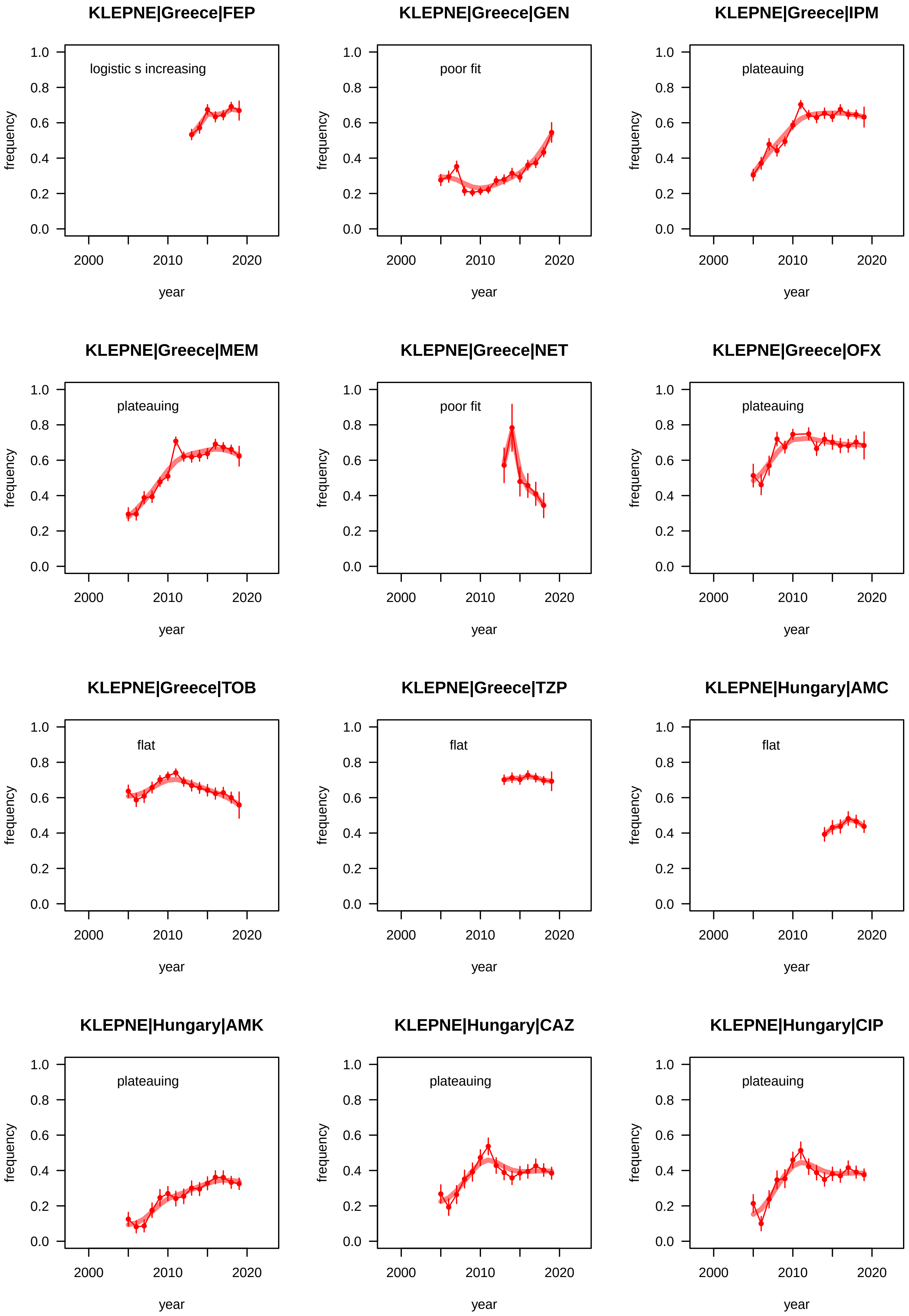
KLEPNE|Greece|FEP
logistic s increasing
KLEPNE|Greece|GEN
poor fit
KLEPNE|Greece|IPM
plateauing
KLEPNE|Greece|MEM
plateauing
KLEPNE|Greece|NET
poor fit
KLEPNE|Greece|OFX
plateauing
KLEPNE|Greece|TOB
flat
KLEPNE|Greece|TZP
flat
KLEPNE|Hungary|AMC
flat
KLEPNE|Hungary|AMK
plateauing
KLEPNE|Hungary|CAZ
plateauing
KLEPNE|Hungary|CIP
plateauing
frequency
year
0.0
0.2
0.4
0.6
0.8
1.0
2000
2010
2020

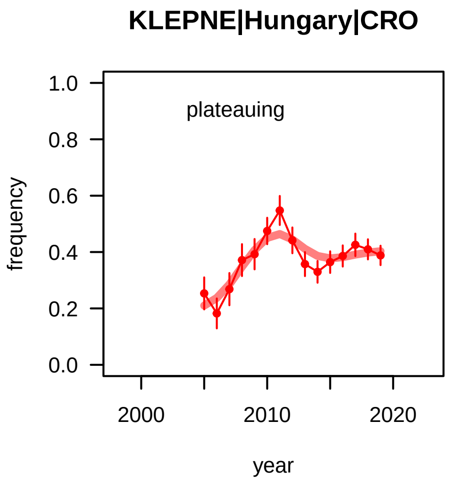
KLEPNE|Hungary|CRO
plateauing
frequency
year

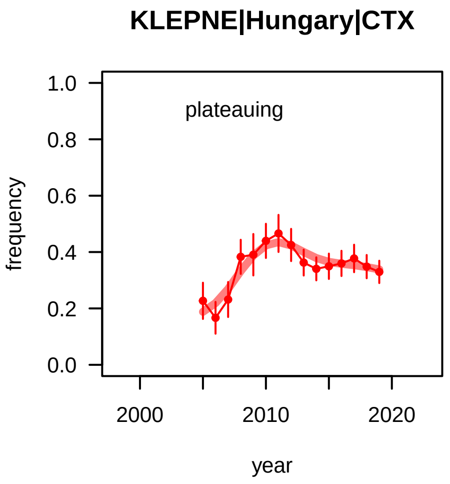
KLEPNE|Hungary|CTX
plateauing
frequency
year

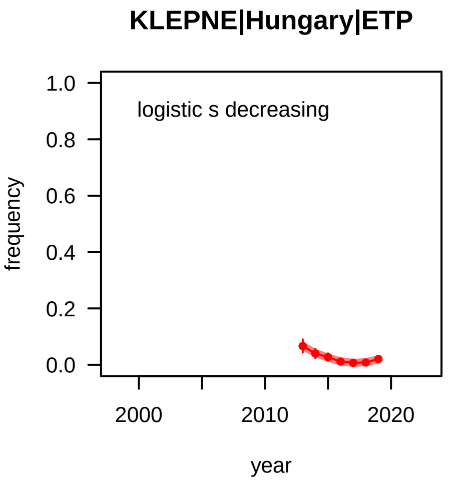
KLEPNE|Hungary|ETP
logistic s decreasing
frequency
year

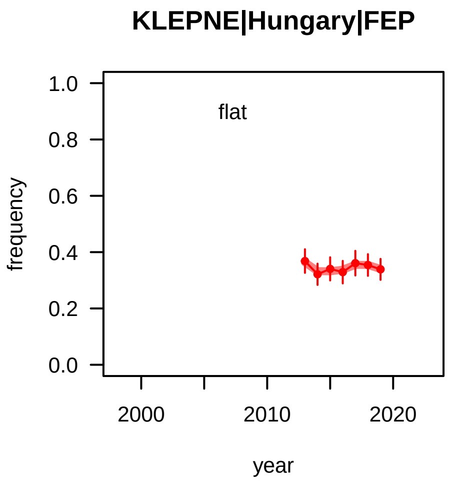
KLEPNE|Hungary|FEP
flat
frequency
year

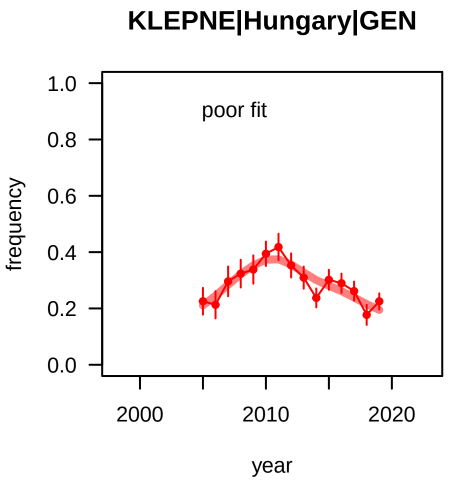
KLEPNE|Hungary|GEN
poor fit
frequency
year

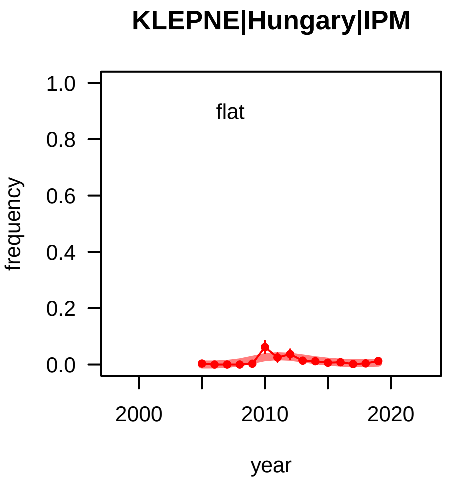
KLEPNE|Hungary|IPM
flat
frequency
year

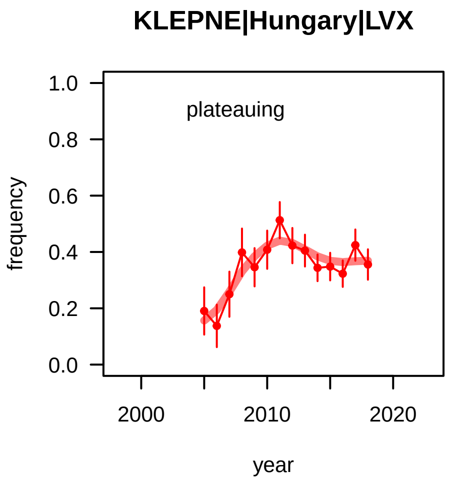
KLEPNE|Hungary|LVX
plateauing
frequency
year

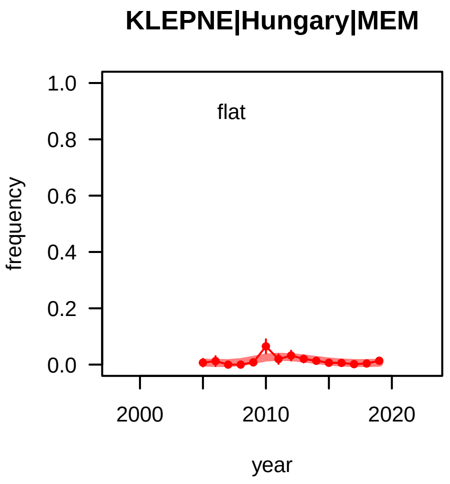
KLEPNE|Hungary|MEM
flat
frequency
year

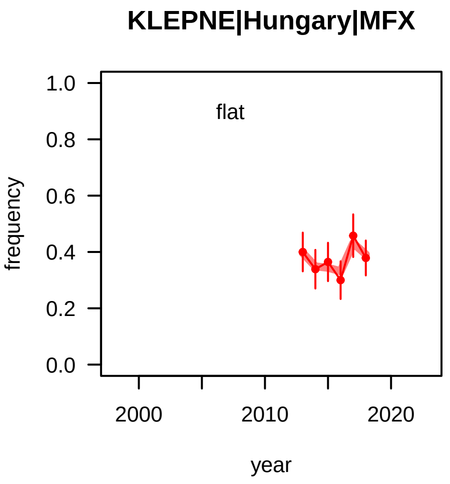
KLEPNE|Hungary|MFX
flat
frequency
year

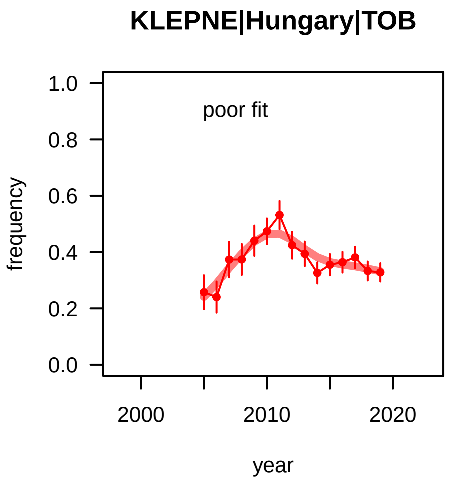
KLEPNE|Hungary|TOB
poor fit
frequency
year

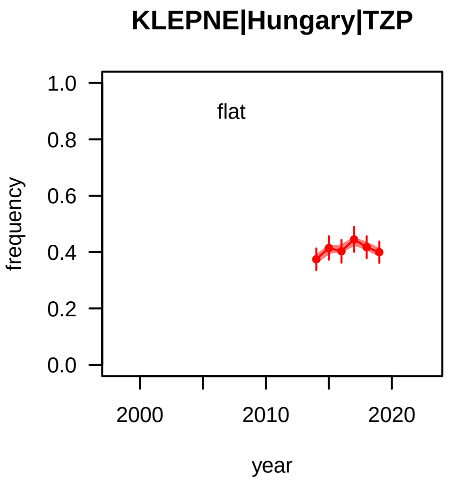
KLEPNE|Hungary|TZP
flat
frequency
year

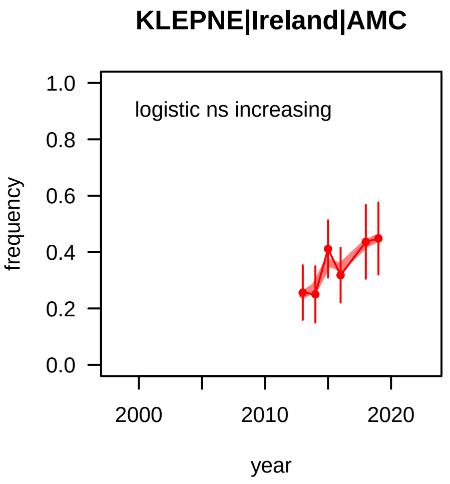
KLEPNE|Ireland|AMC
logistic ns increasing
frequency
year

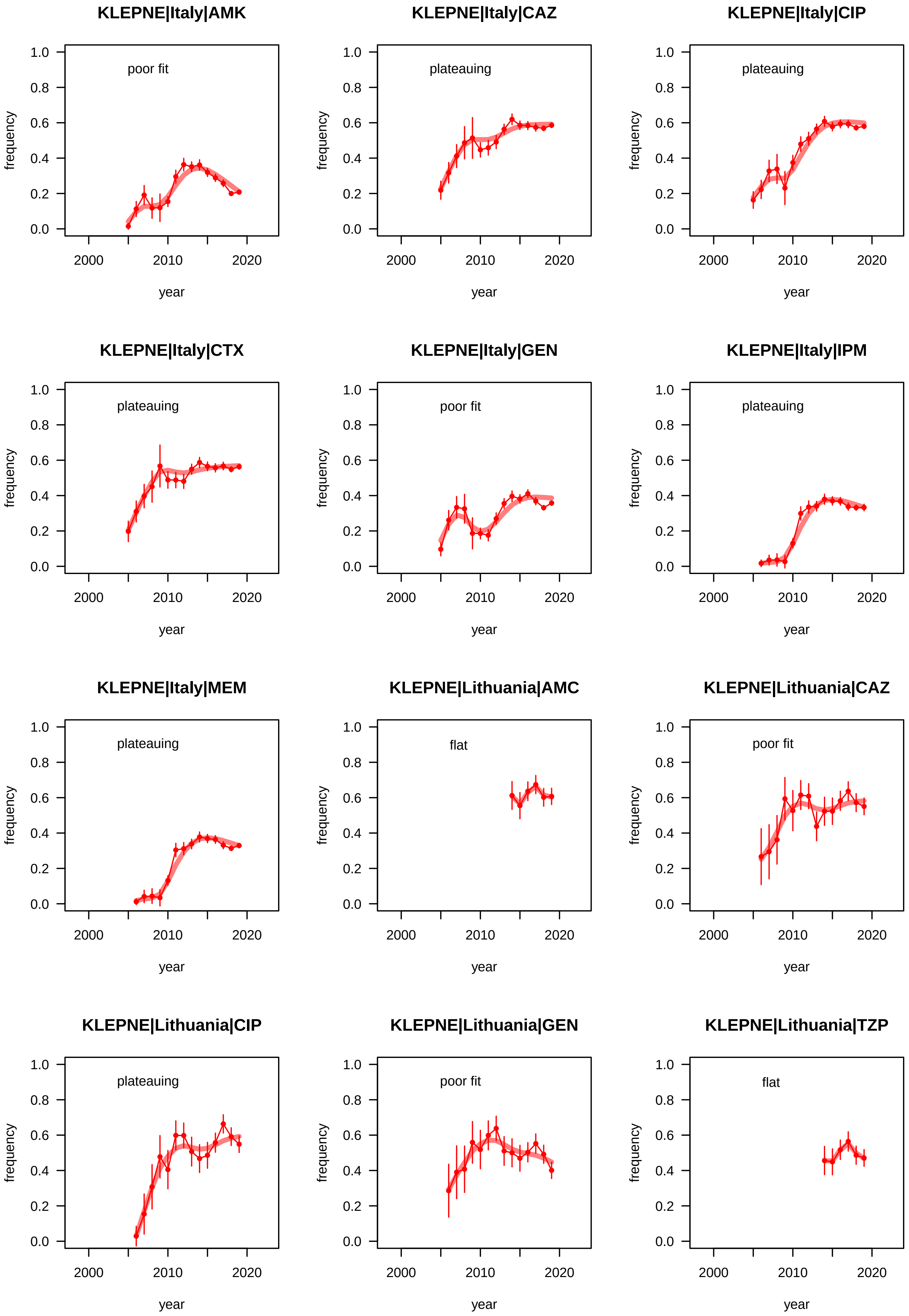
KLEPNE|Italy|AMK
poor fit
KLEPNE|Italy|CAZ
plateauing
KLEPNE|Italy|CIP
plateauing
KLEPNE|Italy|CTX
plateauing
KLEPNE|Italy|GEN
poor fit
KLEPNE|Italy|IPM
plateauing
KLEPNE|Italy|MEM
plateauing
KLEPNE|Lithuania|AMC
flat
KLEPNE|Lithuania|CAZ
poor fit
KLEPNE|Lithuania|CIP
plateauing
KLEPNE|Lithuania|GEN
poor fit
KLEPNE|Lithuania|TZP
flat
frequency
0.0
0.2
0.4
0.6
0.8
1.0
year
2000
2010
2020

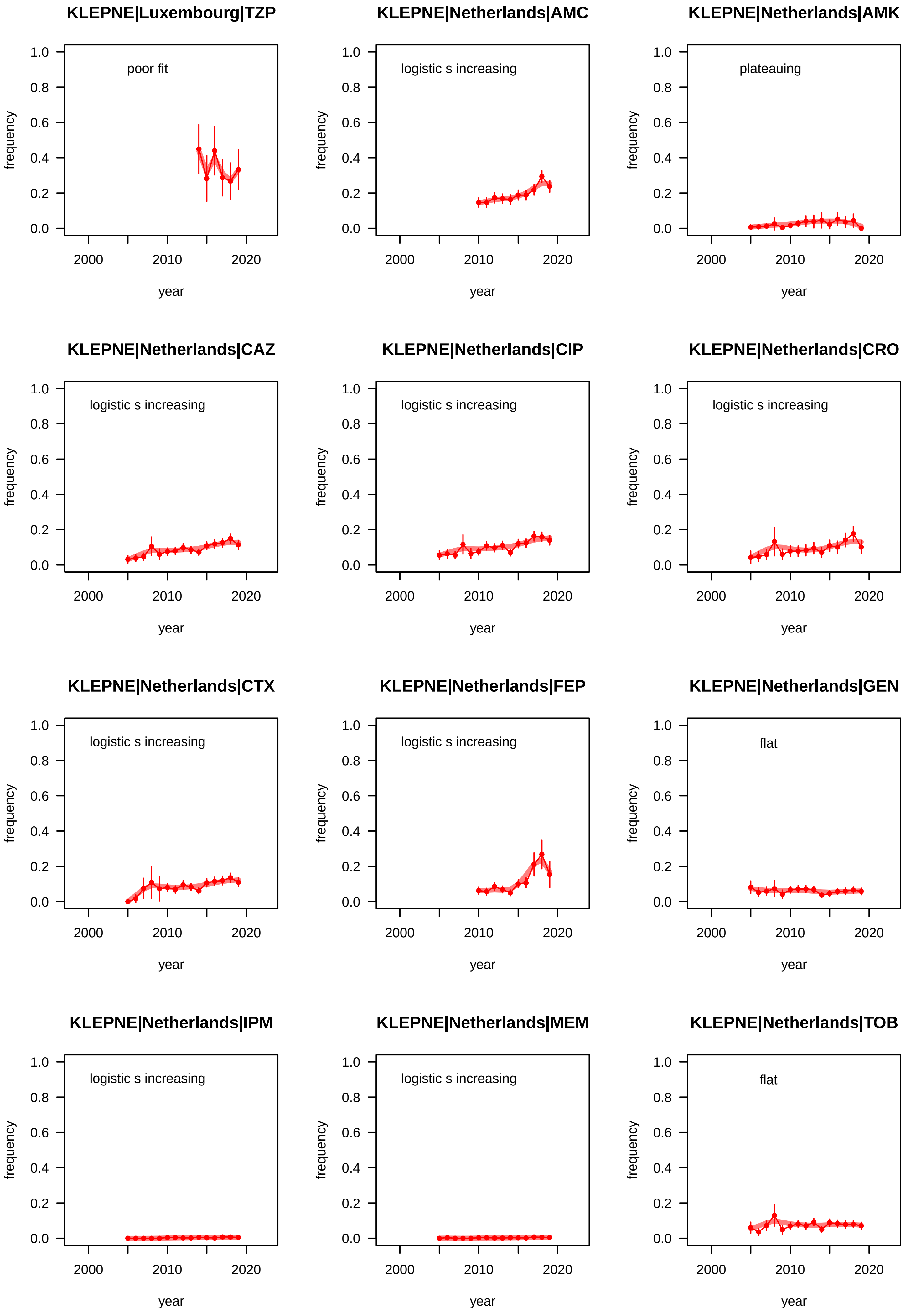

KLEPNE|Luxembourg|TZP
poor fit
KLEPNE|Netherlands|AMC
logistic s increasing
KLEPNE|Netherlands|AMK
plateauing
KLEPNE|Netherlands|CAZ
logistic s increasing
KLEPNE|Netherlands|CIP
logistic s increasing
KLEPNE|Netherlands|CRO
logistic s increasing
KLEPNE|Netherlands|CTX
logistic s increasing
KLEPNE|Netherlands|FEP
logistic s increasing
KLEPNE|Netherlands|GEN
flat
KLEPNE|Netherlands|IPM
logistic s increasing
KLEPNE|Netherlands|MEM
logistic s increasing
KLEPNE|Netherlands|TOB
flat
frequency
year
0.0
0.2
0.4
0.6
0.8
1.0
2000
2010
2020

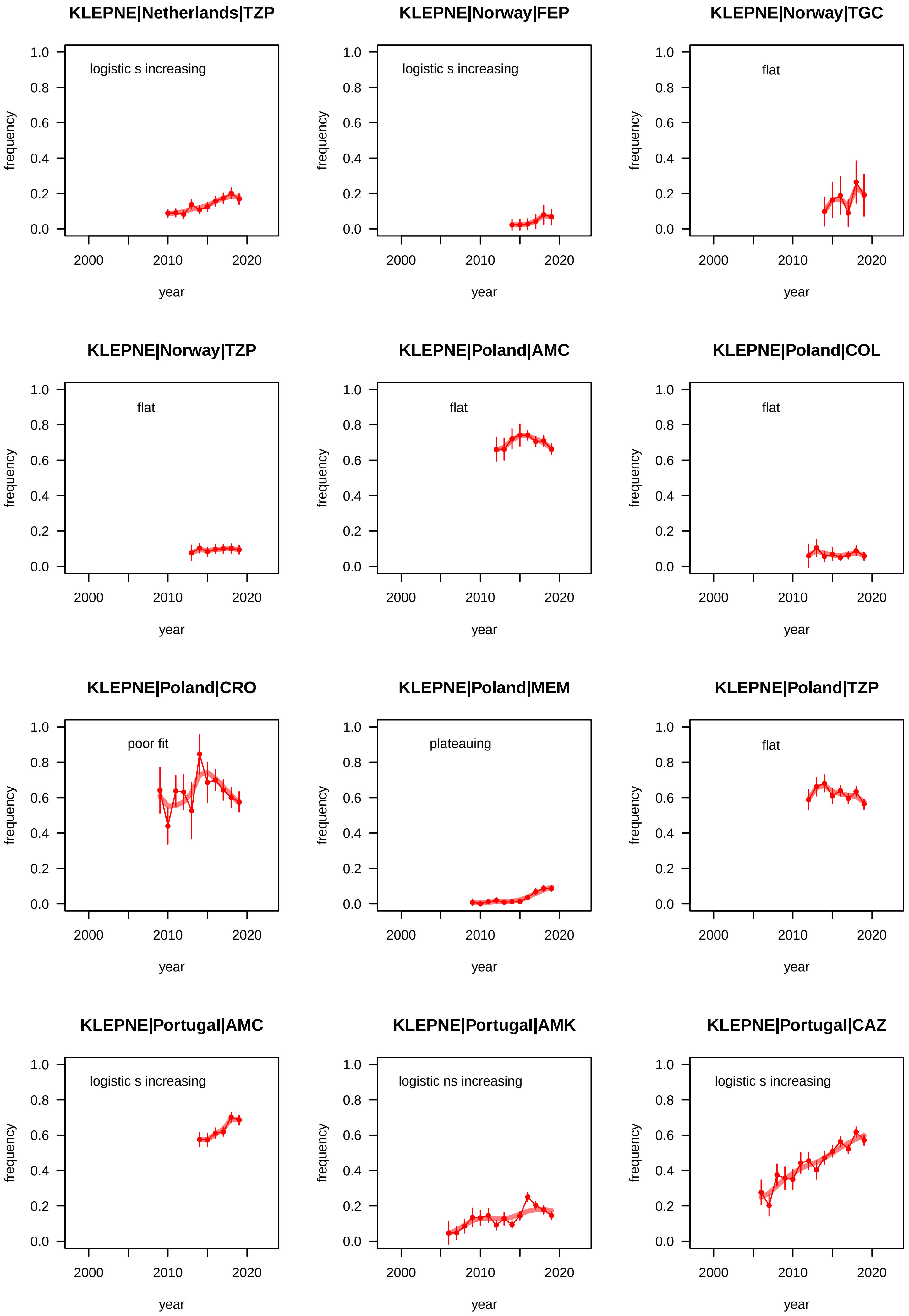
KLEPNE|Netherlands|TZP
logistic s increasing
KLEPNE|Norway|FEP
logistic s increasing
KLEPNE|Norway|TGC
flat
KLEPNE|Norway|TZP
flat
KLEPNE|Poland|AMC
flat
KLEPNE|Poland|COL
flat
KLEPNE|Poland|CRO
poor fit
KLEPNE|Poland|MEM
plateauing
KLEPNE|Poland|TZP
flat
KLEPNE|Portugal|AMC
logistic s increasing
KLEPNE|Portugal|AMK
logistic ns increasing
KLEPNE|Portugal|CAZ
logistic s increasing
frequency
year
0.0
0.2
0.4
0.6
0.8
1.0
2000
2010
2020

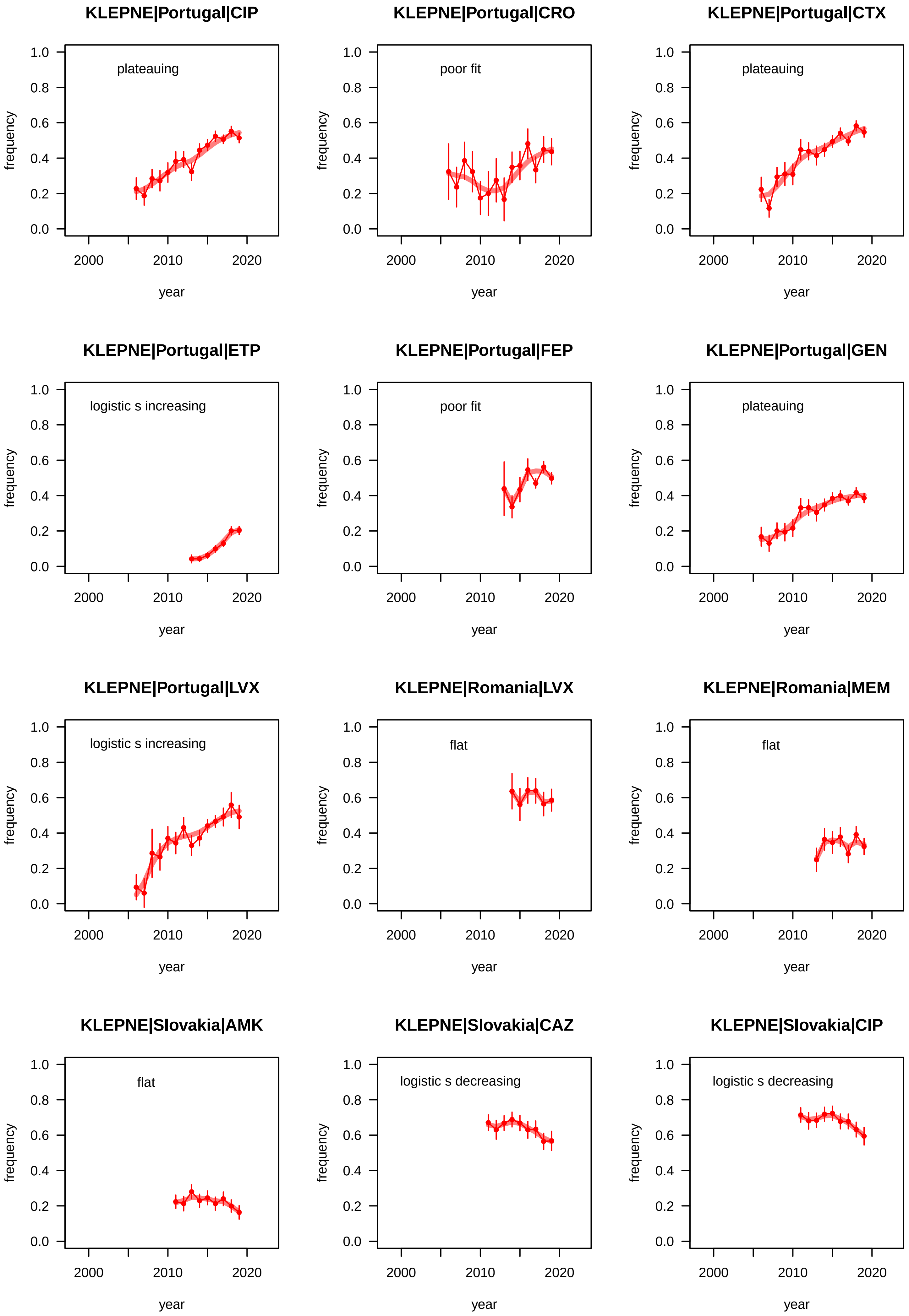
KLEPNE|Portugal|CIP
plateauing
KLEPNE|Portugal|CRO
poor fit
KLEPNE|Portugal|CTX
plateauing
KLEPNE|Portugal|ETP
logistic s increasing
KLEPNE|Portugal|FEP
poor fit
KLEPNE|Portugal|GEN
plateauing
KLEPNE|Portugal|LVX
logistic s increasing
KLEPNE|Romania|LVX
flat
KLEPNE|Romania|MEM
flat
KLEPNE|Slovakia|AMK
flat
KLEPNE|Slovakia|CAZ
logistic s decreasing
KLEPNE|Slovakia|CIP
logistic s decreasing
frequency
year
0.0
0.2
0.4
0.6
0.8
1.0
2000
2010
2020

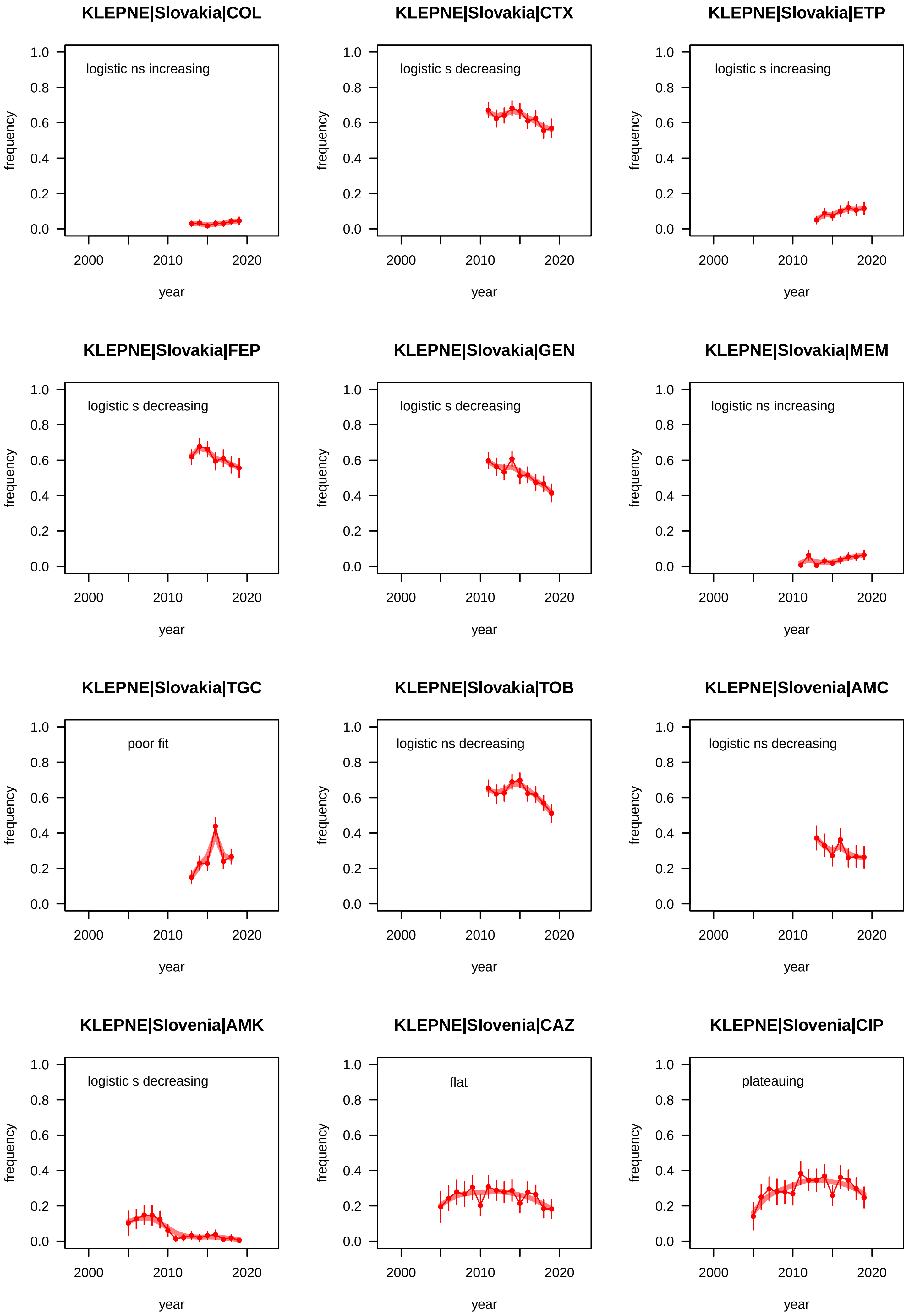
KLEPNE|Slovakia|COL
logistic ns increasing
KLEPNE|Slovakia|CTX
logistic s decreasing
KLEPNE|Slovakia|ETP
logistic s increasing
KLEPNE|Slovakia|FEP
logistic s decreasing
KLEPNE|Slovakia|GEN
logistic s decreasing
KLEPNE|Slovakia|MEM
logistic ns increasing
KLEPNE|Slovakia|TGC
poor fit
KLEPNE|Slovakia|TOB
logistic ns decreasing
KLEPNE|Slovenia|AMC
logistic ns decreasing
KLEPNE|Slovenia|AMK
logistic s decreasing
KLEPNE|Slovenia|CAZ
flat
KLEPNE|Slovenia|CIP
plateauing
frequency
year
0.0
0.2
0.4
0.6
0.8
1.0
2000
2010
2020

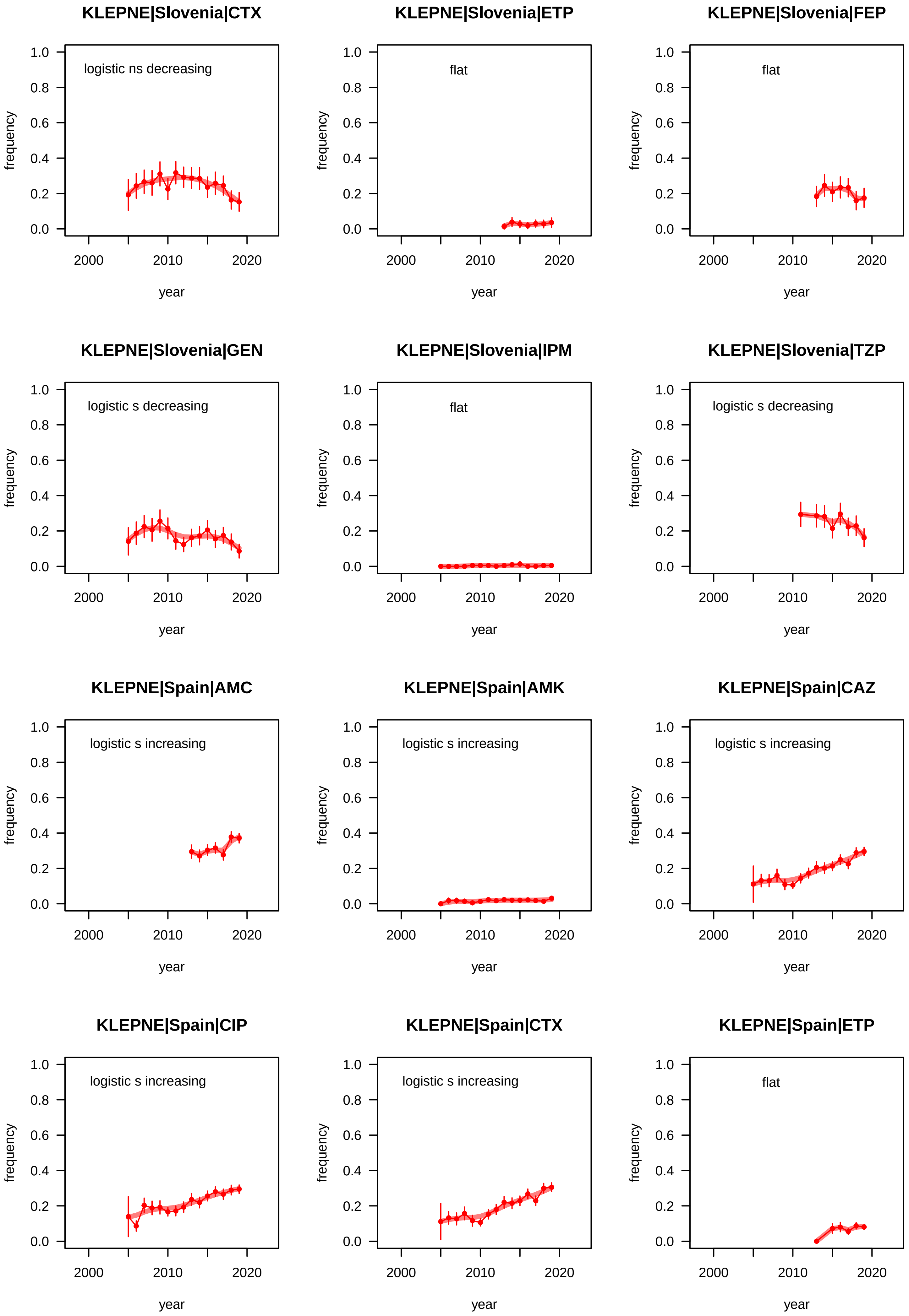
KLEPNE|Slovenia|CTX
logistic ns decreasing
KLEPNE|Slovenia|ETP
flat
KLEPNE|Slovenia|FEP
flat
KLEPNE|Slovenia|GEN
logistic s decreasing
KLEPNE|Slovenia|IPM
flat
KLEPNE|Slovenia|TZP
logistic s decreasing
KLEPNE|Spain|AMC
logistic s increasing
KLEPNE|Spain|AMK
logistic s increasing
KLEPNE|Spain|CAZ
logistic s increasing
KLEPNE|Spain|CIP
logistic s increasing
KLEPNE|Spain|CTX
logistic s increasing
KLEPNE|Spain|ETP
flat
frequency
year
0.0
0.2
0.4
0.6
0.8
1.0
2000
2010
2020

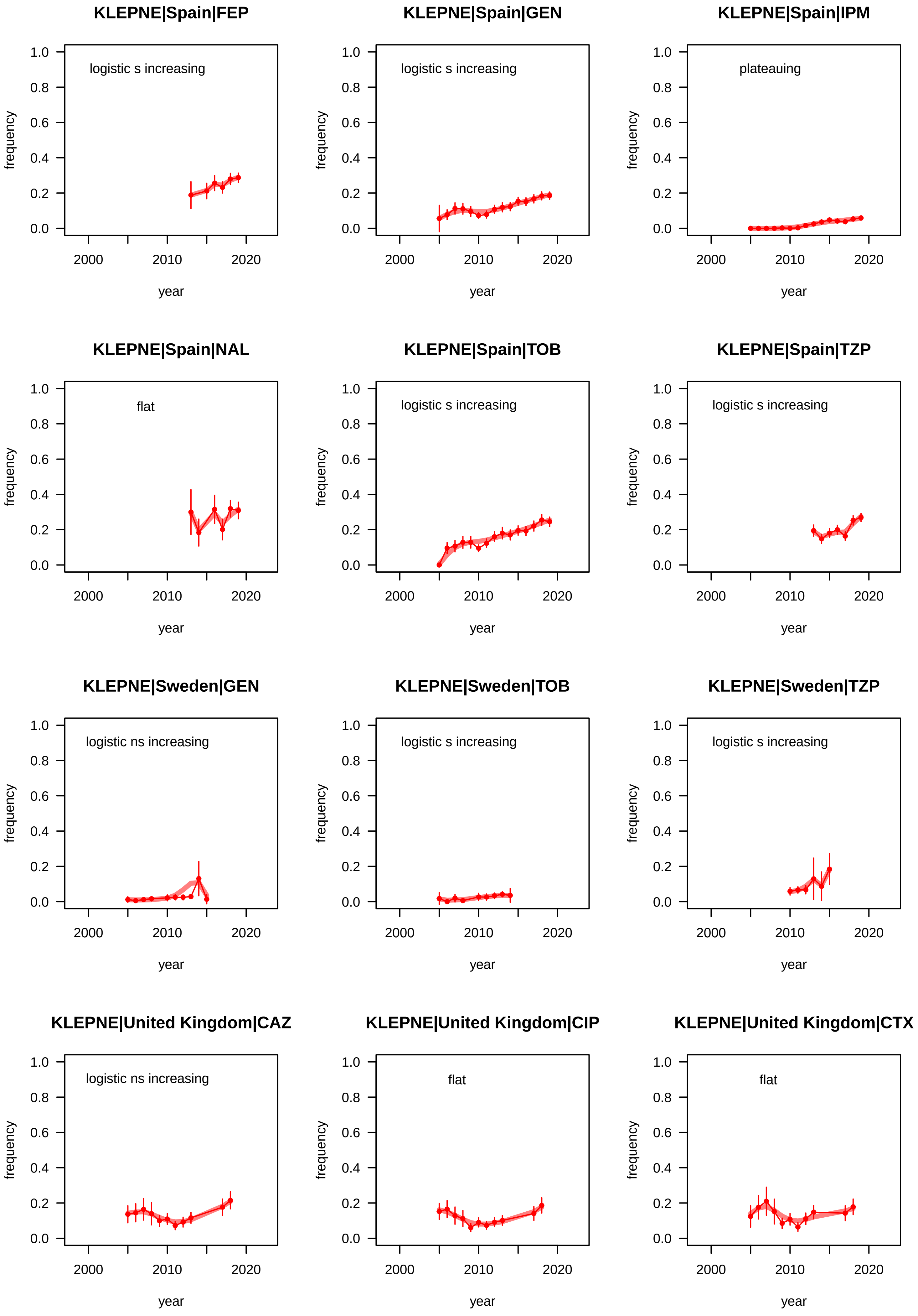

KLEPNE|Spain|FEP
logistic s increasing
KLEPNE|Spain|GEN
logistic s increasing
KLEPNE|Spain|IPM
plateauing
KLEPNE|Spain|NAL
flat
KLEPNE|Spain|TOB
logistic s increasing
KLEPNE|Spain|TZP
logistic s increasing
KLEPNE|Sweden|GEN
logistic ns increasing
KLEPNE|Sweden|TOB
logistic s increasing
KLEPNE|Sweden|TZP
logistic s increasing
KLEPNE|United Kingdom|CAZ
logistic ns increasing
KLEPNE|United Kingdom|CIP
flat
KLEPNE|United Kingdom|CTX
flat
frequency
year
0.0
0.2
0.4
0.6
0.8
1.0
2000
2010
2020

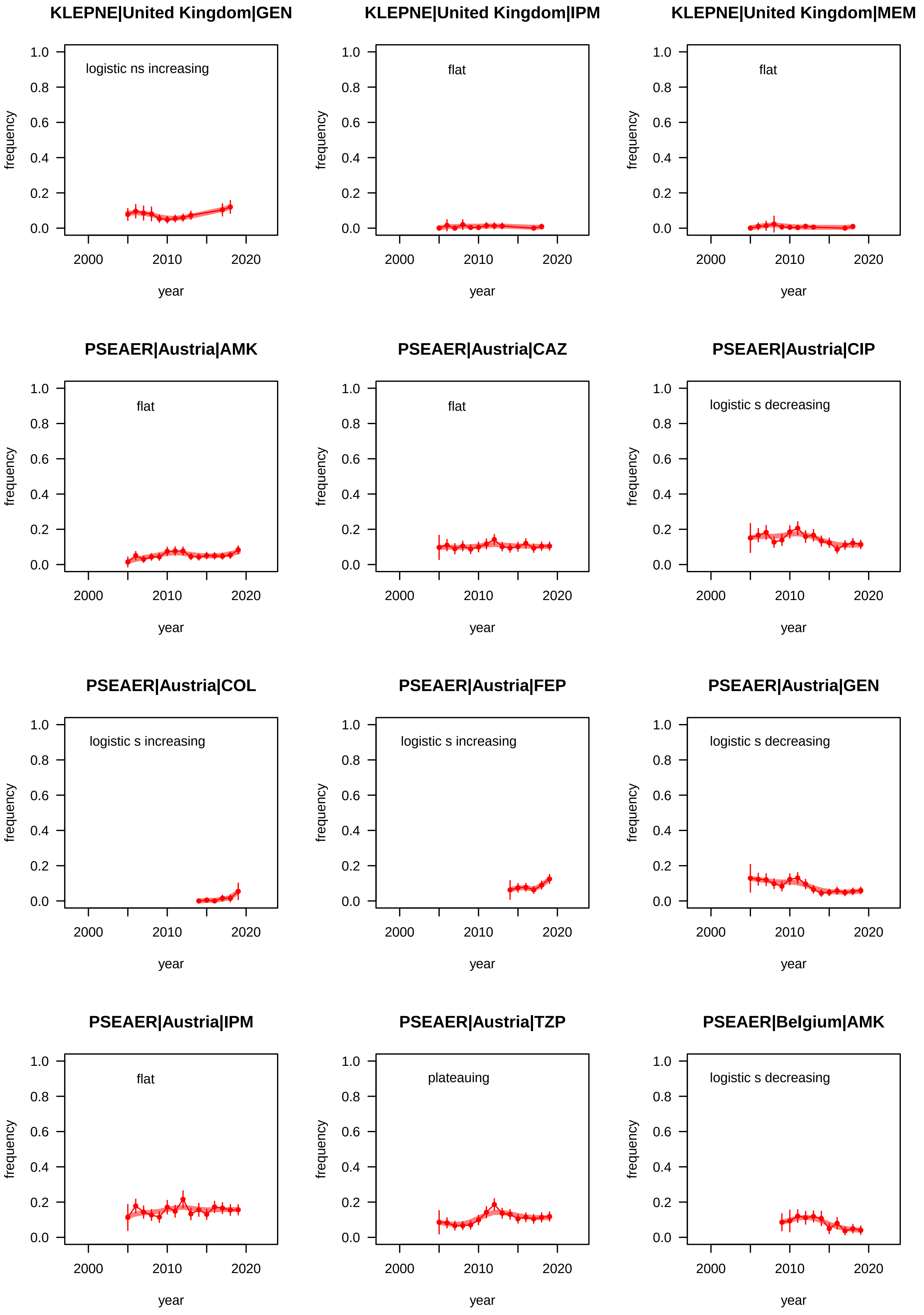
KLEPNE|United Kingdom|GEN
logistic ns increasing
KLEPNE|United Kingdom|IPM
flat
KLEPNE|United Kingdom|MEM
flat
PSEAER|Austria|AMK
flat
PSEAER|Austria|CAZ
flat
PSEAER|Austria|CIP
logistic s decreasing
PSEAER|Austria|COL
logistic s increasing
PSEAER|Austria|FEP
logistic s increasing
PSEAER|Austria|GEN
logistic s decreasing
PSEAER|Austria|IPM
flat
PSEAER|Austria|TZP
plateauing
PSEAER|Belgium|AMK
logistic s decreasing
frequency
0.0
0.2
0.4
0.6
0.8
1.0
year
2000
2010
2020

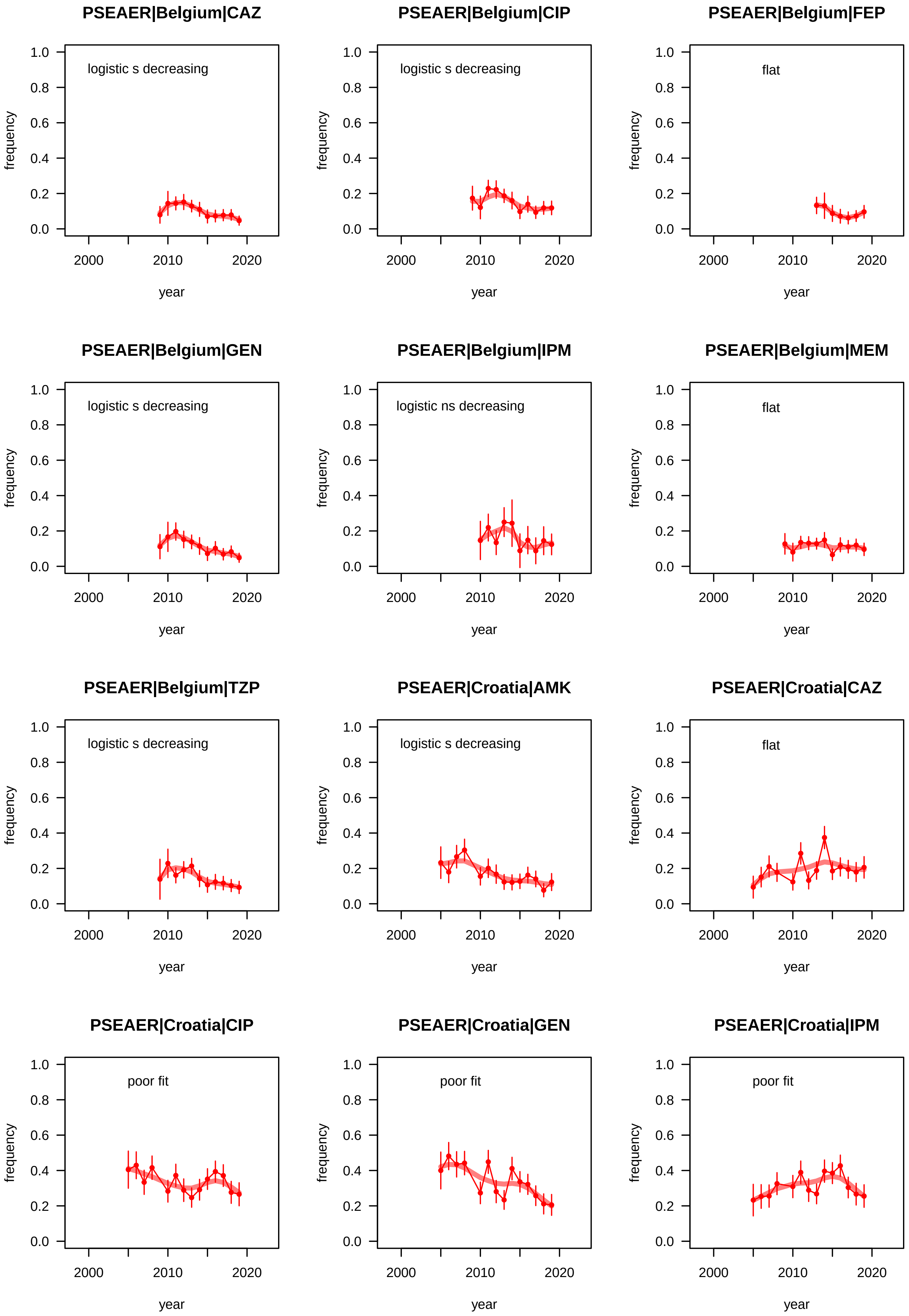

PSEAER|Belgium|CAZ
logistic s decreasing
PSEAER|Belgium|CIP
logistic s decreasing
PSEAER|Belgium|FEP
flat
PSEAER|Belgium|GEN
logistic s decreasing
PSEAER|Belgium|IPM
logistic ns decreasing
PSEAER|Belgium|MEM
flat
PSEAER|Belgium|TZP
logistic s decreasing
PSEAER|Croatia|AMK
logistic s decreasing
PSEAER|Croatia|CAZ
flat
PSEAER|Croatia|CIP
poor fit
PSEAER|Croatia|GEN
poor fit
PSEAER|Croatia|IPM
poor fit
frequency
0.0
0.2
0.4
0.6
0.8
1.0
year
2000
2010
2020

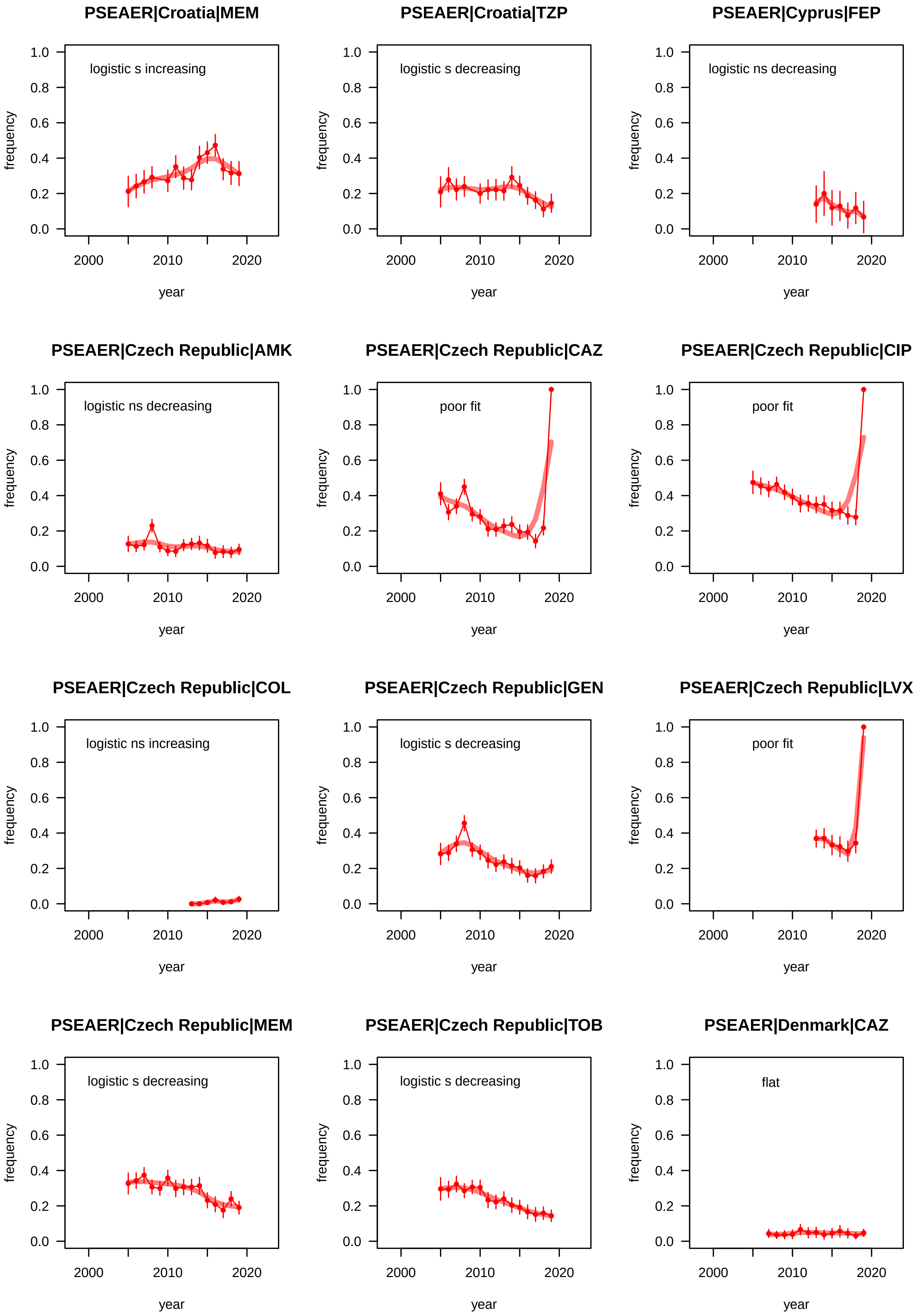
PSEAER|Croatia|MEM
logistic s increasing
PSEAER|Croatia|TZP
logistic s decreasing
PSEAER|Cyprus|FEP
logistic ns decreasing
PSEAER|Czech Republic|AMK
logistic ns decreasing
PSEAER|Czech Republic|CAZ
poor fit
PSEAER|Czech Republic|CIP
poor fit
PSEAER|Czech Republic|COL
logistic ns increasing
PSEAER|Czech Republic|GEN
logistic s decreasing
PSEAER|Czech Republic|LVX
poor fit
PSEAER|Czech Republic|MEM
logistic s decreasing
PSEAER|Czech Republic|TOB
logistic s decreasing
PSEAER|Denmark|CAZ
flat
frequency
year
0.0
0.2
0.4
0.6
0.8
1.0
2000
2010
2020

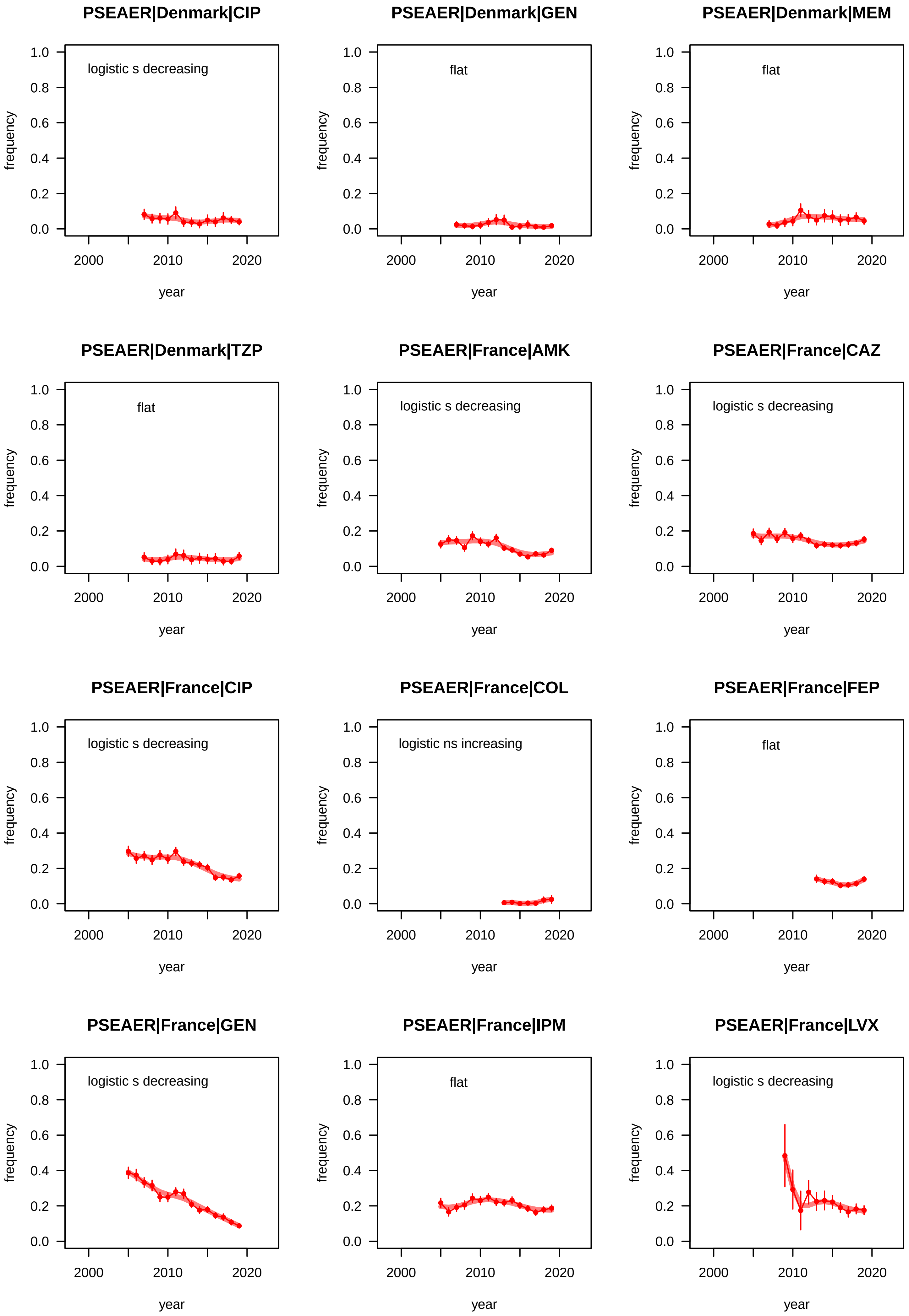
PSEAER|Denmark|CIP
logistic s decreasing
PSEAER|Denmark|GEN
flat
PSEAER|Denmark|MEM
flat
PSEAER|Denmark|TZP
flat
PSEAER|France|AMK
logistic s decreasing
PSEAER|France|CAZ
logistic s decreasing
PSEAER|France|CIP
logistic s decreasing
PSEAER|France|COL
logistic ns increasing
PSEAER|France|FEP
flat
PSEAER|France|GEN
logistic s decreasing
PSEAER|France|IPM
flat
PSEAER|France|LVX
logistic s decreasing
frequency
year
0.0
0.2
0.4
0.6
0.8
1.0
2000
2010
2020

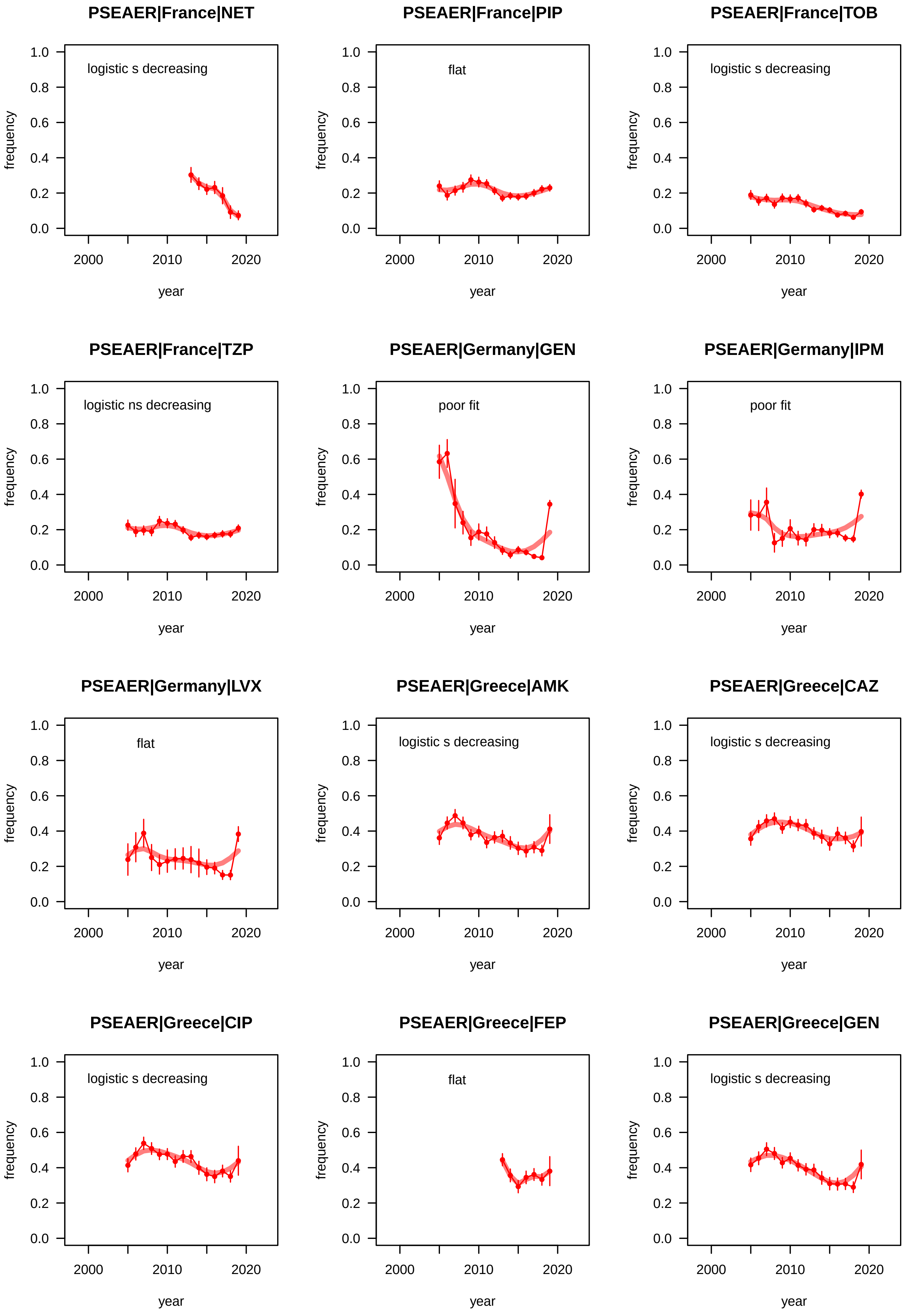

PSEAER|France|NET
logistic s decreasing
PSEAER|France|PIP
flat
PSEAER|France|TOB
logistic s decreasing
PSEAER|France|TZP
logistic ns decreasing
PSEAER|Germany|GEN
poor fit
PSEAER|Germany|IPM
poor fit
PSEAER|Germany|LVX
flat
PSEAER|Greece|AMK
logistic s decreasing
PSEAER|Greece|CAZ
logistic s decreasing
PSEAER|Greece|CIP
logistic s decreasing
PSEAER|Greece|FEP
flat
PSEAER|Greece|GEN
logistic s decreasing
frequency
year
0.0
0.2
0.4
0.6
0.8
1.0
2000
2010
2020

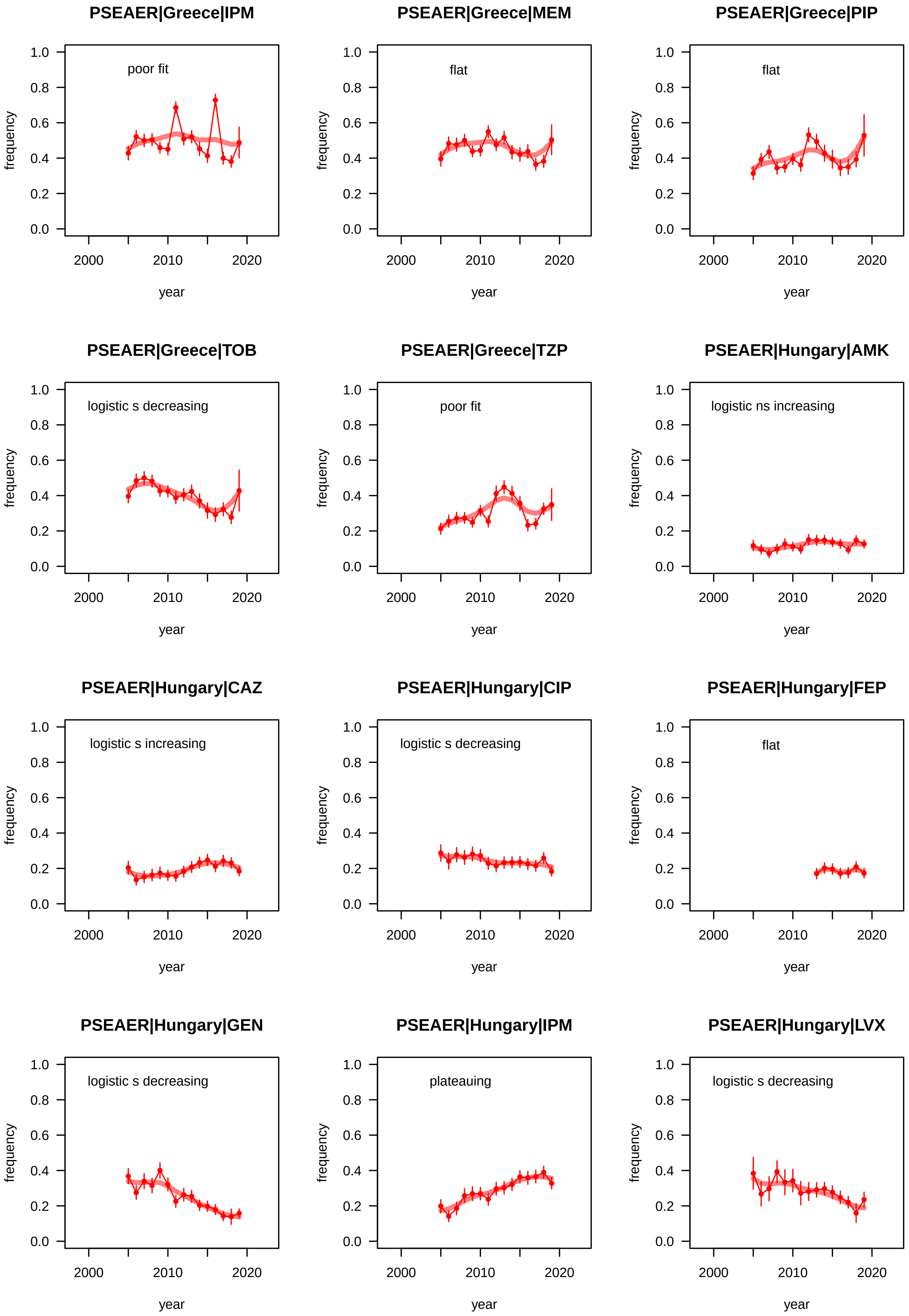
PSEAER|Greece|IPM
poor fit
PSEAER|Greece|MEM
flat
PSEAER|Greece|PIP
flat
PSEAER|Greece|TOB
logistic s decreasing
PSEAER|Greece|TZP
poor fit
PSEAER|Hungary|AMK
logistic ns increasing
PSEAER|Hungary|CAZ
logistic s increasing
PSEAER|Hungary|CIP
logistic s decreasing
PSEAER|Hungary|FEP
flat
PSEAER|Hungary|GEN
logistic s decreasing
PSEAER|Hungary|IPM
plateauing
PSEAER|Hungary|LVX
logistic s decreasing
frequency
year
0.0
0.2
0.4
0.6
0.8
1.0
2000
2010
2020

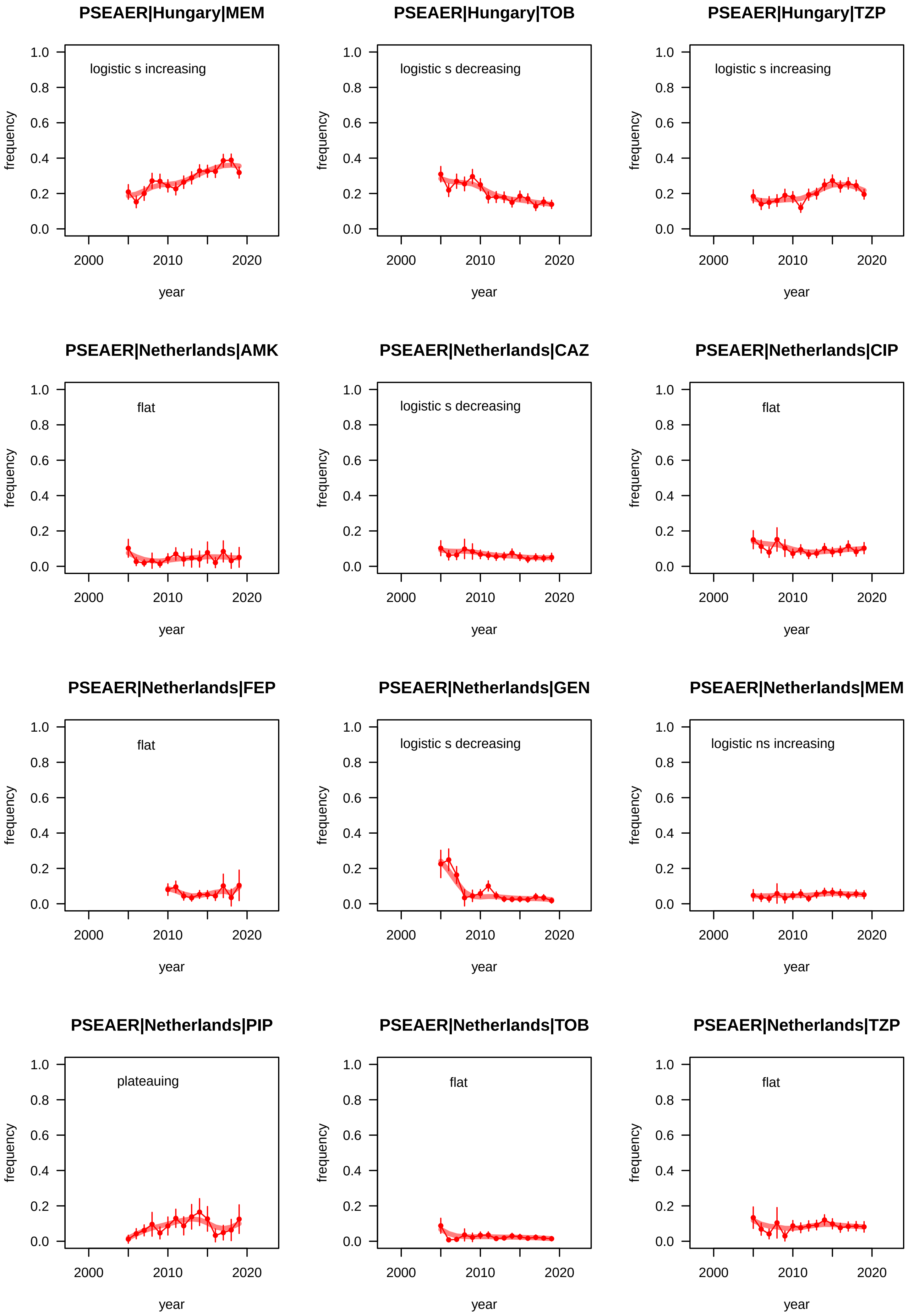
PSEAER|Hungary|MEM
logistic s increasing
PSEAER|Hungary|TOB
logistic s decreasing
PSEAER|Hungary|TZP
logistic s increasing
PSEAER|Netherlands|AMK
flat
PSEAER|Netherlands|CAZ
logistic s decreasing
PSEAER|Netherlands|CIP
flat
PSEAER|Netherlands|FEP
flat
PSEAER|Netherlands|GEN
logistic s decreasing
PSEAER|Netherlands|MEM
logistic ns increasing
PSEAER|Netherlands|PIP
plateauing
PSEAER|Netherlands|TOB
flat
PSEAER|Netherlands|TZP
flat
frequency
0.0
0.2
0.4
0.6
0.8
1.0
year
2000
2010
2020

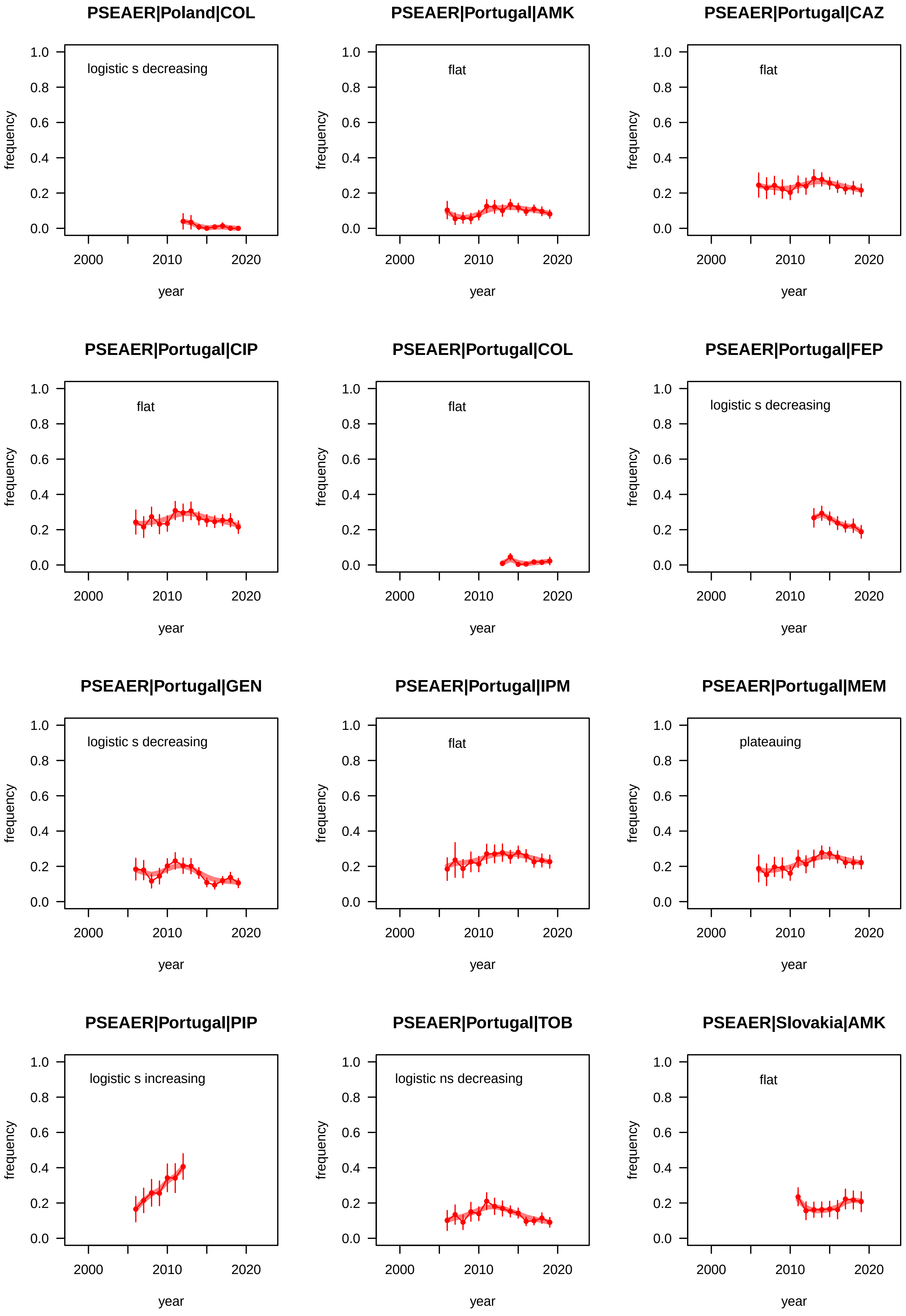
PSEAER|Poland|COL
logistic s decreasing
PSEAER|Portugal|AMK
flat
PSEAER|Portugal|CAZ
flat
PSEAER|Portugal|CIP
flat
PSEAER|Portugal|COL
flat
PSEAER|Portugal|FEP
logistic s decreasing
PSEAER|Portugal|GEN
logistic s decreasing
PSEAER|Portugal|IPM
flat
PSEAER|Portugal|MEM
plateauing
PSEAER|Portugal|PIP
logistic s increasing
PSEAER|Portugal|TOB
logistic ns decreasing
PSEAER|Slovakia|AMK
flat
frequency
year
0.0
0.2
0.4
0.6
0.8
1.0
2000
2010
2020

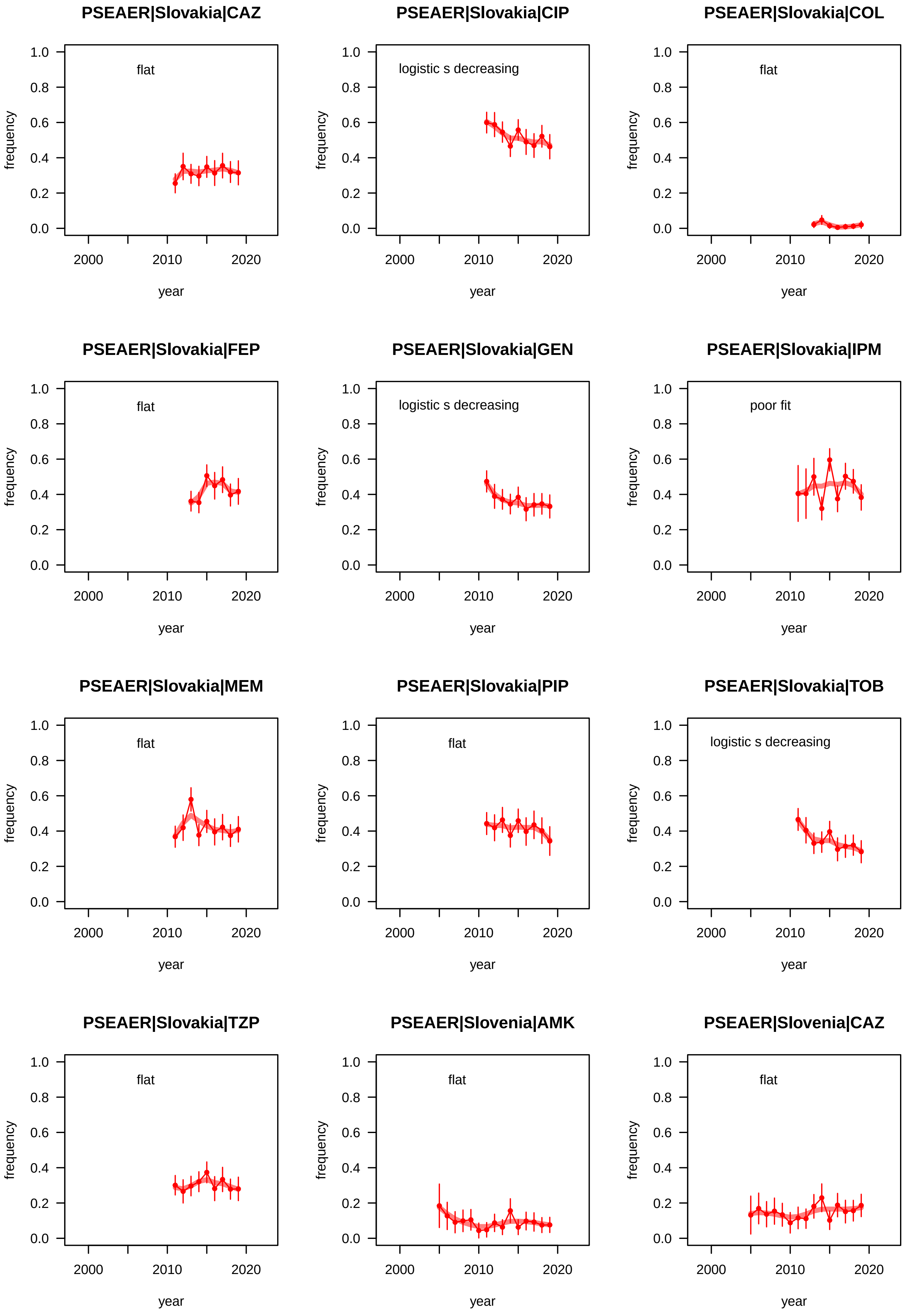
PSEAER|Slovakia|CAZ
flat
PSEAER|Slovakia|CIP
logistic s decreasing
PSEAER|Slovakia|COL
flat
PSEAER|Slovakia|FEP
flat
PSEAER|Slovakia|GEN
logistic s decreasing
PSEAER|Slovakia|IPM
poor fit
PSEAER|Slovakia|MEM
flat
PSEAER|Slovakia|PIP
flat
PSEAER|Slovakia|TOB
logistic s decreasing
PSEAER|Slovakia|TZP
flat
PSEAER|Slovenia|AMK
flat
PSEAER|Slovenia|CAZ
flat
frequency
0.0
0.2
0.4
0.6
0.8
1.0
year
2000
2010
2020

PSEAER|Slovenia|CIP

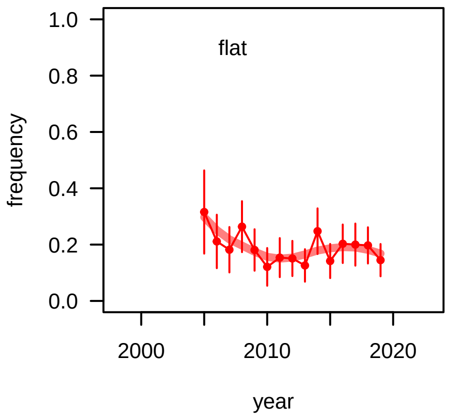

PSEAER|Slovenia|FEP

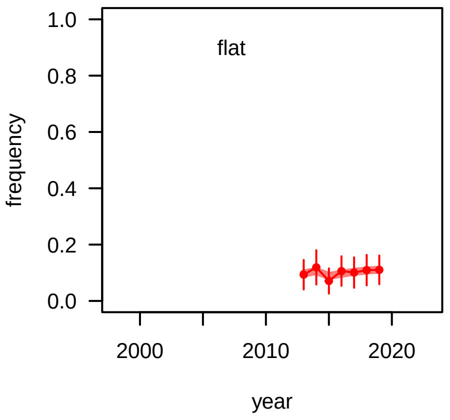

PSEAER|Slovenia|GEN

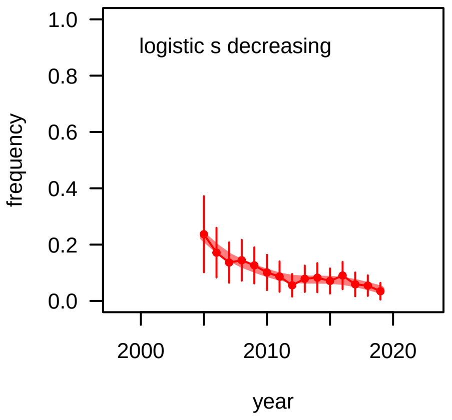

PSEAER|Slovenia|IPM

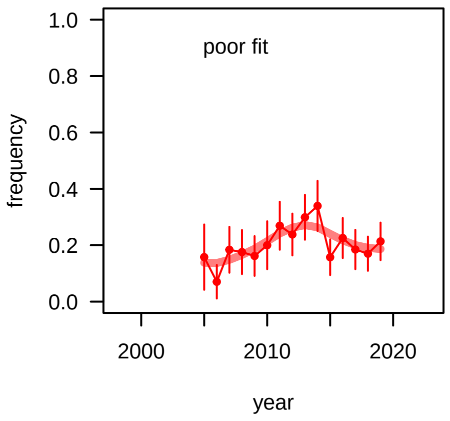

PSEAER|Slovenia|TZP

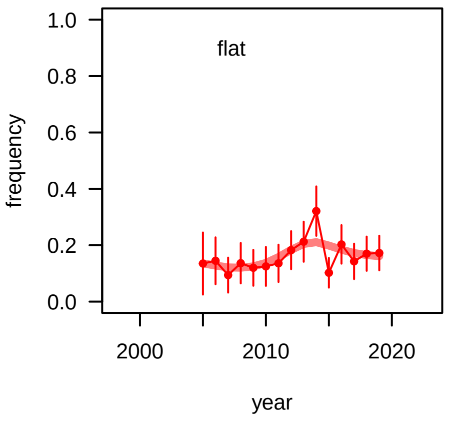

PSEAER|Spain|AMK

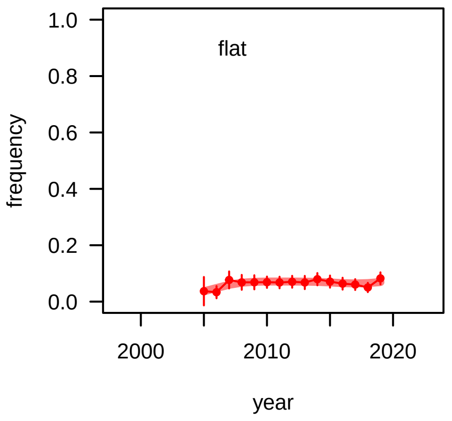

PSEAER|Spain|CAZ

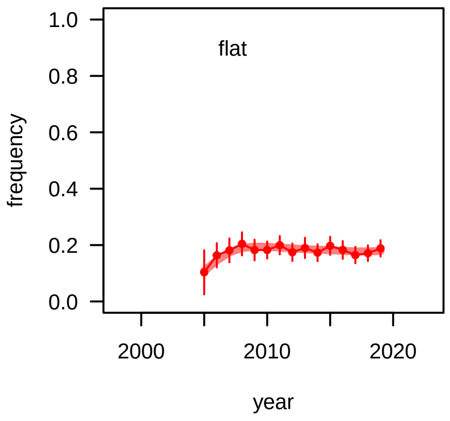

PSEAER|Spain|CIP

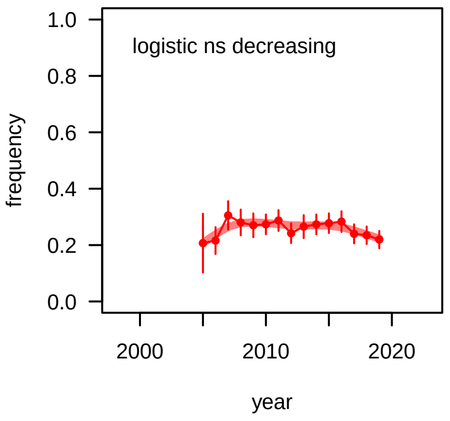

PSEAER|Spain|COL

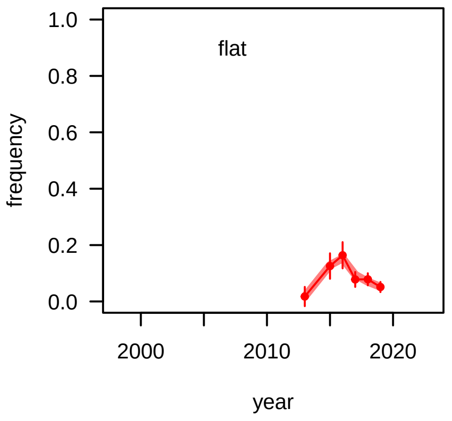

PSEAER|Spain|FEP

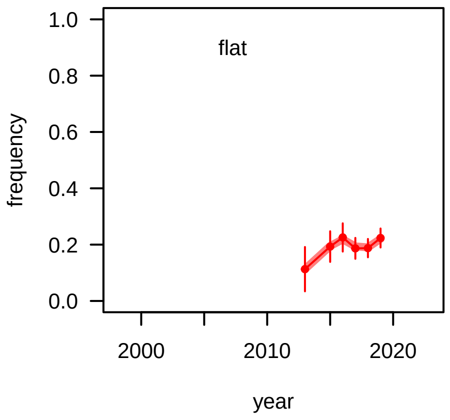

PSEAER|Spain|GEN

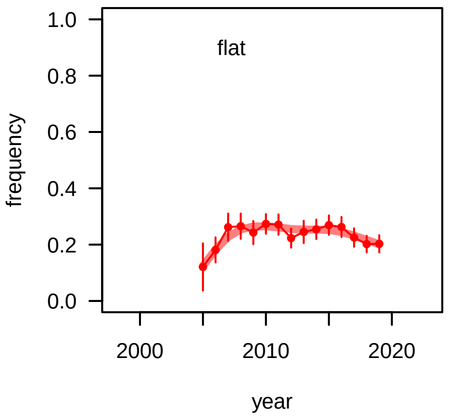

PSEAER|Spain|IPM

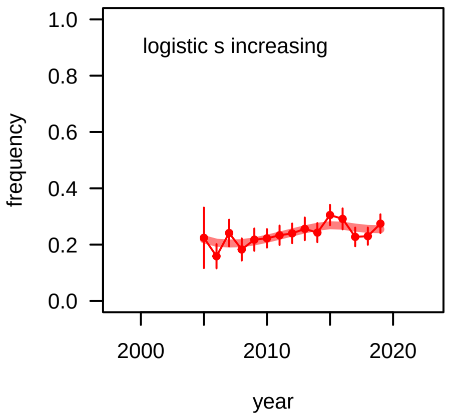

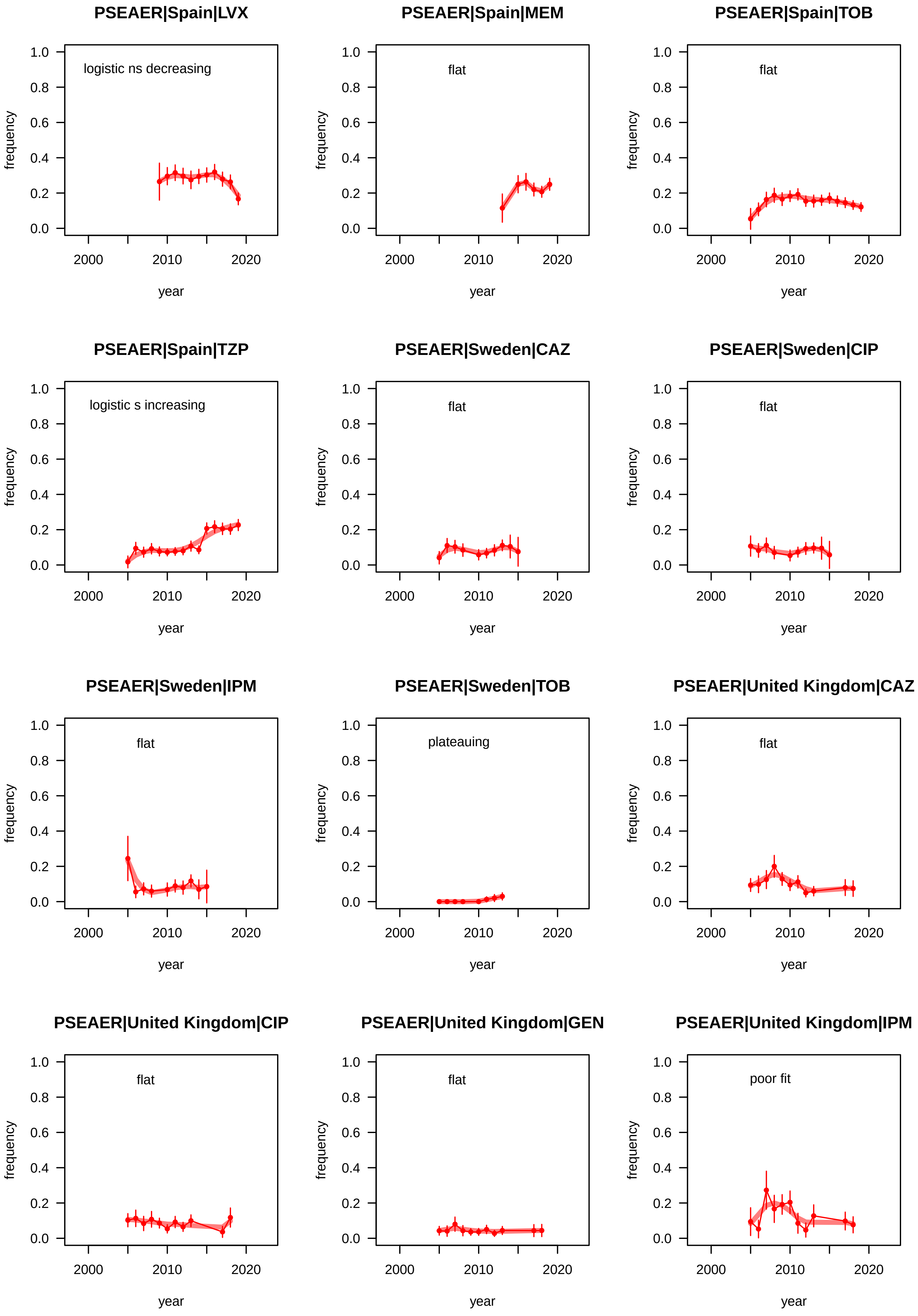
PSEAER|Spain|LVX
logistic ns decreasing
PSEAER|Spain|MEM
flat
PSEAER|Spain|TOB
flat
PSEAER|Spain|TZP
logistic s increasing
PSEAER|Sweden|CAZ
flat
PSEAER|Sweden|CIP
flat
PSEAER|Sweden|IPM
flat
PSEAER|Sweden|TOB
plateauing
PSEAER|United Kingdom|CAZ
flat
PSEAER|United Kingdom|CIP
flat
PSEAER|United Kingdom|GEN
flat
PSEAER|United Kingdom|IPM
poor fit
frequency
0.0
0.2
0.4
0.6
0.8
1.0
year
2000
2010
2020

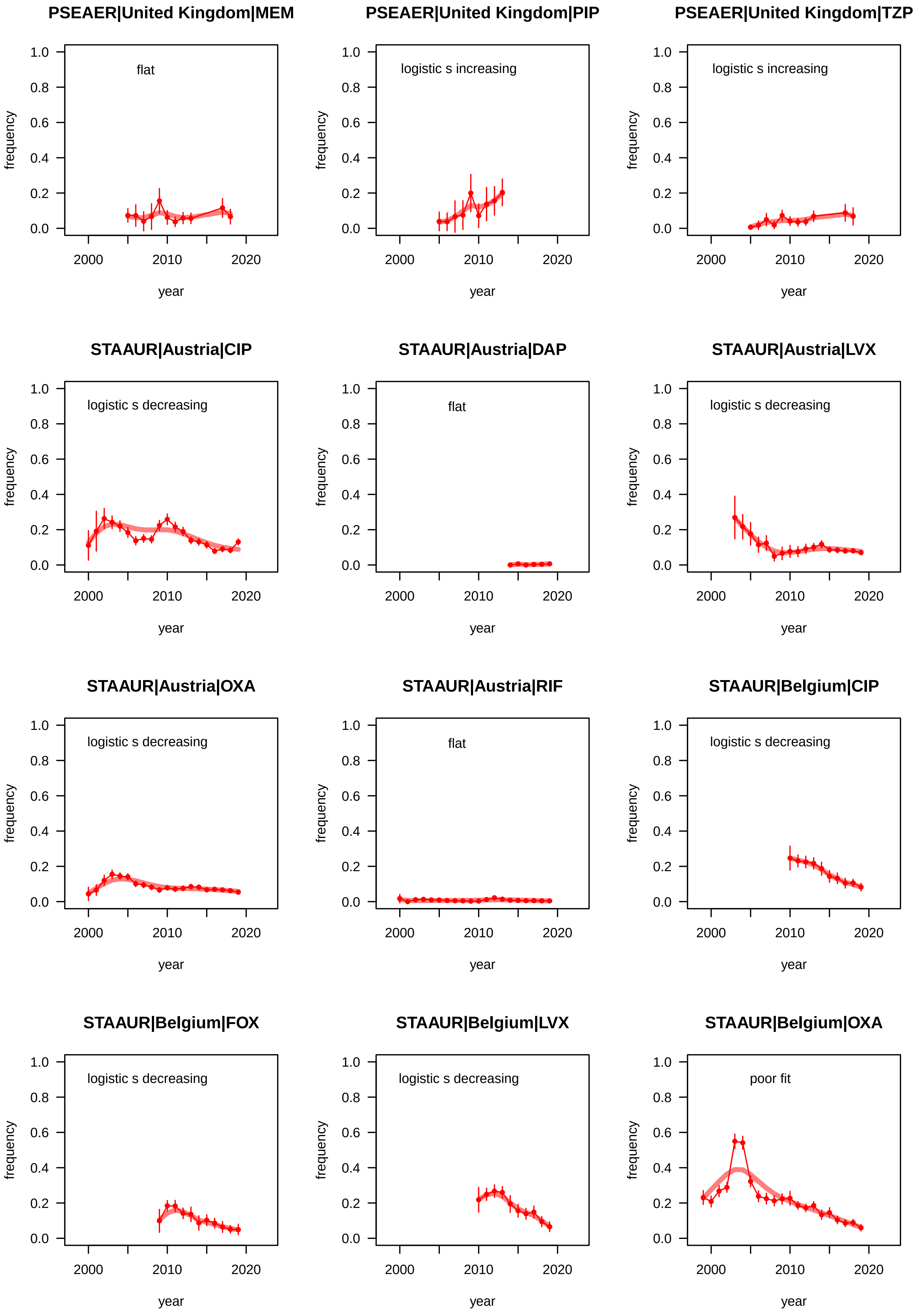
PSEAER|United Kingdom|MEM
flat
PSEAER|United Kingdom|PIP
logistic s increasing
PSEAER|United Kingdom|TZP
logistic s increasing
STAAUR|Austria|CIP
logistic s decreasing
STAAUR|Austria|DAP
flat
STAAUR|Austria|LVX
logistic s decreasing
STAAUR|Austria|OXA
logistic s decreasing
STAAUR|Austria|RIF
flat
STAAUR|Belgium|CIP
logistic s decreasing
STAAUR|Belgium|FOX
logistic s decreasing
STAAUR|Belgium|LVX
logistic s decreasing
STAAUR|Belgium|OXA
poor fit
frequency
year
0.0
0.2
0.4
0.6
0.8
1.0
2000
2010
2020

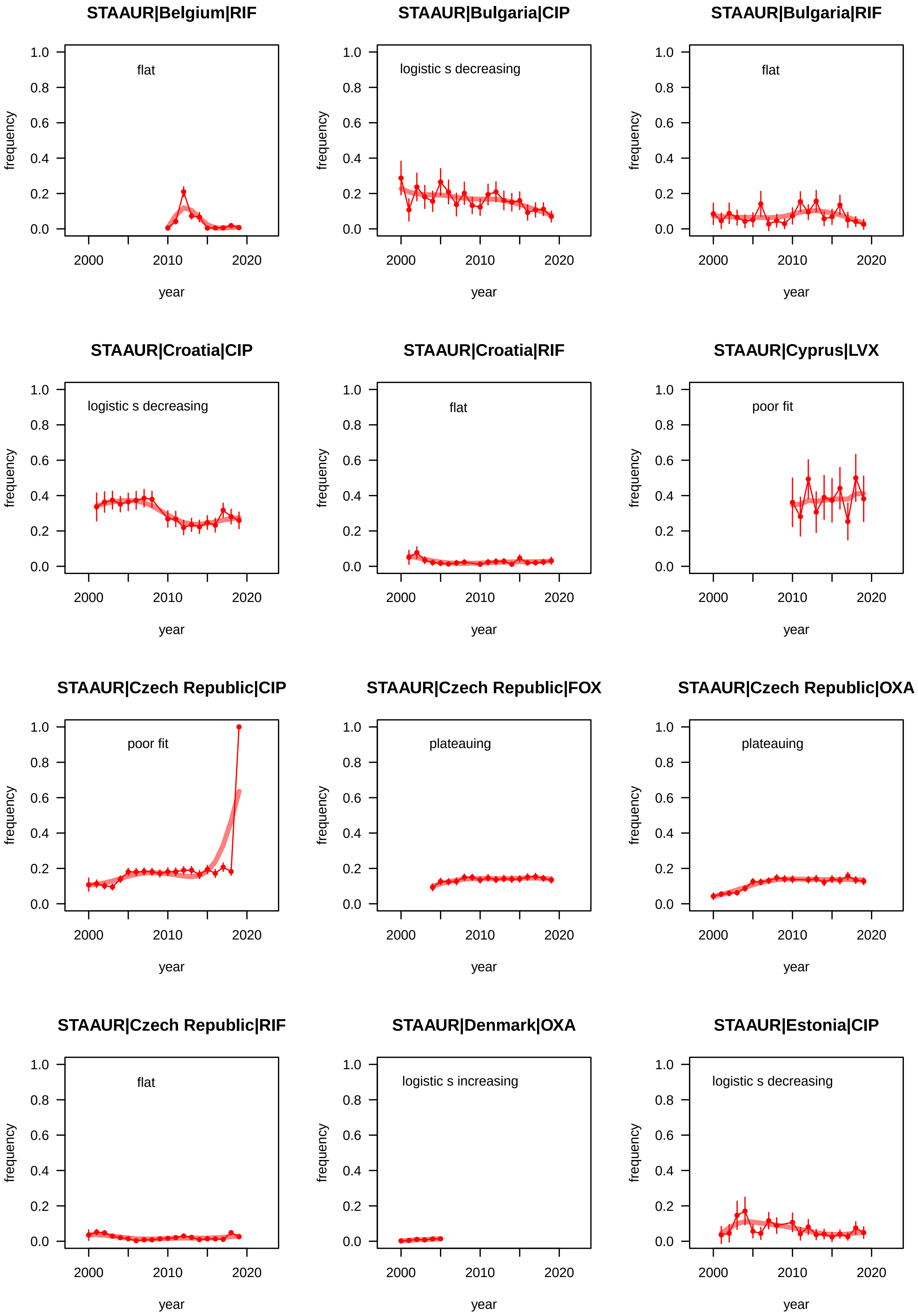
STAAUR|Belgium|RIF
flat
STAAUR|Bulgaria|CIP
logistic s decreasing
STAAUR|Bulgaria|RIF
flat
STAAUR|Croatia|CIP
logistic s decreasing
STAAUR|Croatia|RIF
flat
STAAUR|Cyprus|LVX
poor fit
STAAUR|Czech Republic|CIP
poor fit
STAAUR|Czech Republic|FOX
plateauing
STAAUR|Czech Republic|OXA
plateauing
STAAUR|Czech Republic|RIF
flat
STAAUR|Denmark|OXA
logistic s increasing
STAAUR|Estonia|CIP
logistic s decreasing
frequency
year

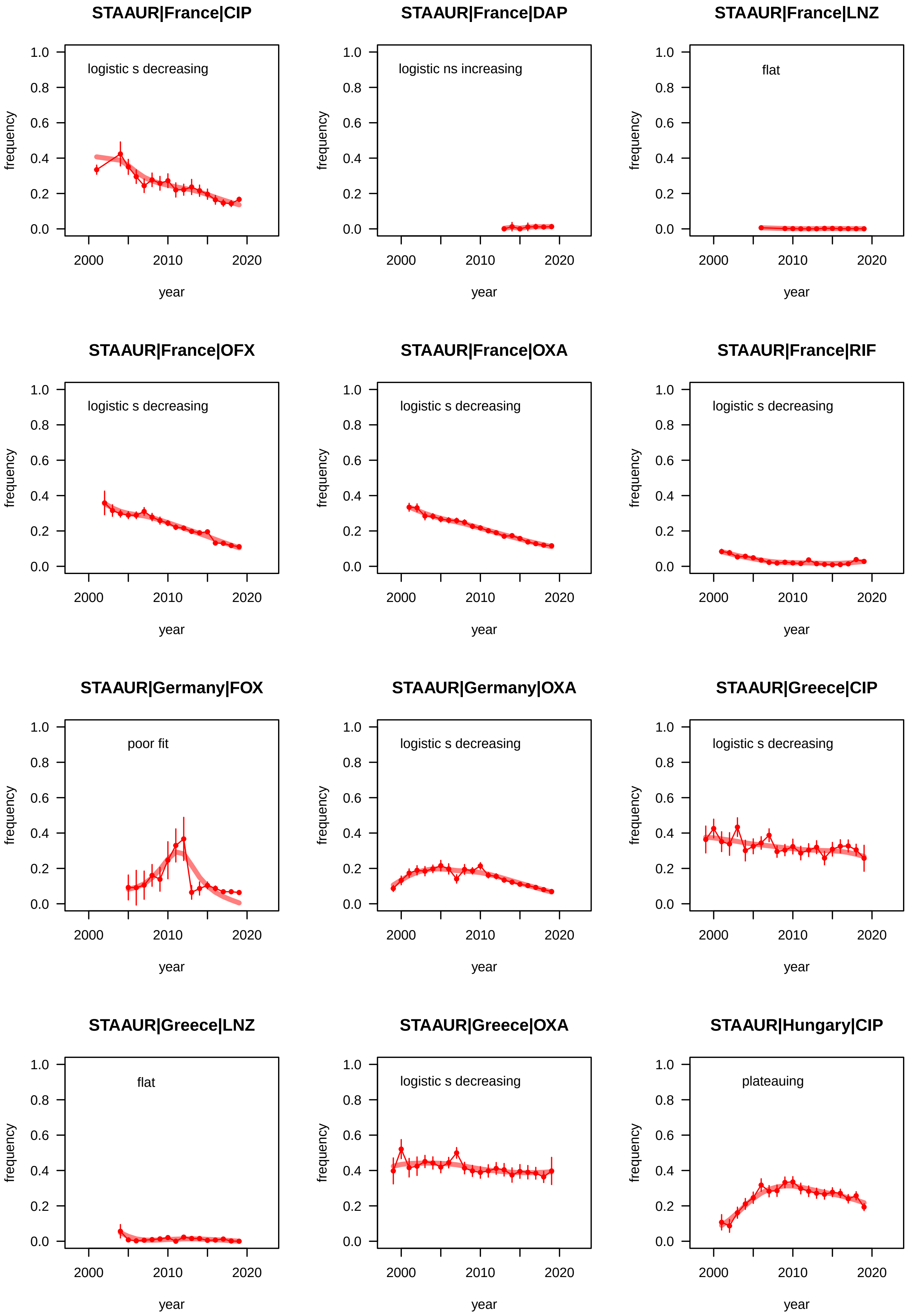
STAAUR|France|CIP
logistic s decreasing
STAAUR|France|DAP
logistic ns increasing
STAAUR|France|LNZ
flat
STAAUR|France|OFX
logistic s decreasing
STAAUR|France|OXA
logistic s decreasing
STAAUR|France|RIF
logistic s decreasing
STAAUR|Germany|FOX
poor fit
STAAUR|Germany|OXA
logistic s decreasing
STAAUR|Greece|CIP
logistic s decreasing
STAAUR|Greece|LNZ
flat
STAAUR|Greece|OXA
logistic s decreasing
STAAUR|Hungary|CIP
plateauing
frequency
year
0.0
0.2
0.4
0.6
0.8
1.0
2000
2010
2020

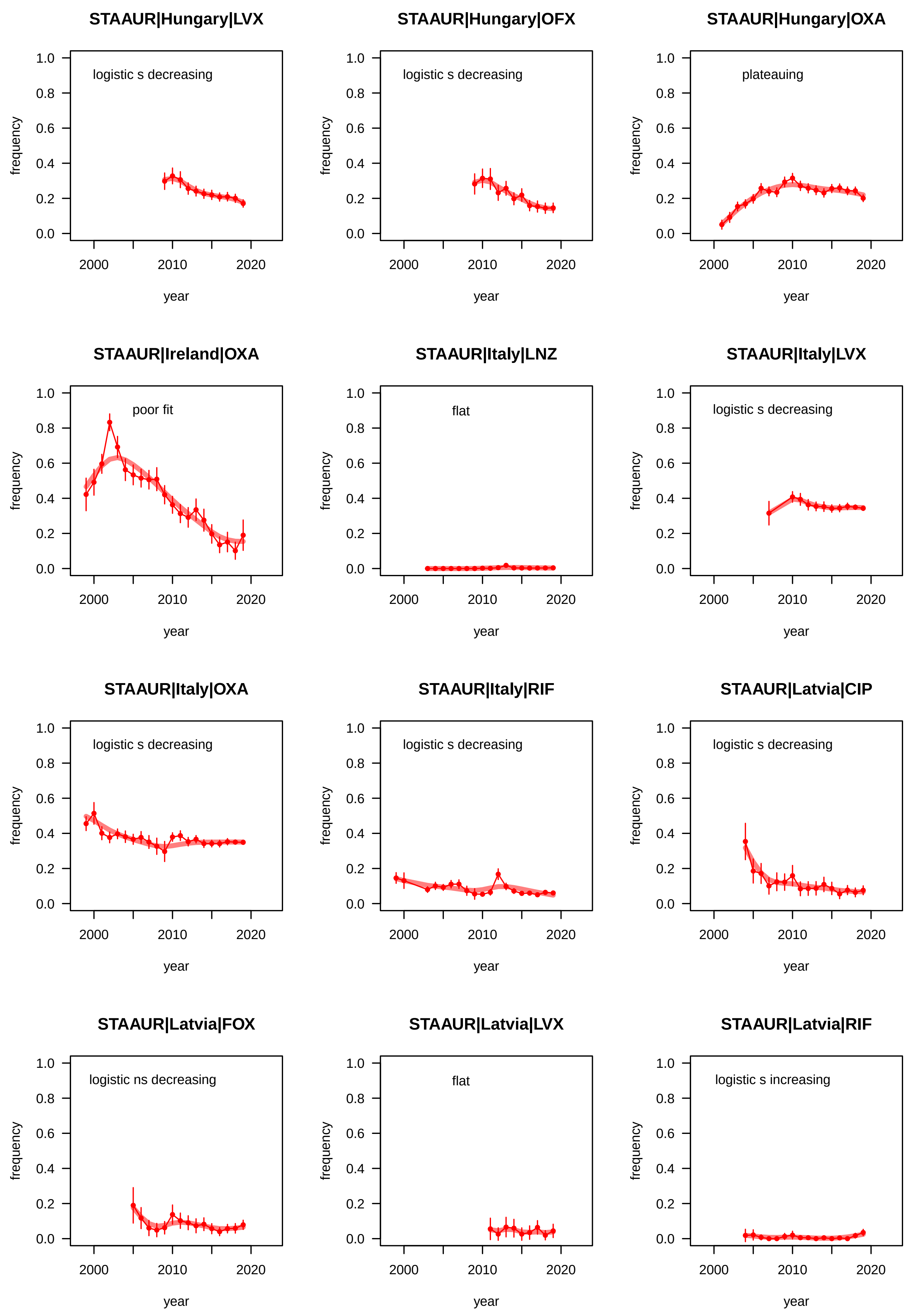
STAAUR|Hungary|LVX
logistic s decreasing
STAAUR|Hungary|OFX
logistic s decreasing
STAAUR|Hungary|OXA
plateauing
STAAUR|Ireland|OXA
poor fit
STAAUR|Italy|LNZ
flat
STAAUR|Italy|LVX
logistic s decreasing
STAAUR|Italy|OXA
logistic s decreasing
STAAUR|Italy|RIF
logistic s decreasing
STAAUR|Latvia|CIP
logistic s decreasing
STAAUR|Latvia|FOX
logistic ns decreasing
STAAUR|Latvia|LVX
flat
STAAUR|Latvia|RIF
logistic s increasing
frequency
year
0.0
0.2
0.4
0.6
0.8
1.0
2000
2010
2020

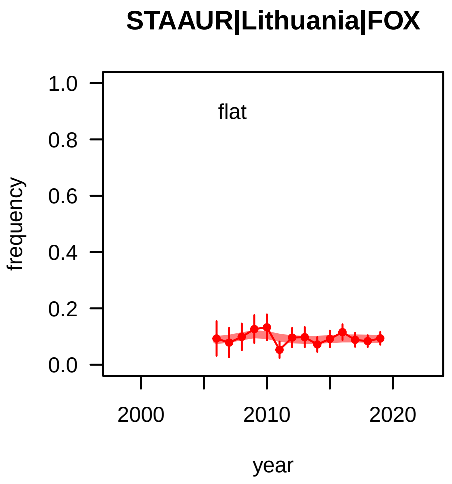
STAAUR|Lithuania|FOX
flat
frequency
year

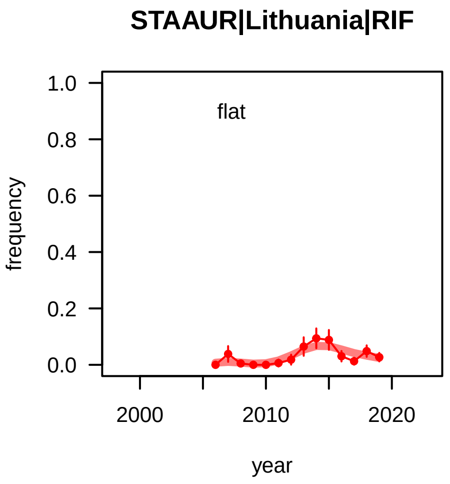
STAAUR|Lithuania|RIF
flat
frequency
year

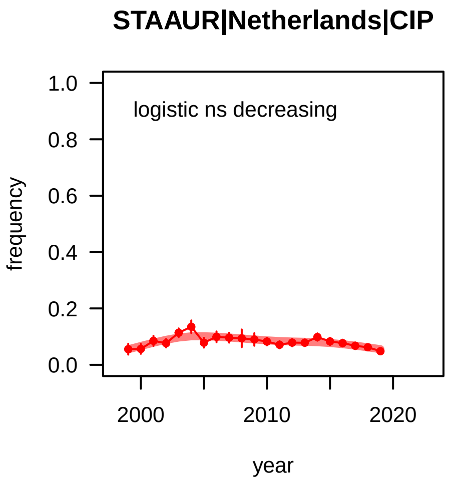
STAAUR|Netherlands|CIP
logistic ns decreasing
frequency
year

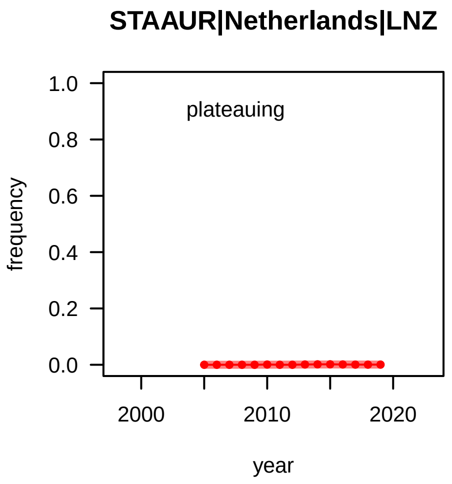
STAAUR|Netherlands|LNZ
plateauing
frequency
year

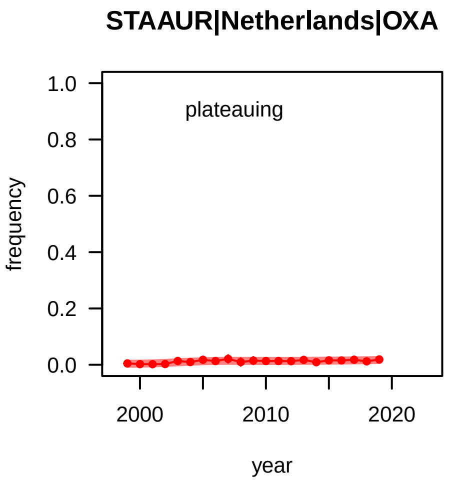
STAAUR|Netherlands|OXA
plateauing
frequency
year

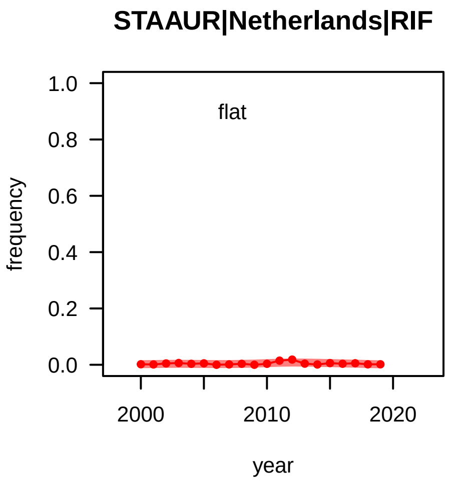
STAAUR|Netherlands|RIF
flat
frequency
year

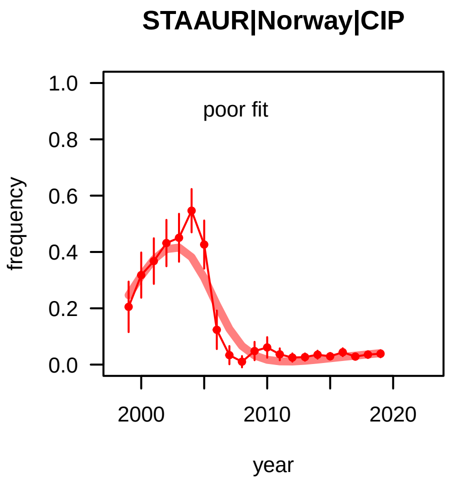
STAAUR|Norway|CIP
poor fit
frequency
year

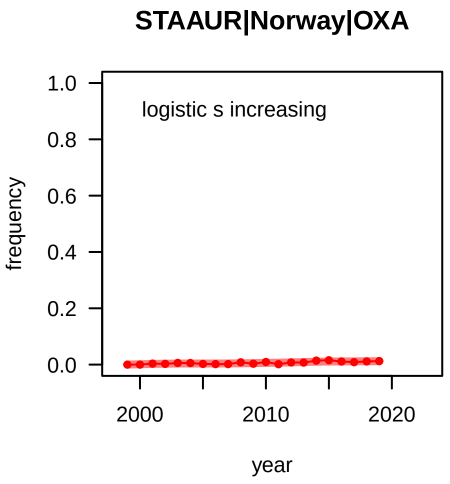
STAAUR|Norway|OXA
logistic s increasing
frequency
year

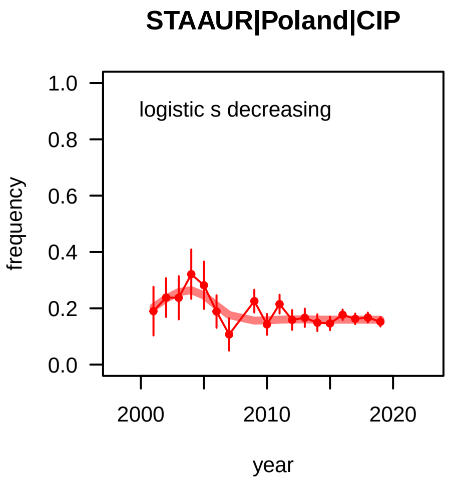
STAAUR|Poland|CIP
logistic s decreasing
frequency
year

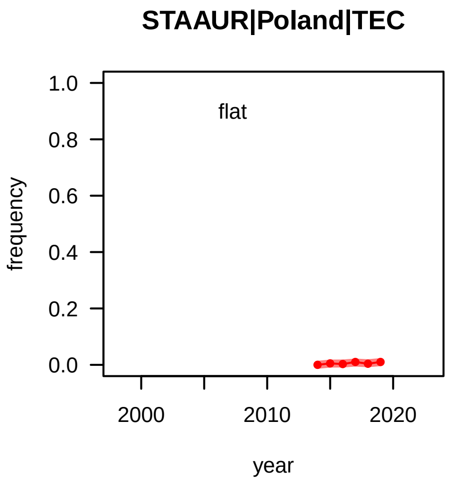
STAAUR|Poland|TEC
flat
frequency
year

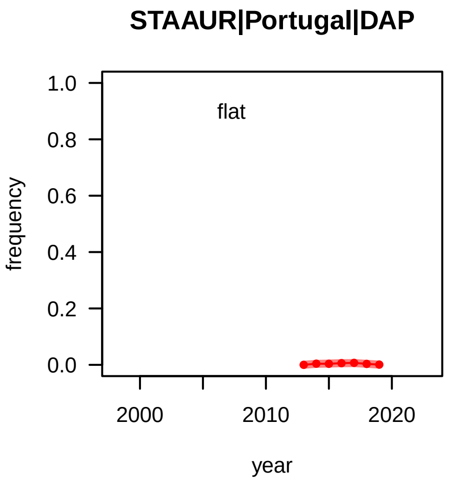
STAAUR|Portugal|DAP
flat
frequency
year

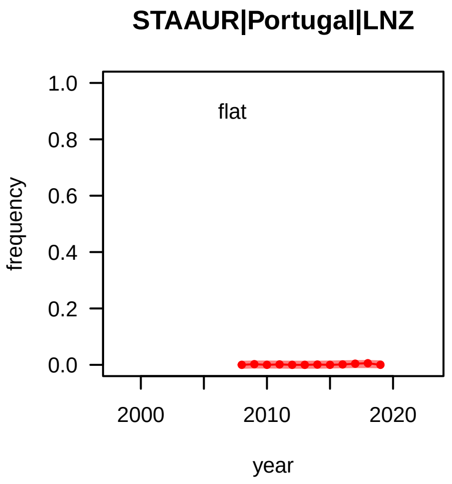
STAAUR|Portugal|LNZ
flat
frequency
year

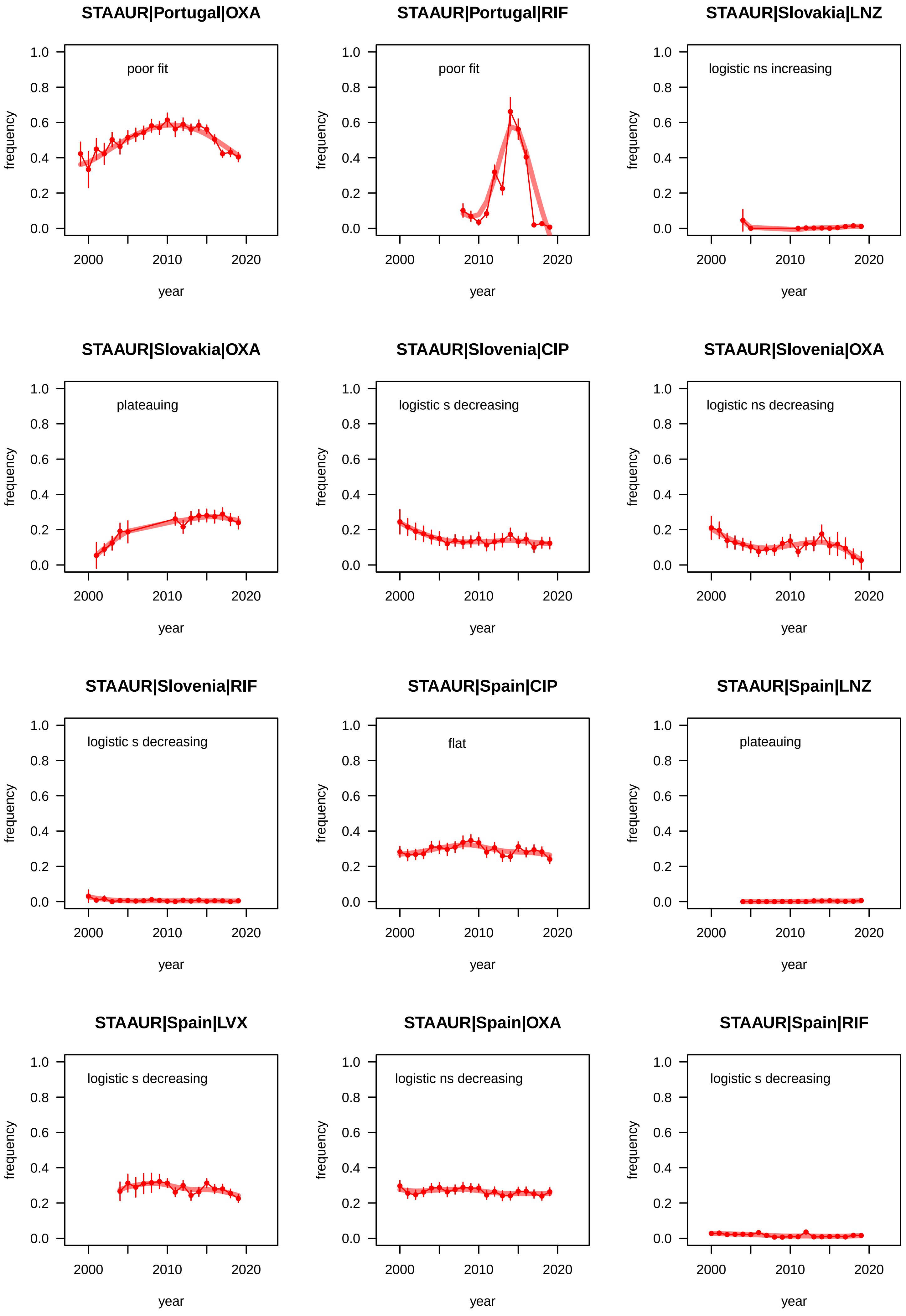
STAAUR|Portugal|OXA
poor fit
STAAUR|Portugal|RIF
poor fit
STAAUR|Slovakia|LNZ
logistic ns increasing
STAAUR|Slovakia|OXA
plateauing
STAAUR|Slovenia|CIP
logistic s decreasing
STAAUR|Slovenia|OXA
logistic ns decreasing
STAAUR|Slovenia|RIF
logistic s decreasing
STAAUR|Spain|CIP
flat
STAAUR|Spain|LNZ
plateauing
STAAUR|Spain|LVX
logistic s decreasing
STAAUR|Spain|OXA
logistic ns decreasing
STAAUR|Spain|RIF
logistic s decreasing
frequency
year
0.0
0.2
0.4
0.6
0.8
1.0
2000
2010
2020

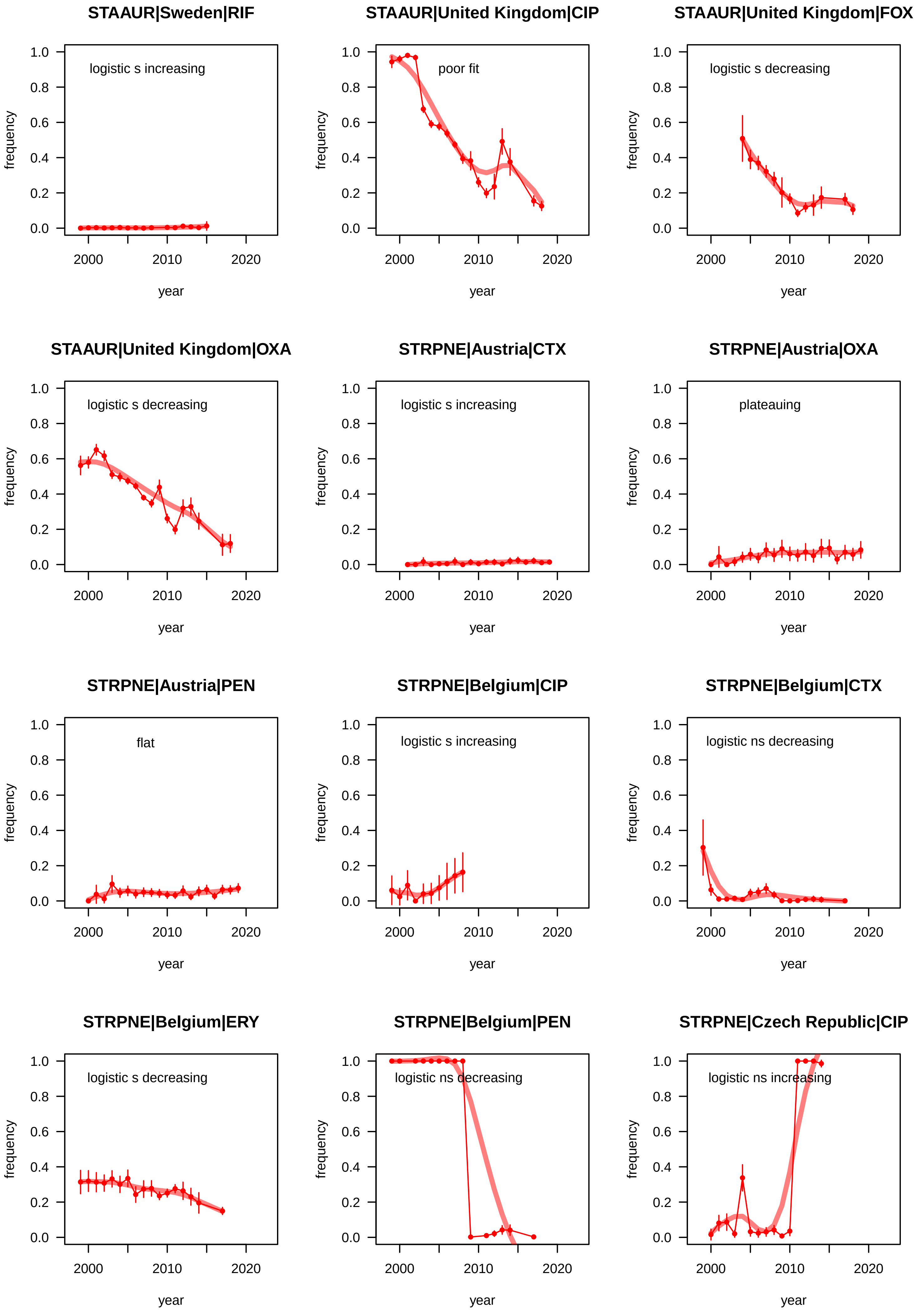
STAAUR|Sweden|RIF
logistic s increasing
STAAUR|United Kingdom|CIP
poor fit
STAAUR|United Kingdom|FOX
logistic s decreasing
STAAUR|United Kingdom|OXA
logistic s decreasing
STRPNE|Austria|CTX
logistic s increasing
STRPNE|Austria|OXA
plateauing
STRPNE|Austria|PEN
flat
STRPNE|Belgium|CIP
logistic s increasing
STRPNE|Belgium|CTX
logistic ns decreasing
STRPNE|Belgium|ERY
logistic s decreasing
STRPNE|Belgium|PEN
logistic ns decreasing
STRPNE|Czech Republic|CIP
logistic ns increasing
frequency
0.0
0.2
0.4
0.6
0.8
1.0
year
2000
2010
2020

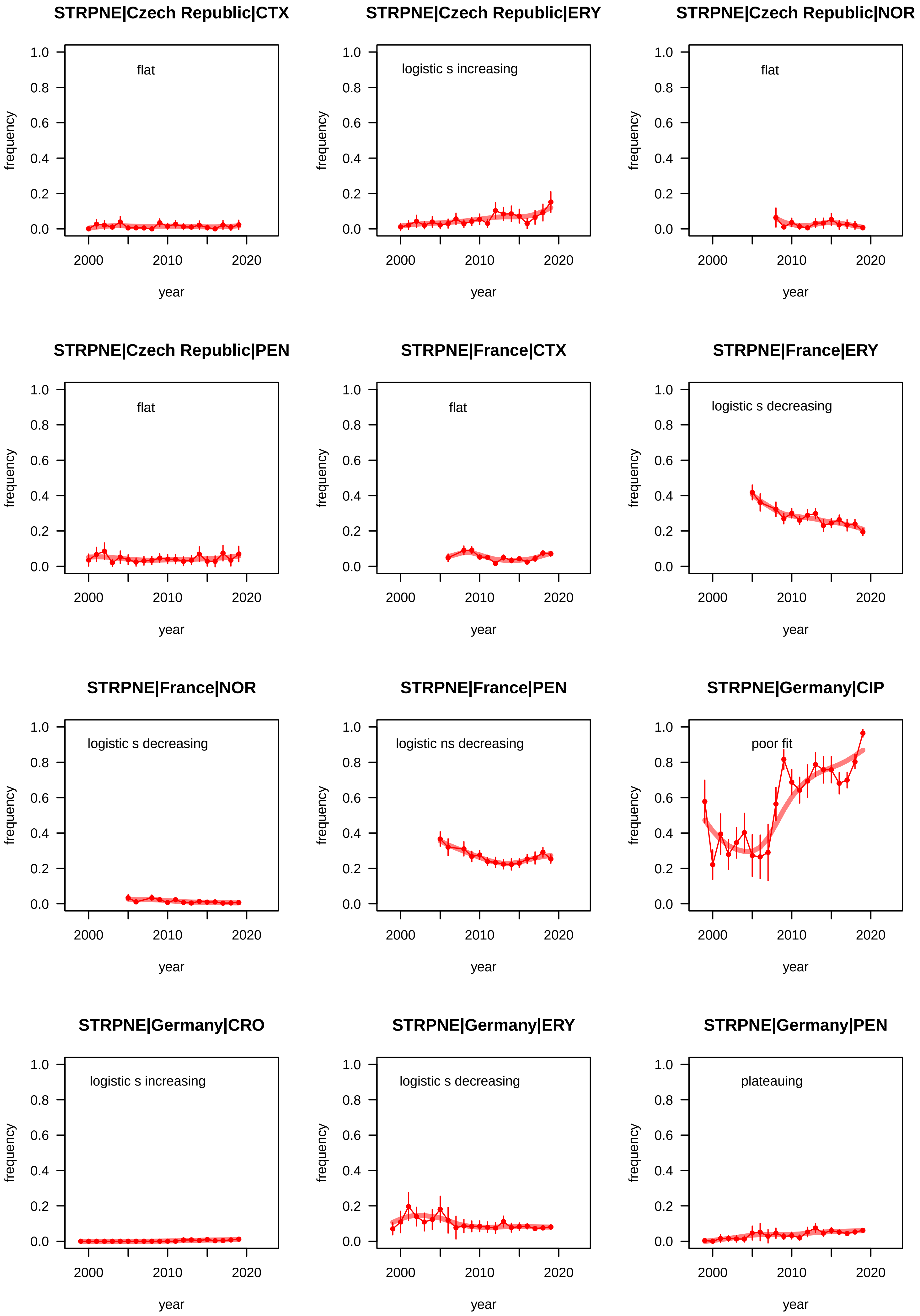
STRPNE|Czech Republic|CTX
flat
STRPNE|Czech Republic|ERY
logistic s increasing
STRPNE|Czech Republic|NOR
flat
STRPNE|Czech Republic|PEN
flat
STRPNE|France|CTX
flat
STRPNE|France|ERY
logistic s decreasing
STRPNE|France|NOR
logistic s decreasing
STRPNE|France|PEN
logistic ns decreasing
STRPNE|Germany|CIP
poor fit
STRPNE|Germany|CRO
logistic s increasing
STRPNE|Germany|ERY
logistic s decreasing
STRPNE|Germany|PEN
plateauing
frequency
year
0.0
0.2
0.4
0.6
0.8
1.0
2000
2010
2020

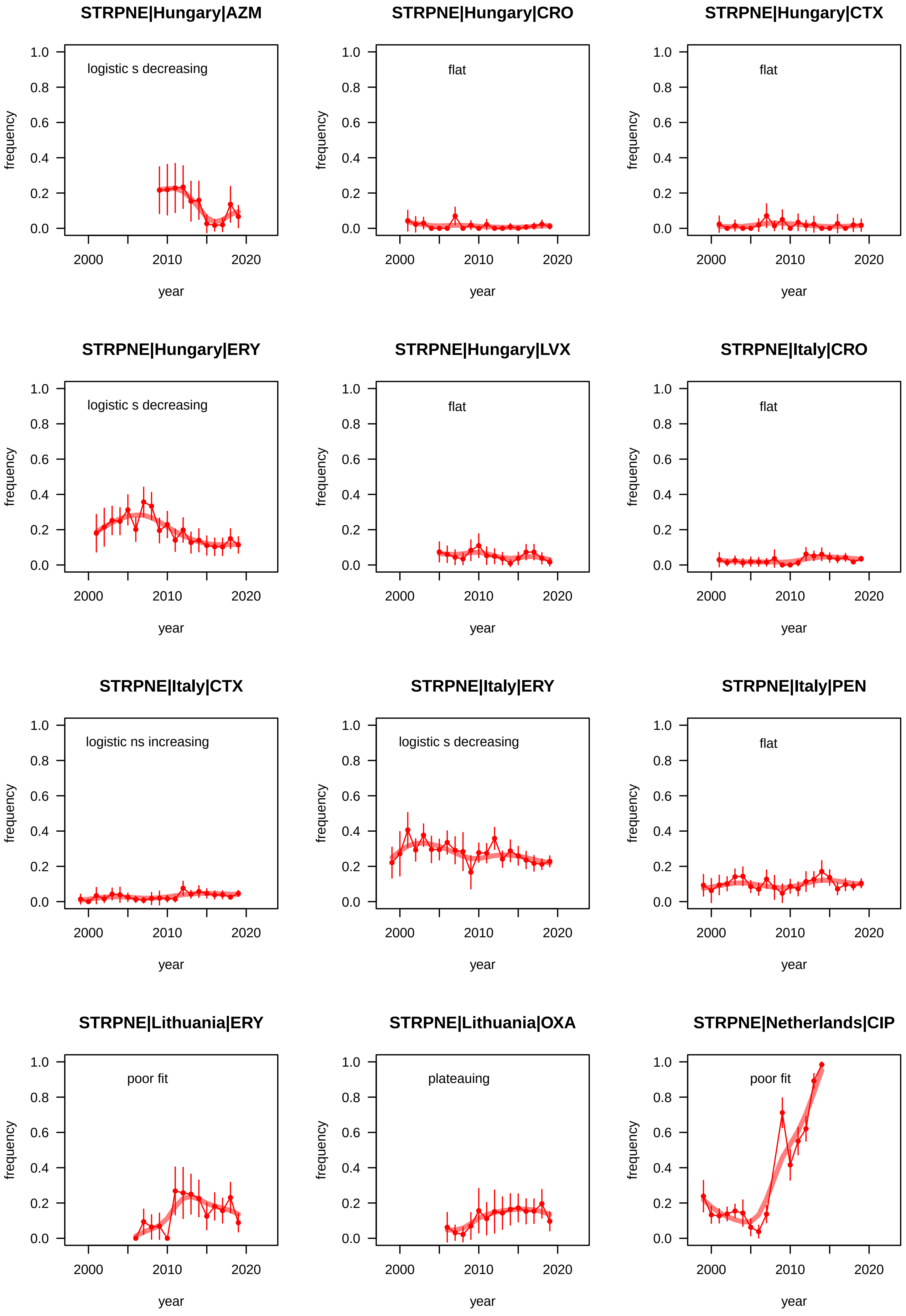

STRPNE|Hungary|AZM
logistic s decreasing
STRPNE|Hungary|CRO
flat
STRPNE|Hungary|CTX
flat
STRPNE|Hungary|ERY
logistic s decreasing
STRPNE|Hungary|LVX
flat
STRPNE|Italy|CRO
flat
STRPNE|Italy|CTX
logistic ns increasing
STRPNE|Italy|ERY
logistic s decreasing
STRPNE|Italy|PEN
flat
STRPNE|Lithuania|ERY
poor fit
STRPNE|Lithuania|OXA
plateauing
STRPNE|Netherlands|CIP
poor fit
frequency
year
1.0
0.8
0.6
0.4
0.2
0.0
2000
2010
2020

STRPNE|Netherlands|CLR

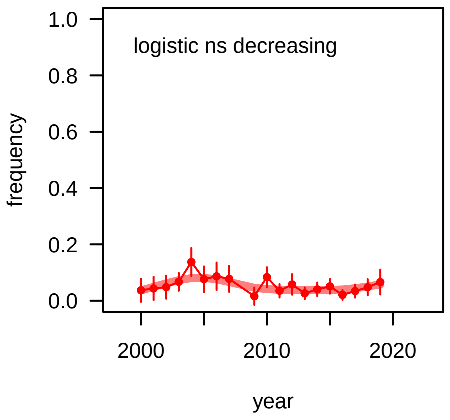

STRPNE|Netherlands|ERY

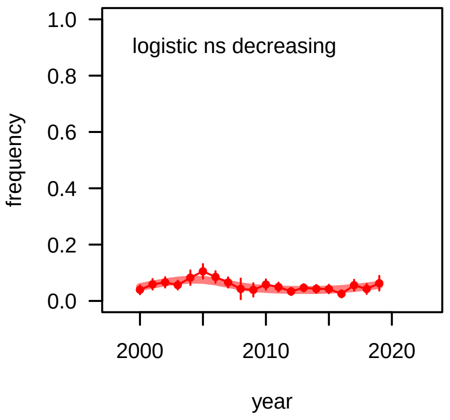

STRPNE|Netherlands|OXA

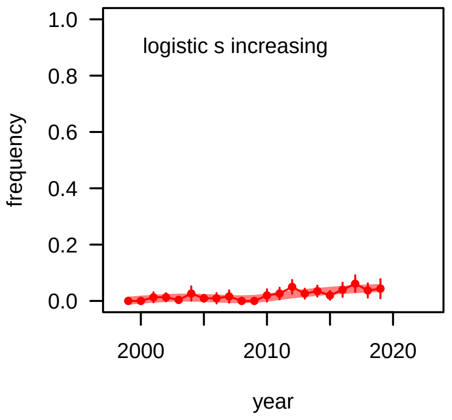

STRPNE|Netherlands|PEN

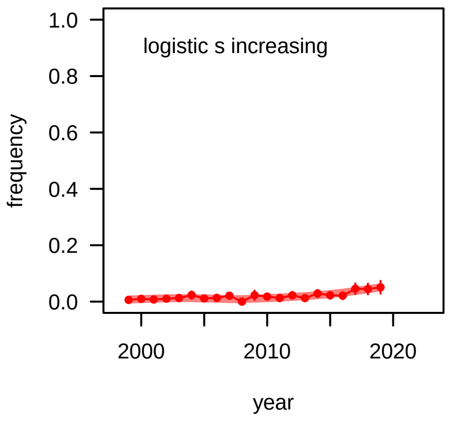

STRPNE|Norway|CTX

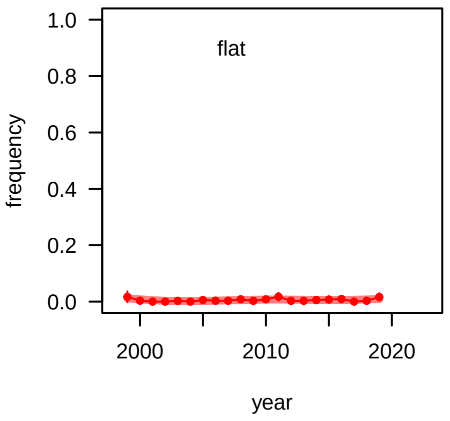

STRPNE|Norway|ERY

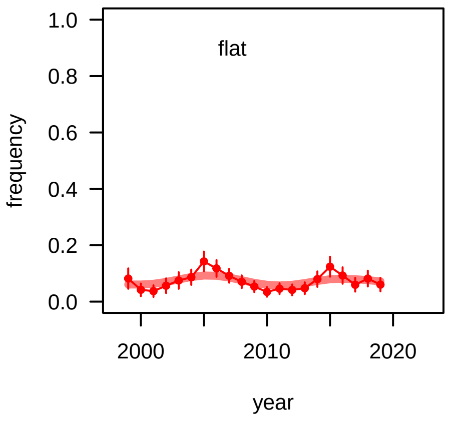

STRPNE|Norway|PEN

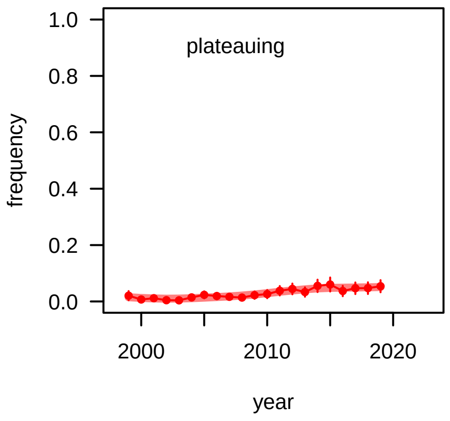

STRPNE|Slovenia|CTX

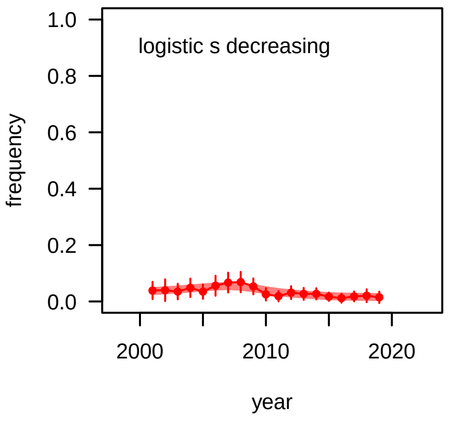

STRPNE|Slovenia|PEN

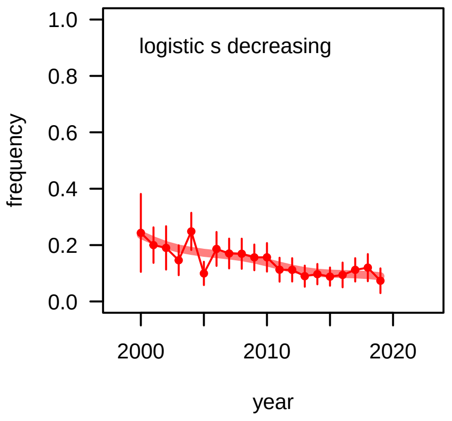

STRPNE|United Kingdom|ERY

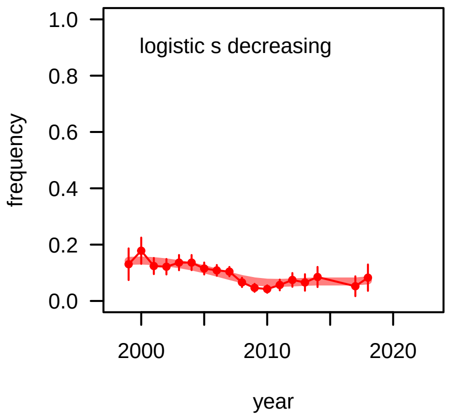

STRPNE|United Kingdom|PEN

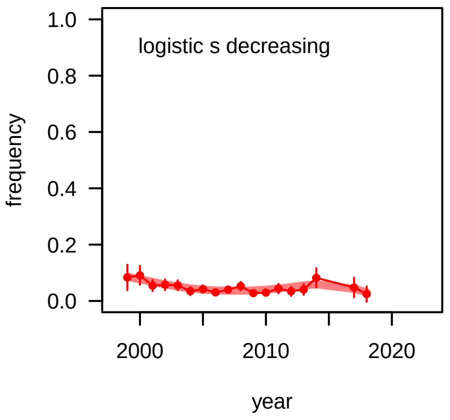

Supplement: S1 Appendix — Antibiotic resistance trajectories for all bug-drug-country combinations. The plots show resistance frequency against calendar year for all bug-drug-country combinations analysed in the paper. The error bars represent 95% confidence intervals. The transparent line shows a smoothing function – not any of the models fitted in the paper. The text within each plot indicates how the trajectory was categorised. (PDF) [file ppat.1012945.s002.pdf]
