# Supplementary material for: The evolution of antibiotic resistance in Europe, 1998–2019
Source: PLoS Pathog. 2025 Apr 3;21(4):e1012945. doi: 10.1371/journal.ppat.1012945 (PMC11967945; doi:10.1371/journal.ppat.1012945)
Supplement: S2 Appendix — Antibiotic consumption trajectories for all drug-country combinations. The plots show antibiotic consumption, as measured in defined daily doses (DDDs), against calendar year for all drug-country combinations. The dark green line shows mean consumption, the light green line median consumption (before removal of outliers). Red points indicate outliers, defined as not within 3x the inter-quartile range. These were removed for the analyses in the paper. (PDF) [file ppat.1012945.s003.pdf]

Appendix B for *Temporal Trends in Antibiotic Resistance in Europe, 1998-2019*

Antibiotic consumption trajectories for all drug-country combinations

The plots show antibiotic consumption, as measured in defined daily doses (DDDs), against calendar year for all drug-country combinations. The dark green line shows mean consumption, the light green line median consumption (before removal of outliers). Red points indicate outliers, defined as not within 3x the inter-quartile range. These were removed for the analyses in the paper.

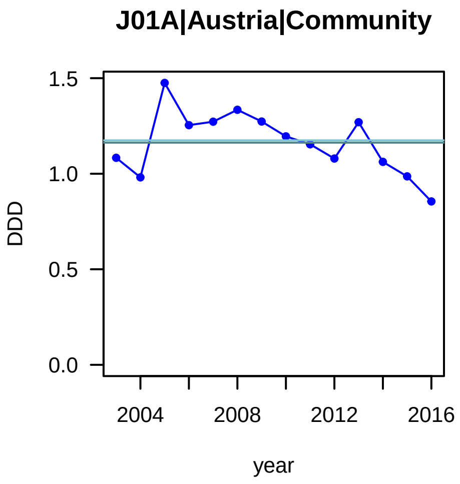
J01A|Austria|Community
DDD
1.5
1.0
0.5
0.0
2004
2008
2012
2016
year

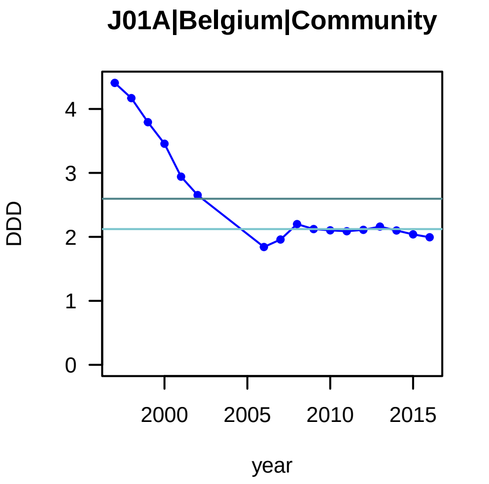
J01A|Belgium|Community
DDD
4
3
2
1
0
2000
2005
2010
2015
year

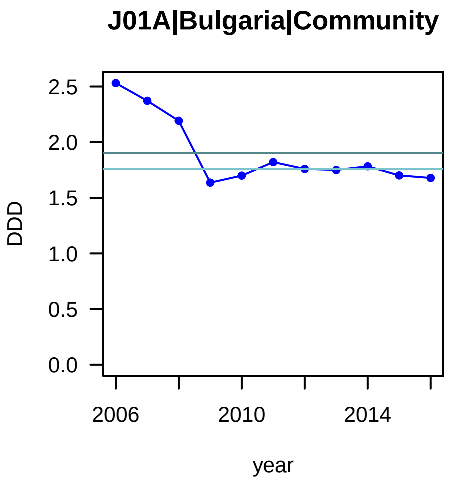
J01A|Bulgaria|Community
DDD
2.5
2.0
1.5
1.0
0.5
0.0
2006
2010
2014
year

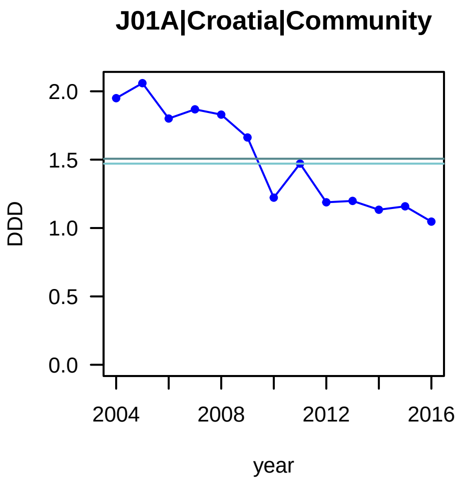
J01A|Croatia|Community
DDD
2.0
1.5
1.0
0.5
0.0
2004
2008
2012
2016
year

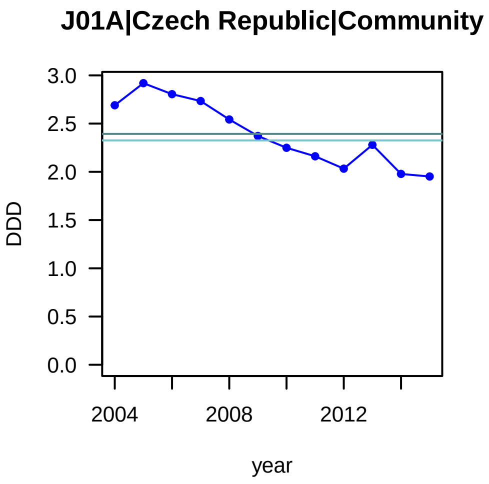
J01A|Czech Republic|Community
DDD
3.0
2.5
2.0
1.5
1.0
0.5
0.0
2004
2008
2012
year

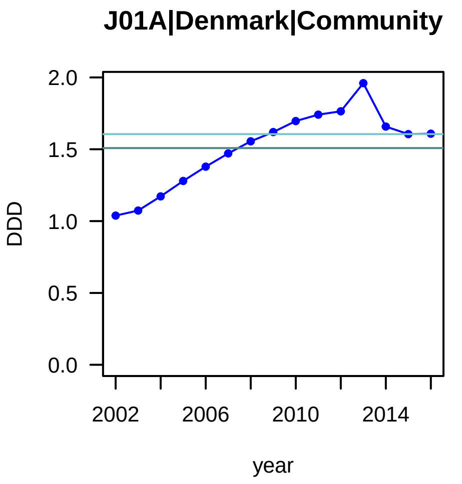
J01A|Denmark|Community
DDD
2.0
1.5
1.0
0.5
0.0
2002
2006
2010
2014
year

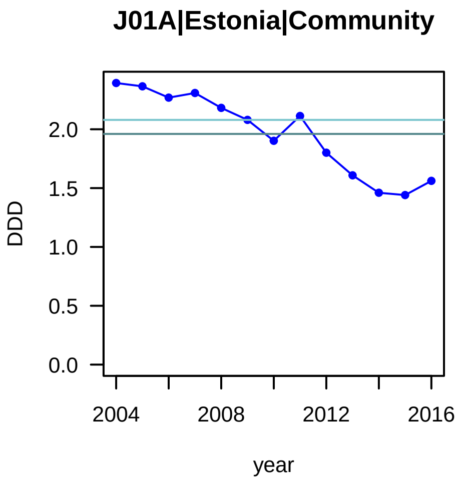
J01A|Estonia|Community
DDD
2.0
1.5
1.0
0.5
0.0
2004
2008
2012
2016
year

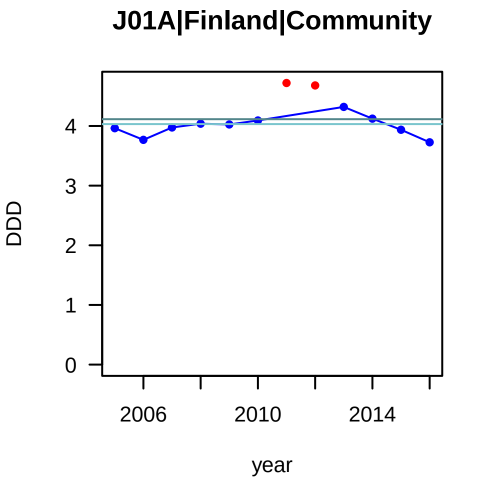
J01A|Finland|Community
DDD
4
3
2
1
0
2006
2010
2014
year

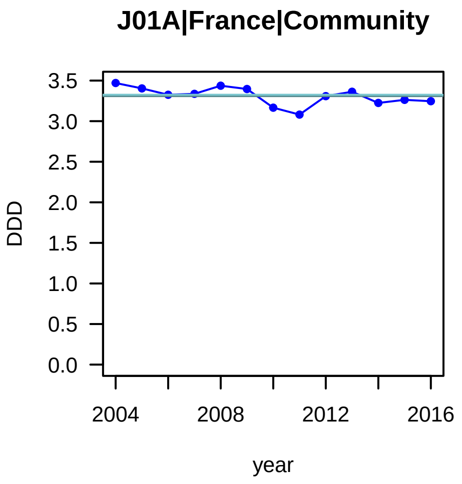
J01A|France|Community
DDD
3.5
3.0
2.5
2.0
1.5
1.0
0.5
0.0
2004
2008
2012
2016
year

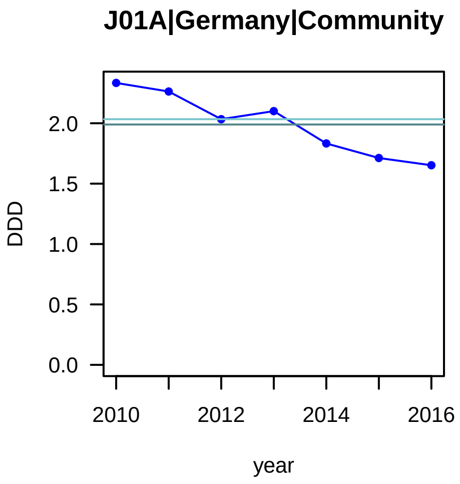
J01A|Germany|Community
DDD
2.0
1.5
1.0
0.5
0.0
2010
2012
2014
2016
year

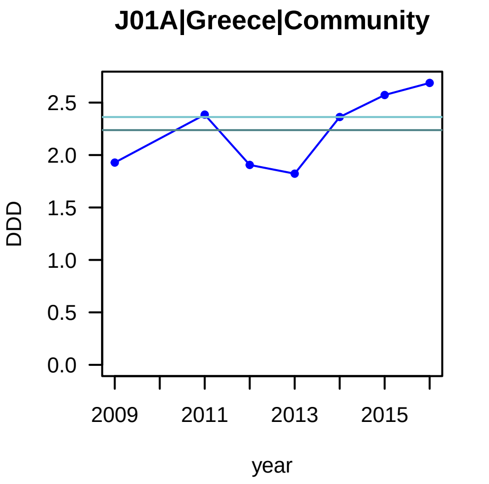
J01A|Greece|Community
DDD
2.5
2.0
1.5
1.0
0.5
0.0
2009
2011
2013
2015
year

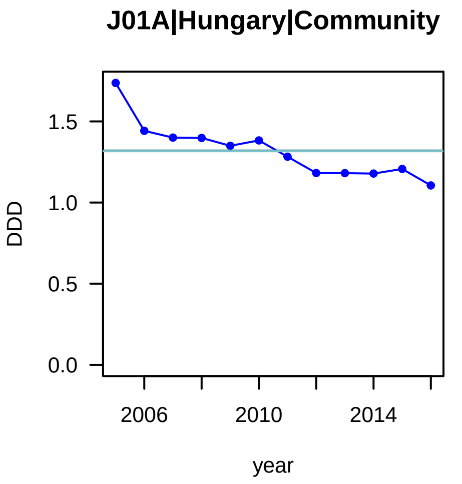
J01A|Hungary|Community
DDD
1.5
1.0
0.5
0.0
2006
2010
2014
year

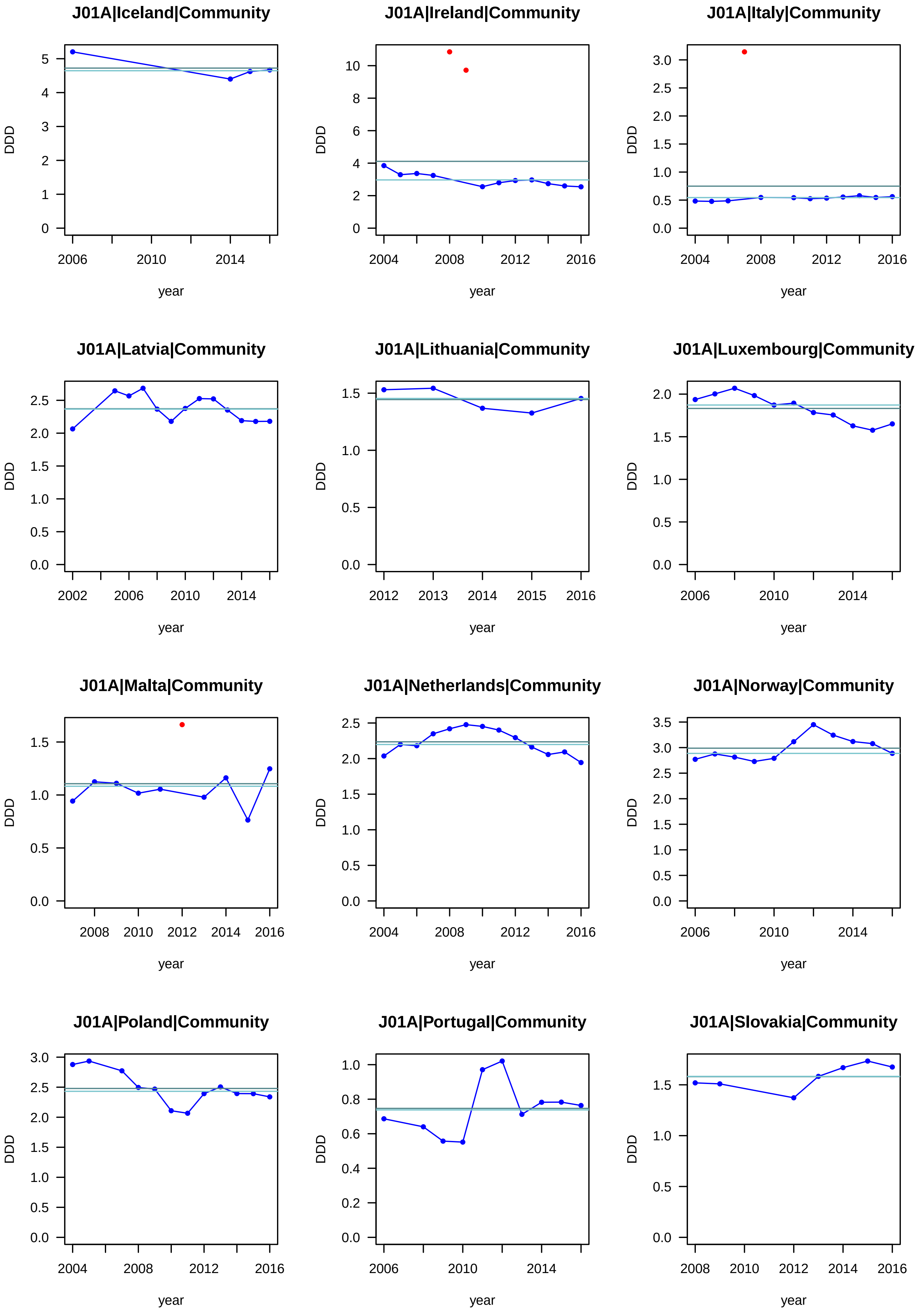
J01A|Iceland|Community
J01A|Ireland|Community
J01A|Italy|Community
J01A|Latvia|Community
J01A|Lithuania|Community
J01A|Luxembourg|Community
J01A|Malta|Community
J01A|Netherlands|Community
J01A|Norway|Community
J01A|Poland|Community
J01A|Portugal|Community
J01A|Slovakia|Community
DDD
year

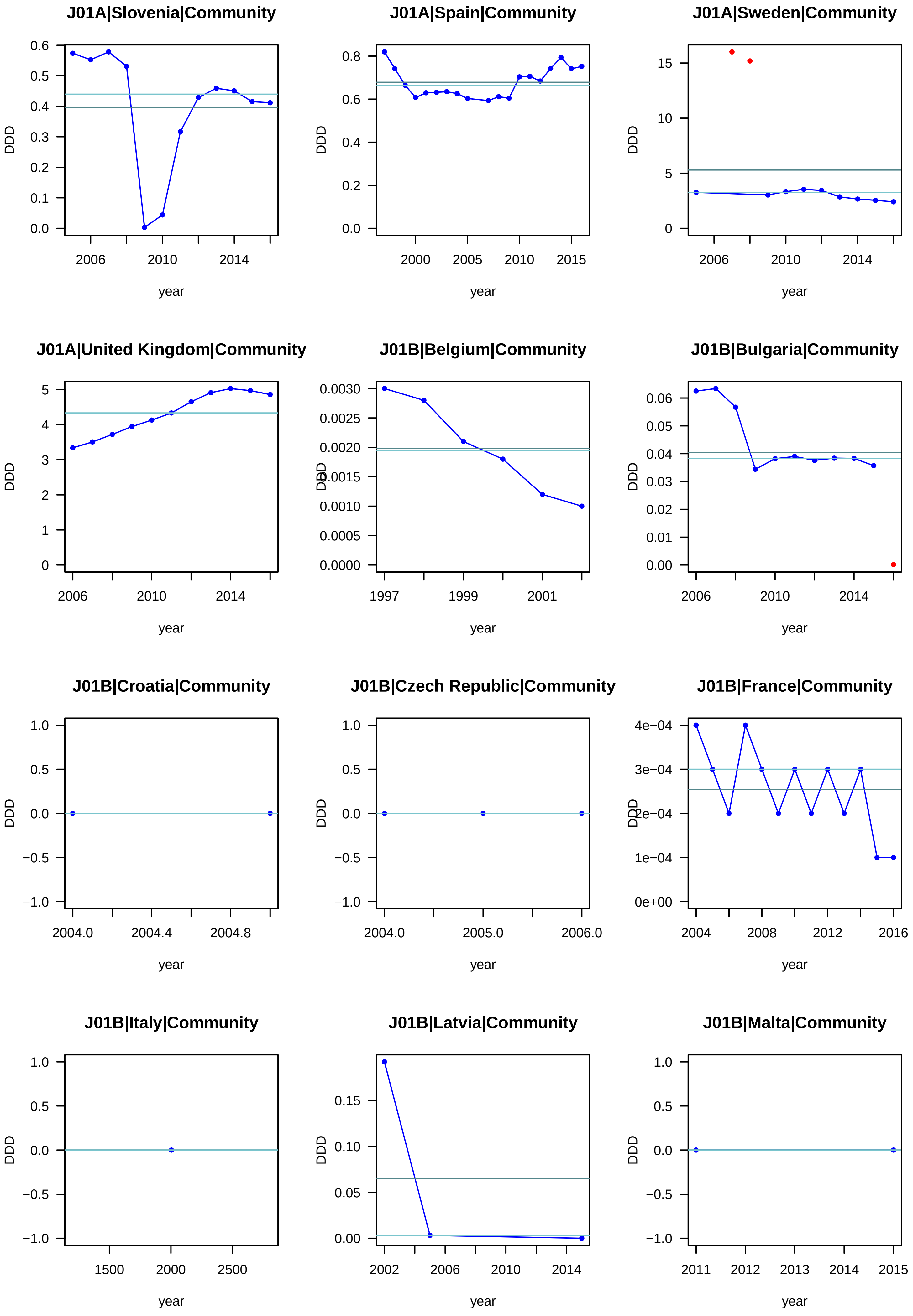
J01A|Slovenia|Community
J01A|Spain|Community
J01A|Sweden|Community
J01A|United Kingdom|Community
J01B|Belgium|Community
J01B|Bulgaria|Community
J01B|Croatia|Community
J01B|Czech Republic|Community
J01B|France|Community
J01B|Italy|Community
J01B|Latvia|Community
J01B|Malta|Community
DDD
year

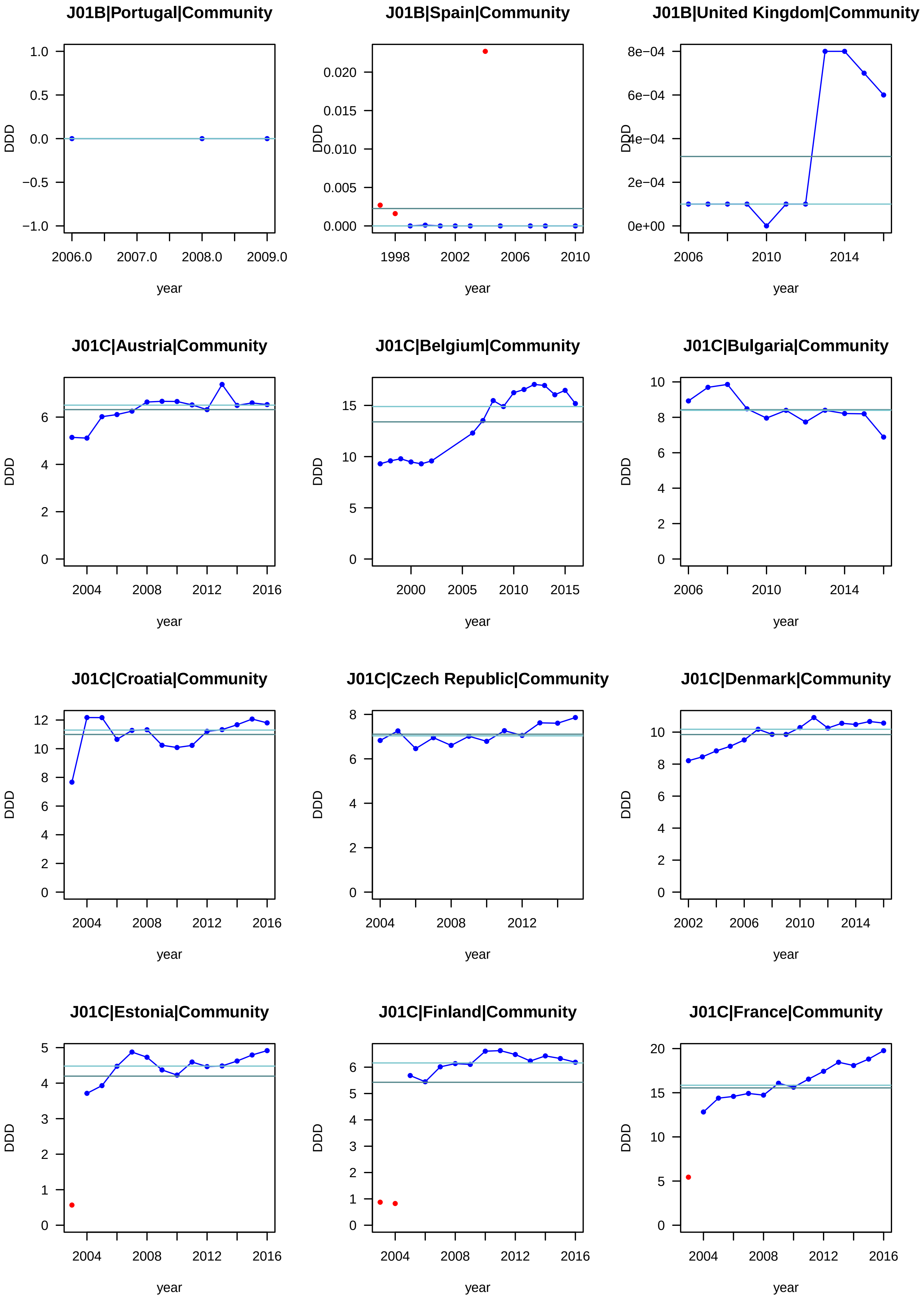
J01B|Portugal|Community
DDD
1.0
0.5
0.0
−0.5
−1.0
2006.0
2007.0
2008.0
2009.0
year
J01B|Spain|Community
DDD
0.020
0.015
0.010
0.005
0.000
1998
2002
2006
2010
year
J01B|United Kingdom|Community
DDD
8e−04
6e−04
4e−04
2e−04
0e+00
2006
2010
2014
year
J01C|Austria|Community
DDD
6
4
2
0
2004
2008
2012
2016
year
J01C|Belgium|Community
DDD
15
10
5
0
2000
2005
2010
2015
year
J01C|Bulgaria|Community
DDD
10
8
6
4
2
0
2006
2010
2014
year
J01C|Croatia|Community
DDD
12
10
8
6
4
2
0
2004
2008
2012
2016
year
J01C|Czech Republic|Community
DDD
8
6
4
2
0
2004
2008
2012
year
J01C|Denmark|Community
DDD
10
8
6
4
2
0
2002
2006
2010
2014
year
J01C|Estonia|Community
DDD
5
4
3
2
1
0
2004
2008
2012
2016
year
J01C|Finland|Community
DDD
6
5
4
3
2
1
0
2004
2008
2012
2016
year
J01C|France|Community
DDD
20
15
10
5
0
2004
2008
2012
2016
year

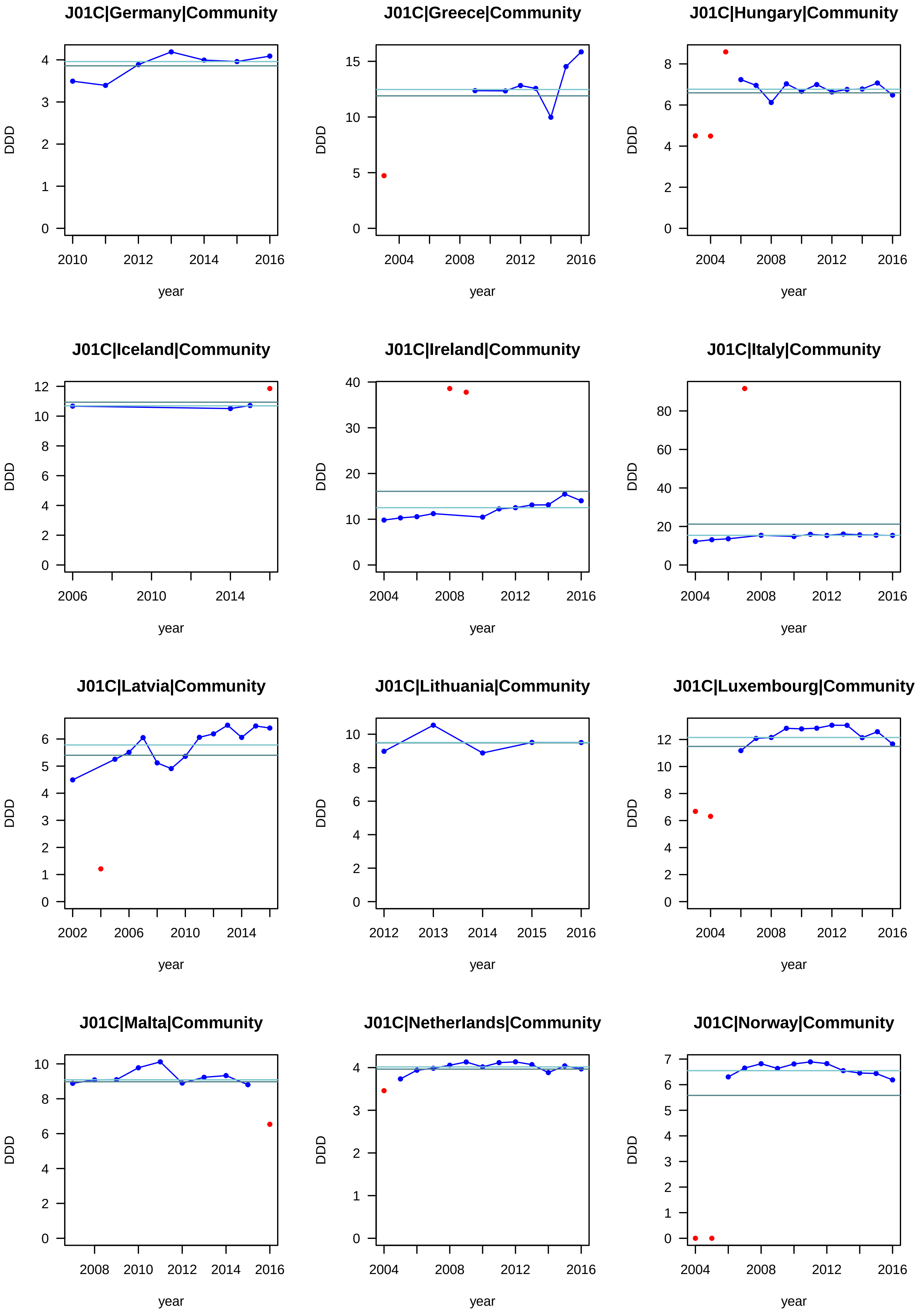
J01C|Germany|Community
DDD
year
J01C|Greece|Community
DDD
year
J01C|Hungary|Community
DDD
year
J01C|Iceland|Community
DDD
year
J01C|Ireland|Community
DDD
year
J01C|Italy|Community
DDD
year
J01C|Latvia|Community
DDD
year
J01C|Lithuania|Community
DDD
year
J01C|Luxembourg|Community
DDD
year
J01C|Malta|Community
DDD
year
J01C|Netherlands|Community
DDD
year
J01C|Norway|Community
DDD
year

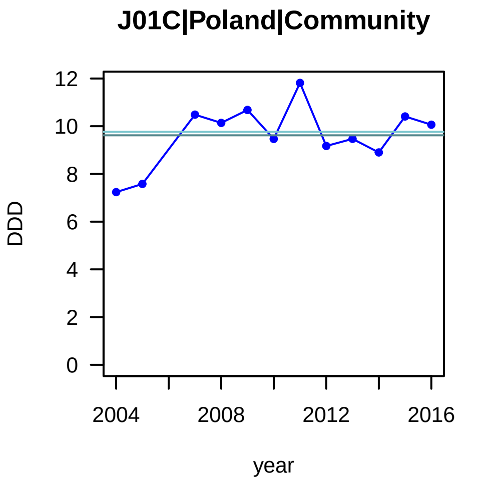
J01C|Poland|Community
DDD
0
2
4
6
8
10
12
2004
2008
2012
2016
year

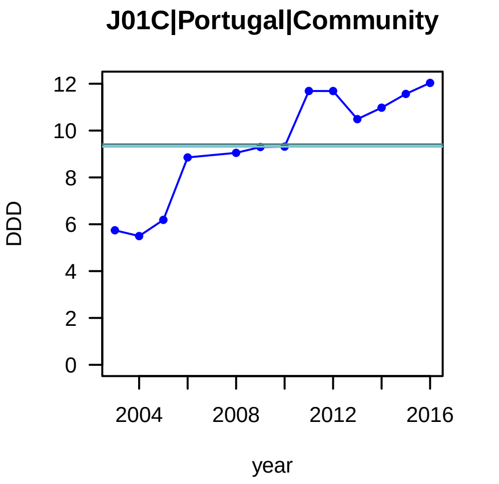
J01C|Portugal|Community
DDD
0
2
4
6
8
10
12
2004
2008
2012
2016
year

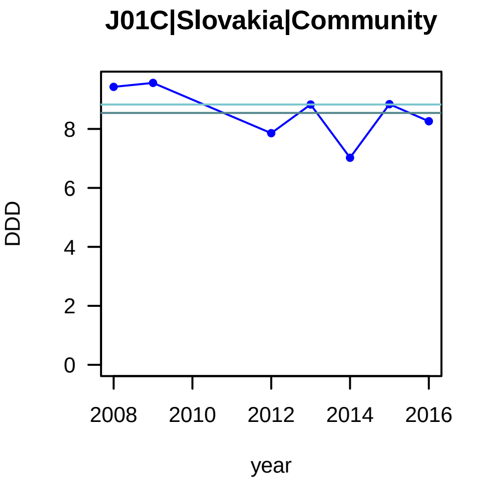
J01C|Slovakia|Community
DDD
0
2
4
6
8
2008
2010
2012
2014
2016
year

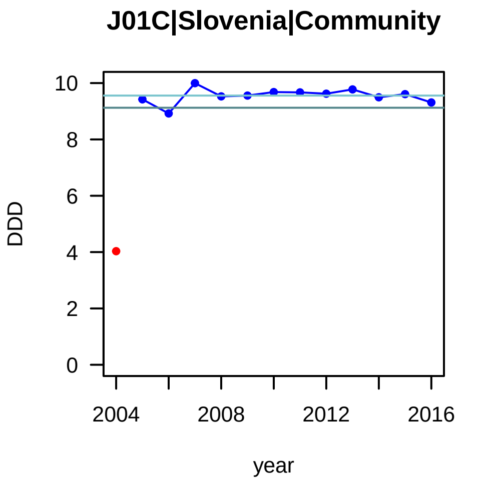
J01C|Slovenia|Community
DDD
0
2
4
6
8
10
2004
2008
2012
2016
year

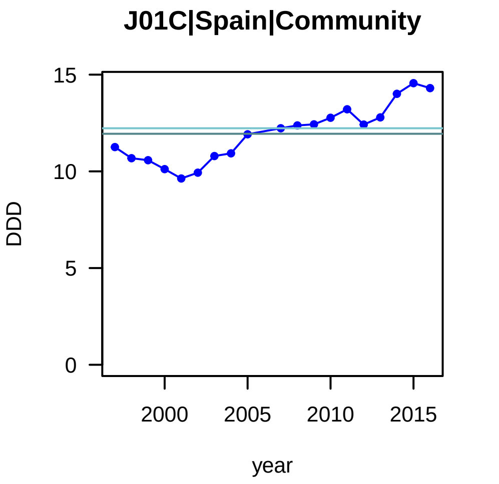
J01C|Spain|Community
DDD
0
5
10
15
2000
2005
2010
2015
year

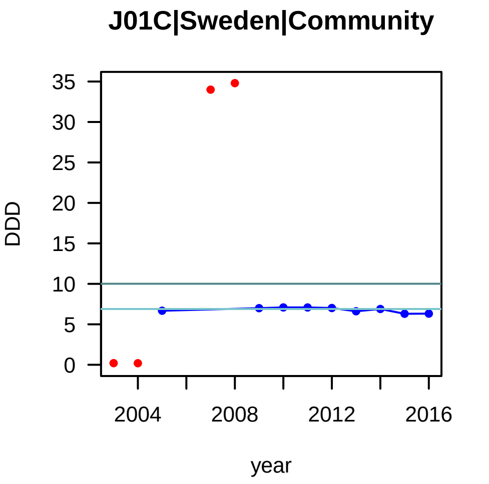
J01C|Sweden|Community
DDD
0
5
10
15
20
25
30
35
2004
2008
2012
2016
year

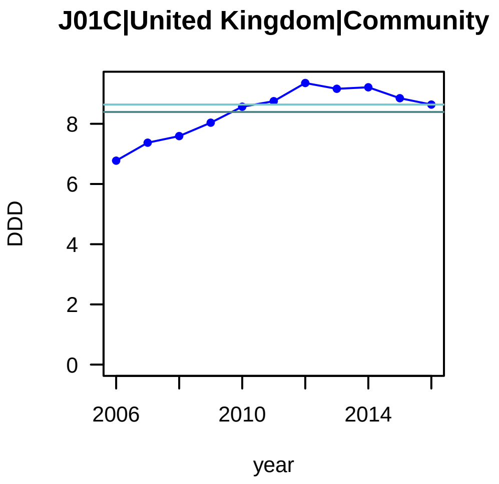
J01C|United Kingdom|Community
DDD
0
2
4
6
8
2006
2010
2014
year

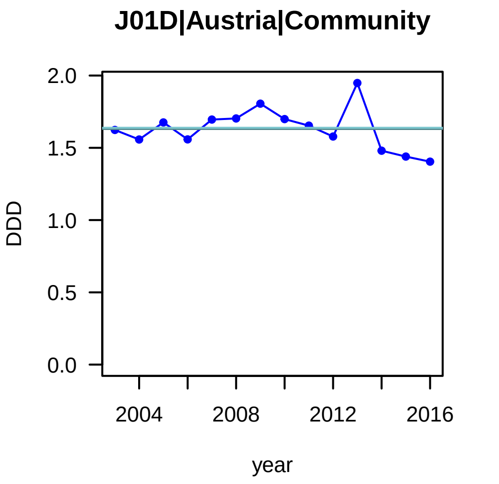
J01D|Austria|Community
DDD
0.0
0.5
1.0
1.5
2.0
2004
2008
2012
2016
year

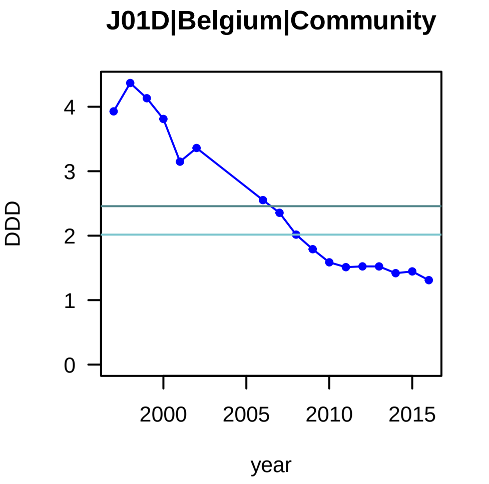
J01D|Belgium|Community
DDD
0
1
2
3
4
2000
2005
2010
2015
year

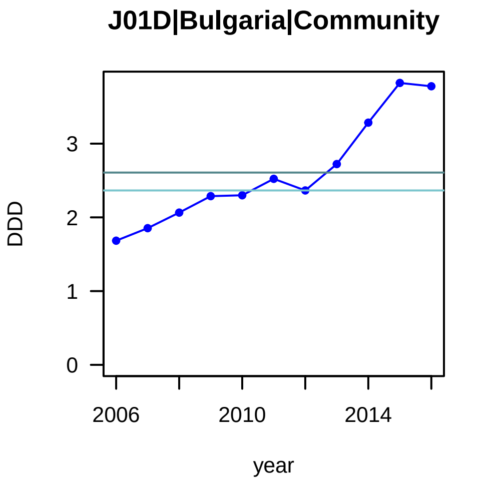
J01D|Bulgaria|Community
DDD
0
1
2
3
2006
2010
2014
year

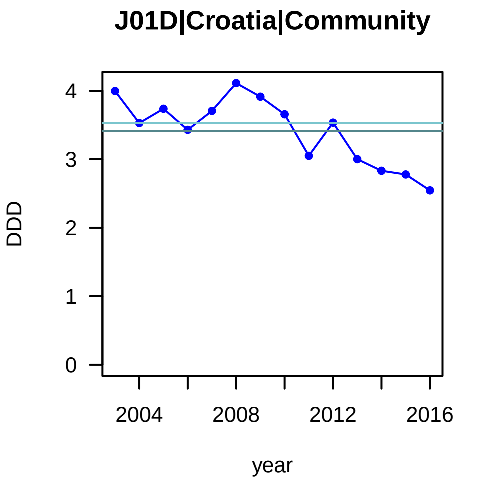
J01D|Croatia|Community
DDD
0
1
2
3
4
2004
2008
2012
2016
year

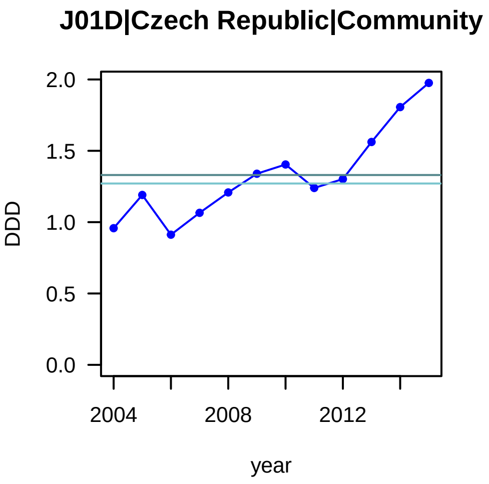
J01D|Czech Republic|Community
DDD
0.0
0.5
1.0
1.5
2.0
2004
2008
2012
year

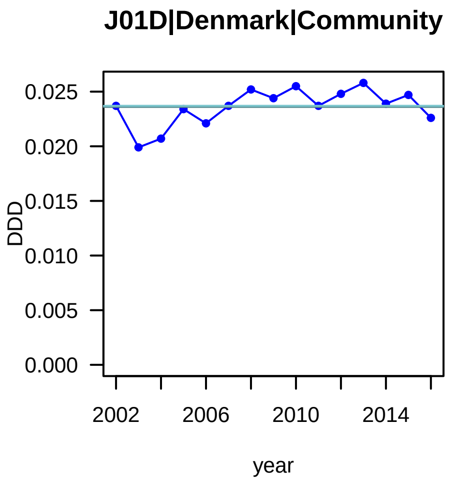
J01D|Denmark|Community
DDD
0.000
0.005
0.010
0.015
0.020
0.025
2002
2006
2010
2014
year

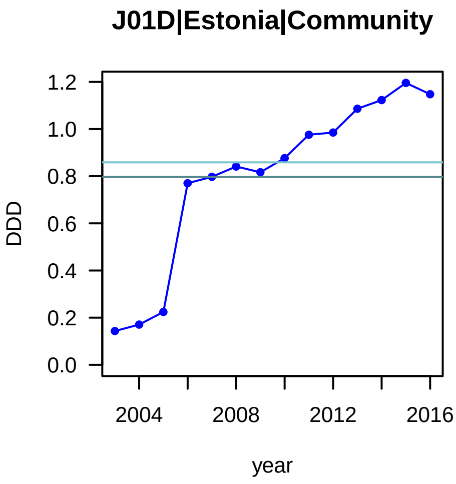
J01D|Estonia|Community
DDD
0.0
0.2
0.4
0.6
0.8
1.0
1.2
2004
2008
2012
2016
year

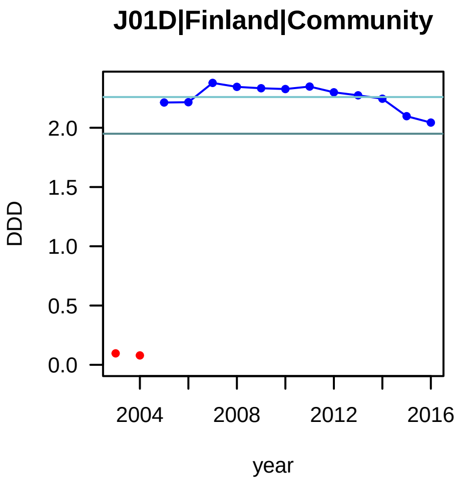
J01D|Finland|Community
DDD
0.0
0.5
1.0
1.5
2.0
2004
2008
2012
2016
year

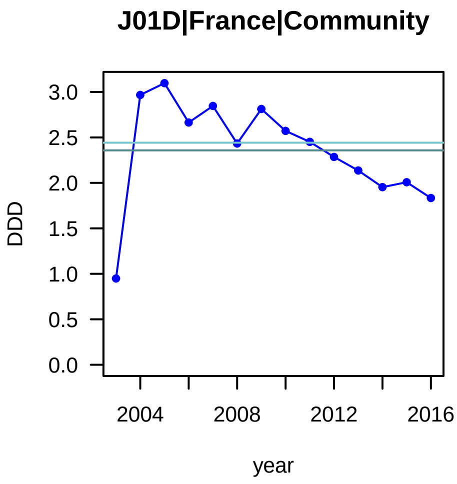
J01D|France|Community
DDD
0.0
0.5
1.0
1.5
2.0
2.5
3.0
2004
2008
2012
2016
year

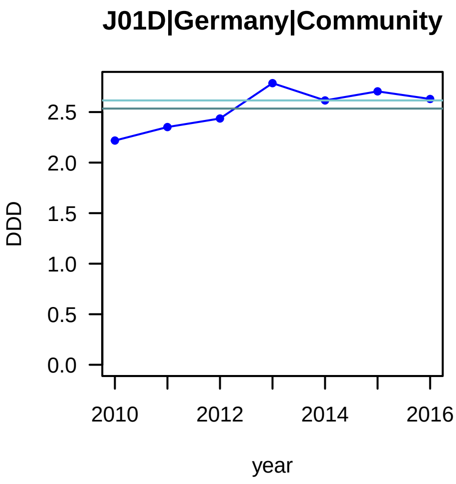
J01D|Germany|Community
DDD
0.0
0.5
1.0
1.5
2.0
2.5
2010
2012
2014
2016
year

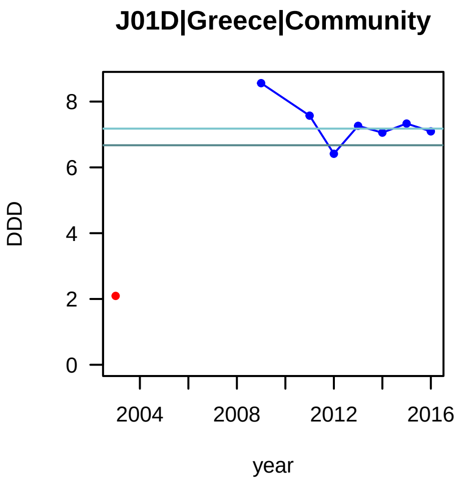
J01D|Greece|Community
DDD
0
2
4
6
8
2004
2008
2012
2016
year

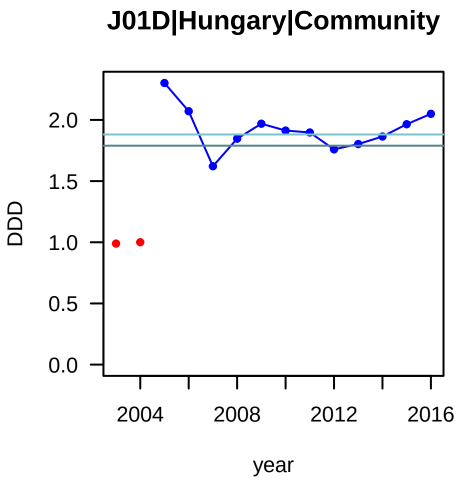
J01D|Hungary|Community
DDD
0.0
0.5
1.0
1.5
2.0
2004
2008
2012
2016
year

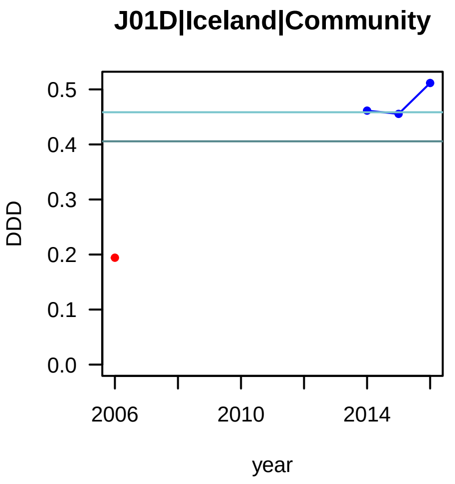
J01D|Iceland|Community
DDD
0.0
0.1
0.2
0.3
0.4
0.5
2006
2010
2014
year

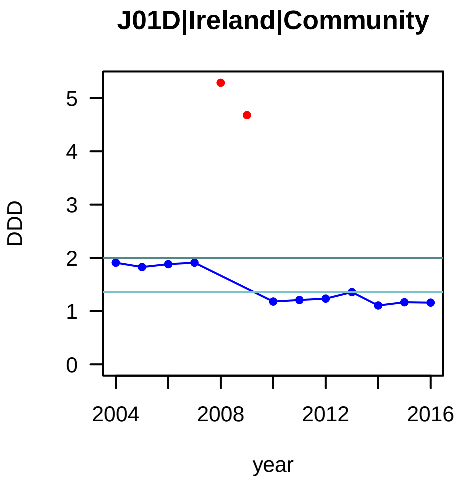
J01D|Ireland|Community
DDD
0
1
2
3
4
5
2004
2008
2012
2016
year

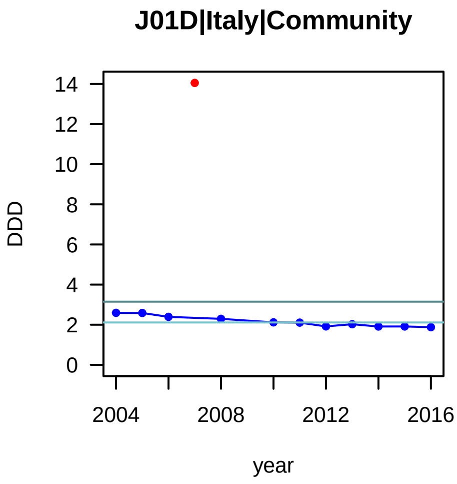
J01D|Italy|Community
DDD
0
2
4
6
8
10
12
14
2004
2008
2012
2016
year

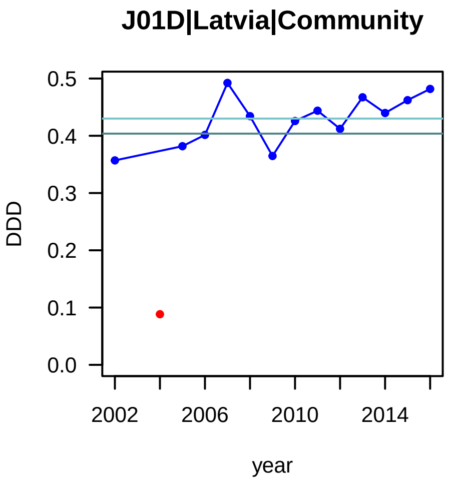
J01D|Latvia|Community
DDD
0.0
0.1
0.2
0.3
0.4
0.5
2002
2006
2010
2014
year

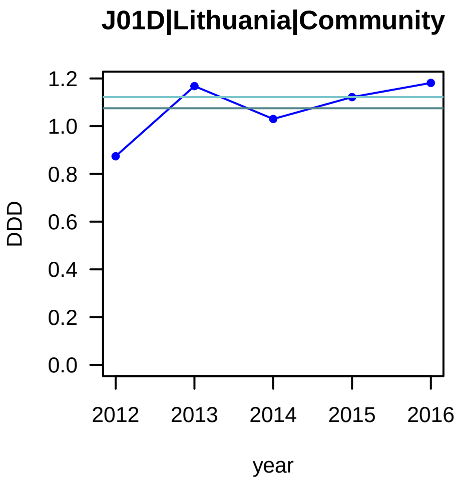
J01D|Lithuania|Community
DDD
0.0
0.2
0.4
0.6
0.8
1.0
1.2
2012
2013
2014
2015
2016
year

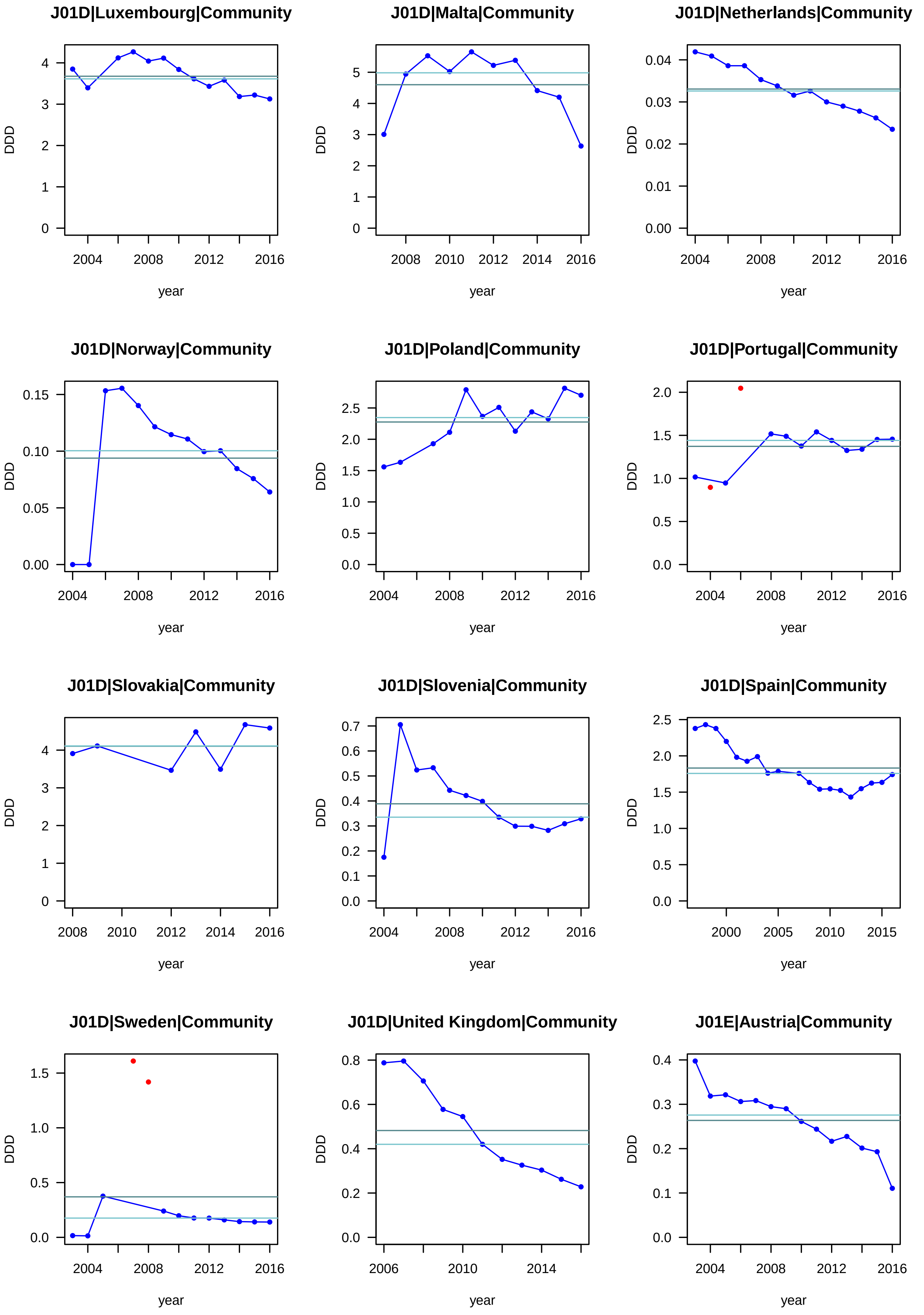
J01D|Luxembourg|Community
J01D|Malta|Community
J01D|Netherlands|Community
J01D|Norway|Community
J01D|Poland|Community
J01D|Portugal|Community
J01D|Slovakia|Community
J01D|Slovenia|Community
J01D|Spain|Community
J01D|Sweden|Community
J01D|United Kingdom|Community
J01E|Austria|Community
DDD
year

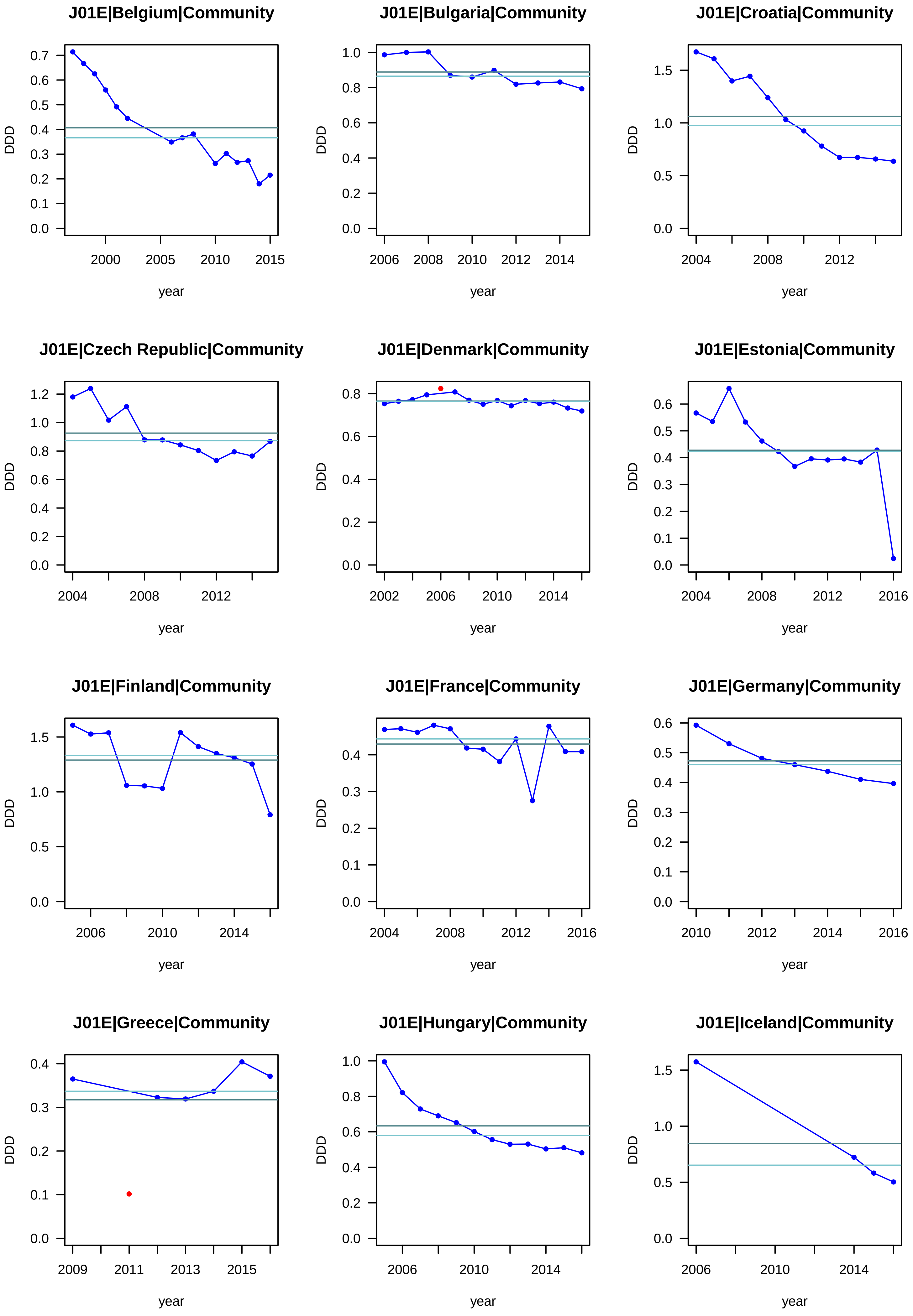

J01E|Belgium|Community
DDD
0.0
0.1
0.2
0.3
0.4
0.5
0.6
0.7
2000
2005
2010
2015
year
J01E|Bulgaria|Community
DDD
0.0
0.2
0.4
0.6
0.8
1.0
2006
2008
2010
2012
2014
year
J01E|Croatia|Community
DDD
0.0
0.5
1.0
1.5
2004
2008
2012
year
J01E|Czech Republic|Community
DDD
0.0
0.2
0.4
0.6
0.8
1.0
1.2
2004
2008
2012
year
J01E|Denmark|Community
DDD
0.0
0.2
0.4
0.6
0.8
2002
2006
2010
2014
year
J01E|Estonia|Community
DDD
0.0
0.1
0.2
0.3
0.4
0.5
0.6
2004
2008
2012
2016
year
J01E|Finland|Community
DDD
0.0
0.5
1.0
1.5
2006
2010
2014
year
J01E|France|Community
DDD
0.0
0.1
0.2
0.3
0.4
2004
2008
2012
2016
year
J01E|Germany|Community
DDD
0.0
0.1
0.2
0.3
0.4
0.5
0.6
2010
2012
2014
2016
year
J01E|Greece|Community
DDD
0.0
0.1
0.2
0.3
0.4
2009
2011
2013
2015
year
J01E|Hungary|Community
DDD
0.0
0.2
0.4
0.6
0.8
1.0
2006
2010
2014
year
J01E|Iceland|Community
DDD
0.0
0.5
1.0
1.5
2006
2010
2014
year

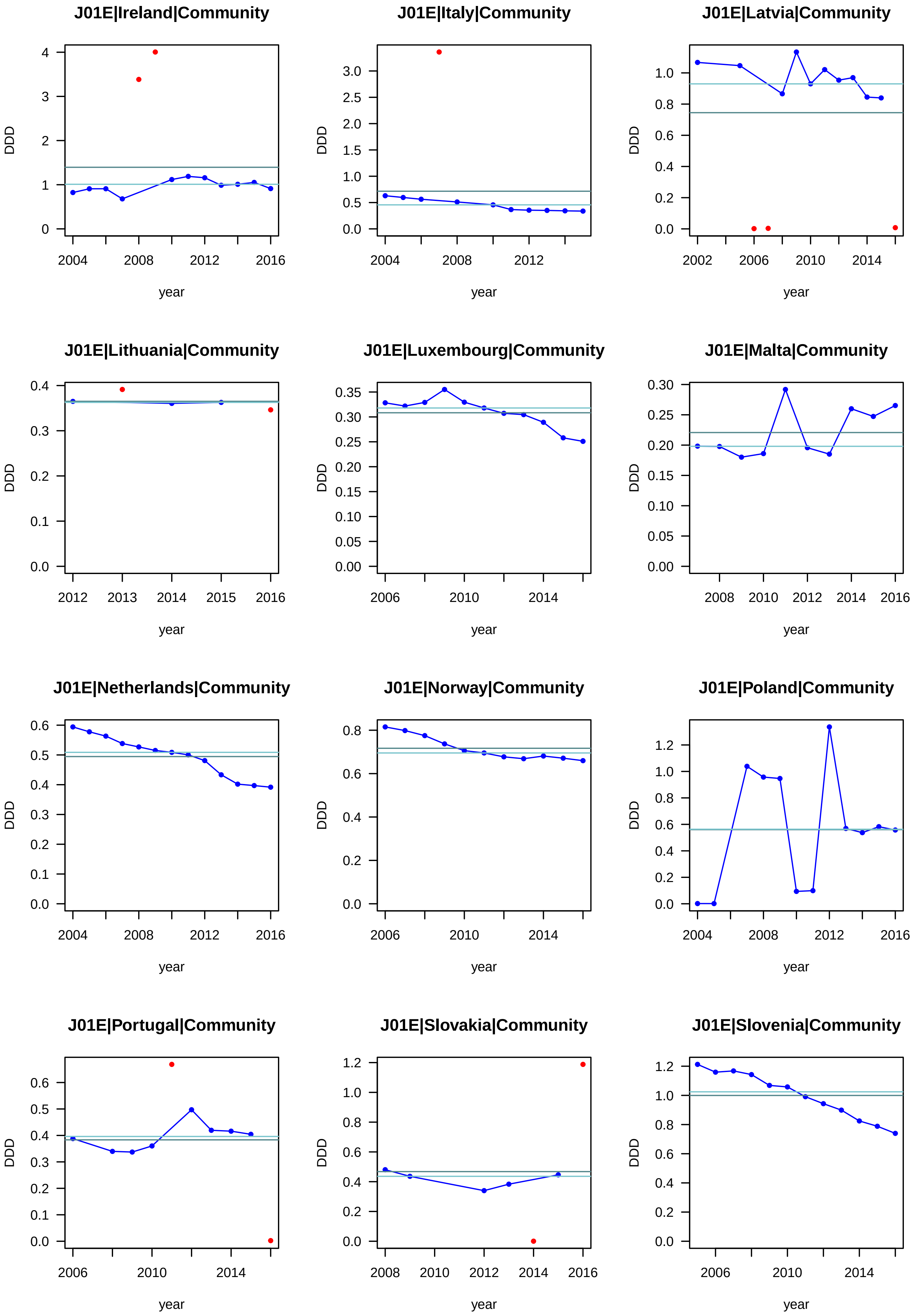

J01E|Ireland|Community
J01E|Italy|Community
J01E|Latvia|Community
J01E|Lithuania|Community
J01E|Luxembourg|Community
J01E|Malta|Community
J01E|Netherlands|Community
J01E|Norway|Community
J01E|Poland|Community
J01E|Portugal|Community
J01E|Slovakia|Community
J01E|Slovenia|Community
DDD
year

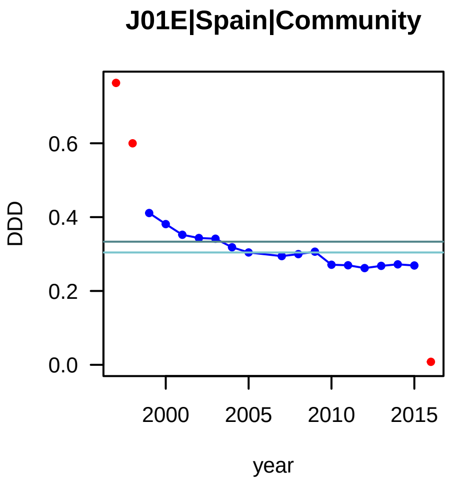
J01E|Spain|Community
DDD
0.0
0.2
0.4
0.6
2000
2005
2010
2015
year

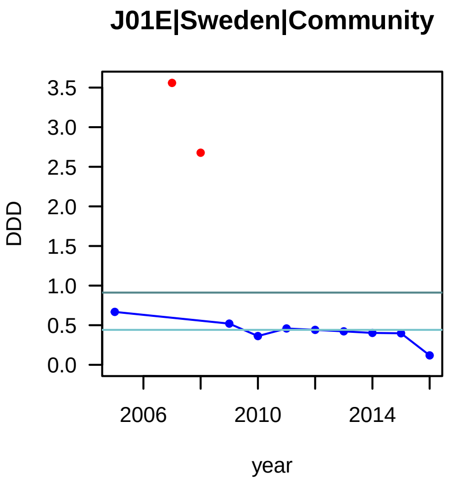
J01E|Sweden|Community
DDD
0.0
0.5
1.0
1.5
2.0
2.5
3.0
3.5
2006
2010
2014
year

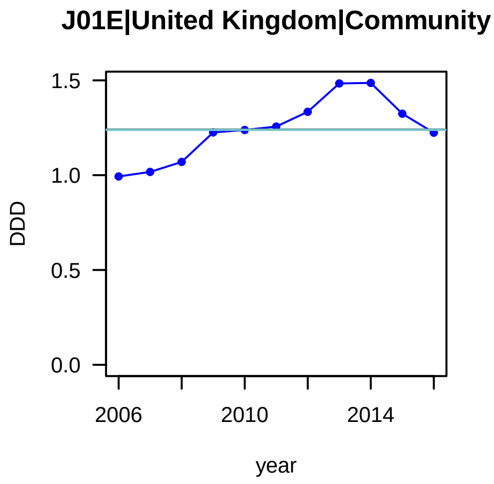
J01E|United Kingdom|Community
DDD
0.0
0.5
1.0
1.5
2006
2010
2014
year

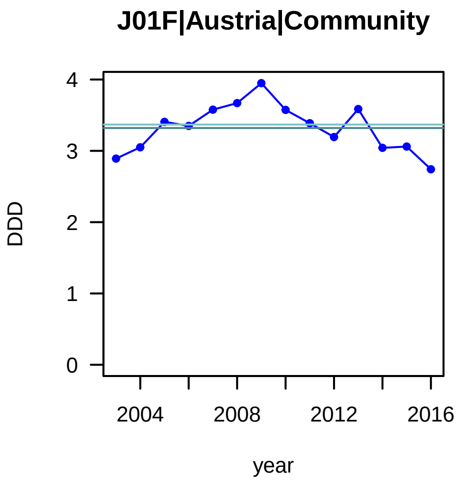
J01F|Austria|Community
DDD
0
1
2
3
4
2004
2008
2012
2016
year

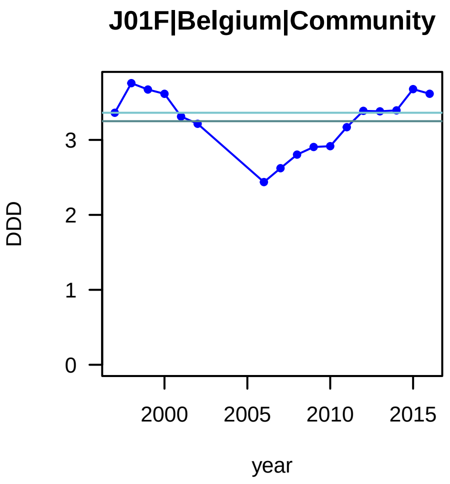
J01F|Belgium|Community
DDD
0
1
2
3
2000
2005
2010
2015
year

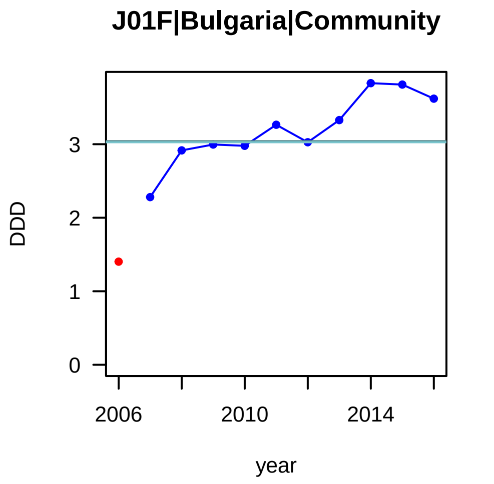
J01F|Bulgaria|Community
DDD
0
1
2
3
2006
2010
2014
year

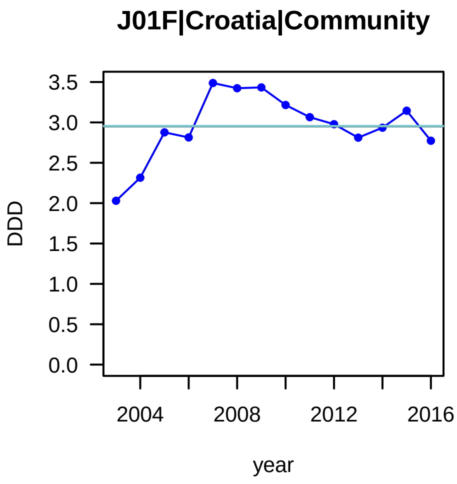
J01F|Croatia|Community
DDD
0.0
0.5
1.0
1.5
2.0
2.5
3.0
3.5
2004
2008
2012
2016
year

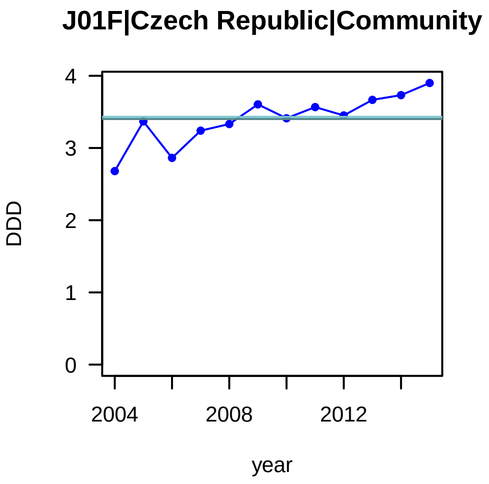
J01F|Czech Republic|Community
DDD
0
1
2
3
4
2004
2008
2012
year

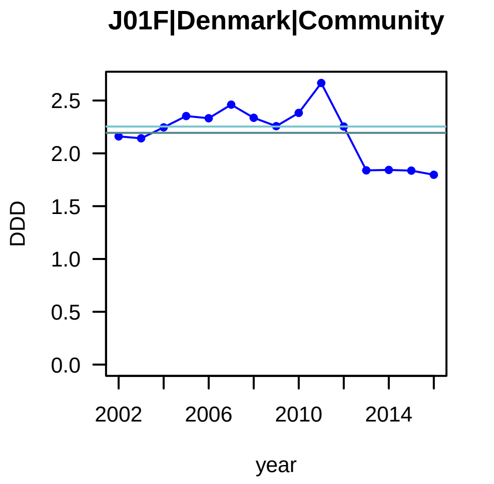
J01F|Denmark|Community
DDD
0.0
0.5
1.0
1.5
2.0
2.5
2002
2006
2010
2014
year

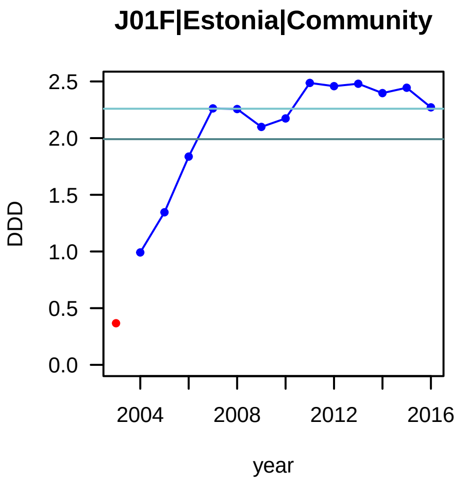
J01F|Estonia|Community
DDD
0.0
0.5
1.0
1.5
2.0
2.5
2004
2008
2012
2016
year

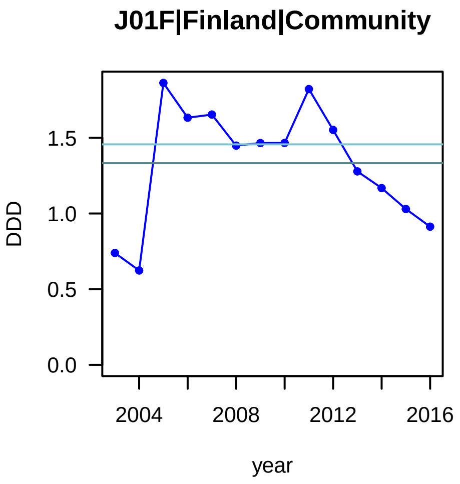
J01F|Finland|Community
DDD
0.0
0.5
1.0
1.5
2004
2008
2012
2016
year

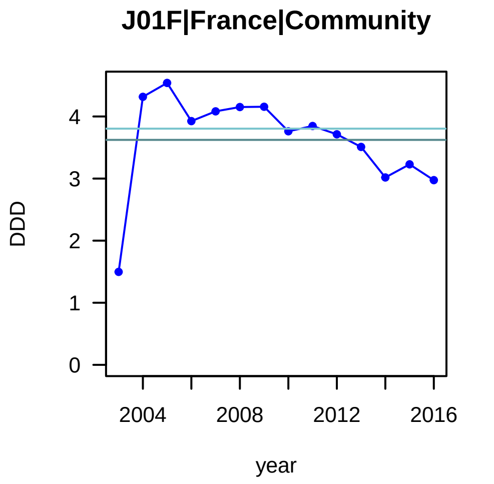
J01F|France|Community
DDD
0
1
2
3
4
2004
2008
2012
2016
year

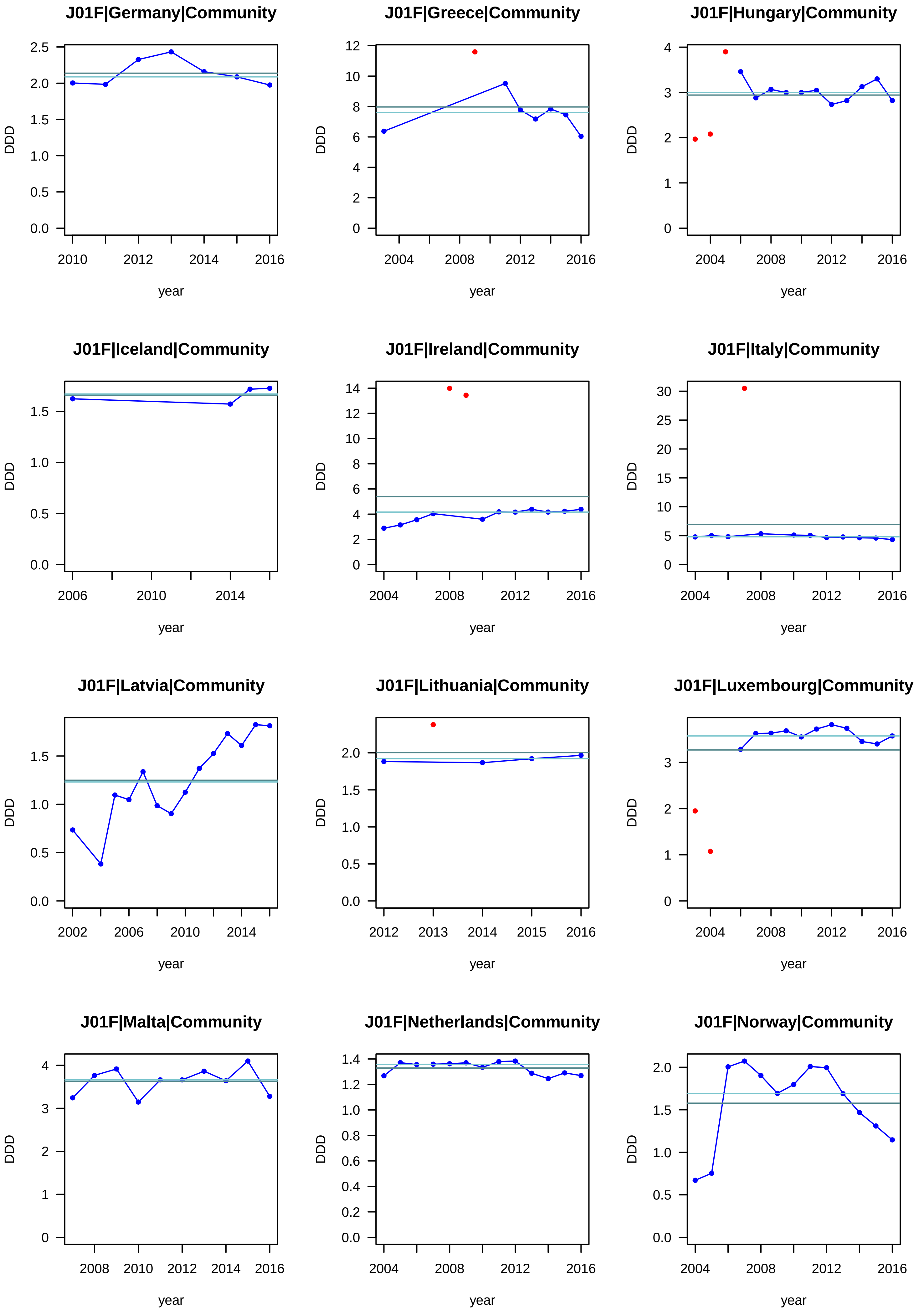
J01F|Germany|Community
J01F|Greece|Community
J01F|Hungary|Community
J01F|Iceland|Community
J01F|Ireland|Community
J01F|Italy|Community
J01F|Latvia|Community
J01F|Lithuania|Community
J01F|Luxembourg|Community
J01F|Malta|Community
J01F|Netherlands|Community
J01F|Norway|Community
DDD
year

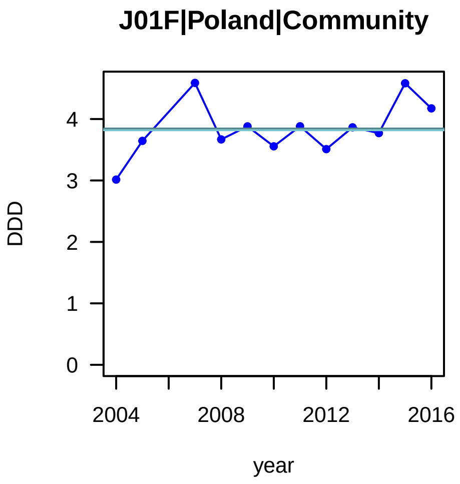
J01F|Poland|Community
DDD
0
1
2
3
4
2004
2008
2012
2016
year

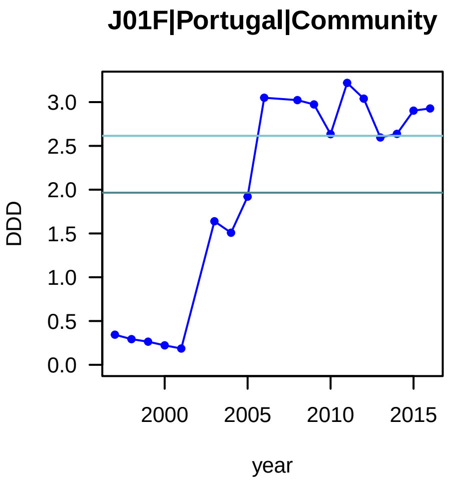
J01F|Portugal|Community
DDD
0.0
0.5
1.0
1.5
2.0
2.5
3.0
2000
2005
2010
2015
year

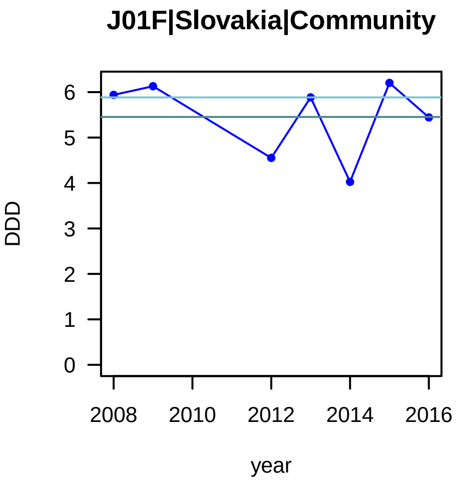
J01F|Slovakia|Community
DDD
0
1
2
3
4
5
6
2008
2010
2012
2014
2016
year

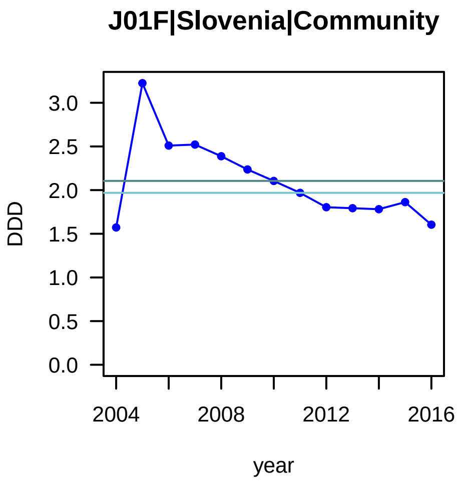
J01F|Slovenia|Community
DDD
0.0
0.5
1.0
1.5
2.0
2.5
3.0
2004
2008
2012
2016
year

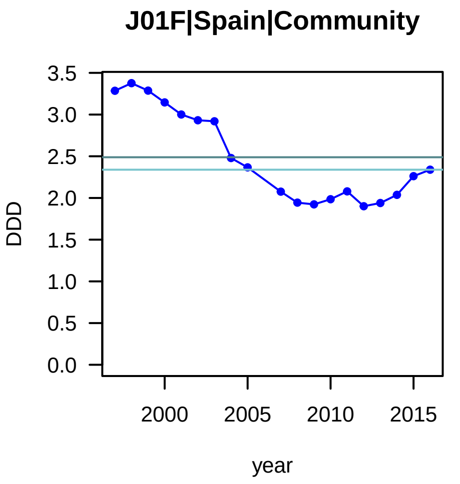
J01F|Spain|Community
DDD
0.0
0.5
1.0
1.5
2.0
2.5
3.0
3.5
2000
2005
2010
2015
year

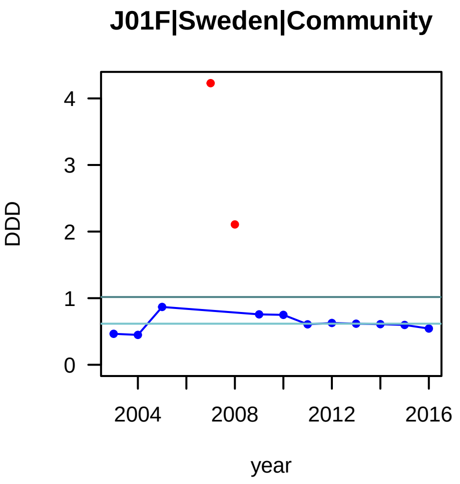
J01F|Sweden|Community
DDD
0
1
2
3
4
2004
2008
2012
2016
year

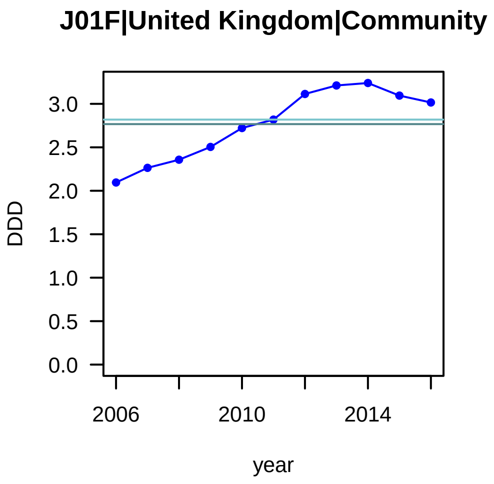
J01F|United Kingdom|Community
DDD
0.0
0.5
1.0
1.5
2.0
2.5
3.0
2006
2010
2014
year

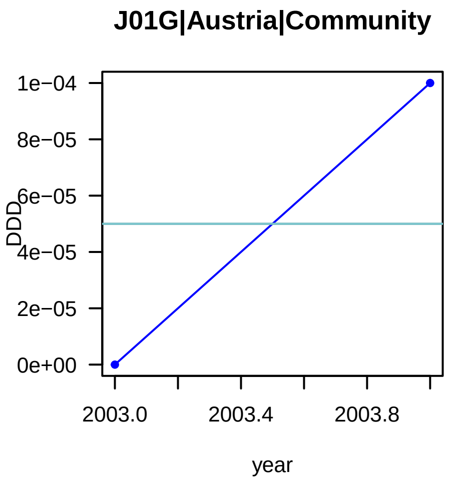
J01G|Austria|Community
DDD
0e+00
2e−05
4e−05
6e−05
8e−05
1e−04
2003.0
2003.4
2003.8
year

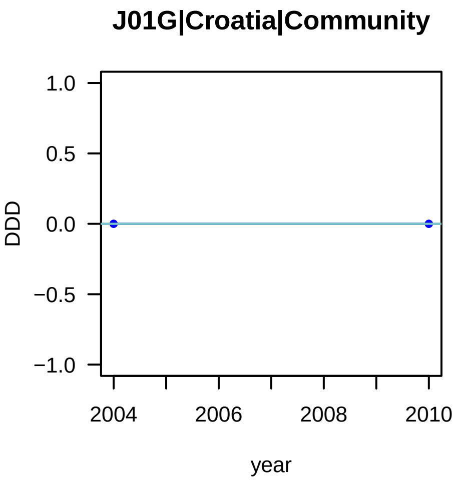
J01G|Croatia|Community
DDD
−1.0
−0.5
0.0
0.5
1.0
2004
2006
2008
2010
year

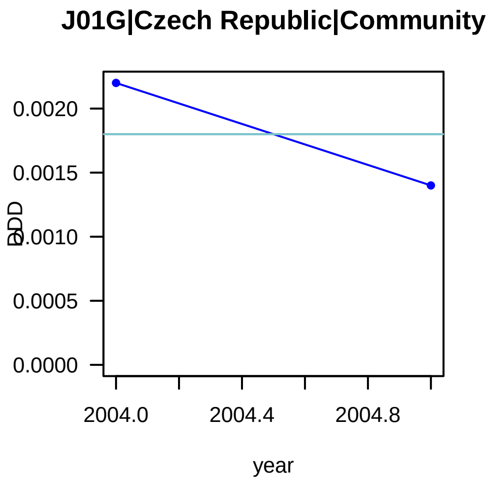
J01G|Czech Republic|Community
DDD
0.0000
0.0005
0.0010
0.0015
0.0020
2004.0
2004.4
2004.8
year

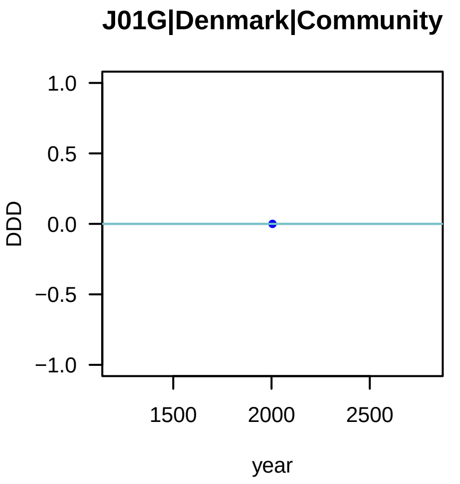
J01G|Denmark|Community
DDD
−1.0
−0.5
0.0
0.5
1.0
1500
2000
2500
year

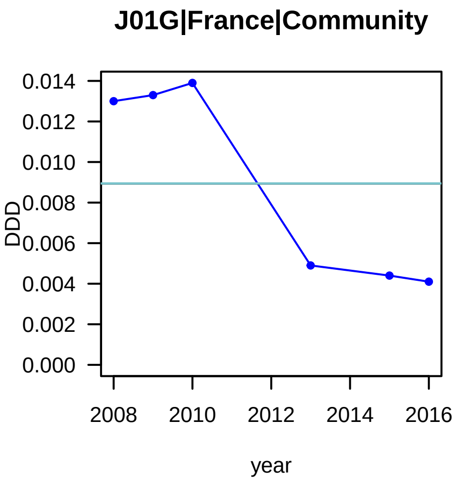
J01G|France|Community
DDD
0.000
0.002
0.004
0.006
0.008
0.010
0.012
0.014
2008
2010
2012
2014
2016
year

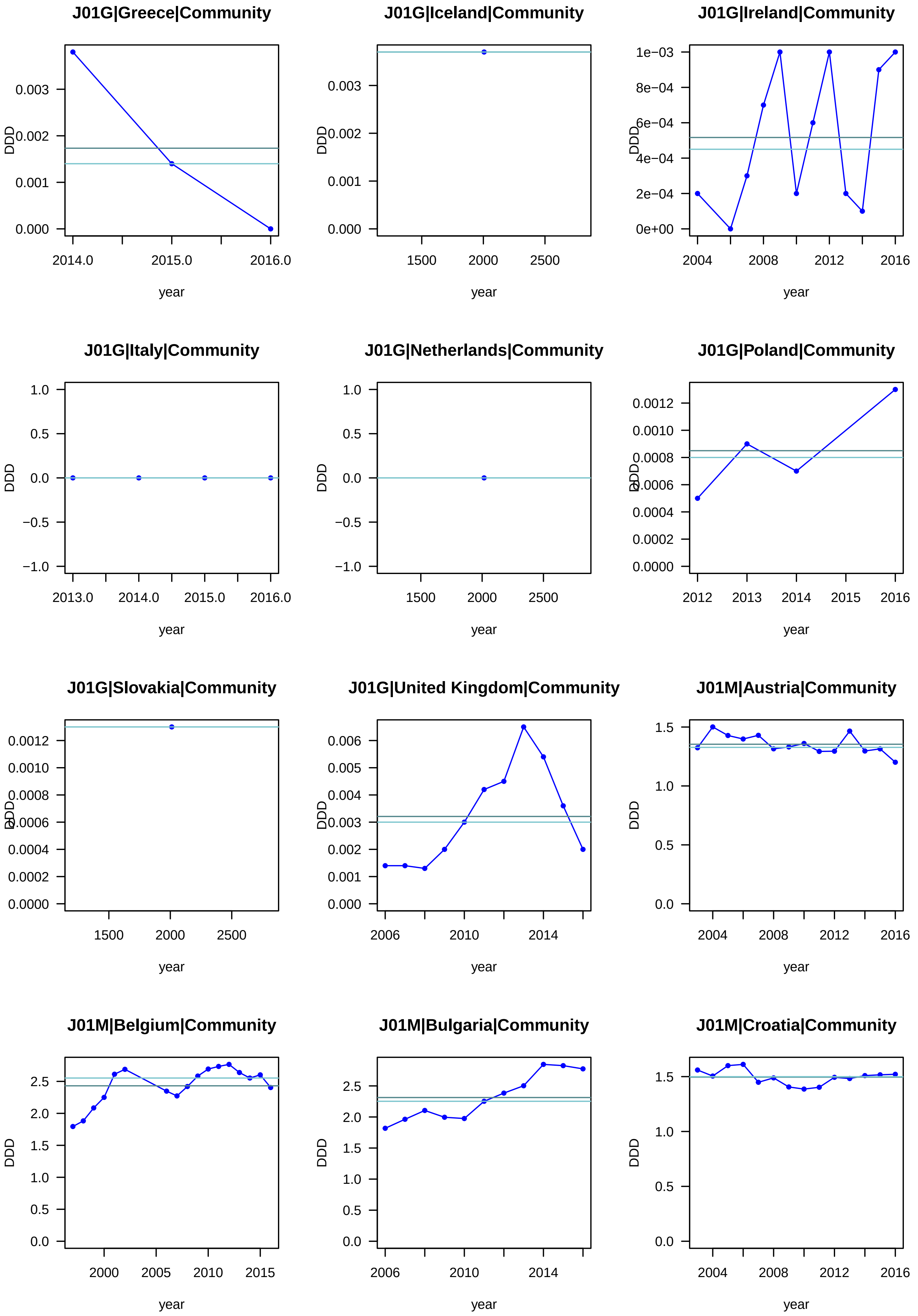
J01G|Greece|Community
DDD
0.000
0.001
0.002
0.003
2014.0
2015.0
2016.0
year
J01G|Iceland|Community
DDD
0.000
0.001
0.002
0.003
1500
2000
2500
year
J01G|Ireland|Community
DDD
0e+00
2e−04
4e−04
6e−04
8e−04
1e−03
2004
2008
2012
2016
year
J01G|Italy|Community
DDD
−1.0
−0.5
0.0
0.5
1.0
2013.0
2014.0
2015.0
2016.0
year
J01G|Netherlands|Community
DDD
−1.0
−0.5
0.0
0.5
1.0
1500
2000
2500
year
J01G|Poland|Community
DDD
0.0000
0.0002
0.0004
0.0006
0.0008
0.0010
0.0012
2012
2013
2014
2015
2016
year
J01G|Slovakia|Community
DDD
0.0000
0.0002
0.0004
0.0006
0.0008
0.0010
0.0012
1500
2000
2500
year
J01G|United Kingdom|Community
DDD
0.000
0.001
0.002
0.003
0.004
0.005
0.006
2006
2010
2014
year
J01M|Austria|Community
DDD
0.0
0.5
1.0
1.5
2004
2008
2012
2016
year
J01M|Belgium|Community
DDD
0.0
0.5
1.0
1.5
2.0
2.5
2000
2005
2010
2015
year
J01M|Bulgaria|Community
DDD
0.0
0.5
1.0
1.5
2.0
2.5
2006
2010
2014
year
J01M|Croatia|Community
DDD
0.0
0.5
1.0
1.5
2004
2008
2012
2016
year

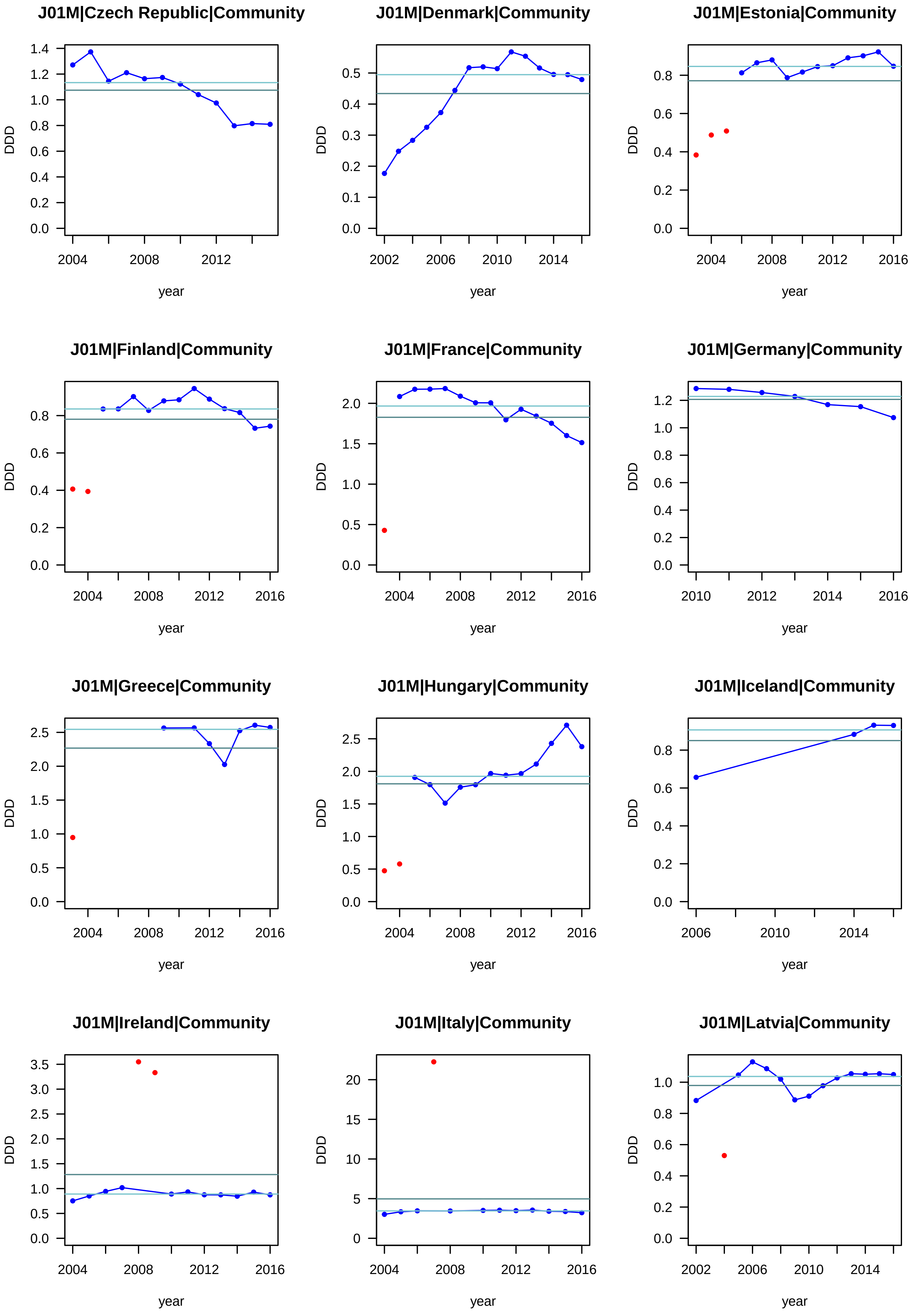
J01M|Czech Republic|Community
DDD
1.4
1.2
1.0
0.8
0.6
0.4
0.2
0.0
2004
2008
2012
year
J01M|Denmark|Community
DDD
0.5
0.4
0.3
0.2
0.1
0.0
2002
2006
2010
2014
year
J01M|Estonia|Community
DDD
0.8
0.6
0.4
0.2
0.0
2004
2008
2012
2016
year
J01M|Finland|Community
DDD
0.8
0.6
0.4
0.2
0.0
2004
2008
2012
2016
year
J01M|France|Community
DDD
2.0
1.5
1.0
0.5
0.0
2004
2008
2012
2016
year
J01M|Germany|Community
DDD
1.2
1.0
0.8
0.6
0.4
0.2
0.0
2010
2012
2014
2016
year
J01M|Greece|Community
DDD
2.5
2.0
1.5
1.0
0.5
0.0
2004
2008
2012
2016
year
J01M|Hungary|Community
DDD
2.5
2.0
1.5
1.0
0.5
0.0
2004
2008
2012
2016
year
J01M|Iceland|Community
DDD
0.8
0.6
0.4
0.2
0.0
2006
2010
2014
year
J01M|Ireland|Community
DDD
3.5
3.0
2.5
2.0
1.5
1.0
0.5
0.0
2004
2008
2012
2016
year
J01M|Italy|Community
DDD
20
15
10
5
0
2004
2008
2012
2016
year
J01M|Latvia|Community
DDD
1.0
0.8
0.6
0.4
0.2
0.0
2002
2006
2010
2014
year

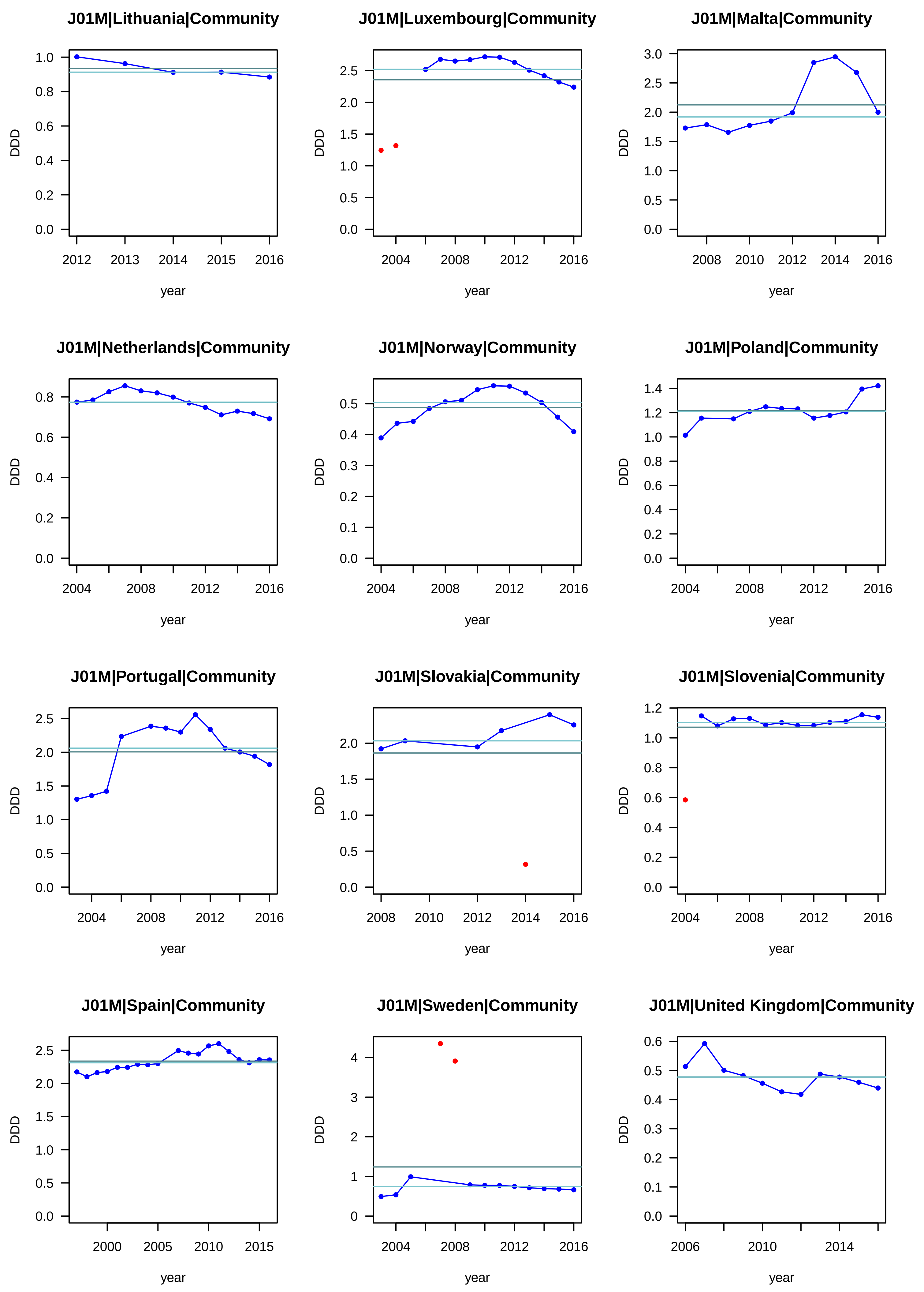
J01M|Lithuania|Community
J01M|Luxembourg|Community
J01M|Malta|Community
J01M|Netherlands|Community
J01M|Norway|Community
J01M|Poland|Community
J01M|Portugal|Community
J01M|Slovakia|Community
J01M|Slovenia|Community
J01M|Spain|Community
J01M|Sweden|Community
J01M|United Kingdom|Community
DDD
year

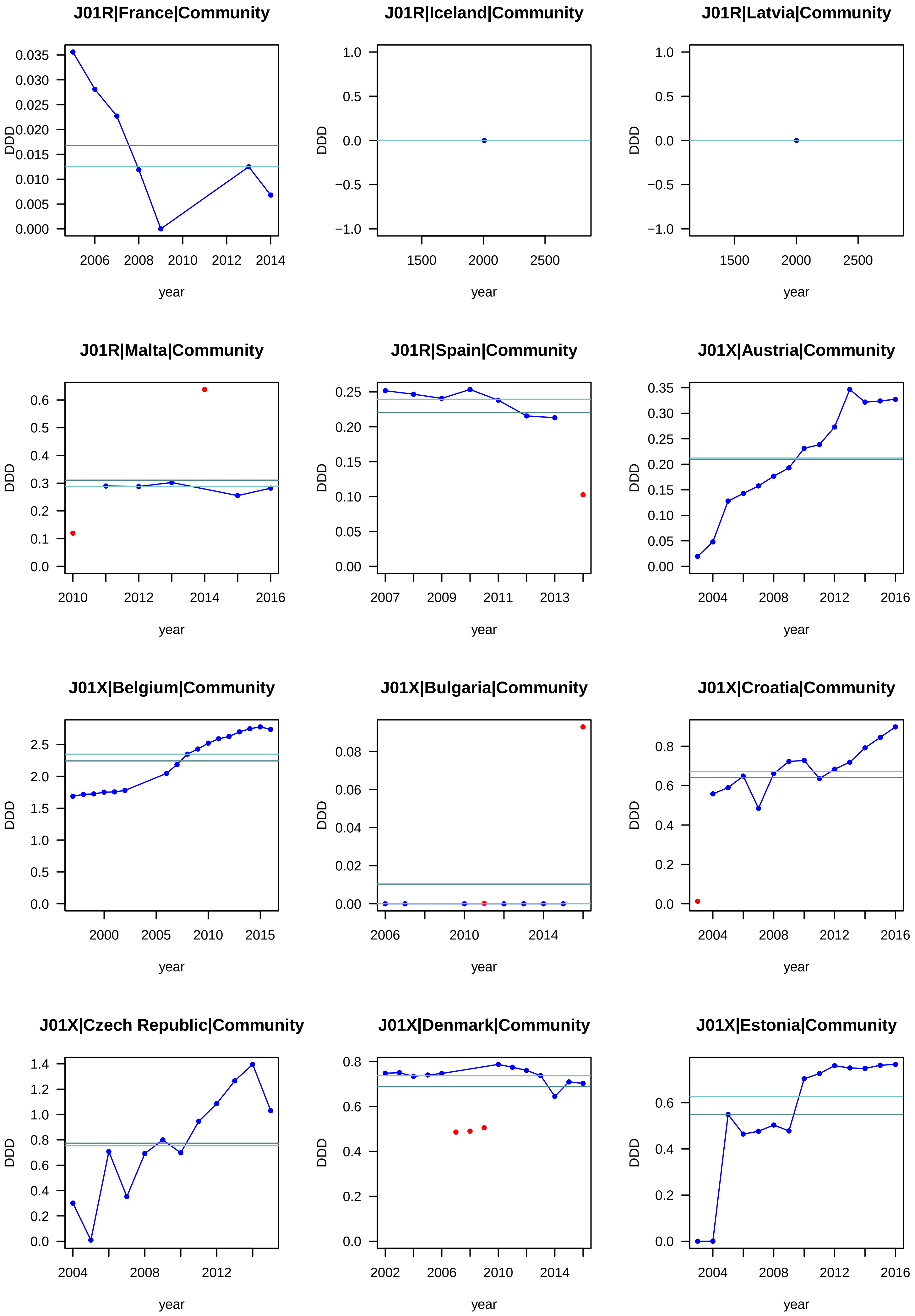
J01R|France|Community
DDD
0.035 0.030 0.025 0.020 0.015 0.010 0.005 0.000
2006 2008 2010 2012 2014
year
J01R|Iceland|Community
DDD
1.0 0.5 0.0 −0.5 −1.0
1500 2000 2500
year
J01R|Latvia|Community
DDD
1.0 0.5 0.0 −0.5 −1.0
1500 2000 2500
year
J01R|Malta|Community
DDD
0.6 0.5 0.4 0.3 0.2 0.1 0.0
2010 2012 2014 2016
year
J01R|Spain|Community
DDD
0.25 0.20 0.15 0.10 0.05 0.00
2007 2009 2011 2013
year
J01X|Austria|Community
DDD
0.35 0.30 0.25 0.20 0.15 0.10 0.05 0.00
2004 2008 2012 2016
year
J01X|Belgium|Community
DDD
2.5 2.0 1.5 1.0 0.5 0.0
2000 2005 2010 2015
year
J01X|Bulgaria|Community
DDD
0.08 0.06 0.04 0.02 0.00
2006 2010 2014
year
J01X|Croatia|Community
DDD
0.8 0.6 0.4 0.2 0.0
2004 2008 2012 2016
year
J01X|Czech Republic|Community
DDD
1.4 1.2 1.0 0.8 0.6 0.4 0.2 0.0
2004 2008 2012
year
J01X|Denmark|Community
DDD
0.8 0.6 0.4 0.2 0.0
2002 2006 2010 2014
year
J01X|Estonia|Community
DDD
0.6 0.4 0.2 0.0
2004 2008 2012 2016
year

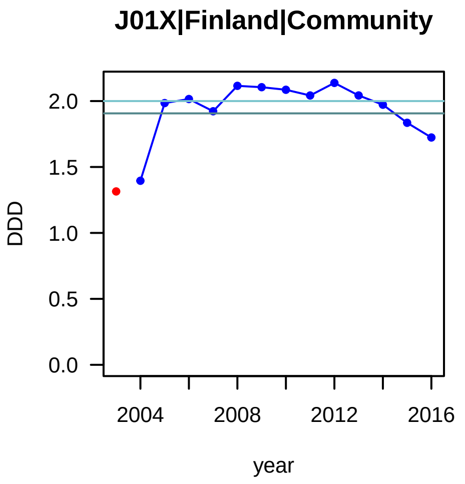
J01X|Finland|Community
DDD
2.0
1.5
1.0
0.5
0.0
2004
2008
2012
2016
year

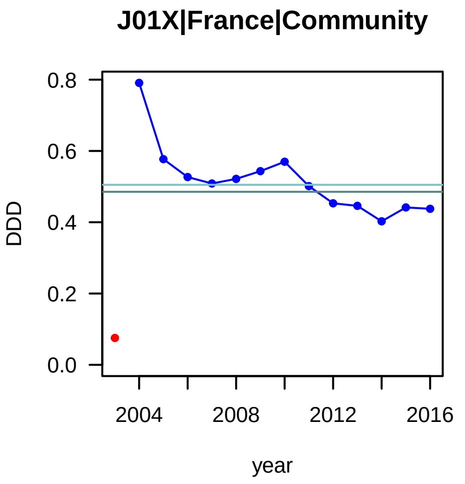
J01X|France|Community
DDD
0.8
0.6
0.4
0.2
0.0
2004
2008
2012
2016
year

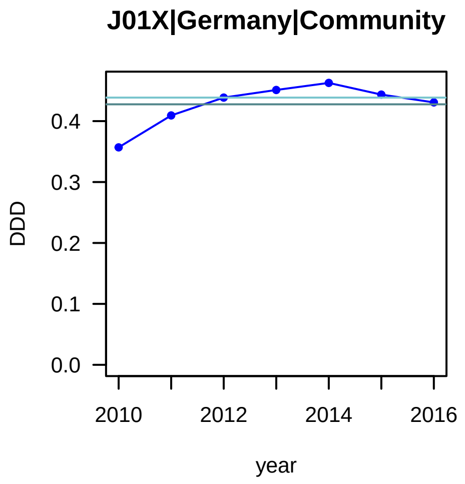
J01X|Germany|Community
DDD
0.4
0.3
0.2
0.1
0.0
2010
2012
2014
2016
year

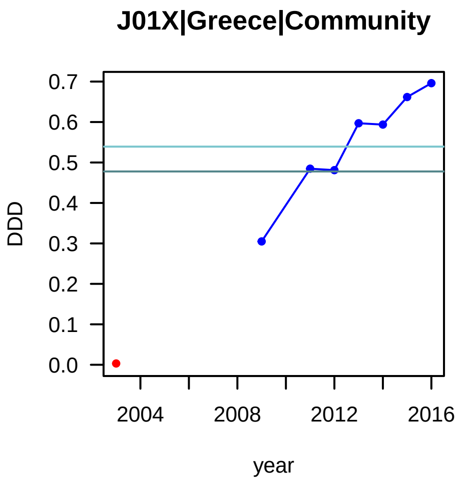
J01X|Greece|Community
DDD
0.7
0.6
0.5
0.4
0.3
0.2
0.1
0.0
2004
2008
2012
2016
year

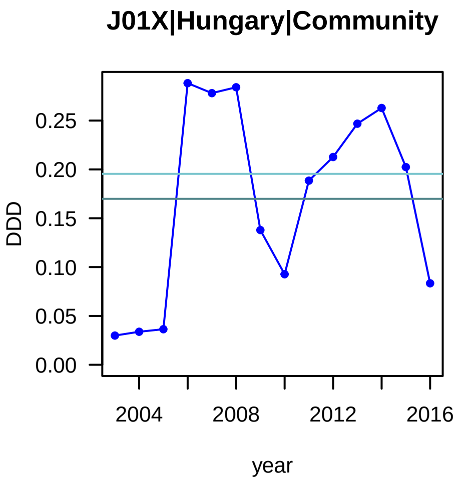
J01X|Hungary|Community
DDD
0.25
0.20
0.15
0.10
0.05
0.00
2004
2008
2012
2016
year

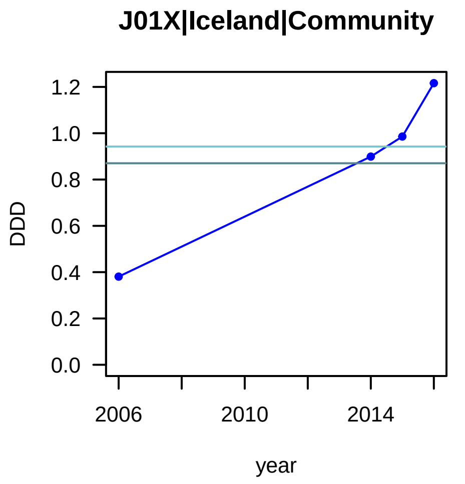
J01X|Iceland|Community
DDD
1.2
1.0
0.8
0.6
0.4
0.2
0.0
2006
2010
2014
year

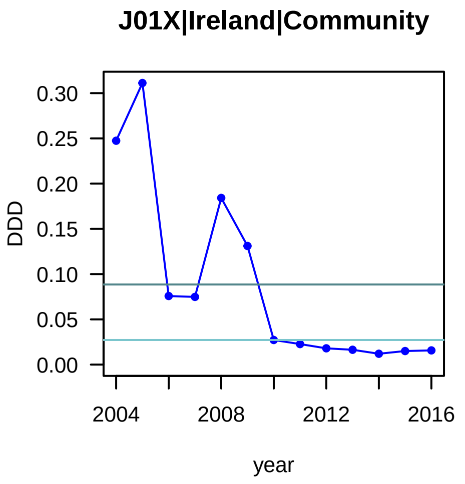
J01X|Ireland|Community
DDD
0.30
0.25
0.20
0.15
0.10
0.05
0.00
2004
2008
2012
2016
year

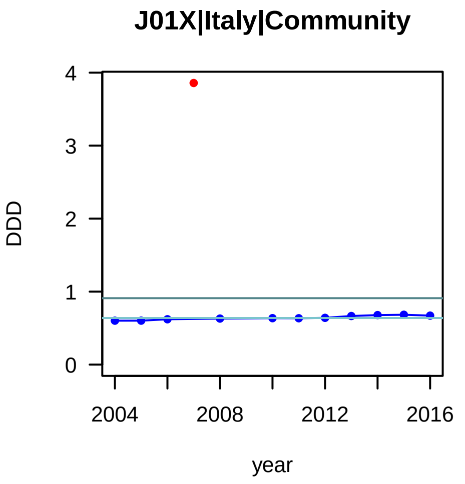
J01X|Italy|Community
DDD
4
3
2
1
0
2004
2008
2012
2016
year

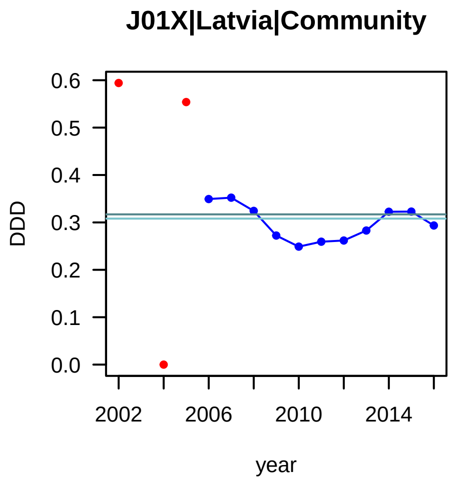
J01X|Latvia|Community
DDD
0.6
0.5
0.4
0.3
0.2
0.1
0.0
2002
2006
2010
2014
year

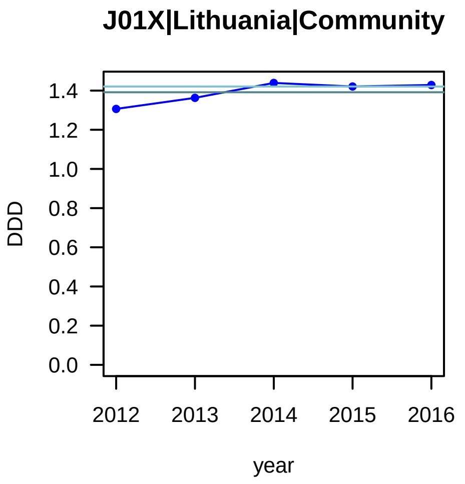
J01X|Lithuania|Community
DDD
1.4
1.2
1.0
0.8
0.6
0.4
0.2
0.0
2012
2013
2014
2015
2016
year

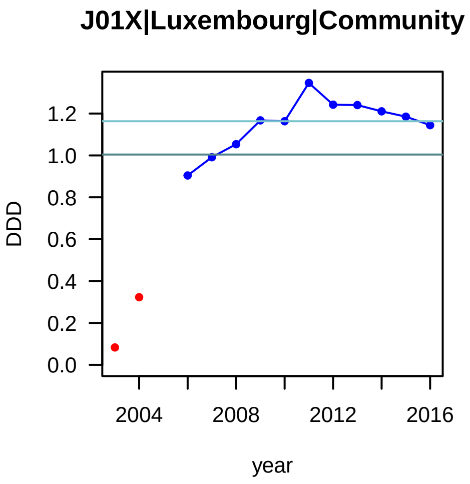
J01X|Luxembourg|Community
DDD
1.2
1.0
0.8
0.6
0.4
0.2
0.0
2004
2008
2012
2016
year

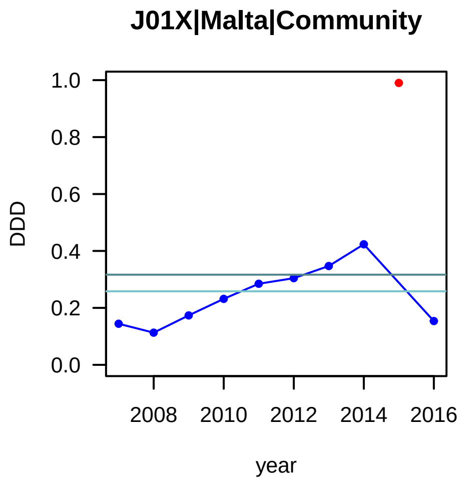
J01X|Malta|Community
DDD
1.0
0.8
0.6
0.4
0.2
0.0
2008
2010
2012
2014
2016
year

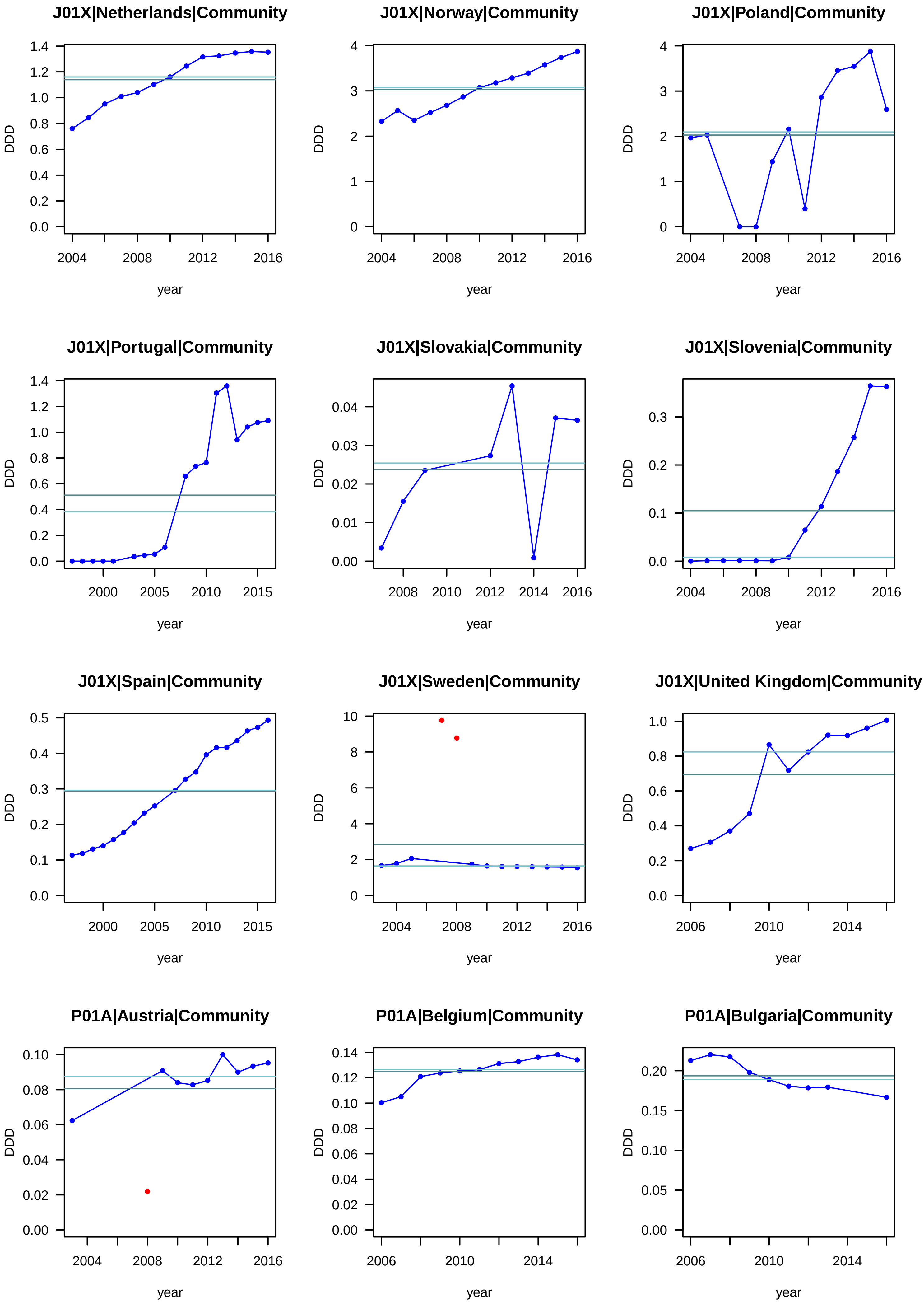

J01X|Netherlands|Community
J01X|Norway|Community
J01X|Poland|Community
J01X|Portugal|Community
J01X|Slovakia|Community
J01X|Slovenia|Community
J01X|Spain|Community
J01X|Sweden|Community
J01X|United Kingdom|Community
P01A|Austria|Community
P01A|Belgium|Community
P01A|Bulgaria|Community
DDD
year

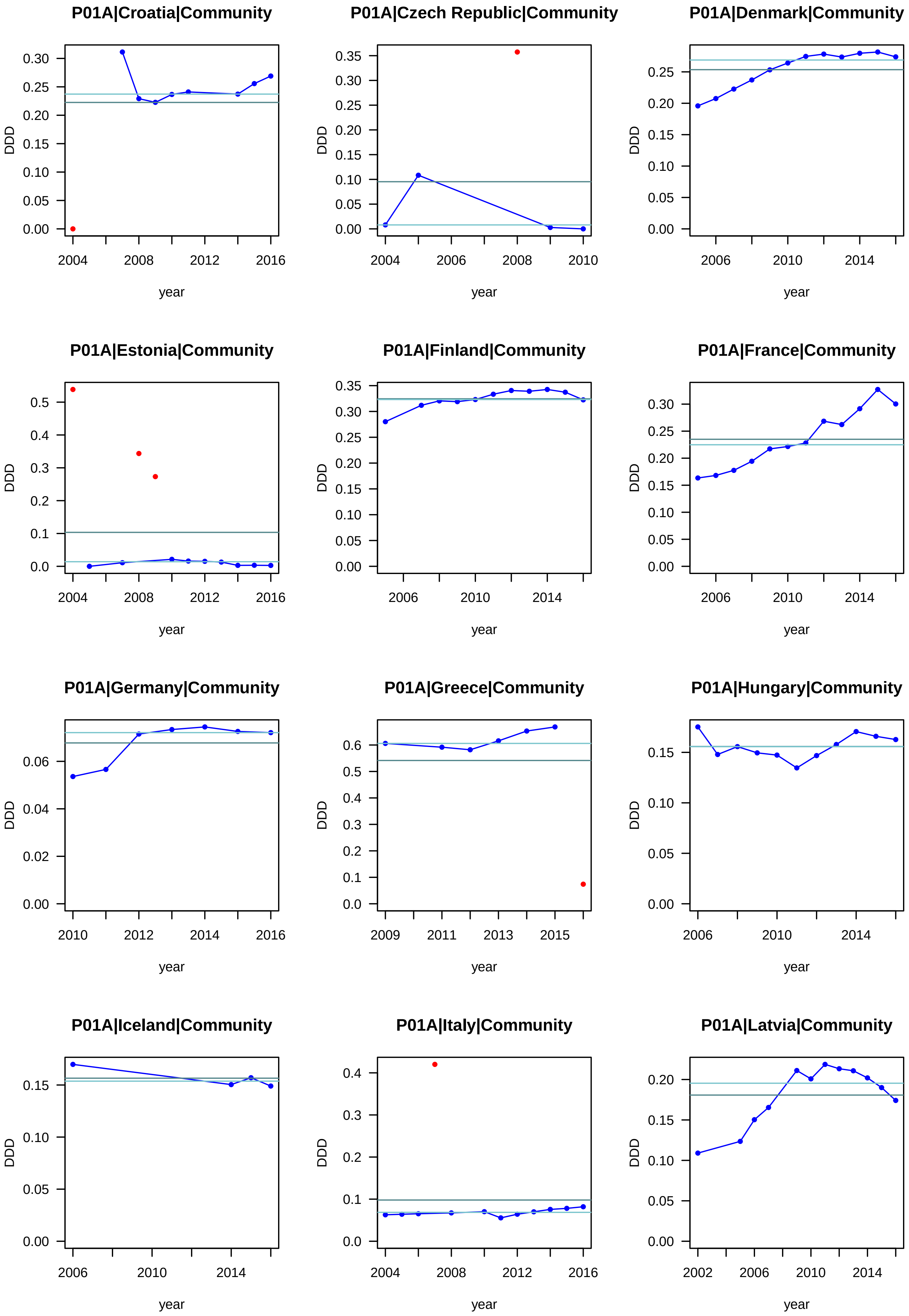
P01A|Croatia|Community
DDD
0.00 0.05 0.10 0.15 0.20 0.25 0.30
2004 2008 2012 2016
year
P01A|Czech Republic|Community
DDD
0.00 0.05 0.10 0.15 0.20 0.25 0.30 0.35
2004 2006 2008 2010
year
P01A|Denmark|Community
DDD
0.00 0.05 0.10 0.15 0.20 0.25
2006 2010 2014
year
P01A|Estonia|Community
DDD
0.0 0.1 0.2 0.3 0.4 0.5
2004 2008 2012 2016
year
P01A|Finland|Community
DDD
0.00 0.05 0.10 0.15 0.20 0.25 0.30 0.35
2006 2010 2014
year
P01A|France|Community
DDD
0.00 0.05 0.10 0.15 0.20 0.25 0.30
2006 2010 2014
year
P01A|Germany|Community
DDD
0.00 0.02 0.04 0.06
2010 2012 2014 2016
year
P01A|Greece|Community
DDD
0.0 0.1 0.2 0.3 0.4 0.5 0.6
2009 2011 2013 2015
year
P01A|Hungary|Community
DDD
0.00 0.05 0.10 0.15
2006 2010 2014
year
P01A|Iceland|Community
DDD
0.00 0.05 0.10 0.15
2006 2010 2014
year
P01A|Italy|Community
DDD
0.0 0.1 0.2 0.3 0.4
2004 2008 2012 2016
year
P01A|Latvia|Community
DDD
0.00 0.05 0.10 0.15 0.20
2002 2006 2010 2014
year

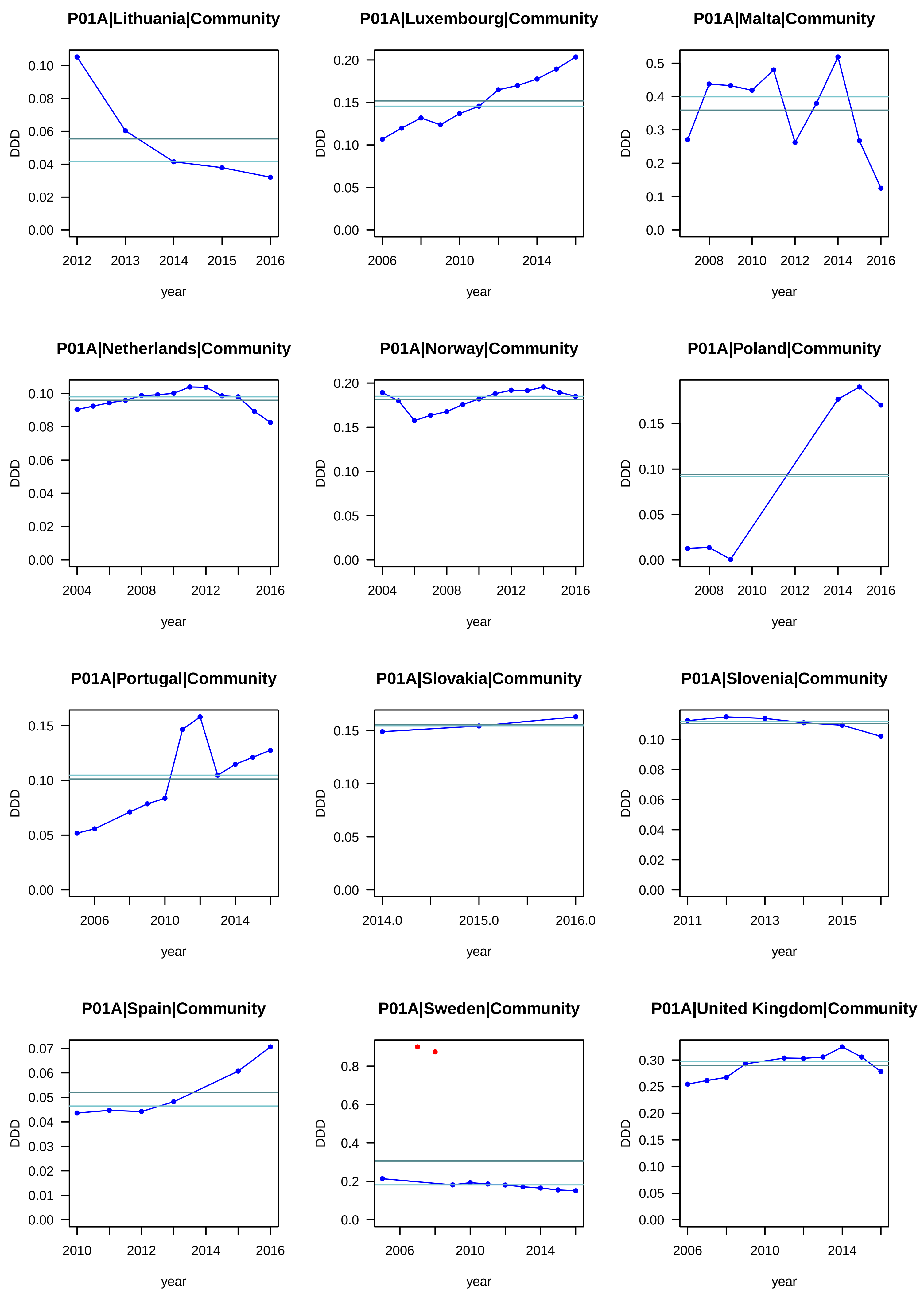
P01A|Lithuania|Community
DDD
0.00 0.02 0.04 0.06 0.08 0.10
2012 2013 2014 2015 2016
year
P01A|Luxembourg|Community
DDD
0.00 0.05 0.10 0.15 0.20
2006 2010 2014
year
P01A|Malta|Community
DDD
0.0 0.1 0.2 0.3 0.4 0.5
2008 2010 2012 2014 2016
year
P01A|Netherlands|Community
DDD
0.00 0.02 0.04 0.06 0.08 0.10
2004 2008 2012 2016
year
P01A|Norway|Community
DDD
0.00 0.05 0.10 0.15 0.20
2004 2008 2012 2016
year
P01A|Poland|Community
DDD
0.00 0.05 0.10 0.15
2008 2010 2012 2014 2016
year
P01A|Portugal|Community
DDD
0.00 0.05 0.10 0.15
2006 2010 2014
year
P01A|Slovakia|Community
DDD
0.00 0.05 0.10 0.15
2014.0 2015.0 2016.0
year
P01A|Slovenia|Community
DDD
0.00 0.02 0.04 0.06 0.08 0.10
2011 2013 2015
year
P01A|Spain|Community
DDD
0.00 0.01 0.02 0.03 0.04 0.05 0.06 0.07
2010 2012 2014 2016
year
P01A|Sweden|Community
DDD
0.0 0.2 0.4 0.6 0.8
2006 2010 2014
year
P01A|United Kingdom|Community
DDD
0.00 0.05 0.10 0.15 0.20 0.25 0.30
2006 2010 2014
year
